# Supplementary material for: Age- and Stage-Dependent Prostate Cancer Aggressiveness Associated with Differential Notch Signaling
Source: Int J Mol Sci. 2022 Dec 22;24(1):164. doi: 10.3390/ijms24010164 (PMC9820176; doi:10.3390/ijms24010164)
Supplement: Supplementary file 1 [file ijms-24-00164-s001.zip › Supplementary File S2.pdf]

Supplementary File S2. The UpSet analysis to identify specific profile of Notch signaling that correlates with poor patient outcomes.

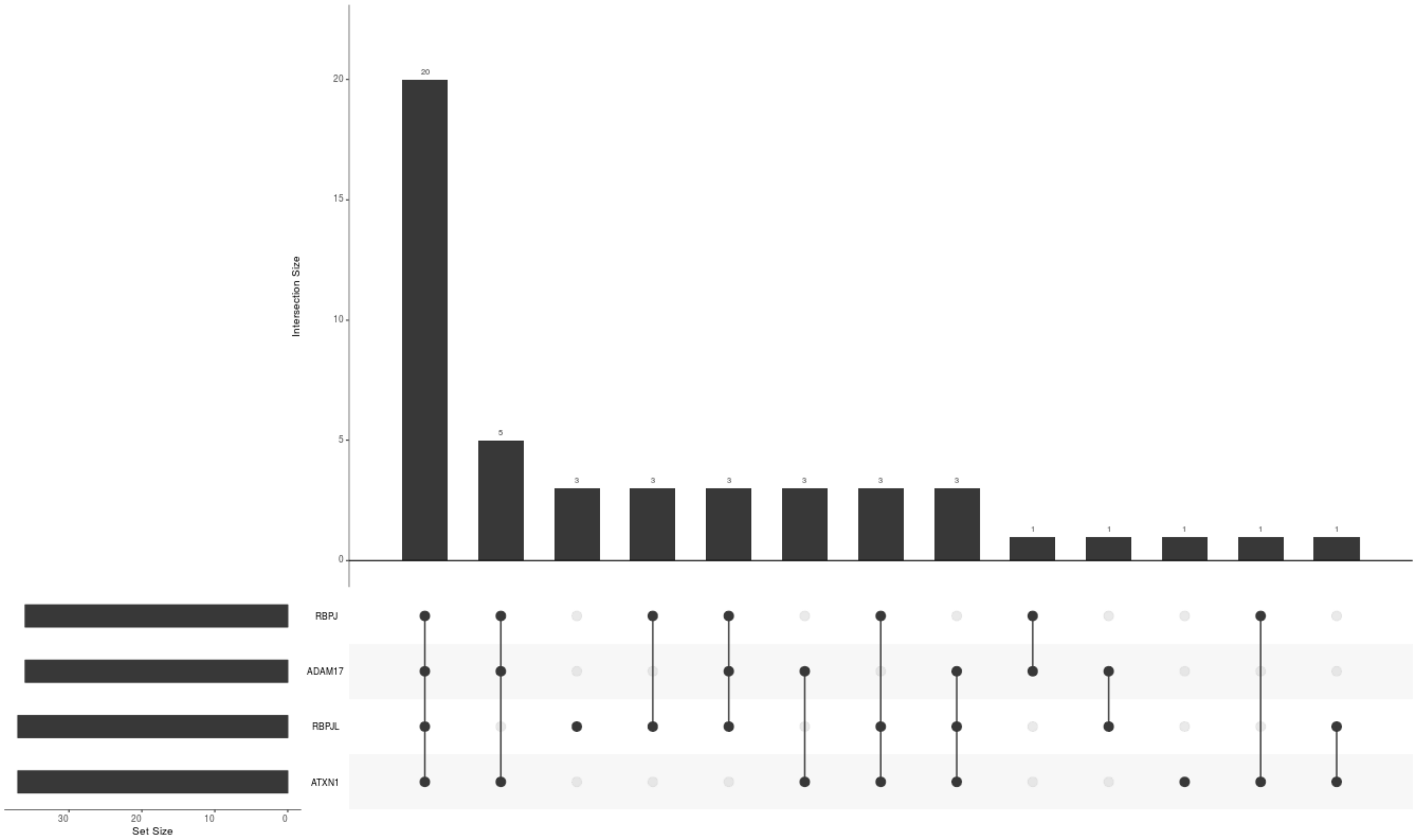

Figure S1. The UpSet analysis of the Notch core profiles associated with DFS for early stage PC in patients below 55 years old revealed the signature involving *RBPJ, ADAM17, RBPJL,* and *ATXN1*. The favorable profile of the signature was identified among 20 (37.7%) out of 53 patients.

Supplementary File S2. The UpSet analysis to identify specific profile of Notch signaling that correlates with poor patient outcomes.

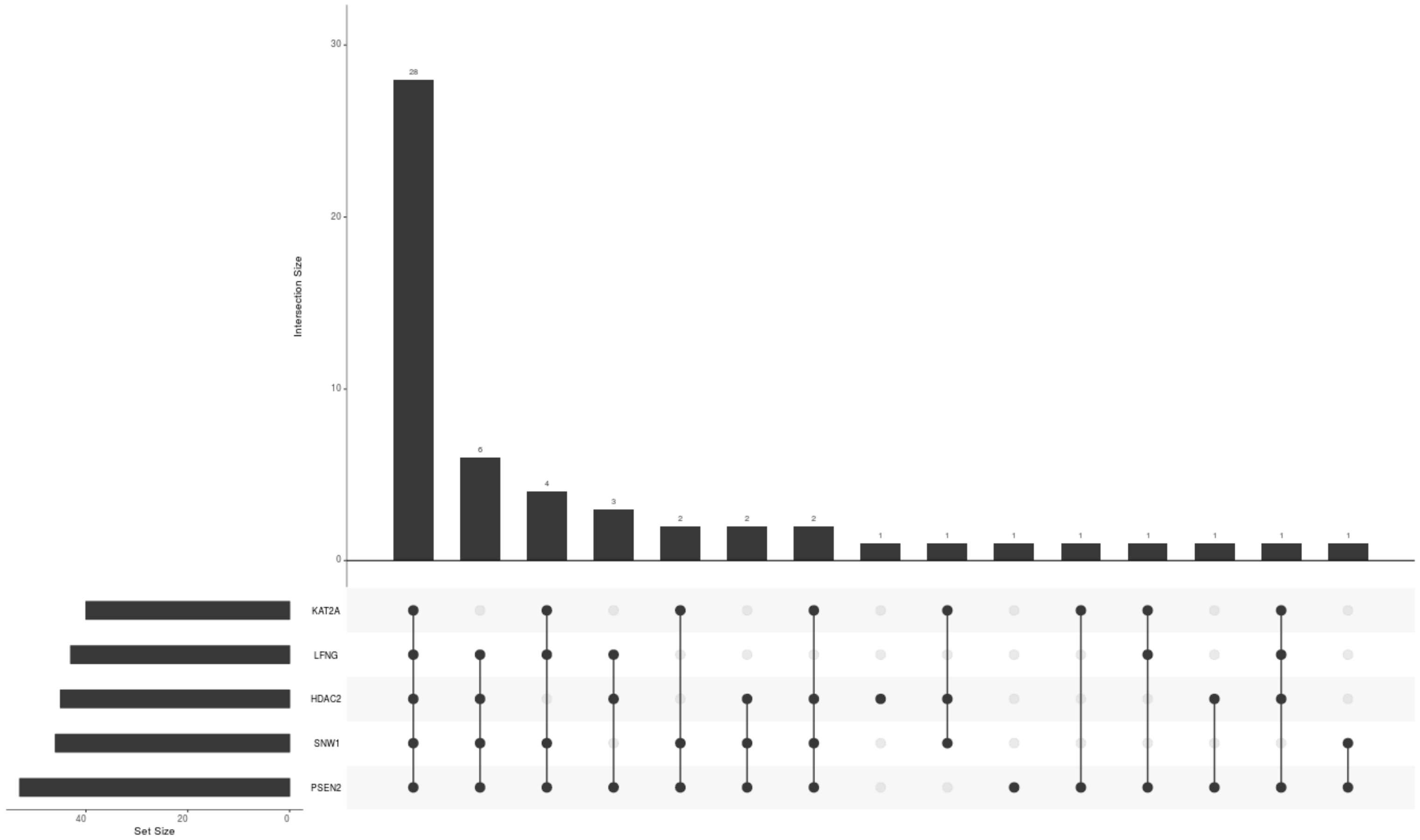

Figure S2. The UpSet analysis of the Notch core profiles associated with DFS for late stage PC in patients below 55 years old revealed the signature involving *KAT2A, LFNG, HDAC2, SNW1,* and *PSEN2*. The favorable profile of the signature was identified among 28 (50%) out of 57 patients.

Supplementary File S2. The UpSet analysis to identify specific profile of Notch signaling that correlates with poor patient outcomes.

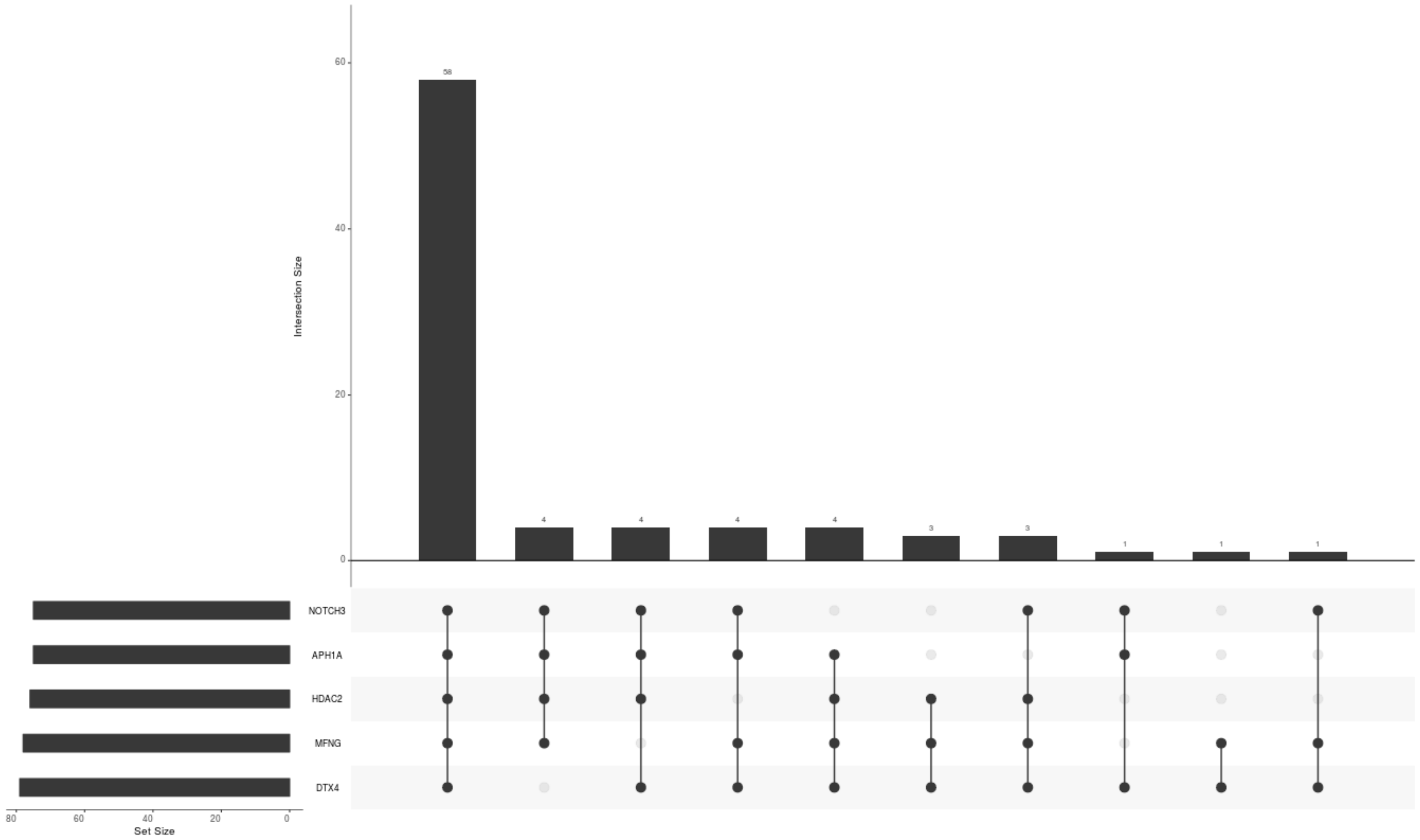

Figure S3. The UpSet analysis of the Notch core profiles associated with DFS for early stage PC in patients aged 60-70 years old revealed the signature involving *NOTCH3, APH1A, HDAC2, MFNG,* and *DTX4*. The favorable profile of the signature was identified among 58 (69%) out of 84 patients.

Supplementary File S2. The UpSet analysis to identify specific profile of Notch signaling that correlates with poor patient outcomes.

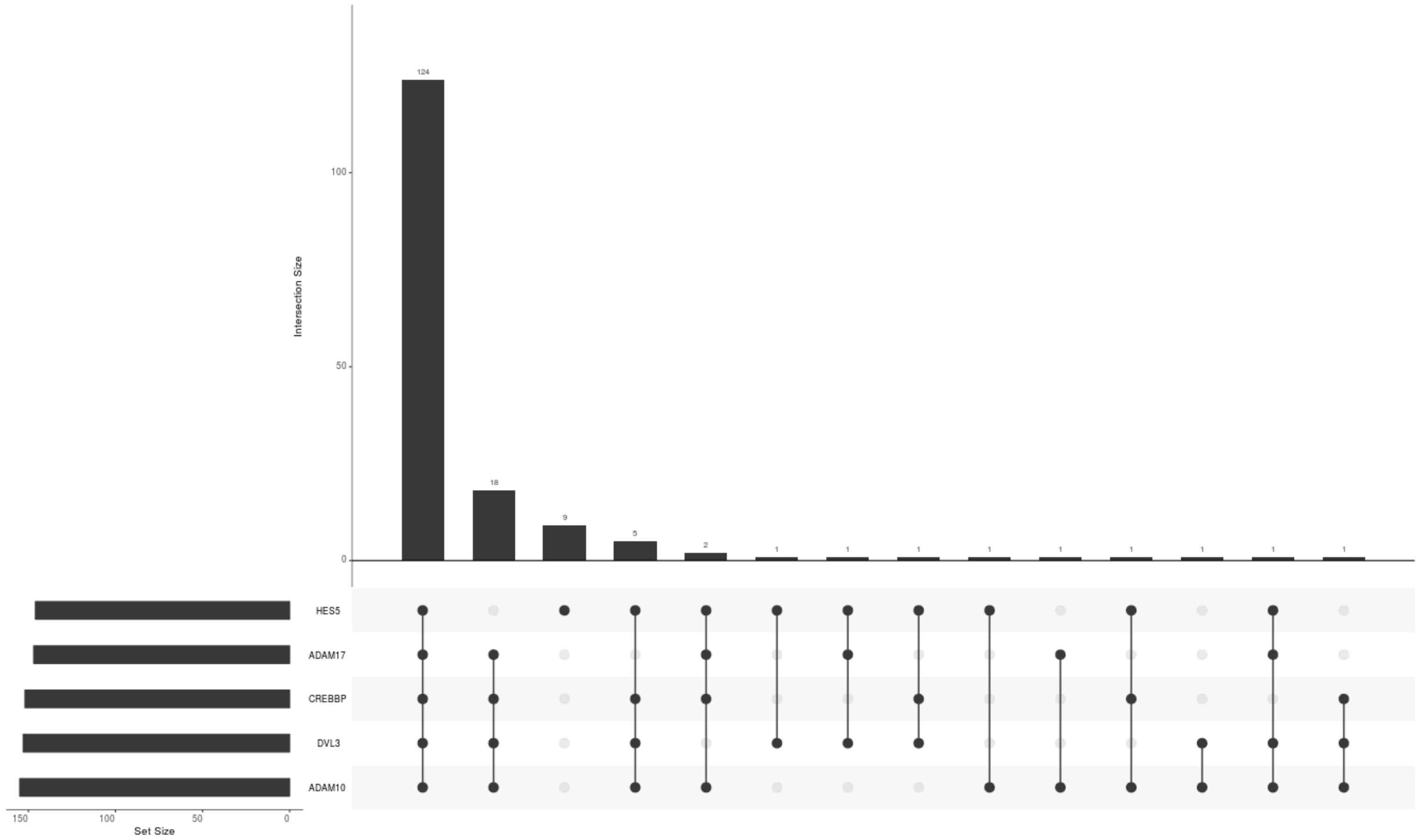

Figure S4. The UpSet analysis of the Notch core profiles associated with DFS for late stage PC in patients aged 60-70 years old revealed the signature involving *HES5, ADAM17, CREBBP, DVL3,* and ADAM10. The favorable profile of the signature was identified among 124 (73.4%) out of 169 patients.

Supplementary File S2. The UpSet analysis to identify specific profile of Notch signaling that correlates with poor patient outcomes.

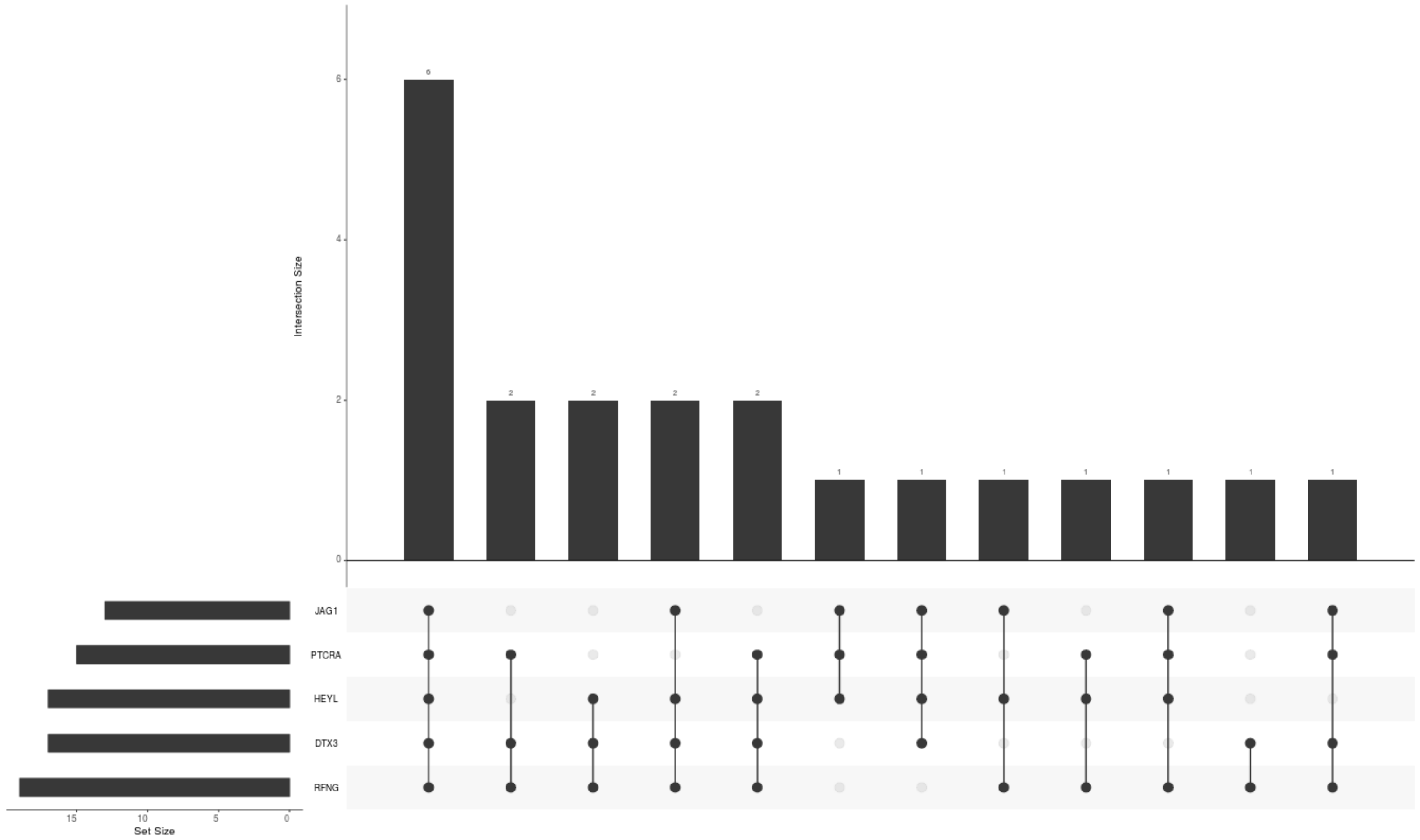

Figure S5. The UpSet analysis of the Notch core profiles associated with DFS for late stage PC in patients ABOVE 70 years old revealed the signature involving *JAG1, PTCRA, HEYL, DTX3,* and *RFNG*. The favorable profile of the signature was identified among 6 (27.3%) out of 22 patients.
