# Supplementary material for: Age- and Stage-Dependent Prostate Cancer Aggressiveness Associated with Differential Notch Signaling
Source: Int J Mol Sci. 2022 Dec 22;24(1):164. doi: 10.3390/ijms24010164 (PMC9820176; doi:10.3390/ijms24010164)
Supplement: Supplementary file 1 [file ijms-24-00164-s001.zip › Supplementary File S3.pdf]

Supplementary File S3. The ExpressCluster analysis of unique and common expression profiles among the downstream targets of Notch signaling.

I - TCGA data

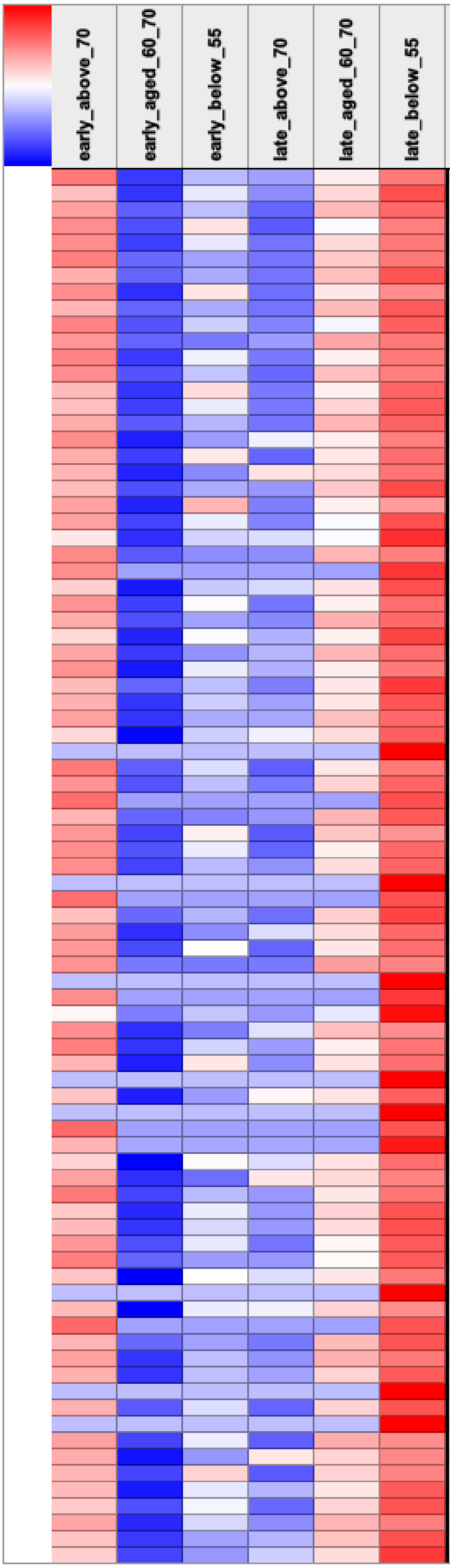

Probe ID	Descripti...	Correlation
ADD3		0.97181
APPL2		0.98714
ARHGAP5		0.96644
ATP11A		0.91187
ATP6V1A		0.96865
BCL2L11		0.94849
CAMSAP2		0.97594
CLINT1		0.92067
DCUN1D1		0.97113
EEA1		0.97924
EFR3B		0.92157
ENPP4		0.96108
HBP1		0.95812
HCFC2		0.93946
HERC3		0.97717
INVS		0.97105
KPNB1		0.91854
MSI2		0.94072
NAA50		0.85992
NEK7		0.98948
ORC4		0.85088
PANK3		0.98444
PDHX		0.93406
PPP2R5E		0.94016
PRSS3		0.86419
RAP1A		0.95686
RB1		0.96243
RB1CC1		0.96603
RDX		0.95899
RECQL		0.95796
RNF38		0.95897
SHOC2		0.98962
SPTLC1		0.99625
TBC1D23		0.97899
TPD52		0.91848
TULP2		0.70590
AMOTL1		0.95136
BROX		0.98263
GIMAP5		0.84710
ARFGEF1		0.95418
BTAF1		0.90947
CMTM6		0.96450
EXOC2		0.99017
FGL1		0.70590
GATA1		0.84731
GOLGA4		0.97765
GSTCD		0.94569
HIF1A		0.95830
HOXA6		0.89214
HOXC8		0.70590
MPP4		0.86316
MYO7A		0.92219
NBN		0.89372
OSBPL11		0.97574
PPP1R12A		0.94402
PROX1		0.70590
R3HDM1		0.90445
SCGB1A1		0.70590
SLC26A7		0.84587
SPINK2		0.86100
SPOPL		0.90489
SRPK2		0.83750
TMOD3		0.97639
UBQLN1		0.97871
VRK2		0.99310
WDR44		0.97964
WDR47		0.97322
PRPF40A		0.90427
ORM1		0.70590
SLC38A3		0.87789
CNGA4		0.84314
FANCM		0.96667
FRS2		0.96683
GFM1		0.99225
KCNK4		0.70590
KIAA0895		0.97443
KIF5A		0.70590
MFAP3		0.92361
NFXL1		0.85530
PHC3		0.89428
PTPN11		0.97565
REEP3		0.96568
RSRC1		0.96248
ZFP91		0.97591
ZHX1		0.96097

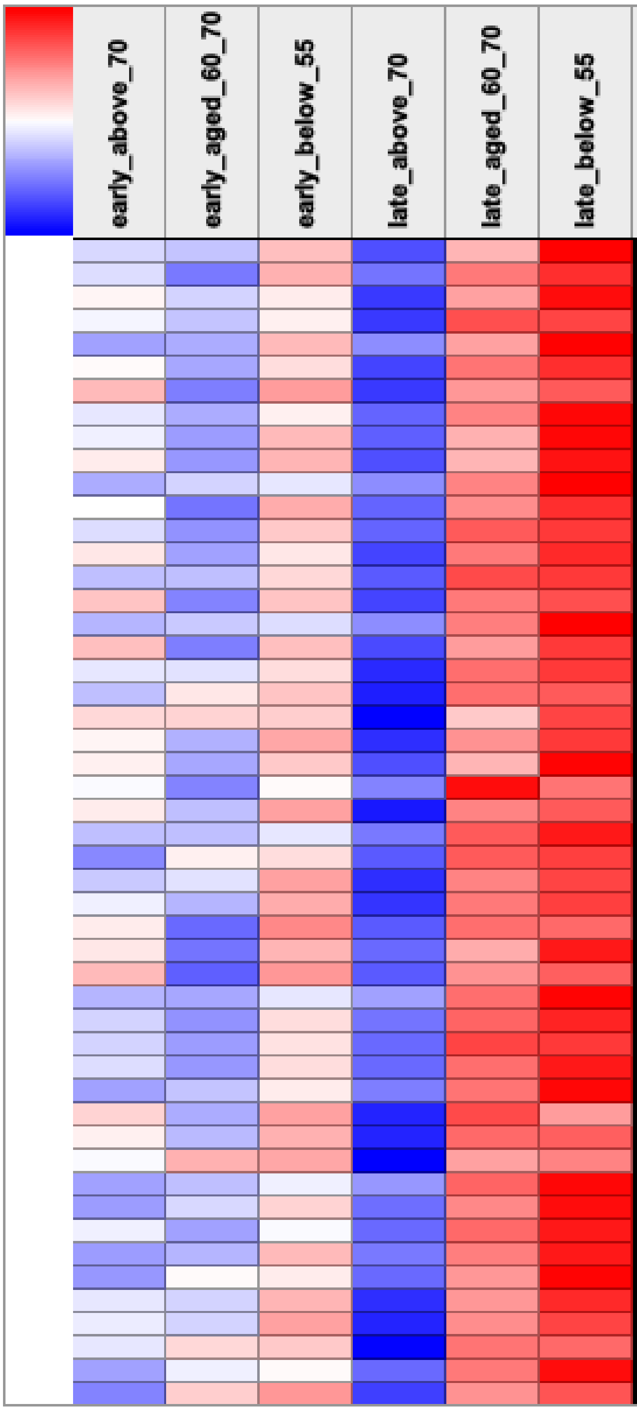

Probe ID	Descripti...	Correlation
ACSL1		0.97179
ARHGAP24		0.96868
ARHGAP29		0.97277
ATXN3		0.97985
BZW1		0.94224
CARD8		0.99101
DYNC1I2		0.91077
EGLN1		0.98637
ELF1		0.97124
FHDC1		0.96552
GNPTAB		0.92329
GRAMD1B		0.96306
LRCH1		0.98339
RAP2A		0.98241
RCOR3		0.98108
RSBN1		0.94213
SEC14L1		0.92024
SNX9		0.93531
SORL1		0.98323
TIAM1		0.92962
WDFY2		0.85342
HSPA1L		0.97809
ALDH1L2		0.96677
ALX4		0.88353
C2orf49		0.96131
CD109		0.95126
CDH10		0.91629
CYBRD1		0.97164
DCAF5		0.99057
EML1		0.91764
FBXO3		0.94357
GRIP1		0.88621
KDM6A		0.91657
LAMC1		0.97875
NID1		0.97327
PAPOLG		0.98859
PPEF1		0.95502
RNF144A		0.88869
SPARCL1		0.96462
THSD7B		0.80093
TMEM154		0.92540
ZBTB8A		0.96146
C17orf80		0.97485
CHM		0.96434
CLSTN3		0.92343
EIF5A2		0.98693
LCK		0.97395
LRP1		0.90196
MAP3K5		0.94541
RYR1		0.85558

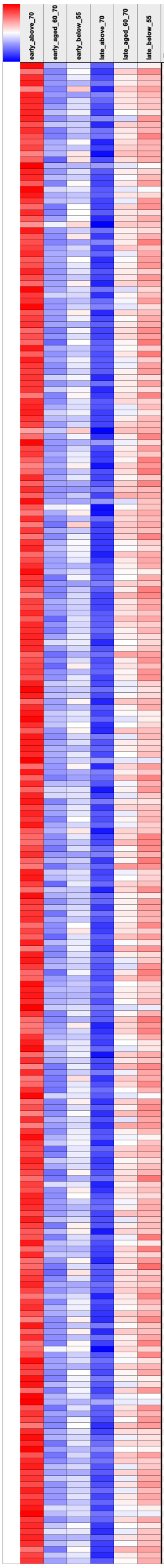

Probe ID	Descripti...	Correlation
AASDH		0.95856
ANKRD12		0.95984
ANKRD17		0.99662
ANKS1A		0.97765
AQR		0.91119
ARHGAP21		0.95497
ARHGAP32		0.98642
ARID1A		0.99751
ARID5B		0.97293
ASCC3		0.93628
ATP10D		0.97836
AVL9		0.98057
BACH1		0.95784
BAZ2B		0.97504
BTBD7		0.98735
CCDC88C		0.91751
CDC42BPA		0.95229
CDS2		0.88881
CNNM4		0.98986
CYLD		0.98864
DAAM1		0.97948
DDX43		0.91867
DICER1		0.98196
DNAL1		0.97992
DPP8		0.96439
EIF3A		0.97791
EP300		0.97964
EPAS1		0.84223
EPG5		0.97656
EXD2		0.97514
FBXW11		0.93726
FLNB		0.99274
GNS		0.98014
GOLGA5		0.97925
GORASP2		0.99045
GSPT1		0.99341
HEATR5A		0.99161
HECTD1		0.96321
HELB		0.95441
HERC1		0.97630
IQGAP1		0.97826
JMY		0.94878
KANSL1L		0.97470
KIAA1109		0.99638
KLHL18		0.98735
KLHL8		0.96718
KPNA6		0.99239
LONP2		0.94493
MFSD1		0.98265
MON2		0.96995
MYO9A		0.98229
NBEAL1		0.98869
NUP98		0.99268
OGDH		0.96529
OSBP		0.97890
PAM		0.96680
PIK3C2A		0.94820
POLR1B		0.98987
PSME4		0.98588
PTPRF		0.95157
PTPRG		0.99000
RAPGEF2		0.99470
RERE		0.84574
RNF111		0.96629
RNF24		0.93148
RNF4		0.99458
SAMD8		0.99451
SEC24D		0.97054
SEC31A		0.93030
SETX		0.96175
SLC30A6		0.99420
SMAP2		0.95417
SNX29		0.95509
SSH2		0.96757
STARD13		0.92267
SUPT6H		0.92409
TAF1B		0.94312
TBC1D14		0.98683
TBC1D15		0.92553
TBC1D5		0.99251
TMF1		0.96218
TNRC6B		0.98650
TP53BP1		0.97417
TRAK2		0.96697
TRIP11		0.99528
TRIP12		0.98757
TTBK2		0.96499
USO1		0.98105
UTP20		0.95240
VAMP7		0.96821
VPS13B		0.93912
VPS13C		0.99717
WDPCP		0.99323
WNK1		0.99346
XRN1		0.96756
ERCC4		0.99056
GTF2A1		0.98829
MTMR4		0.98516
RNF123		0.96127
SON		0.99590
TBCCD1		0.92735
AHCYL1		0.98969
ARL15		0.97103
ARL5B		0.98025
ARNTL		0.98695
CASZ1		0.97004
CHAMP1		0.95920
DYNC1H1		0.98131
FSTL1		0.96336
IPPK		0.99028
MAML2		0.96045
POLR2A		0.94261
R3HDM2		0.97078
RALGAPA2		0.97145
REL		0.97149
TRIM44		0.98926
TSR1		0.97803
ARHGAP42		0.99384
CLMN		0.87000
MGA		0.96403
ABCA9		0.96521
ACAP2		0.93144
ACTR8		0.98112
AP3D1		0.95230
ARL6IP5		0.96168
ASXL2		0.96896
ATG2B		0.99287
ATRN		0.98588
ATRX		0.98930
CALU		0.97898
CCNG2		0.95784
CEP104		0.97019
CEP350		0.95440
CHMP2B		0.97331
CNNM2		0.95830
CPQ		0.96702
DLG1		0.91209
DNAJB9		0.97179
EDEM1		0.98126
EXOC6B		0.97629
FKBP9		0.95447
GOLPH3L		0.96818
GPR107		0.98683
HERC2		0.98888
HIPK3		0.98264
HUWE1		0.99459
IREB2		0.96476
ITGAV		0.97721
KIDINS220		0.96045
KIF1B		0.97464
LANCL1		0.95285
LYST		0.98004
MBTPS2		0.98077
NCAM2		0.97103
NDUFS1		0.95539
NIN		0.96653
NOTCH2		0.97007
OGFRL1		0.97844
OMD		0.97067
PCCB		0.98948
PDE5A		0.89384
PDS5B		0.99174
PIK3CA		0.94076
PKN2		0.94865
PLEKHA1		0.96345
PRRC2C		0.99122
PRRG1		0.96156
PTPN4		0.95941
REV1		0.95848
RNASEL		0.99473
RNF217		0.93033
SAP130		0.97665
SDCCAG8		0.93814
SEC24B		0.96709
SLC30A9		0.97846
SLC9A2		0.92279
SMARCA2		0.95699
SMC1A		0.98232
SNX13		0.94252
SNX19		0.98276
SNX27		0.93810
SOS1		0.99588
TMED8		0.95592
TRPC4AP		0.97537
TSTD2		0.98114
UBR2		0.98785
UBR4		0.97297
USP46		0.99854
USP8		0.97717
USP9X		0.93248
VPS13D		0.96272
WDR11		0.97871
WDR7		0.98010
ZNF462		0.98938
HPS5		0.98016
ZC3H6		0.98605
CBX5		0.97737
ETV3		0.98151
ZNF585B		0.96179
ARHGEF12		0.95811
PI4KA		0.97004
AKAP13		0.95862
ANO6		0.97999
ATR		0.96256
BCORL1		0.95374
BTC		0.95729
CLPX		0.99692
CSNK1G1		0.96796
CYB5RL		0.98244
DDB1		0.95524
DDI2		0.99502
DENND4A		0.98713
DENND5B		0.98609
EFCAB7		0.94500
ERN1		0.97701
FUT11		0.96975
HIPK1		0.98548
HMGXB4		0.95511
HNF4G		0.90291
LAP3		0.98322
LMBRD2		0.97035
LMLN		0.98214
LTN1		0.98923
M6PR		0.95336
MAN1A2		0.96729
MIER1		0.97029
MIOS		0.94362
NUDT16		0.86252
PBRM1		0.97528
PCYT1A		0.97843
PDCD6IP		0.98707
PLEKHM3		0.95406
PRR14L		0.98809
RAD54L2		0.96728
RAPGEF6		0.97293
RGP1		0.92353
RUFY2		0.97460
SENP2		0.96838
SIK3		0.97860
SLC12A6		0.97788
SLC30A7		0.97795
SOCS4		0.99194
SRP72		0.98309
TAP1		0.99783
TBC1D9B		0.99138
TECPR2		0.94514
TRIM56		0.91862
UBXN7		0.98227
ZDHHC5		0.96735
ZDHHC7		0.95558
ZFP1		0.98159
ZNF490		0.98882
ZNF689		0.94931
ZNF774		0.98351
ZYG11B		0.99454

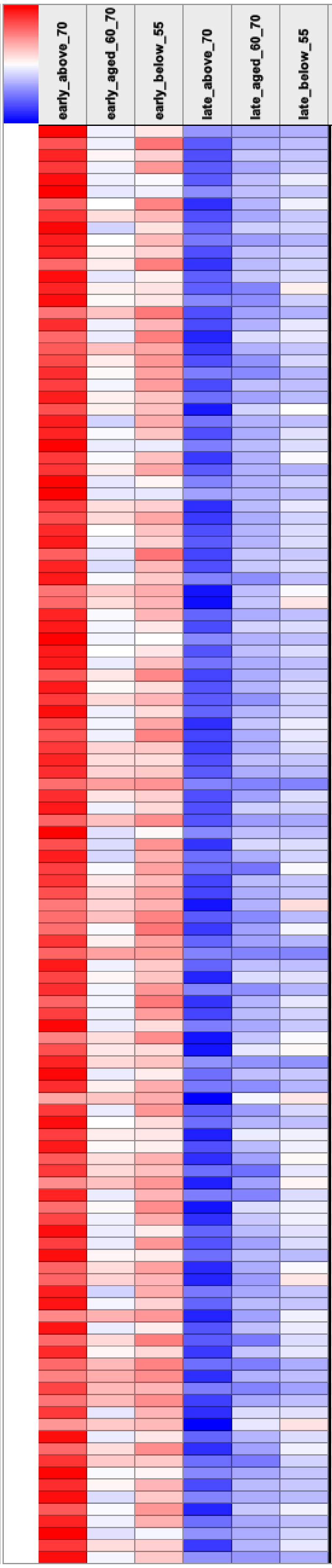

Probe ID	Descripti...	Correlation
ACOXL		0.92954
APP		0.96023
ARHGEF7		0.99190
C16orf70		0.98827
CCT6B		0.95217
DMGDH		0.90743
DNAH7		0.95937
EGF		0.99610
EPM2A		0.95422
ESRP2		0.97873
FGFR2		0.99144
FRMD4A		0.95899
GLG1		0.95899
GOT2		0.96129
GSTO2		0.95585
IVD		0.93250
LMO7		0.99479
LRIG1		0.93701
NAGLU		0.97008
NANS		0.98674
NCAPD3		0.97212
NEDD9		0.98815
NFATC3		0.98629
NRL		0.96205
PDCD4		0.97059
PDE11A		0.99588
PLCL1		0.91951
PLOD1		0.98836
PTPRN2		0.98896
RAB27A		0.94393
SLC1A1		0.88344
SLC38A10		0.98508
STXBP2		0.98848
TACC2		0.99719
TXNDC11		0.98833
TXNDC15		0.95142
ZFHX3		0.98278
CHMP7		0.96868
CTDP1		0.93790
OS9		0.93318
MLYCD		0.99335
PDLIM5		0.97044
TAPBPL		0.93498
TIPARP		0.98167
ZNF395		0.98069
ST14		0.97824
ACADL		0.98881
ACP2		0.99083
AFF3		0.97841
AGA		0.98311
ASTN2		0.97265
BTBD11		0.99060
CAMK2B		0.98592
CBFA2T3		0.98692
CBLN2		0.86432
CC2D2A		0.99421
CD44		0.98097
CYP27A1		0.94383
DHX32		0.92938
DNAJC10		0.96017
FAM13C		0.97925
FMO4		0.96405
FMO5		0.99629
GCAT		0.98414
GFRA1		0.91896
GLB1L2		0.93863
GOLM1		0.93961
HLF		0.98849
KCNH1		0.87941
KIAA0319L		0.98492
LCP1		0.97402
MAMLD1		0.96576
NAAA		0.95659
RNF185		0.99053
SCIN		0.96708
SCN7A		0.92439
SDK2		0.93622
SLC38A4		0.93823
SLC7A8		0.96052
SMPD2		0.97507
TRAF3IP2		0.80809
TTC12		0.98510
UNC80		0.97728
USP40		0.94574
WFS1		0.97225
HEATR4		0.97070
IL31RA		0.96261
P4HB		0.90162
ALDH7A1		0.97119
ANXA3		0.93460
ARFIP2		0.97959
ASMTL		0.96743
CENPN		0.98533
DENND2D		0.96781
GALNS		0.96648
GPRC5C		0.94614
GREB1		0.97075
LMAN1L		0.98282
LMAN2		0.91254
MAN2B2		0.96740
MICA		0.94443
MPI		0.98574
MSRA		0.91738
PER1		0.91893
PHYHD1		0.94239
PICK1		0.94072
SATB1		0.97404
SEMA4B		0.84606
SEMA4G		0.97227
SGSH		0.96064
SLC22A18		0.96883
SMPDL3A		0.94435
SPINT1		0.99770
SUOX		0.96105
TCEAL3		0.96728
TSPAN1		0.98692
VGLL3		0.91254
XYLT2		0.98871
YIPF1		0.95117

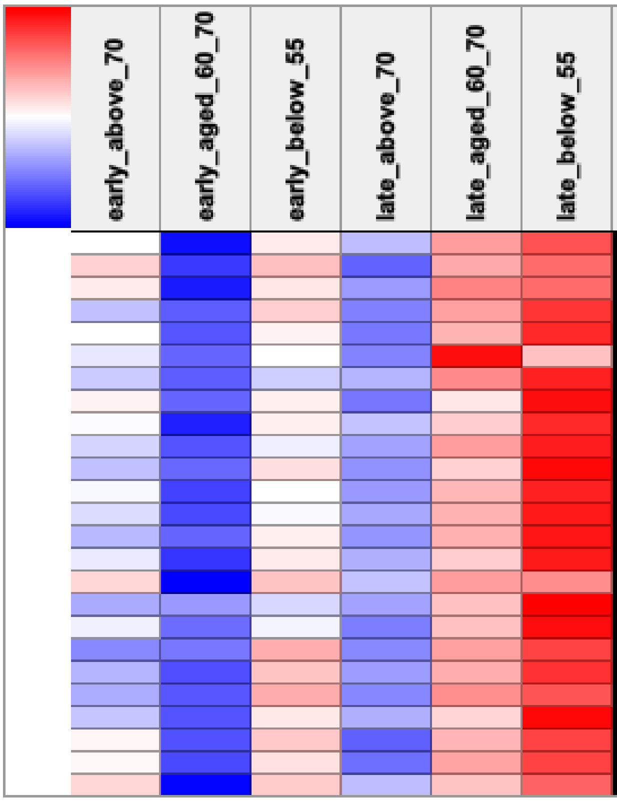

Probe ID	Description	Correlation
BLZF1		0.97057
CLDN12		0.93440
G2E3		0.95768
LRIF1		0.98523
MAP4K3		0.98962
NKTR		0.78342
RAB21		0.95419
SESN1		0.95651
TANK		0.97046
UHMK1		0.98544
UPRT		0.97147
WWC2		0.99271
ACTR2		0.98996
ATF1		0.98105
FAM199X		0.98525
IMPG2		0.89113
LPGAT1		0.90416
MOB1A		0.97420
SENP6		0.92545
SMAP1		0.97975
TWSG1		0.95296
GLIPR1L2		0.97278
TMEM135		0.97417
TOP1		0.98591
TRUB1		0.92937

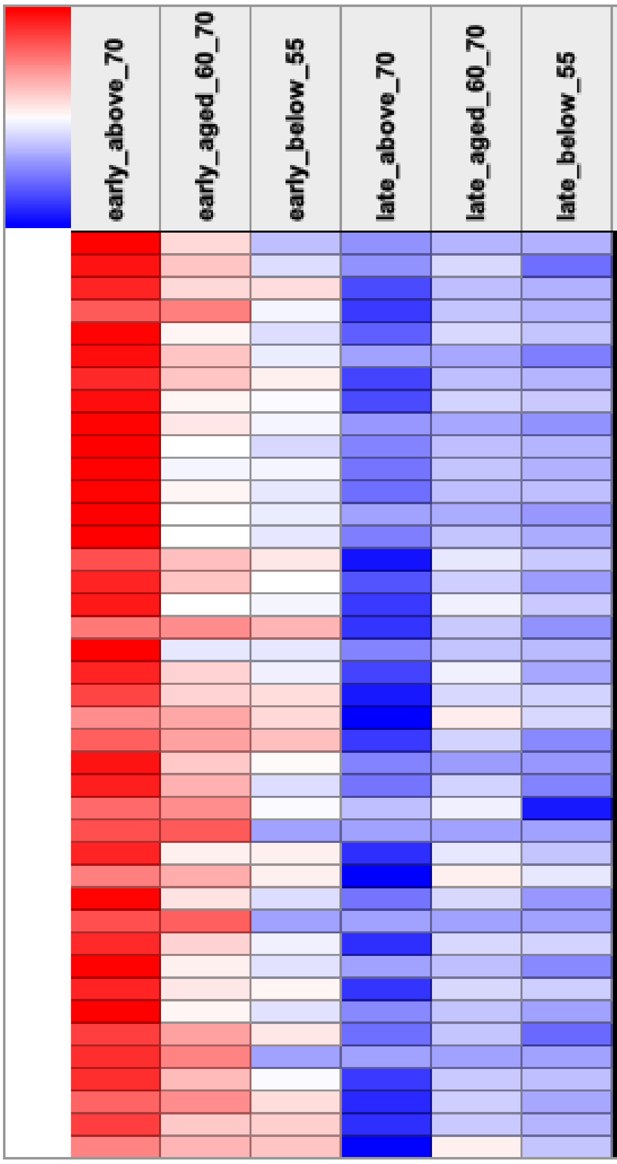

Probe ID	Description	Correlation
CSRNP1		0.95191
DTNB		0.96026
ETV2		0.98558
GADD45G		0.95041
LPCAT3		0.97131
NR4A1		0.95161
PLEKHA6		0.99319
PTPRS		0.97878
ZNF18		0.95703
ZNF215		0.95271
SPTBN4		0.96678
ADHFE1		0.97379
AKAP3		0.93628
BOC		0.96467
C7		0.95253
CAMKMT		0.99947
CBX7		0.96069
CHST8		0.90281
CNKSR2		0.94051
CRTC1		0.98922
DGCR2		0.95141
EMILIN2		0.83251
FAAH		0.93922
GALM		0.97628
IQSEC3		0.98253
MUC5B		0.78571
MYBPC2		0.84267
PGR		0.96897
PML		0.85320
SEMA3F		0.98100
SLC4A10		0.84661
ALG14		0.97930
GGT7		0.94480
ITGA2		0.97457
PAOX		0.96338
PHLDB3		0.95854
PIWIL2		0.89029
SLC25A35		0.99002
SPG7		0.94016
TMEM125		0.96983
TMEM198		0.86056

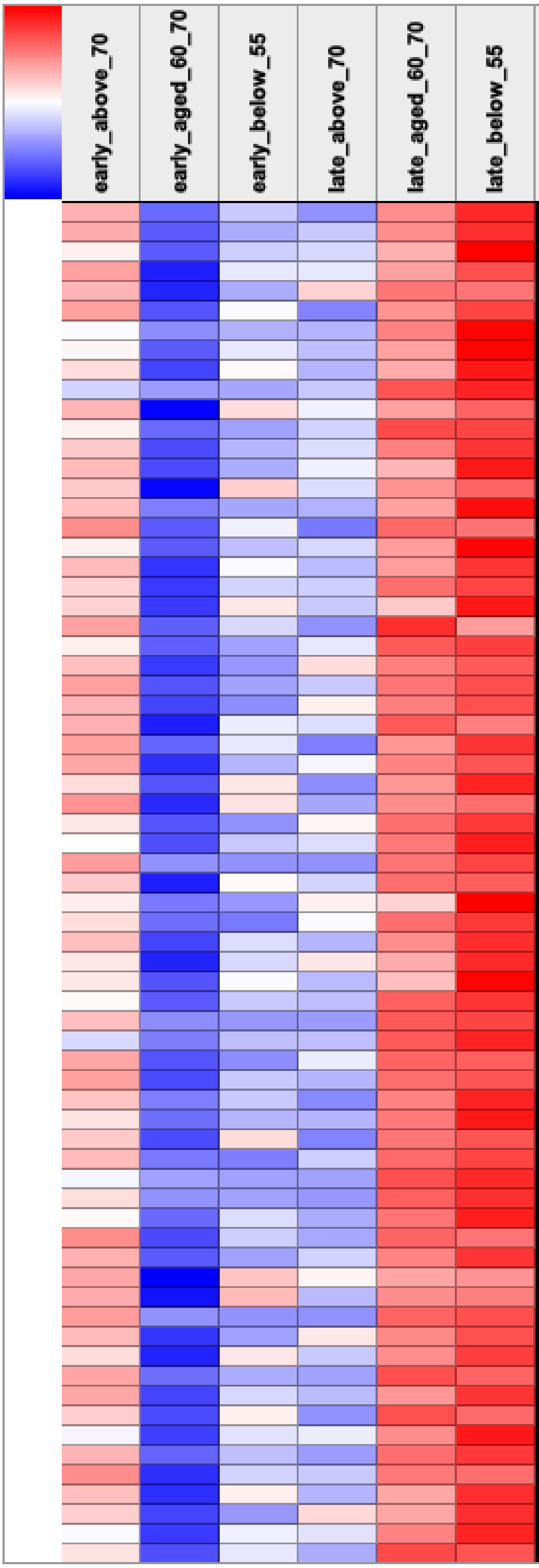

Probe ID	Descripti...	Correlation
ACER3		0.97769
ADAM17		0.98787
CALCOCO2		0.96145
CDK8		0.96500
CEP85		0.91529
DIP2B		0.95302
FAM126A		0.94519
FMR1		0.96343
FUBP3		0.96536
GABPB2		0.89739
GMCL1		0.91411
KLHL24		0.96340
LMBR1		0.99489
NEK4		0.96131
PDCD10		0.91219
RABGAP1L		0.96103
SMG1		0.92521
ST3GAL1		0.97234
STAG2		0.97925
TAF2		0.99560
TBCEL		0.93427
TNIK		0.90024
UBE3C		0.96664
VGLL4		0.93347
VCPIP1		0.97773
XPOT		0.94584
ADNP		0.95149
AHCTF1		0.95219
ANKIB1		0.97203
CCAR1		0.94499
EAF1		0.92452
GAB1		0.95584
GNA13		0.97811
GUCY2C		0.93067
HLTF		0.96545
IL17RD		0.88620
MTDH		0.93841
NCK1		0.99428
NEDD1		0.94341
PGK1		0.94707
PHF20L1		0.98034
PKD2		0.94656
RALGPS1		0.93411
RBL1		0.95395
RCOR1		0.98687
RRM2B		0.96360
SLC25A24		0.98457
STAG1		0.93693
STK4		0.95388
TAC1		0.91462
TRAM1		0.94691
JRKL		0.97417
NR2C2		0.95653
PPP4R2		0.98487
BMI1		0.82809
CASP8AP2		0.88276
DDX39B		0.92727
FOXN2		0.95223
GOLIM4		0.95902
HOXC6		0.95802
JAG1		0.98879
JMJD1C		0.94495
OCLN		0.96201
OTUD4		0.98378
PAWR		0.96325
PRDM10		0.96393
RRAGC		0.92790
ZBTB11		0.96926
ZBTB6		0.97211

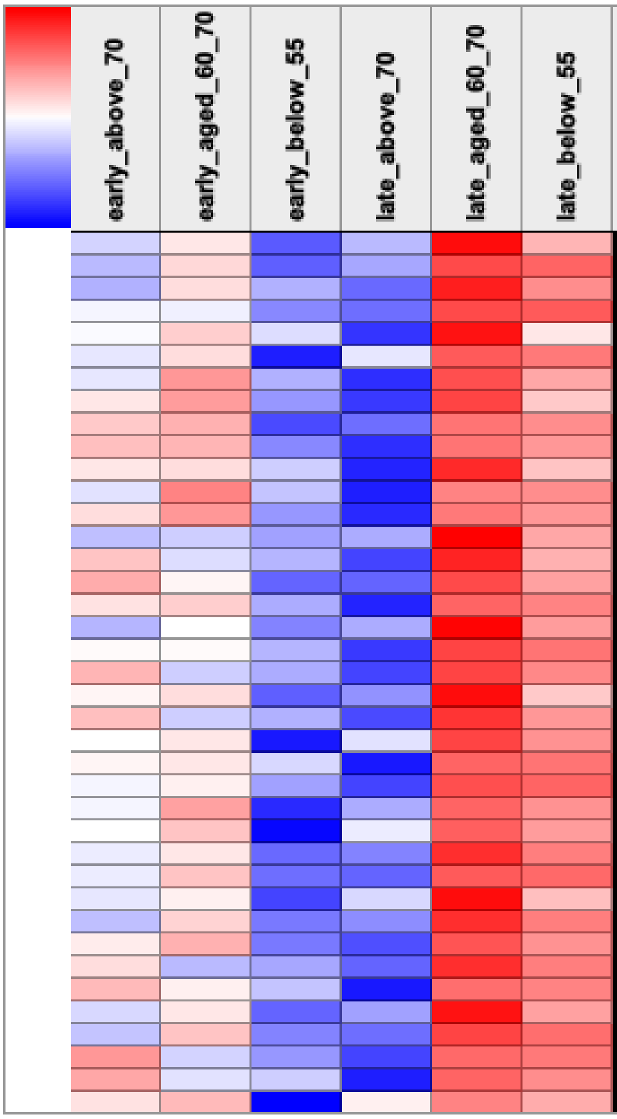

Probe ID	Description	Correlation
CERK		0.92289
GLT8D2		0.93568
PLEKHH2		0.94602
PREX1		0.97269
SLCO1A2		0.88436
TMCC2		0.85872
TRIM22		0.93330
UNC5B		0.94207
MVP		0.94674
NOTCH4		0.94194
PLEKHG2		0.92442
SLC1A7		0.87089
ELFN1		0.92406
ADAM19		0.88480
COL21A1		0.93585
DOCK6		0.95879
GRAMD1A		0.95369
ISM1		0.92115
JAM2		0.96747
KSR1		0.92593
NLRP1		0.95787
OSBPL6		0.93079
PDGFRB		0.87184
RASGRF1		0.91607
RHOJ		0.97400
SLFN13		0.90839
ST6GAL2		0.82551
TIMP2		0.99011
USHBP1		0.98223
XAF1		0.88835
COL18A1		0.96385
TGM2		0.98068
ZNF521		0.92902
GPR146		0.90659
NBEAL2		0.96061
SLCO3A1		0.96678
TGFBR2		0.90054
TMEM63C		0.87817
TRPV2		0.71980

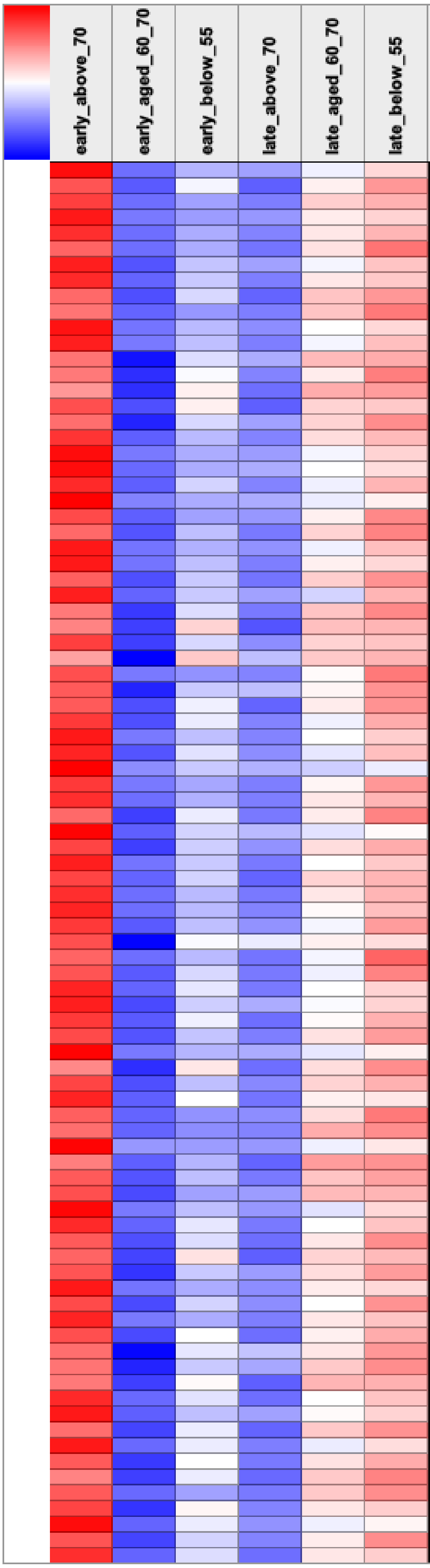

Probe ID	Descripti...	Correlation
ABL1		0.96181
AKAP10		0.97756
AKAP9		0.98111
ATP13A3		0.96199
DNAJC13		0.98678
GCLM		0.95980
GNAQ		0.98314
HIVEP1		0.99353
ITCH		0.97483
JAK2		0.94078
KAT6A		0.97060
KIF2A		0.98023
KIF3A		0.94255
LYPLA1		0.94969
MAPK8		0.90263
METTL14		0.96291
NAA25		0.96225
NCOA2		0.99510
NCOR1		0.96152
PANX1		0.95755
PARP8		0.99175
PTPN21		0.92087
PUS7L		0.97504
RBM27		0.97483
RC3H2		0.97300
ROCK2		0.97687
RSF1		0.98400
SLC38A1		0.96828
SP4		0.95865
SPATA5		0.90396
STAT1		0.99156
STEAP1B		0.81930
TCEANC2		0.95325
THUMPD3		0.96227
TRAPPC8		0.98034
UEVLD		0.98809
UHRF1BP1		0.97807
UHRF1BP1L		0.98398
USH2A		0.86863
USP25		0.98023
ZNF41		0.99026
ZNF800		0.97066
CDK12		0.92340
DDX21		0.99501
STT3B		0.98378
ATP2A2		0.98959
SOWAHC		0.99297
TMCC1		0.98703
BBX		0.99003
CCL28		0.88080
CDC27		0.95219
CLTC		0.97661
DOCK7		0.98208
EPB41L5		0.97359
FBXO21		0.99045
FILIP1		0.99655
GUCY1A2		0.92742
HACE1		0.92448
L2HGDH		0.99601
MARVELD2		0.96399
MED13		0.95712
MMRN1		0.93714
MYH10		0.92034
NEK1		0.93232
NPAT		0.98451
PEX1		0.97058
PIK3CB		0.95381
PPP2R3A		0.98704
RIF1		0.98558
SLC35F5		0.94819
SLK		0.98588
CTNNB1		0.96926
LRRC58		0.99177
SLC5A3		0.98015
ZNF91		0.98232
C5orf51		0.93012
DCAF12		0.95642
EIF2AK2		0.94110
FBXL14		0.98580
GTF3C4		0.97673
INTS2		0.96585
LIN52		0.96674
NSUN3		0.97983
PPTC7		0.94834
SOWAHB		0.96898
TOPORS		0.97173
WIPF2		0.94752
ZNF148		0.98993
ZNF791		0.98795

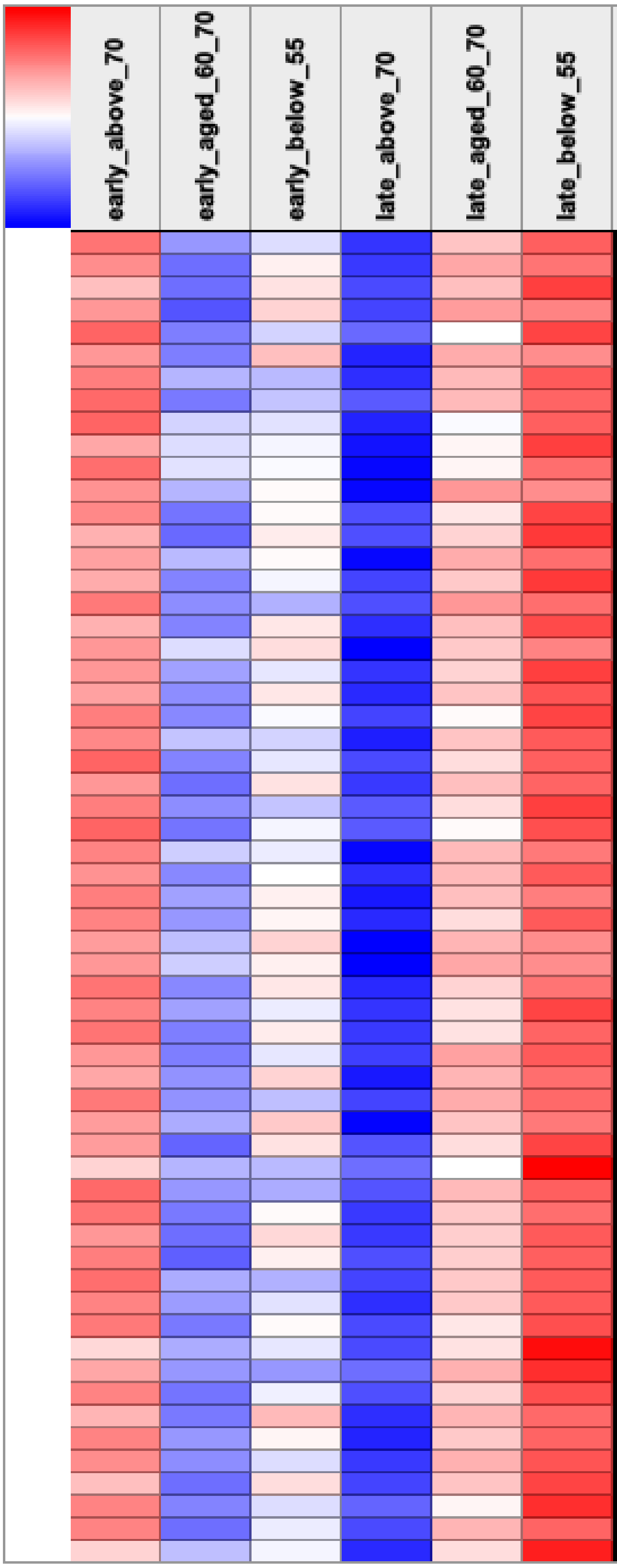

Probe ID	Descripti...	Correlation
ANAPC1		0.99232
ARID4A		0.98198
ARID4B		0.96815
BDP1		0.94719
CPEB4		0.95536
DCAF17		0.94782
DGKH		0.97049
DHX8		0.97021
DNM2		0.95063
FAM214B		0.94863
GBP3		0.94213
IKZF2		0.95456
IPMK		0.98674
KIF21A		0.97451
LIMA1		0.97178
LIMCH1		0.98909
MIS18BP1		0.94988
NCOA1		0.98634
NMT1		0.93238
NPC1		0.99435
POLR2B		0.99300
PTCD2		0.98195
PTPRK		0.97624
RAB3GAP2		0.98315
SBF2		0.98626
SERAC1		0.97380
SLC25A16		0.96825
SLC2A13		0.96366
SOS2		0.99783
SPTBN1		0.98382
STXBP3		0.99544
TEC		0.94239
UBE3B		0.94105
USP16		0.98775
USP53		0.99286
DNAJB14		0.98857
AKAP6		0.98759
CCDC6		0.97711
EIF4G3		0.97408
FAM214A		0.96136
FNBP1L		0.97250
GRHL1		0.87267
IDS		0.95516
KTN1		0.99041
LRP12		0.98315
NCKAP1		0.97909
OSBPL1A		0.96390
PER3		0.99678
RANBP9		0.98634
SLC10A7		0.93521
SPAG9		0.91858
UBE3A		0.99058
WDFY1		0.95653
CPSF2		0.99625
CNST		0.99234
KLHL28		0.96794
RNF11		0.96727
TAOK1		0.98842
TPM4		0.94870

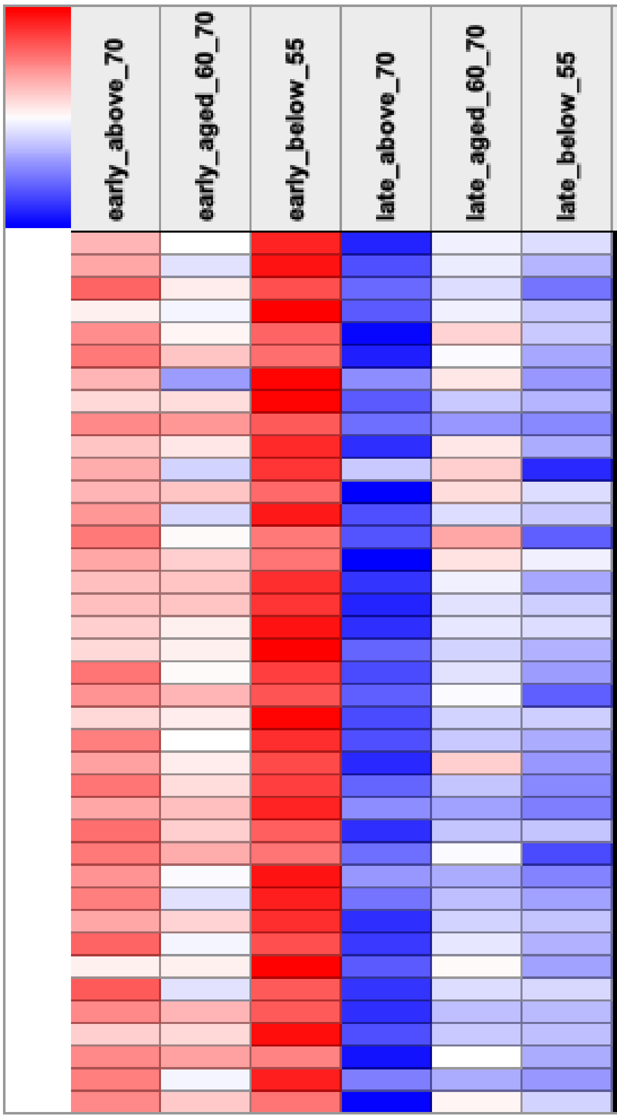

Probe ID	Description	Correlation
ATP2B4		0.97140
CAB39L		0.97878
CREB3L4		0.95848
DGKG		0.92933
GRTP1		0.94304
IL20RA		0.96345
KIAA1549		0.87038
MYH11		0.95934
SOX9		0.90973
VIPR1		0.97871
SIM2		0.77816
ARID3B		0.91853
ING1		0.97006
TSSK4		0.88038
TNS1		0.90343
AGTR1		0.98620
ARHGAP20		0.97255
ARHGEF4		0.96182
CUX2		0.95805
FAM189A2		0.98873
GRIA3		0.94720
ITIH5		0.95756
MYOCD		0.98778
NEK3		0.96863
NRK		0.97602
PGM5		0.92898
PITPNM3		0.96266
RNF157		0.90320
SLC22A3		0.93313
NQO1		0.96186
NSUN6		0.98882
MAGI2		0.97296
SLC25A21		0.94245
CRELD1		0.94389
ELL3		0.96380
FZD9		0.97075
KCNJ11		0.93095
PDZRN4		0.95317
RORC		0.94128

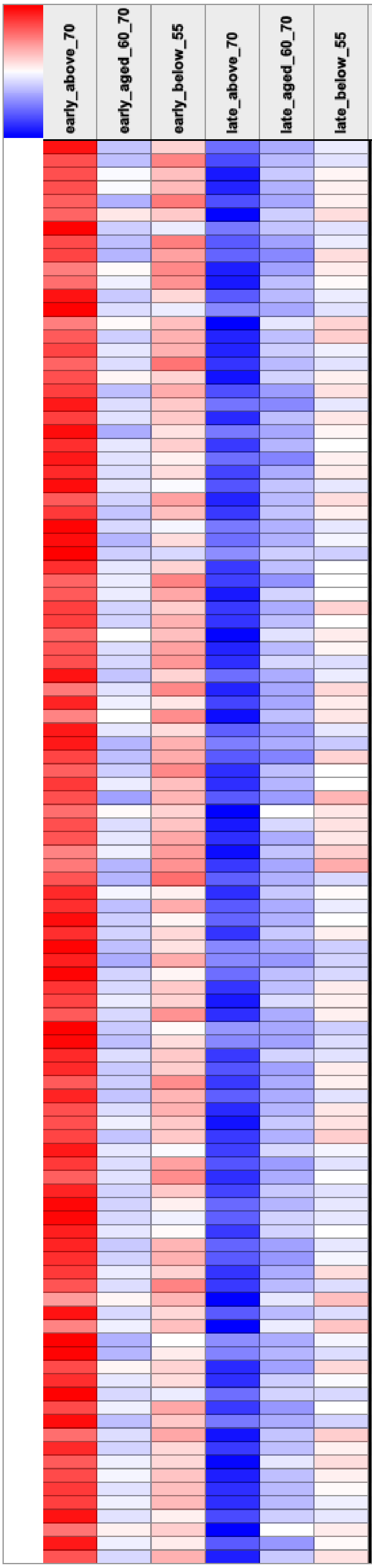

Probe ID	Descripti...	Correlation
AACS		0.97330
ALG6		0.96253
APH1B		0.98057
B4GALT6		0.98627
CDH1		0.94949
CGNL1		0.94432
COBL		0.92233
COG3		0.95625
DHCR24		0.96192
DNAJC1		0.93598
DNAJC3		0.95988
EEF2K		0.98366
EHHADH		0.91177
FGF2		0.91344
GPR63		0.97388
GTF3C1		0.98837
HEXB		0.94918
HIVEP3		0.97007
IQGAP2		0.98362
LDLR		0.96441
MANBA		0.99409
NEDD4		0.95606
NEO1		0.99794
NIPAL3		0.95291
PAFAH2		0.99233
PCDH1		0.95950
PCYOX1		0.98019
PIGB		0.99528
PLIN3		0.93878
SCARB1		0.96922
SLC4A4		0.86942
SORD		0.99616
SREK1IP1		0.95371
TM9SF1		0.97561
UGGT1		0.98736
VEGFA		0.99661
PPIL2		0.95238
RBM47		0.98528
SLIT1		0.96935
AACS		0.97330
ABCC4		0.94788
ACOX3		0.98269
ACSS3		0.93050
ALDH3A2		0.97956
ASAH1		0.94629
B4GALT1		0.96509
BBS4		0.97041
C20orf194		0.99737
CDKL2		0.94407
CECR2		0.92113
CLCC1		0.98165
CTBS		0.98444
EPN3		0.93170
FRK		0.91873
GPLD1		0.93334
KIAA0513		0.97792
LIFR		0.98467
MAP2K4		0.96945
MGAT4A		0.99413
MTMR9		0.93323
NEDD4L		0.93558
PLA2R1		0.94823
RABEP1		0.99740
RHBDD1		0.97687
RPN2		0.97593
SLC2A12		0.90583
SLC39A14		0.94164
SLC7A2		0.98967
SSR1		0.99009
STT3A		0.97279
TCTN3		0.98209
TMEM59		0.99029
TTC39A		0.97854
ZBTB16		0.98340
ZC3H12C		0.96463
TMEM232		0.98293
CALR		0.97119
CCPG1		0.99038
FXR2		0.96037
LRRC28		0.94292
SYT7		0.97302
TSPYL1		0.98014
ACOX1		0.98835
ALDH1A3		0.98967
ATMIN		0.96215
C2orf16		0.86661
CANT1		0.97828
CNNM3		0.90869
DHTKD1		0.91973
DMXL1		0.93223
EIF5B		0.97376
ERMAP		0.98946
KCND3		0.92492
PCCA		0.98413
PDP2		0.95790
PIGG		0.95807
PIGT		0.99541
RTN4RL2		0.95857
RUNDC1		0.98672
SLC39A7		0.99873
SLC44A4		0.99362
SLC9B1		0.97523
SYVN1		0.90503
TK2		0.97347
TMED10		0.98493

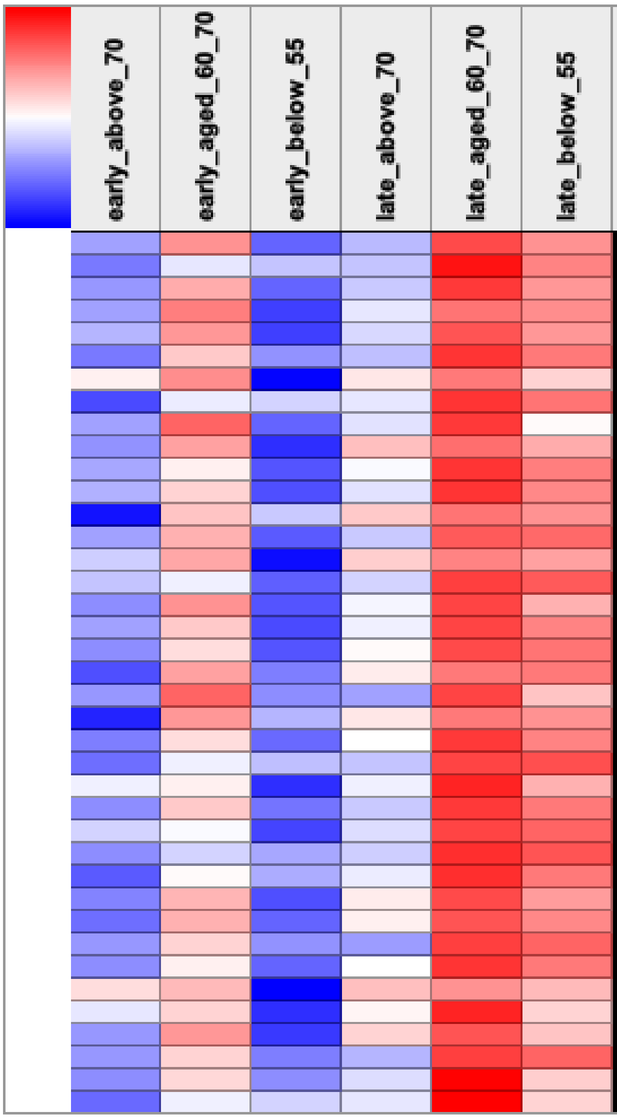

Probe ID	Description	Correlation
ADAM8		0.96988
CDH13		0.89197
GAD1		0.98968
KIFC3		0.94927
MMP2		0.96871
PHLDB1		0.97257
ZFP36L2		0.79711
ADAM23		0.86648
ARHGAP22		0.88141
BATF3		0.92311
DOK5		0.98298
ENG		0.99004
NALCN		0.81713
PCDHB7		0.97771
RAB38		0.88732
SGIP1		0.93918
ST6GALNA...		0.97617
GLI1		0.99403
ADAMTSL2		0.98938
FLT3LG		0.93930
SCARF2		0.89137
STAC3		0.86235
VIM		0.99193
ABTB2		0.89206
DENND4B		0.91289
DNM1		0.98951
ESAM		0.93886
F2R		0.89805
FZD2		0.93771
GAS6		0.98565
LAMC3		0.98387
LTBP3		0.93995
MTMR11		0.98430
NXNL2		0.74205
PDE4C		0.90632
PODNL1		0.93910
PRELP		0.97014
SEMA3G		0.94249
TTLL3		0.84247

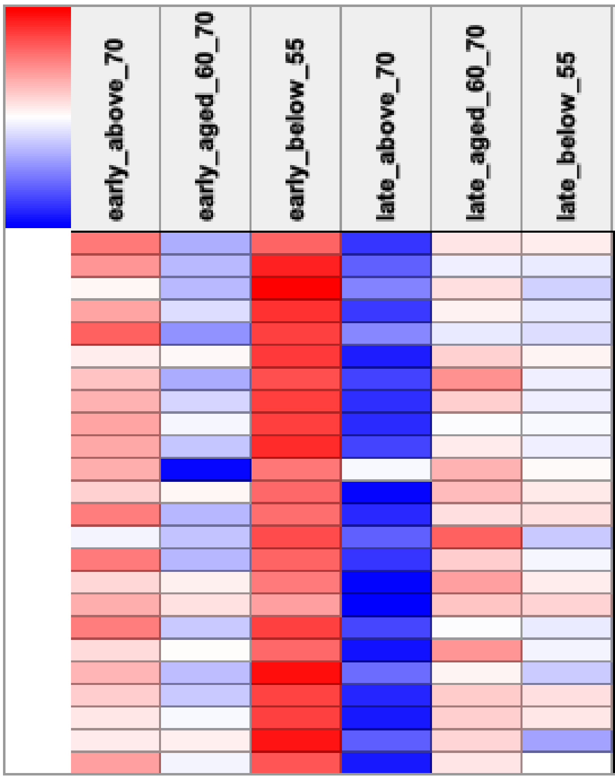

Probe ID	Description	Correlation
ANK3		0.96109
GNAL		0.95589
HOXA9		0.90493
SORBS1		0.98878
TXNDC16		0.87517
MSRB3		0.95720
MYRIP		0.96648
KLF8		0.99933
MCEE		0.97394
PDE3B		0.99182
PMFBP1		0.56136
AMACR		0.94641
FUT4		0.95698
AFAP1L2		0.86124
ANKRA2		0.97368
CCDC30		0.91765
HENMT1		0.87539
LDLRAD3		0.96734
NHLRC1		0.93930
PHOSPHO2		0.95312
PLEKHH1		0.98375
SAMD5		0.96303
SEC11C		0.89955
ZNF785		0.98114

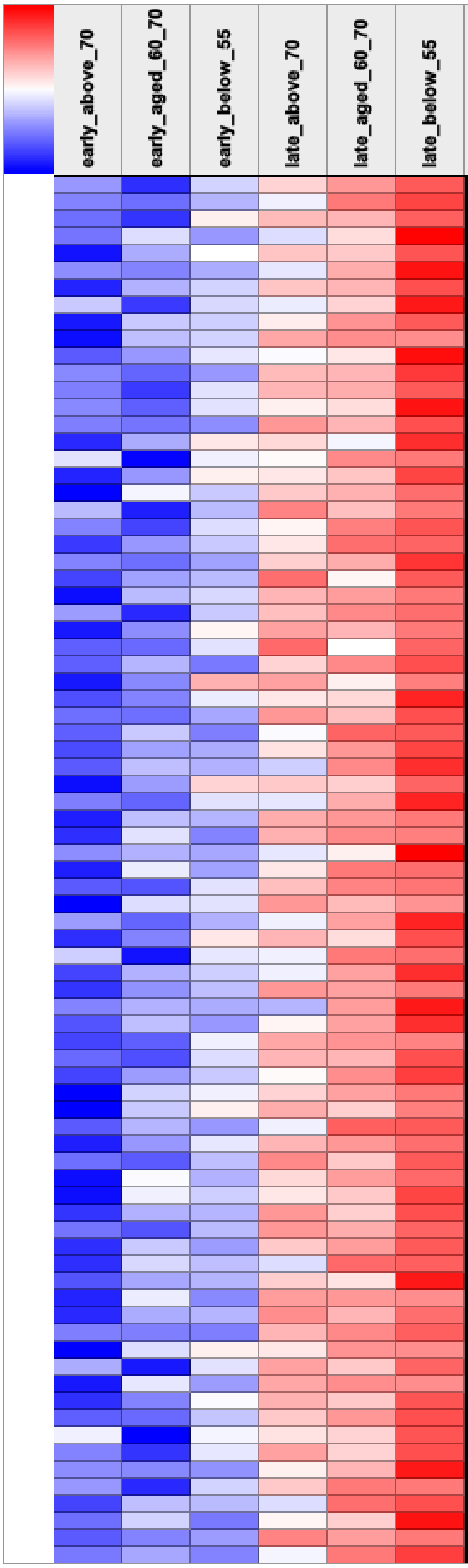

Probe ID	Descripti...	Correlation
AP1AR		0.92967
ASAP2		0.94892
FAM117B		0.92504
FSIP1		0.86496
GNAS		0.95293
LACTB		0.93284
LDHA		0.97982
LPIN1		0.83441
LPXN		0.95649
NREP		0.93131
NRN1		0.93709
NRP1		0.96081
PCNP		0.94736
SNX3		0.93248
SPAG1		0.95161
TEAD2		0.89672
TMEM163		0.75663
ZFAND5		0.95688
ZFP37		0.90067
ARL6IP1		0.83851
ARHGEF3		0.93996
DBN1		0.97517
RAD51B		0.97158
AMMECR1		0.91203
ANKRD29		0.95277
ARMC1		0.91004
CHORDC1		0.93968
CLCN4		0.88667
CRTAM		0.94792
DCLK1		0.83217
DNAH8		0.96746
ENAH		0.96535
FAP		0.90898
GPR137B		0.99027
LAPTM4B		0.92072
NGEF		0.91048
PARP1		0.94060
PDGFRL		0.95234
PMP22		0.90465
PRKAR2B		0.87438
PROCR		0.90974
SCLT1		0.96377
SEC11A		0.87739
SIX4		0.93078
SLCO4A1		0.94003
SMC3		0.79799
STX6		0.96549
TPM3		0.96869
ZFHX4		0.87625
ZNF541		0.95848
ARL6IP6		0.95191
THAP2		0.97060
CASP3		0.98430
DDIT4		0.91870
FKBP1A		0.89131
MEX3B		0.92594
SIAH1		0.97340
UBE2V2		0.94802
ADAMTSL1		0.90240
AATK		0.91239
ALDOC		0.96298
ANKRD46		0.95912
ARPC5		0.96148
CACNB1		0.90545
DNASE2		0.96348
DUSP7		0.87854
HSF2BP		0.95658
ISL2		0.94964
KIF7		0.87020
MEAF6		0.87046
MITD1		0.89643
MLLT11		0.96716
RAB33B		0.99235
SNAPC3		0.72352
SQLE		0.92184
SSTR1		0.94805
TXNRD1		0.89763
UAP1L1		0.93121
VWA7		0.90449
YWHAZ		0.94837
ZNF467		0.93247

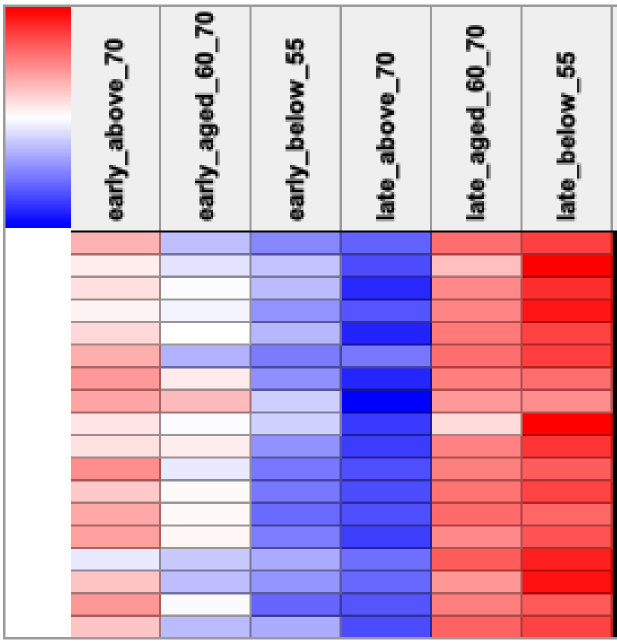

Probe ID	Description	Correlation
ETS1		0.97955
PPP2R5A		0.94461
ABCA6		0.98571
CLEC1A		0.98164
EPHA3		0.98627
GNB4		0.96173
IL33		0.96849
IQCA1		0.88783
MKX		0.92339
MRAS		0.98944
PLCL2		0.97492
PRKCQ		0.99176
PTPRE		0.97318
SLC1A2		0.98510
USP31		0.93907
APLP1		0.97334
BEST1		0.97401
PCNT		0.97943

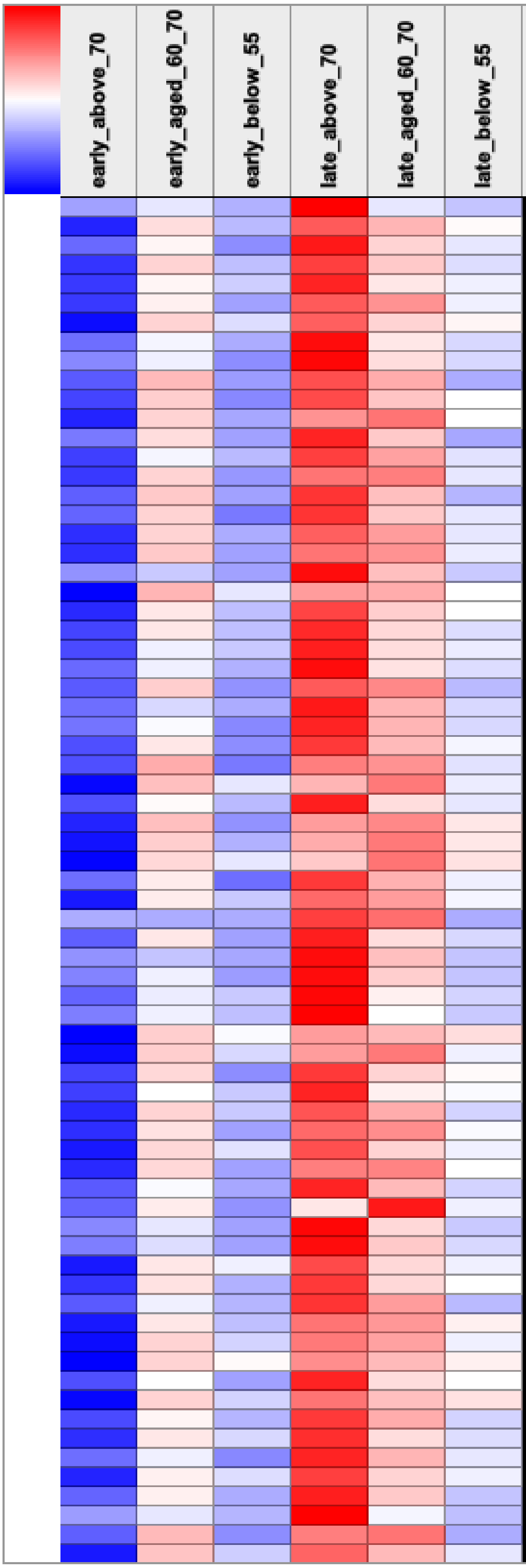

Probe ID	Descripti...	Correlation
ABLIM1		0.84726
C1orf159		0.98646
C7orf50		0.96979
CCNB1IP1		0.99211
CLTA		0.97315
DAZAP1		0.98560
HIGD2A		0.95572
HOXA2		0.95524
ITGB3BP		0.93002
LRGUK		0.95949
POLE4		0.98056
RBM6		0.92836
RPL18A		0.95067
SFSWAP		0.99220
SNRPA1		0.96307
TRMT1		0.97103
UBXN11		0.97226
CCDC84		0.98747
MST1		0.96973
NPM1		0.91205
PSPN		0.89209
SNRPD2		0.98820
EXOSC4		0.98709
PPIH		0.97275
PPP1R14B		0.95720
RPS27		0.96083
UQCRH		0.96224
CHTF18		0.96913
CTU1		0.98814
GYPC		0.92854
LUC7L		0.85212
MEST		0.98196
NPHP3		0.90484
NRXN2		0.88938
PBX4		0.81612
STX1A		0.96281
TELO2		0.97375
TRPM5		0.82452
CDK2AP2		0.97419
CCDC7		0.90382
HES4		0.94291
AP4M1		0.94214
BCL2L12		0.91287
C1orf35		0.86422
CCNL2		0.90571
CEMP1		0.97988
COPS6		0.96131
DDX56		0.98766
EPOR		0.97691
LSM7		0.96528
LTB4R		0.95827
MAMDC4		0.98815
MAPK8IP3		0.69598
MCM7		0.92935
MRPL11		0.94455
NDUFS6		0.95443
NXT1		0.98618
POLR1D		0.97592
RAC3		0.96326
RNASEH2C		0.95812
ROMO1		0.88425
SHARPIN		0.97736
SIVA1		0.94387
SLC39A4		0.99558
TCHH		0.97543
TEX22		0.96640
TOMM22		0.97462
TOMM7		0.97830
UNC5CL		0.86280
WFIKKN1		0.91152
ZNF205		0.97023

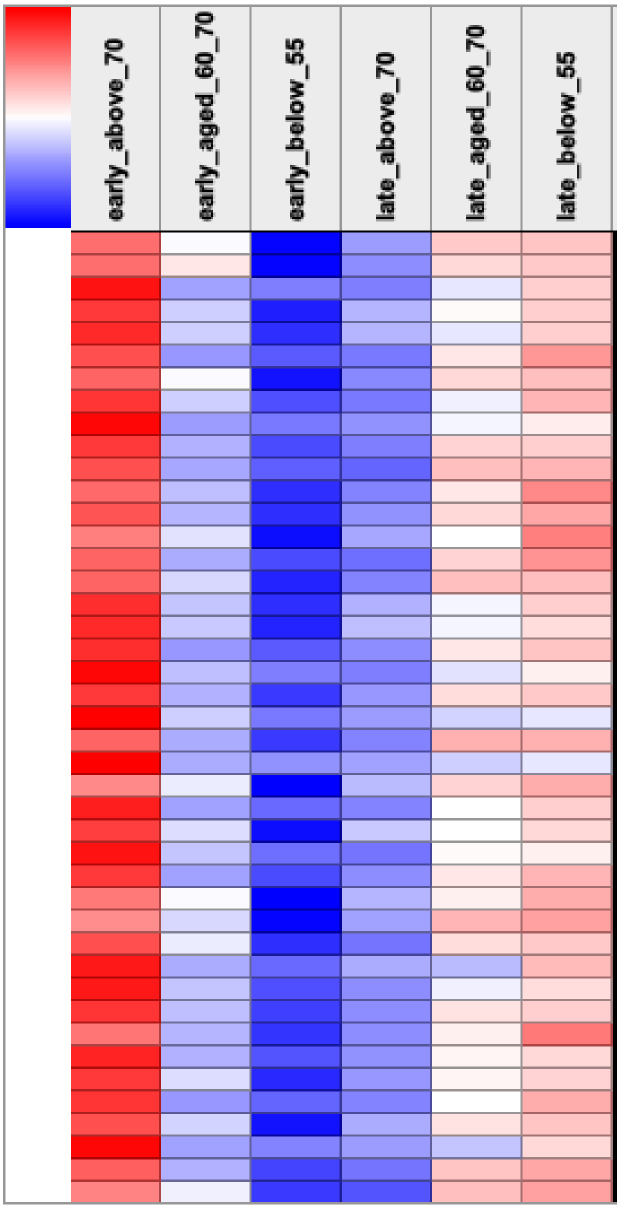

Probe ID	Description	Correlation
AOAH		0.94856
CSF3R		0.93173
ESYT1		0.95465
IQCG		0.98706
KCNC3		0.98007
LNX2		0.96944
MET		0.96465
NFATC1		0.98993
NOP14		0.94655
PIP4K2A		0.99026
PODXL		0.96446
RNF145		0.97353
RNF19B		0.98954
SLC39A11		0.93737
SWAP70		0.96711
FILIP1L		0.97603
MCL1		0.98871
AK5		0.97709
BMP2K		0.98287
CAMKK1		0.94240
CDK5RAP2		0.99486
DUSP10		0.90074
ENTPD1		0.96637
EPHA6		0.86880
KMO		0.92176
LCA5L		0.97699
NOX1		0.96202
PCDH17		0.96248
PLXNA3		0.99110
PRKG2		0.93519
RASGEF1B		0.92552
RUNDC3B		0.98334
ST8SIA1		0.93935
DLC1		0.98488
FLI1		0.99730
BCL9L		0.95452
CLPB		0.98841
CXCL16		0.99284
FRMD3		0.97613
LGALS2		0.98442
RDH10		0.92237
TBC1D2B		0.97302
TNXB		0.92673

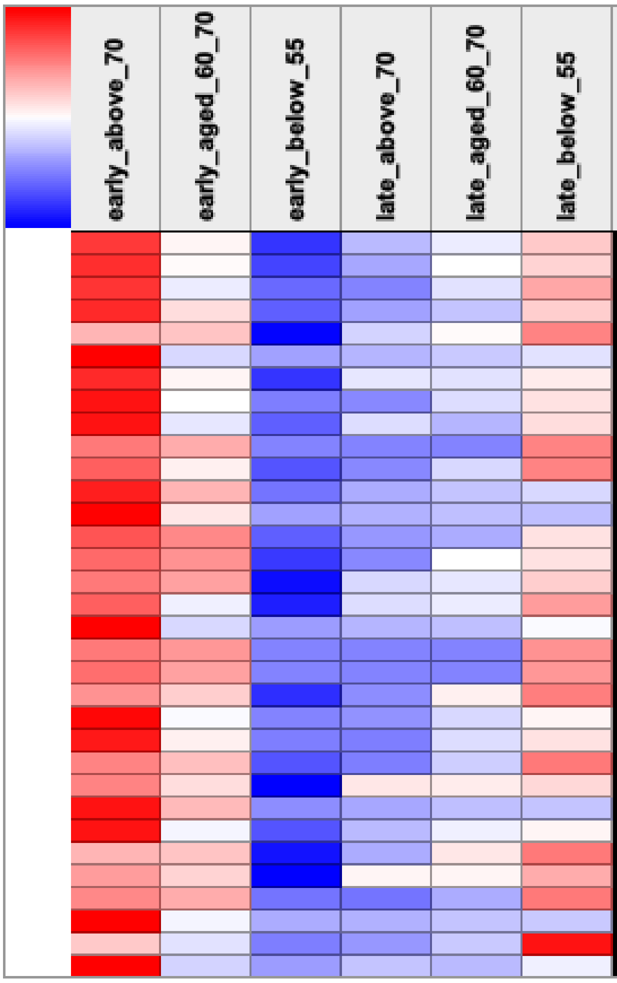

Probe ID	Description	Correlation
ADAM15		0.98442
AMOTL2		0.98009
BIRC3		0.96683
C3orf52		0.99492
CHST9		0.82761
GCDH		0.85945
NCOA7		0.95389
TJP3		0.96163
PMEL		0.94384
APOB		0.88217
ICAM1		0.95925
MMP7		0.92406
OLFML3		0.84743
PTGES		0.93633
RARRES1		0.93030
SESN2		0.91673
SIPA1L2		0.93369
TLL1		0.88537
CPEB1		0.87642
ESRRB		0.89593
APOBR		0.89904
C2CD2L		0.94878
CTSD		0.96419
CYP26B1		0.92217
EHD1		0.83117
HAS3		0.88161
LETM1		0.95679
MACC1		0.82843
MEIG1		0.82368
PRSS22		0.88347
PTGER4		0.83394
RSPH1		0.67202
SRGAP1		0.87123

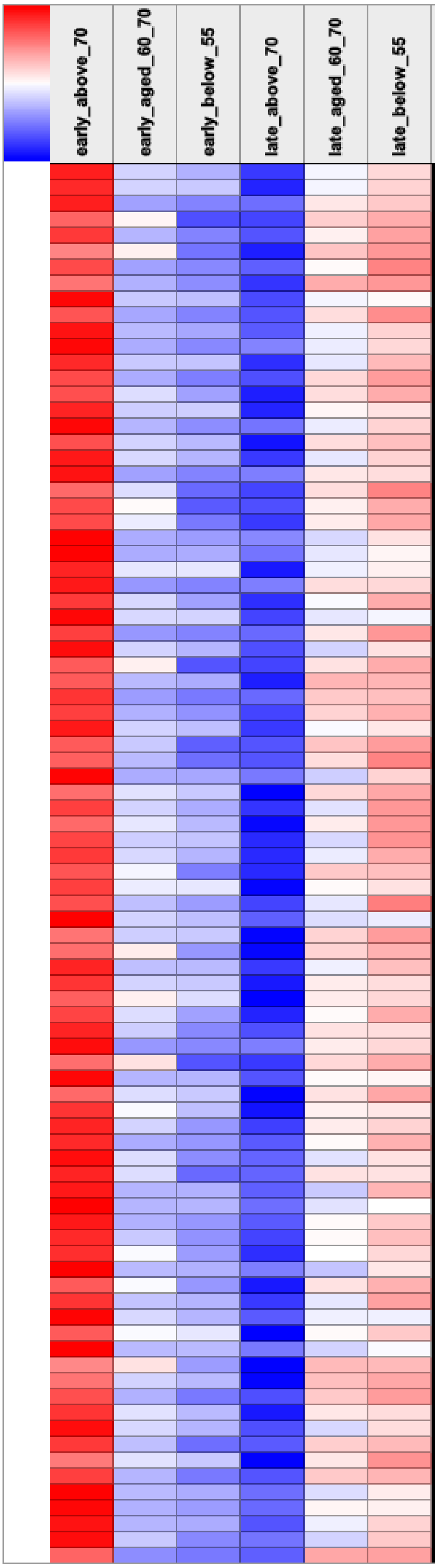

Probe ID	Descripti...	Correlation
AHNAK		0.99248
AMBRA1		0.98198
ANKH		0.97411
BTN3A3		0.92561
CCR6		0.98580
CIC		0.91320
CMIP		0.95589
CRIM1		0.93423
DENND3		0.96815
DOCK4		0.96101
DOCK8		0.98600
DSE		0.95555
EVI5		0.98529
FAR2		0.97207
GIPC2		0.98396
GLCE		0.97824
GLIS3		0.96852
MEF2D		0.97940
MFSD11		0.98799
NAMPT		0.95627
PCSK5		0.94591
PTPN14		0.95589
RAB11FIP5		0.98111
RNF125		0.93775
SHROOM3		0.94657
SRCAP		0.94450
TCF20		0.95218
TMEM127		0.99384
TMEM8B		0.94093
TNFAIP1		0.95987
TSGA10		0.96817
TSPAN15		0.93934
USP13		0.96579
VPS41		0.96102
ZNFX1		0.98734
ZZEF1		0.98034
EXT1		0.95041
HEG1		0.94915
STIM1		0.94668
C2CD2		0.94098
CCNB3		0.98158
CLEC16A		0.95275
CLTCL1		0.96445
CTSH		0.98737
DST		0.96738
HDLBP		0.94289
HSPA2		0.96319
KIF3B		0.93283
LEPR		0.93560
MAP2K5		0.93535
NBAS		0.98980
OSMR		0.97857
PLAGL1		0.91523
PLXDC2		0.99151
POLR1A		0.98952
PYGL		0.95556
SCN1B		0.91512
SPTLC2		0.97040
WARS2		0.95181
ZNF214		0.96527
MINK1		0.99622
ATXN2		0.98916
KLHL21		0.96936
COL14A1		0.97000
AGPAT3		0.96494
ALAS1		0.94140
ANKRD52		0.98920
APBB2		0.99870
ATG2A		0.98414
CRAT		0.92895
EBF1		0.97015
IPO13		0.98595
KIAA0100		0.94736
KIAA1522		0.92654
KLHL26		0.92051
LZTR1		0.89504
MAN2A2		0.94020
MAP3K3		0.96447
NAV2		0.98311
PITRM1		0.97352
PTPN23		0.97641
RIMKLB		0.93081
RNF213		0.97710
SLC38A9		0.95634
SLFN5		0.96734
TAF3		0.98487
XRRA1		0.96546
ZHX3		0.91432

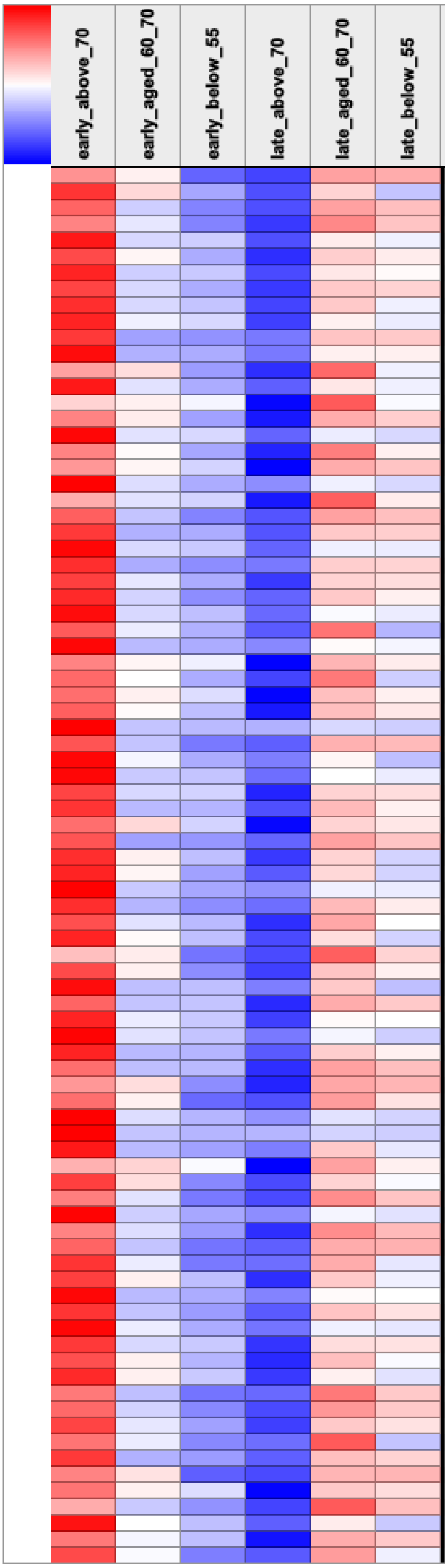

Probe ID	Descripti...	Correlation
ABCB1		0.88777
ADAM22		0.94451
ARHGAP31		0.95995
BHLHE41		0.93309
CADPS2		0.96777
DAB2		0.98729
DDHD1		0.97606
DEPDC5		0.99047
DGKD		0.98868
DNAJC5		0.95886
DSTYK		0.94329
EGFR		0.94284
GLI2		0.87148
IQSEC1		0.97419
PAN2		0.74138
RGL1		0.94024
RPAP1		0.91378
RUNX1T1		0.93585
RUNX2		0.89730
SLC45A4		0.90033
TMCC3		0.85620
WDR19		0.95769
XPR1		0.97297
ZCCHC14		0.93216
ZFYVE26		0.95250
SLC3A2		0.99292
GBA2		0.98808
INSR		0.94551
PATL2		0.91273
PRKCE		0.92677
SNED1		0.90744
STARD9		0.93399
ZFYVE28		0.93806
FAF1		0.97490
AASS		0.81734
ABCC9		0.95389
ALX3		0.91507
ARAP2		0.93844
ARHGEF17		0.97629
CALCOCO1		0.98720
COL6A2		0.92920
CREBBP		0.94191
CUBN		0.96883
H6PD		0.96403
HACL1		0.89720
ITGA8		0.97132
KLHL3		0.98874
NKD1		0.96495
NOTCH1		0.80952
PCDH10		0.98496
PEX5L		0.92705
RAB15		0.96442
RASGRF2		0.96693
SLC46A1		0.91128
SPEN		0.98084
SPTB		0.95157
SYNE1		0.89679
TACR1		0.94621
TM7SF3		0.87236
WBP11		0.80764
BAHCC1		0.95305
FAM43A		0.81488
PPP1R3E		0.96900
SHANK3		0.93496
PAPPA		0.91139
TCF4		0.93564
ABCA2		0.93629
ABCA3		0.96759
ABR		0.98060
ARHGAP35		0.93287
BTN3A1		0.98853
DCUN1D3		0.93217
FAT4		0.98166
NCOR2		0.98308
NEURL4		0.95827
NLRC5		0.90651
PIK3C2B		0.95891
PLEC		0.99659
PPM1J		0.87868
RELL2		0.97167
SBNO2		0.90208
SCN4B		0.93022
SCN8A		0.85005
SERPINB8		0.94275
SZT2		0.95204
WNT7B		0.97525

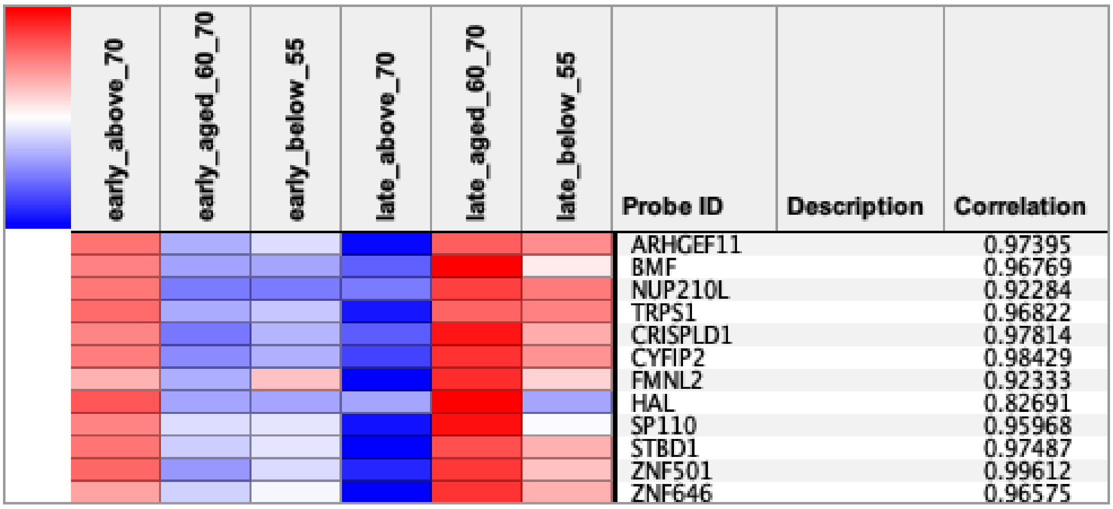
early_above_70
early_aged_60_70
early_below_55
late_above_70
late_aged_60_70
late_below_55
Probe ID
Description
Correlation
ARHGEF11 0.97395
BMF 0.96769
NUP210L 0.92284
TRPS1 0.96822
CRISPLD1 0.97814
CYFIP2 0.98429
FMNL2 0.92333
HAL 0.82691
SP110 0.95968
STBD1 0.97487
ZNF501 0.99612
ZNF646 0.96575

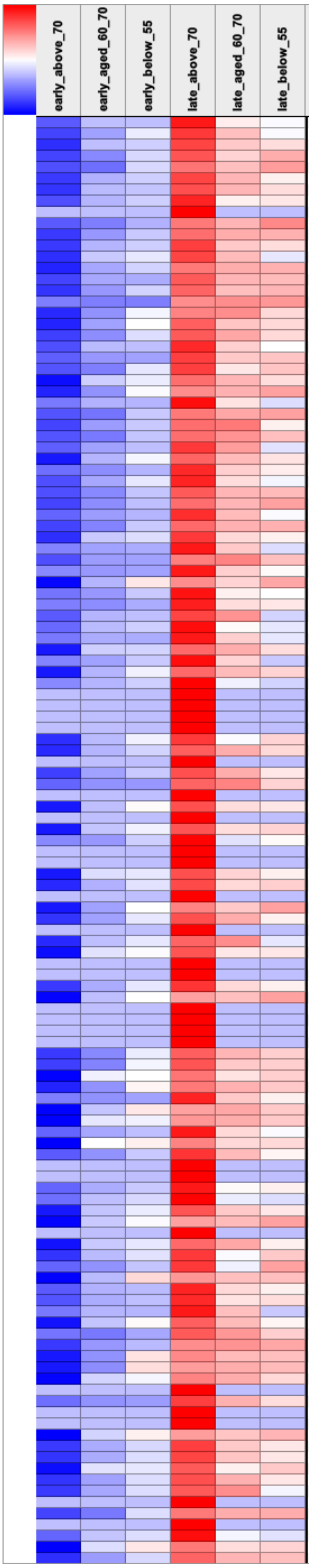

Probe ID	Descripti...	Correlation
APTX		0.98694
BRIX1		0.98821
C12orf65		0.98684
CASP2		0.95981
CBX1		0.91274
CBX3		0.98881
CCT6A		0.98080
CDKN2A		0.99347
CDX1		0.80487
CHPT1		0.88489
COPS5		0.94013
DNAJC2		0.99266
EIF3E		0.96813
ENY2		0.92371
GEMIN2		0.95811
GMNN		0.95691
GPR84		0.80772
HNRNPA2...		0.90753
HNRNPC		0.96117
HNRNPH3		0.96391
HOXA1		0.99461
HSPD1		0.96799
LBR		0.97300
LYAR		0.93398
MNAT1		0.88396
NDC80		0.96399
NIPSNAP3A		0.90035
NR2C1		0.92541
NUP107		0.92315
PFDN4		0.96314
PHGDH		0.95630
POLE3		0.98522
PRKRIP1		0.99271
PSMA7		0.95689
RTN4R		0.93269
SKP2		0.98434
SLC44A1		0.93902
SNRPF		0.98704
SOX4		0.95667
TATDN1		0.89361
TTK		0.96138
UPF3B		0.86686
CDK2		0.97914
PTGES3		0.97203
ISG15		0.94524
MFF		0.97048
NAP1L1		0.97025
PTMA		0.94588
TUBB		0.94879
EXOSC3		0.95105
TMED6		0.92602
ADAM20		0.80487
ATP1A4		0.80487
BIRC7		0.80487
C11orf53		0.80487
C9orf78		0.97235
CCDC59		0.96311
CDH8		0.80487
CLNS1A		0.98127
CNKSR3		0.91294
DPEP1		0.80487
DYNLT1		0.96444
FCRLA		0.80487
FHAD1		0.96457
GOLGA7		0.94261
GSDMC		0.80487
LGI1		0.80487
LUC7L3		0.96032
MED10		0.98187
PHACTR3		0.80487
PIR		0.89718
PSMB7		0.97522
PYY		0.80487
RBMX		0.93432
SEC61G		0.94051
SEL1L2		0.80487
SLC3A1		0.80487
SNRPC		0.99519
SYCP3		0.84661
SYT16		0.80487
TMC2		0.80487
XPNPEP2		0.80487
ZIC5		0.80487
GATC		0.96061
ZNF77		0.96819
ARL2BP		0.89214
CDK2AP1		0.92306
HYLS1		0.96899
NDUFA4		0.83535
RAB26		0.85099
TAF4		0.98982
TRA2A		0.85320
TUBB3		0.99020
XIRP1		0.80487
GRM8		0.80487
ARMC10		0.98697
BAIAP2L2		0.94765
C12orf73		0.96984
C16orf91		0.84127
CALML6		0.80487
CHTOP		0.94091
COX6A1		0.97470
ELOVL6		0.93818
ERH		0.84879
FN3KRP		0.99575
FNTA		0.99523
GRIN1		0.94976
HINFP		0.94650
INTS8		0.92323
MED30		0.88264
NAA20		0.88826
NDUFA12		0.85232
NKIRAS2		0.89949
NPHS1		0.80487
ORC6		0.96017
PCDHA1		0.80487
PCDHA7		0.80487
PFDN2		0.84412
PSMA2		0.99354
PSMC3IP		0.99254
SARNP		0.93498
TAF11		0.97987
TAF1D		0.95303
TAT		0.80487
TCEA1		0.91945
TNNT1		0.80487
TXN		0.96129
UBL5		0.88635
ZNF823		0.95552

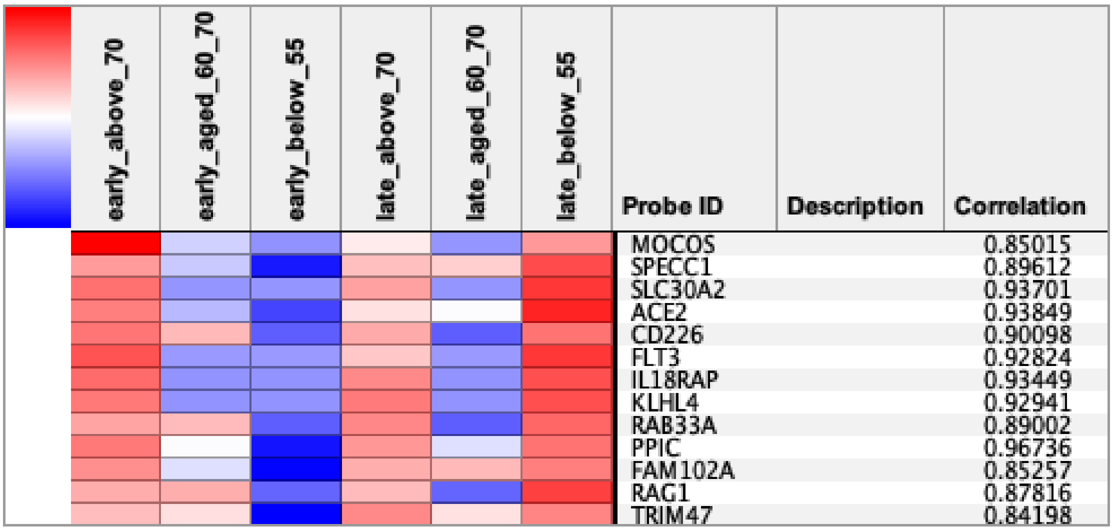

early_above_70
early_aged_60_70
early_below_55
late_above_70
late_aged_60_70
late_below_55
Probe ID
Description
Correlation
MOCOS 0.85015
SPECC1 0.89612
SLC30A2 0.93701
ACE2 0.93849
CD226 0.90098
FLT3 0.92824
IL18RAP 0.93449
KLHL4 0.92941
RAB33A 0.89002
PPIC 0.96736
FAM102A 0.85257
RAG1 0.87816
TRIM47 0.84198

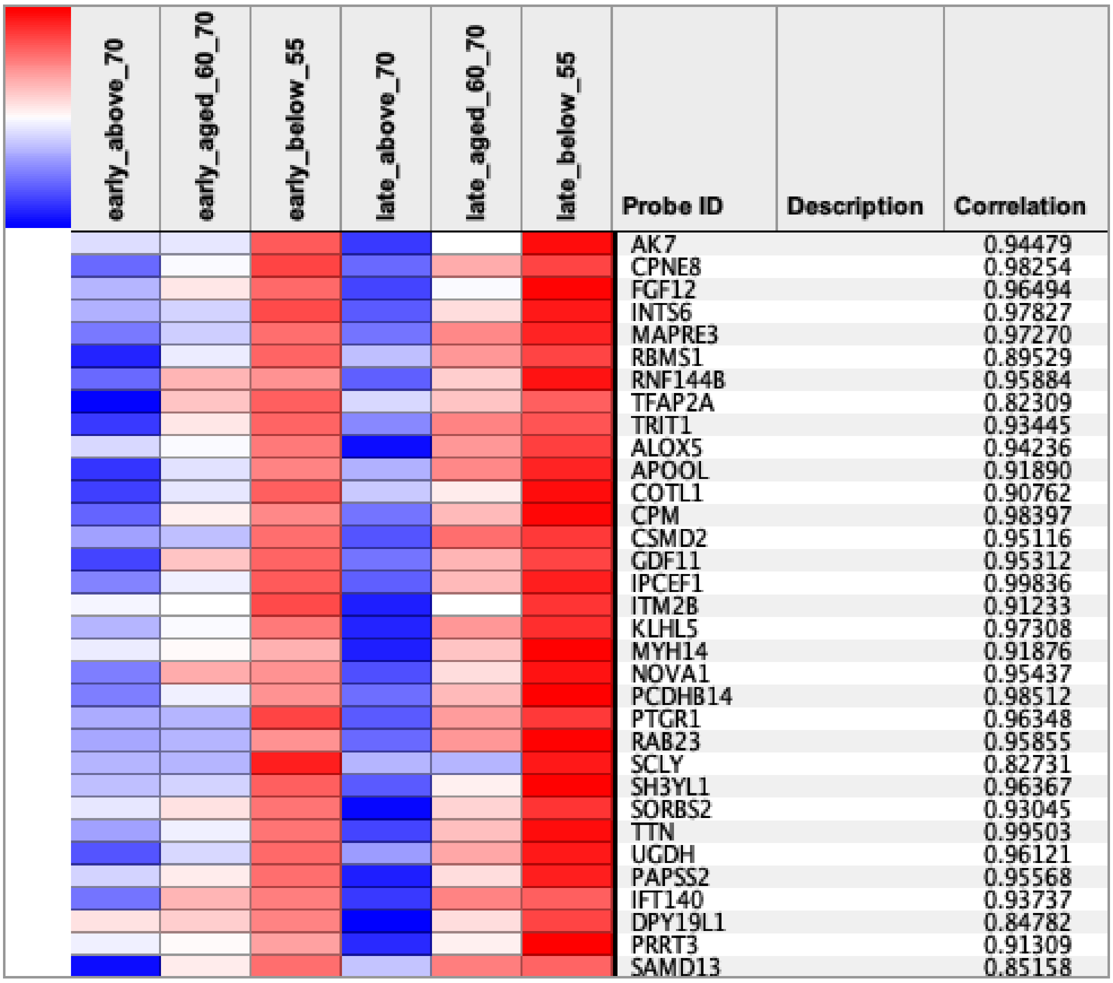

early_above_70
early_aged_60_70
early_below_55
late_above_70
late_aged_60_70
late_below_55
Probe ID
Description
Correlation
AK7 0.94479
CPNE8 0.98254
FGF12 0.96494
INTS6 0.97827
MAPRE3 0.97270
RBMS1 0.89529
RNF144B 0.95884
TFAP2A 0.82309
TRIT1 0.93445
ALOX5 0.94236
APOOL 0.91890
COTL1 0.90762
CPM 0.98397
CSMD2 0.95116
GDF11 0.95312
IPCEF1 0.99836
ITM2B 0.91233
KLHL5 0.97308
MYH14 0.91876
NOVA1 0.95437
PCDHB14 0.98512
PTGR1 0.96348
RAB23 0.95855
SCLY 0.82731
SH3YL1 0.96367
SORBS2 0.93045
TTN 0.99503
UGDH 0.96121
PAPSS2 0.95568
IFT140 0.93737
DPY19L1 0.84782
PRRT3 0.91309
SAMD13 0.85158

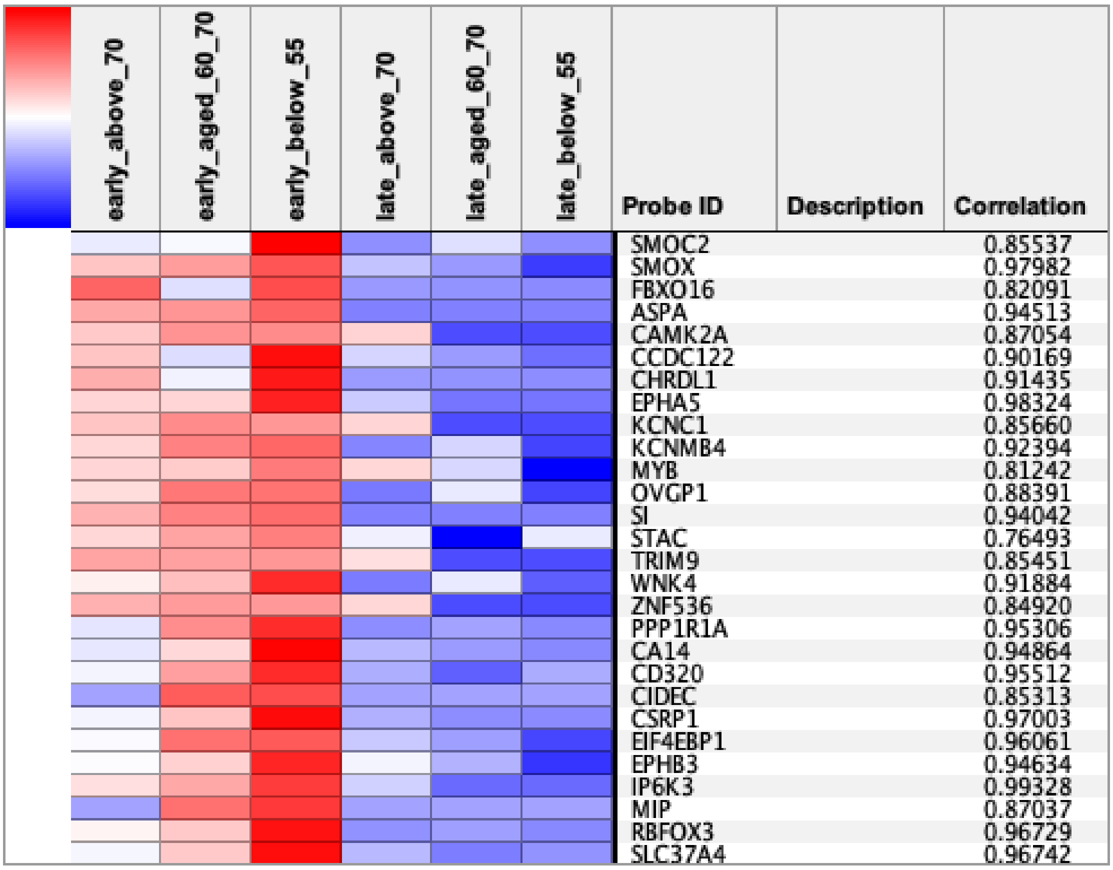

early_above_70
early_aged_60_70
early_below_55
late_above_70
late_aged_60_70
late_below_55
Probe ID
Description
Correlation
SMOC2 0.85537
SMOX 0.97982
FBXO16 0.82091
ASPA 0.94513
CAMK2A 0.87054
CCDC122 0.90169
CHRDL1 0.91435
EPHA5 0.98324
KCNC1 0.85660
KCNMB4 0.92394
MYB 0.81242
OVGP1 0.88391
SI 0.94042
STAC 0.76493
TRIM9 0.85451
WNK4 0.91884
ZNF536 0.84920
PPP1R1A 0.95306
CA14 0.94864
CD320 0.95512
CIDEC 0.85313
CSRP1 0.97003
EIF4EBP1 0.96061
EPHB3 0.94634
IP6K3 0.99328
MIP 0.87037
RBFOX3 0.96729
SLC37A4 0.96742

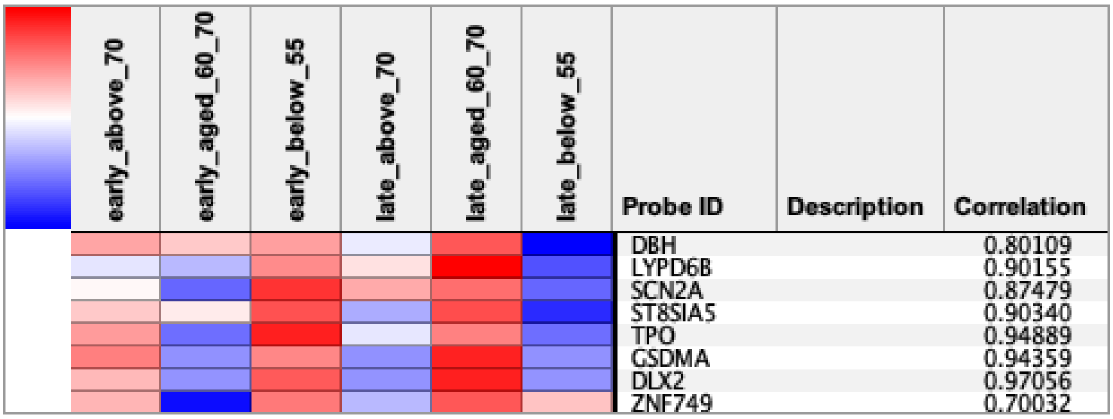

early_above_70
early_aged_60_70
early_below_55
late_above_70
late_aged_60_70
late_below_55
Probe ID
Description
Correlation
DBH 0.80109
LYPD6B 0.90155
SCN2A 0.87479
ST8SIA5 0.90340
TPO 0.94889
GSDMA 0.94359
DLX2 0.97056
ZNF749 0.70032

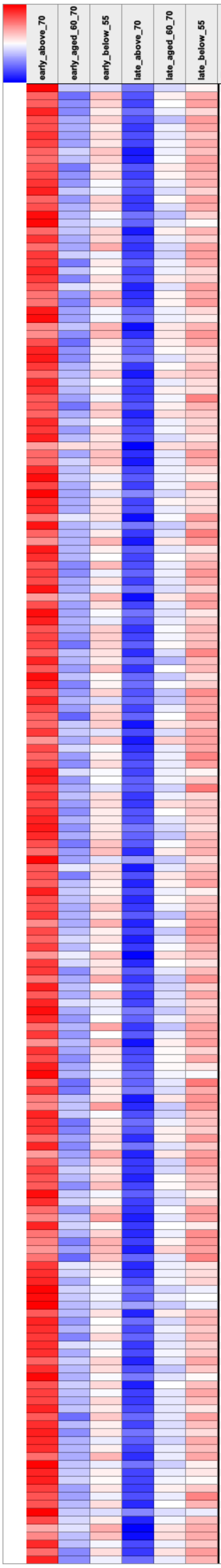

Probe ID	Descripti...	Correlation
ABHD2		0.91184
ACADM		0.95352
ADAM10		0.98404
AFF4		0.97185
APLP2		0.97707
ARFGEF2		0.99544
BCL2L13		0.99484
BIRC6		0.99752
CALD1		0.96872
CCDC91		0.97477
CLASP1		0.97024
CLCN3		0.99153
CLOCK		0.98780
CNOT1		0.97492
COPA		0.97874
CPD		0.99368
CPEB3		0.94919
DBT		0.93796
DCAF8		0.97225
EIF2AK4		0.99457
FAM177A1		0.95811
FBXL5		0.97677
FBXO36		0.97707
FBXO42		0.98416
FCHO2		0.97857
GDE1		0.97199
GOSR1		0.96125
GPD2		0.95604
HMBOX1		0.97332
IPO11		0.95161
ITGA6		0.93124
KCTD18		0.93183
LARP1B		0.96352
LATS1		0.97515
LPP		0.95415
MAN2A1		0.99628
MCM9		0.95916
MERTK		0.97865
MFN2		0.98375
MIA3		0.96762
MIB1		0.96928
MPP7		0.97128
MSH3		0.98301
MTMR6		0.99455
MYCBP2		0.97991
MYOF		0.81998
NAA35		0.97389
PCSK6		0.96342
PFKFB4		0.98846
PHLPP2		0.95093
PIAS1		0.99868
PRKAR2A		0.92620
PRKCH		0.98528
RBL2		0.99031
RBM26		0.93361
RDH12		0.94347
RETSAT		0.96762
REV3L		0.92388
RREB1		0.97717
SCAPER		0.99143
SEC23IP		0.97619
SEC24A		0.97398
SERINC1		0.99698
SETD7		0.96744
SLC26A2		0.90688
SLC2A1		0.99371
SLC35A3		0.95578
SPPL2A		0.97709
STAM		0.99276
STXBP1		0.99546
TCF12		0.97502
TEX9		0.97024
TMBIM6		0.96533
TMEM33		0.98220
TMX4		0.95543
UBA5		0.96934
UBE2Q2		0.96633
UBE4A		0.95734
USP6NL		0.99520
ZSCAN20		0.94564
EXOC8		0.96349
GNG12		0.97072
PBX1		0.91506
STAT3		0.97784
ACBD5		0.97204
AFF1		0.98559
ANKRD11		0.95194
CDS1		0.98357
IQUB		0.94683
KDM4A		0.99072
PDXDC1		0.98318
ACO2		0.99073
ADAT1		0.98967
ALG10		0.96271
ARCN1		0.97914
ATE1		0.97292
ATF6		0.96899
ATP11C		0.90632
CPPED1		0.96625
DENND4C		0.97348
DIP2C		0.98015
DNAJC6		0.96987
EPS15		0.99114
EXT2		0.99112
FBXL17		0.97849
FBXL3		0.93835
FIG4		0.97600
FOXO1		0.96948
GBF1		0.99379
HDAC6		0.88444
HIPK2		0.97385
HSPA13		0.98963
IDE		0.93982
IL6ST		0.97663
IMPACT		0.98412
IPO8		0.96985
LAMP2		0.94775
LNPEP		0.98474
MAGT1		0.94527
MAPK1		0.98848
MFSD6		0.92256
MFSD9		0.99374
MID2		0.98702
NFAT5		0.97513
NIPAL2		0.93767
OCRL		0.93578
P4HA1		0.96417
PHKA1		0.95048
PHKB		0.91593
PIGK		0.97900
POLK		0.96844
PREPL		0.98181
PRKD3		0.97178
RPGRIP1L		0.97163
SECISBP2L		0.90986
STX12		0.97648
TFPI2		0.98844
TGFBRAP1		0.98968
TIRAP		0.98156
TMCO3		0.95021
TMED5		0.98110
TOR1AIP1		0.97198
UGGT2		0.93159
URB1		0.96852
XIAP		0.96624
ZC3H13		0.93880
ZC3H14		0.91392
SLC25A46		0.92726
LUZP1		0.96788
TUBGCP5		0.98167
ZBTB38		0.98624
PDE4B		0.90929
AHCYL2		0.94250
ALDH6A1		0.84565
BSCL2		0.98538
CCDC47		0.97533
CHD9		0.99409
CRADD		0.98326
DOLK		0.97018
EIF2AK3		0.99449
ELMOD2		0.97669
GLB1		0.98333
GOLGB1		0.93690
GPHN		0.98143
GRN		0.98850
HARBI1		0.99819
MGAT2		0.97757
MOB1B		0.95420
MPZL1		0.99412
NHLRC2		0.96688
PIGS		0.97542
PIP4K2C		0.99712
PJA2		0.97152
PLBD2		0.92922
PPARA		0.97580
PTPN13		0.95920
RALGAPB		0.99415
SLC31A1		0.96614
SLC33A1		0.99200
SLC41A1		0.87609
SPATA2		0.98051
STXBP5		0.86677
TM9SF4		0.93630
TMEM170A		0.98638
TRIM33		0.96428
UBR1		0.97170

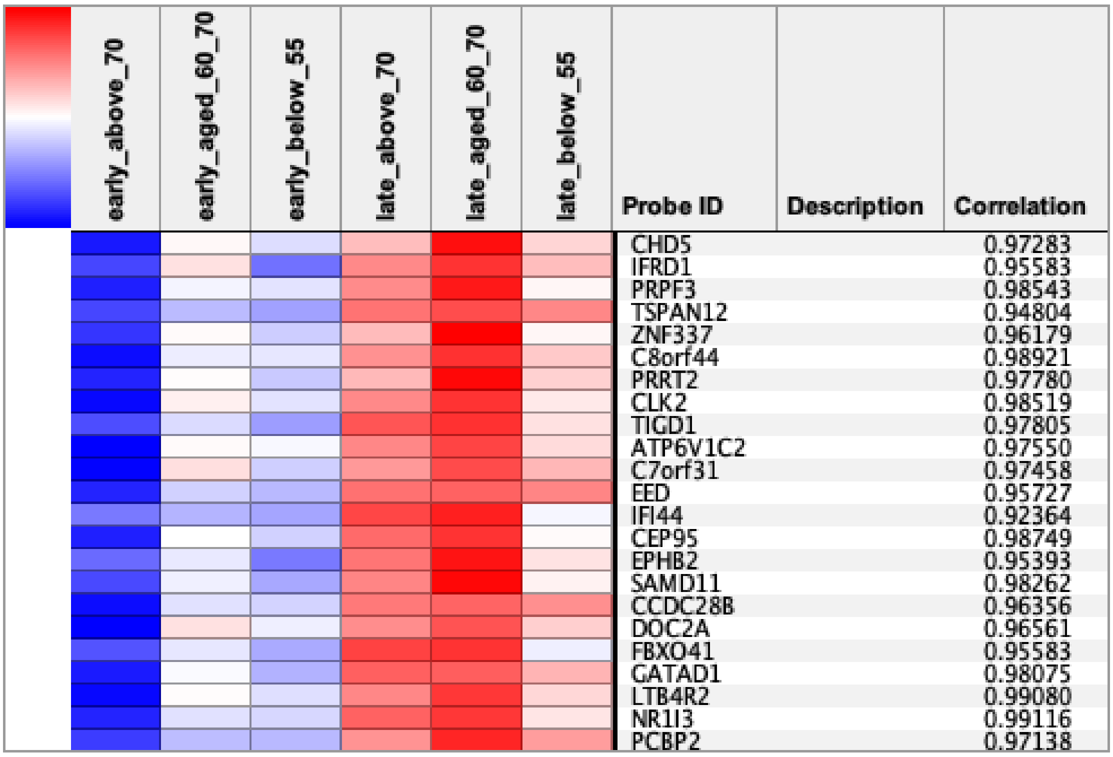
early_above_70
early_aged_60_70
early_below_55
late_above_70
late_aged_60_70
late_below_55
Probe ID
Description
Correlation
CHD5 0.97283
IFRD1 0.95583
PRPF3 0.98543
TSPAN12 0.94804
ZNF337 0.96179
C8orf44 0.98921
PRRT2 0.97780
CLK2 0.98519
TIGD1 0.97805
ATP6V1C2 0.97550
C7orf31 0.97458
EED 0.95727
IFI44 0.92364
CEP95 0.98749
EPHB2 0.95393
SAMD11 0.98262
CCDC28B 0.96356
DOC2A 0.96561
FBXO41 0.95583
GATAD1 0.98075
LTB4R2 0.99080
NR1I3 0.99116
PCBP2 0.97138

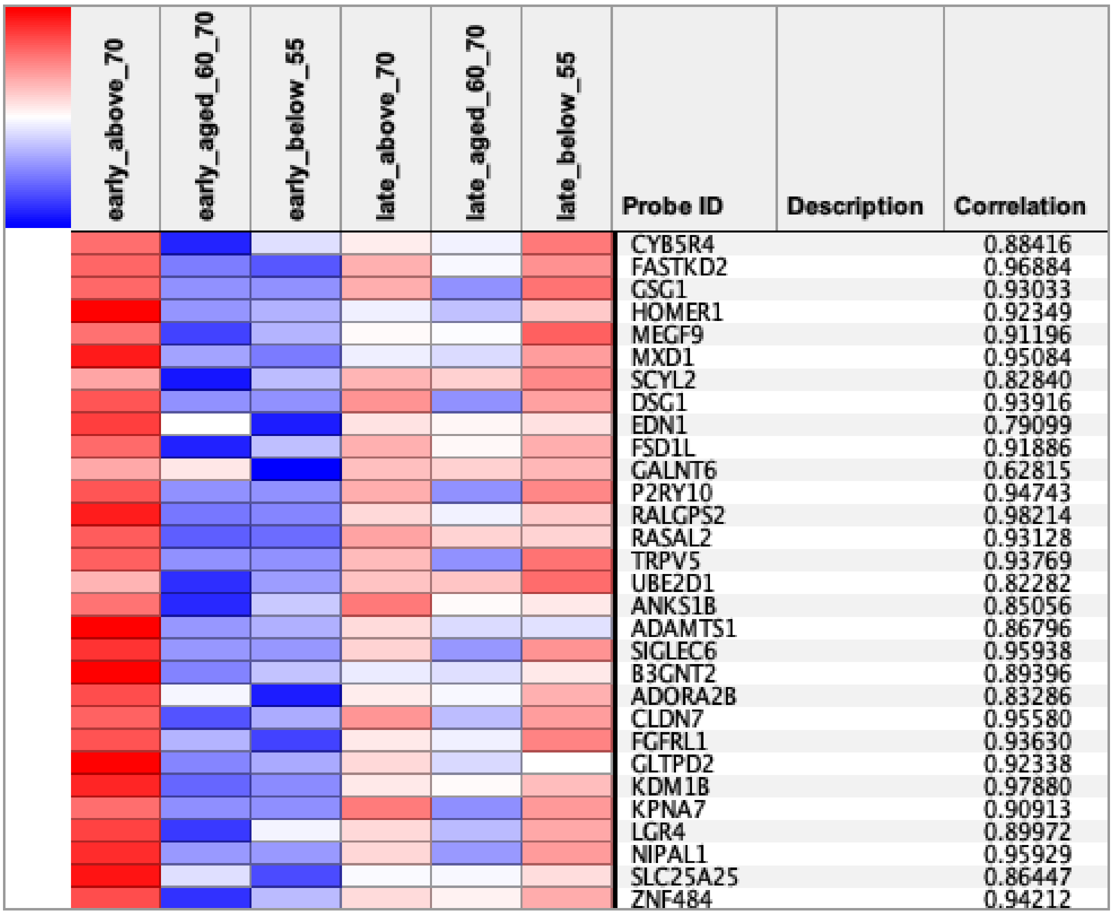
early_above_70
early_aged_60_70
early_below_55
late_above_70
late_aged_60_70
late_below_55
Probe ID
Description
Correlation
CYB5R4 0.88416
FASTKD2 0.96884
GSG1 0.93033
HOMER1 0.92349
MEGF9 0.91196
MXD1 0.95084
SCYL2 0.82840
DSG1 0.93916
EDN1 0.79099
FSD1L 0.91886
GALNT6 0.62815
P2RY10 0.94743
RALGPS2 0.98214
RASAL2 0.93128
TRPV5 0.93769
UBE2D1 0.82282
ANKS1B 0.85056
ADAMTS1 0.86796
SIGLEC6 0.95938
B3GNT2 0.89396
ADORA2B 0.83286
CLDN7 0.95580
FGFRL1 0.93630
GLTPD2 0.92338
KDM1B 0.97880
KPNA7 0.90913
LGR4 0.89972
NIPAL1 0.95929
SLC25A25 0.86447
ZNF484 0.94212

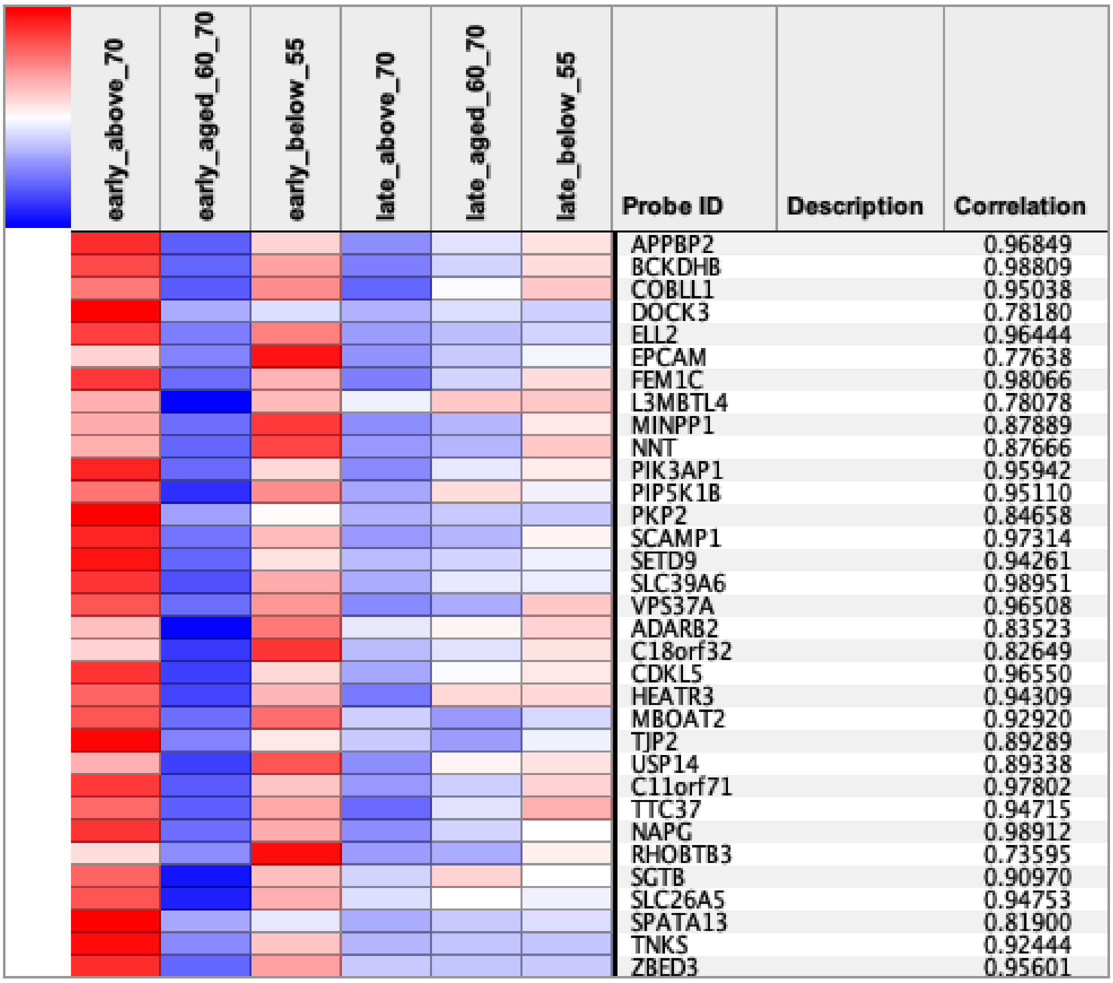
early_above_70
early_aged_60_70
early_below_55
late_above_70
late_aged_60_70
late_below_55
Probe ID
Description
Correlation
APPBP2 0.96849
BCKDHB 0.98809
COBLL1 0.95038
DOCK3 0.78180
ELL2 0.96444
EPCAM 0.77638
FEM1C 0.98066
L3MBTL4 0.78078
MINPP1 0.87889
NNT 0.87666
PIK3AP1 0.95942
PIP5K1B 0.95110
PKP2 0.84658
SCAMP1 0.97314
SETD9 0.94261
SLC39A6 0.98951
VPS37A 0.96508
ADARB2 0.83523
C18orf32 0.82649
CDKL5 0.96550
HEATR3 0.94309
MBOAT2 0.92920
TJP2 0.89289
USP14 0.89338
C11orf71 0.97802
TTC37 0.94715
NAPG 0.98912
RHOBTB3 0.73595
SGTB 0.90970
SLC26A5 0.94753
SPATA13 0.81900
TNKS 0.92444
ZBED3 0.95601

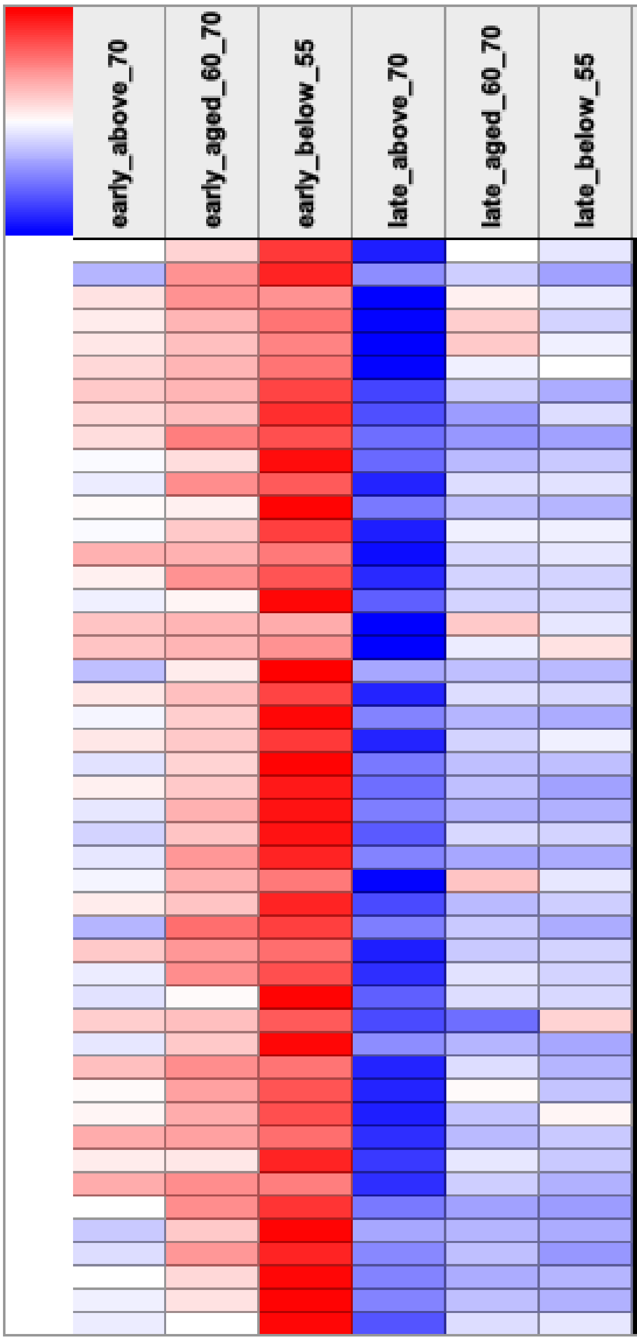

Probe ID	Descripti...	Correlation
ABCC8		0.97296
ACTA2		0.90876
ADAMTS17		0.89534
ARHGEF19		0.91244
CACNA1C		0.87995
DHRS12		0.93216
FGFR3		0.97890
HHAT		0.97556
KCTD1		0.93499
LDB3		0.97057
MEIS2		0.97252
MYLK		0.94698
MYOM1		0.97821
POLN		0.92581
RAPGEF3		0.98420
RBM24		0.95722
SGK1		0.81690
SPRY2		0.86636
TPM1		0.87456
ZNF516		0.99088
PIPOX		0.94742
HERPUD1		0.98393
AKAP7		0.95766
ALDH1A2		0.96779
COL23A1		0.95676
CRISPLD2		0.97533
FERMT1		0.94765
HYDIN		0.88654
KANK4		0.99353
KCNIP3		0.90596
NTRK3		0.95770
OSBPL7		0.98078
PRDM8		0.94907
PTN		0.87426
PYGM		0.94027
TGFB3		0.94474
ADCY5		0.97200
ANXA11		0.96953
APOF		0.93834
CPAMD8		0.98113
FAM3D		0.92191
HIF3A		0.94724
JPH4		0.89565
RASL12		0.93730
SLC2A4		0.94852
SYNM		0.94110
VPS36		0.94994

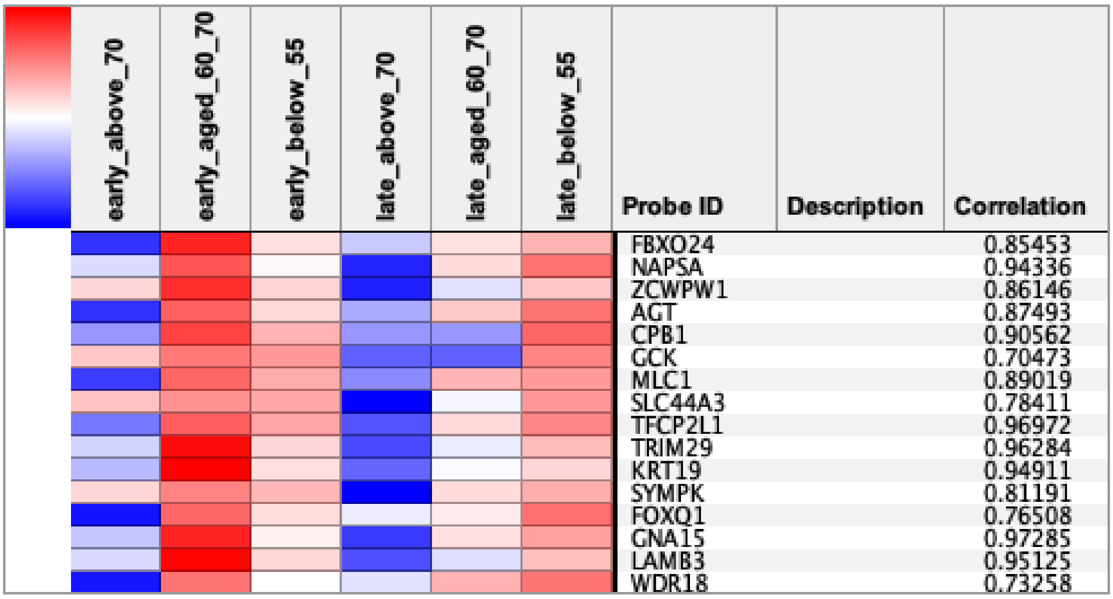
early_above_70
early_aged_60_70
early_below_55
late_above_70
late_aged_60_70
late_below_55
Probe ID
Description
Correlation
FBXO24 0.85453
NAPSA 0.94336
ZCWPW1 0.86146
AGT 0.87493
CPB1 0.90562
GCK 0.70473
MLC1 0.89019
SLC44A3 0.78411
TFCP2L1 0.96972
TRIM29 0.96284
KRT19 0.94911
SYMPK 0.81191
FOXQ1 0.76508
GNA15 0.97285
LAMB3 0.95125
WDR18 0.73258

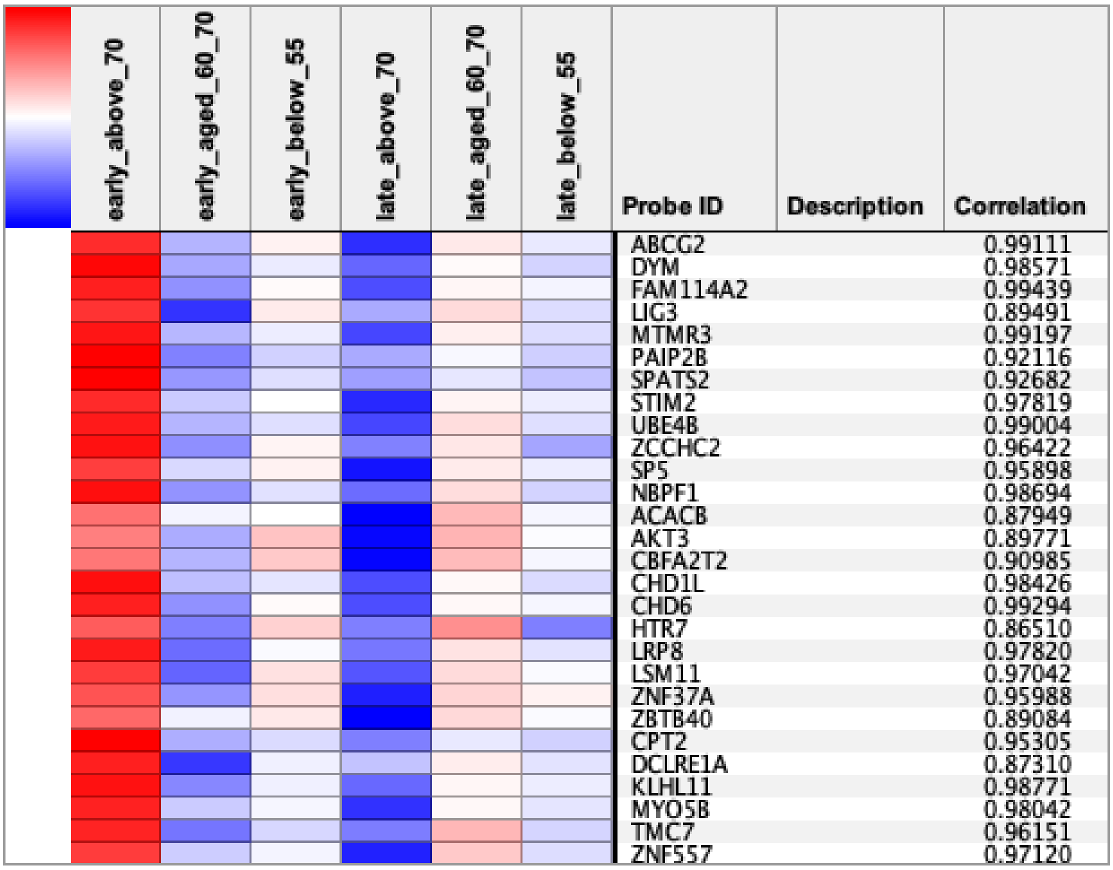
early_above_70
early_aged_60_70
early_below_55
late_above_70
late_aged_60_70
late_below_55
Probe ID
Description
Correlation
ABCG2 0.99111
DYM 0.98571
FAM114A2 0.99439
LIG3 0.89491
MTMR3 0.99197
PAIP2B 0.92116
SPATS2 0.92682
STIM2 0.97819
UBE4B 0.99004
ZCCHC2 0.96422
SP5 0.95898
NBPF1 0.98694
ACACB 0.87949
AKT3 0.89771
CBFA2T2 0.90985
CHD1L 0.98426
CHD6 0.99294
HTR7 0.86510
LRP8 0.97820
LSM11 0.97042
ZNF37A 0.95988
ZBTB40 0.89084
CPT2 0.95305
DCLRE1A 0.87310
KLHL11 0.98771
MYO5B 0.98042
TMC7 0.96151
ZNF557 0.97120

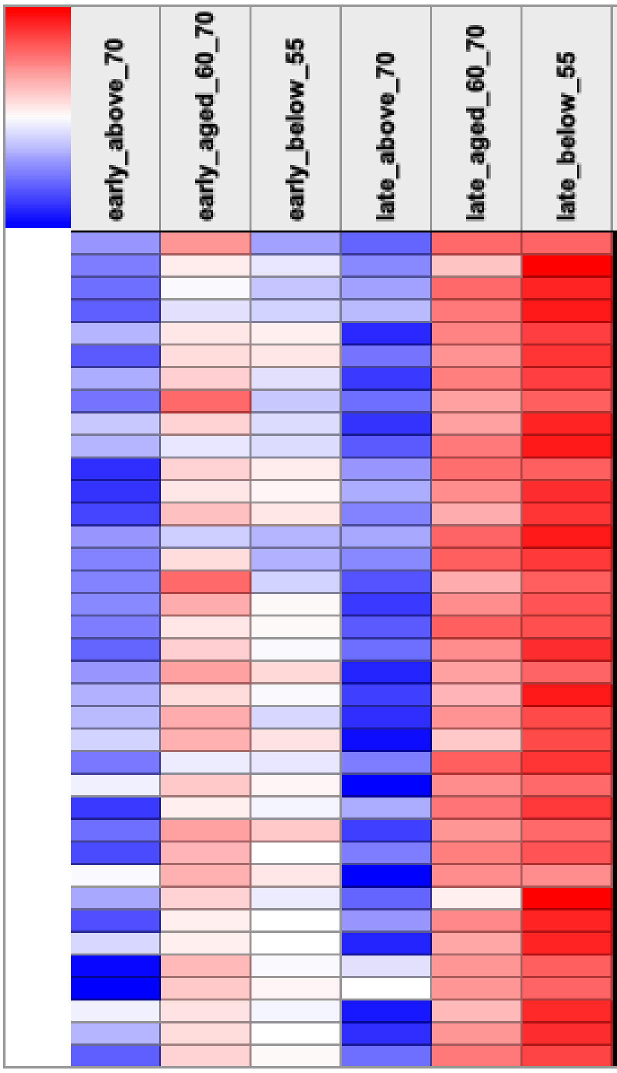

Probe ID	Description	Correlation
ARRDC2		0.93095
CACNA2D4		0.95234
CBLN3		0.94768
DLG4		0.91326
FAS		0.96898
NTN4		0.98239
SAMD4A		0.98236
TBX6		0.92348
TMEM138		0.95843
ABLIM2		0.95125
C1QTNF3		0.93712
EFEMP1		0.93600
EPHX1		0.96057
FBLN7		0.89584
FRAS1		0.95888
GHRL		0.92963
GJA1		0.98003
HDAC9		0.98394
KCNC4		0.99505
MATN2		0.94615
OBSCN		0.97156
PAMR1		0.95851
PELI2		0.90048
SAMD15		0.96269
SLC5A1		0.87801
SMC1B		0.94006
SPAG16		0.95082
TFPI		0.96974
TIMP3		0.82583
S100P		0.92107
DERL3		0.96498
ABCC11		0.93459
AIFM3		0.83440
GLTP		0.77756
IRF6		0.89446
SMAD3		0.97254
TSPAN5		0.98947

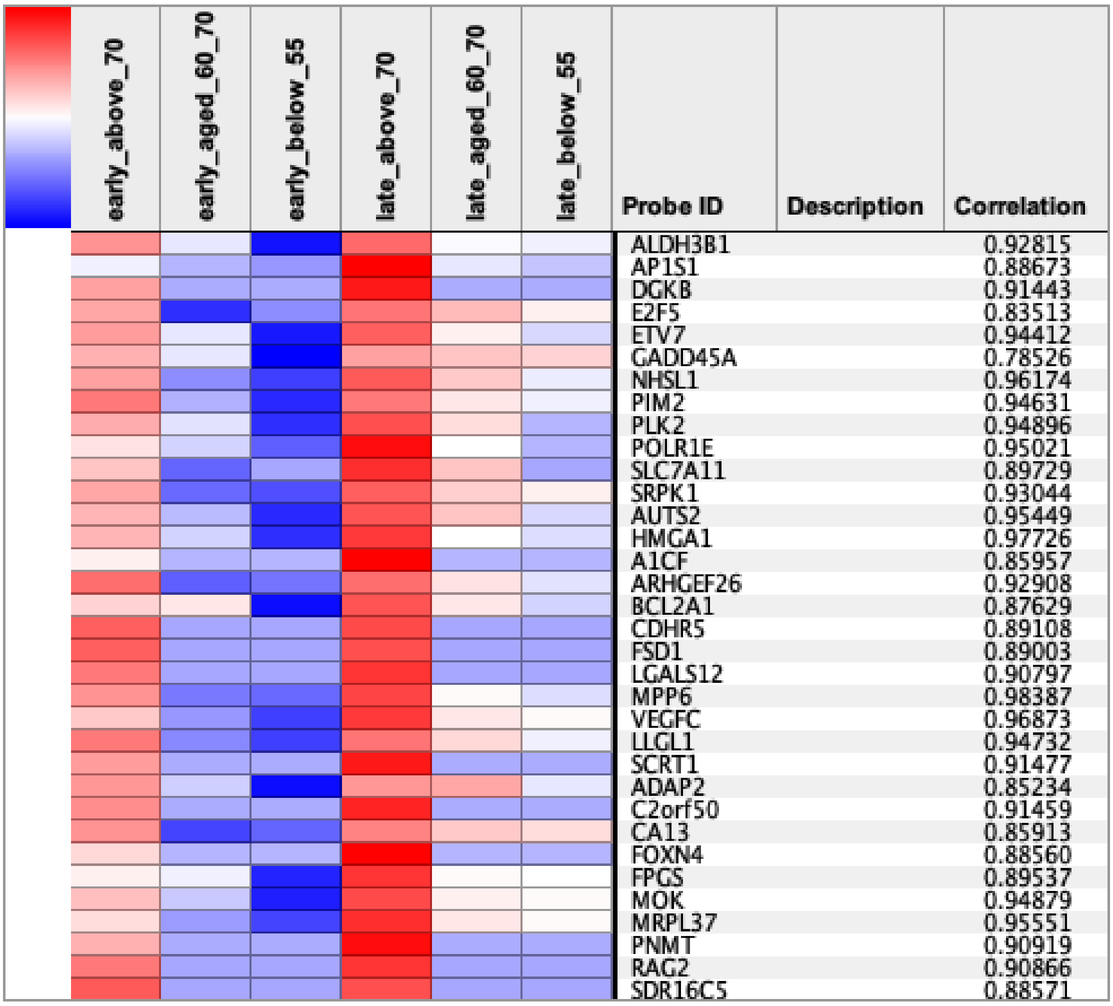

early_above_70
early_aged_60_70
early_below_55
late_above_70
late_aged_60_70
late_below_55
Probe ID
Description
Correlation
ALDH3B1 0.92815
AP1S1 0.88673
DGKB 0.91443
E2F5 0.83513
ETV7 0.94412
GADD45A 0.78526
NHSL1 0.96174
PIM2 0.94631
PLK2 0.94896
POLR1E 0.95021
SLC7A11 0.89729
SRPK1 0.93044
AUTS2 0.95449
HMGA1 0.97726
A1CF 0.85957
ARHGEF26 0.92908
BCL2A1 0.87629
CDHR5 0.89108
FSD1 0.89003
LGALS12 0.90797
MPP6 0.98387
VEGFC 0.96873
LLGL1 0.94732
SCRT1 0.91477
ADAP2 0.85234
C2orf50 0.91459
CA13 0.85913
FOXN4 0.88560
FPGS 0.89537
MOK 0.94879
MRPL37 0.95551
PNMT 0.90919
RAG2 0.90866
SDR16C5 0.88571

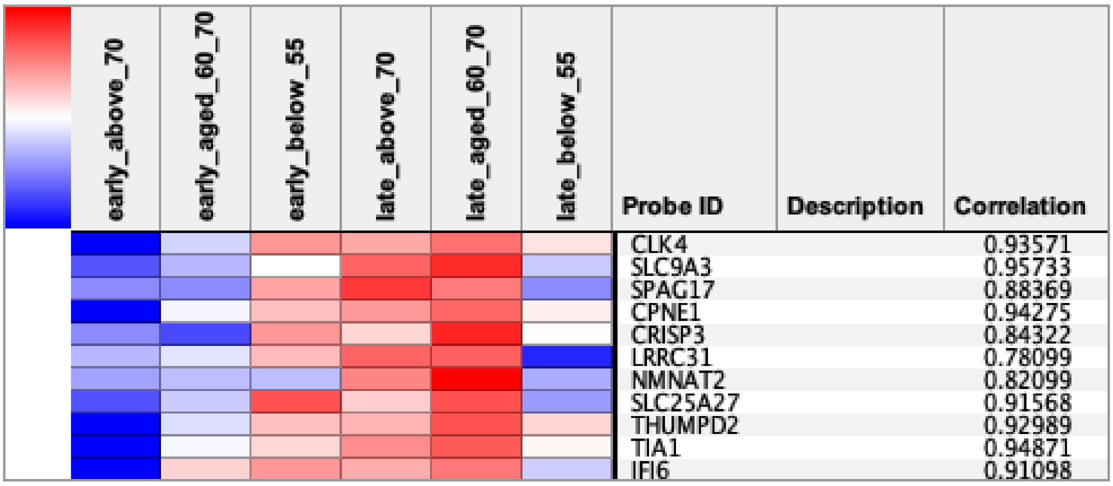

early_above_70
early_aged_60_70
early_below_55
late_above_70
late_aged_60_70
late_below_55
Probe ID
Description
Correlation
CLK4 0.93571
SLC9A3 0.95733
SPAG17 0.88369
CPNE1 0.94275
CRISP3 0.84322
LRRC31 0.78099
NMNAT2 0.82099
SLC25A27 0.91568
THUMPD2 0.92989
TIA1 0.94871
IFI6 0.91098

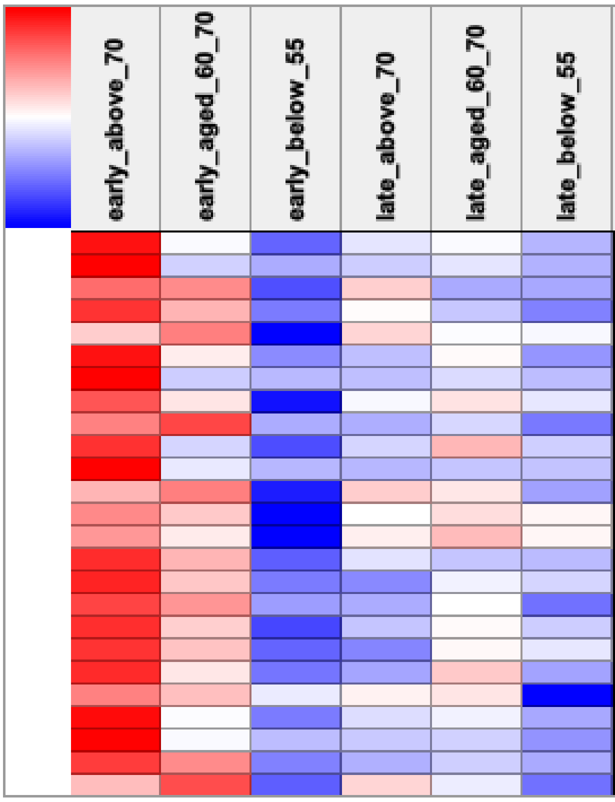

Probe ID	Description	Correlation
APC2		0.96801
CD36		0.87423
CDKN1A		0.87486
DENND2A		0.95879
EPHA2		0.70193
MAST2		0.96526
NT5DC3		0.84283
PKD2L1		0.91911
SLC27A5		0.80219
SYTL2		0.88584
TMBIM1		0.86423
WNT10A		0.79656
SYN3		0.82723
ABCC2		0.76254
CHI3L1		0.98022
EGR2		0.94049
LMO3		0.92746
STAT5A		0.98436
PPFIBP2		0.92918
ASB1		0.94909
BCL2L15		0.66282
CTSB		0.96343
FAM83E		0.89331
MFSD12		0.94995
ROBO3		0.74743

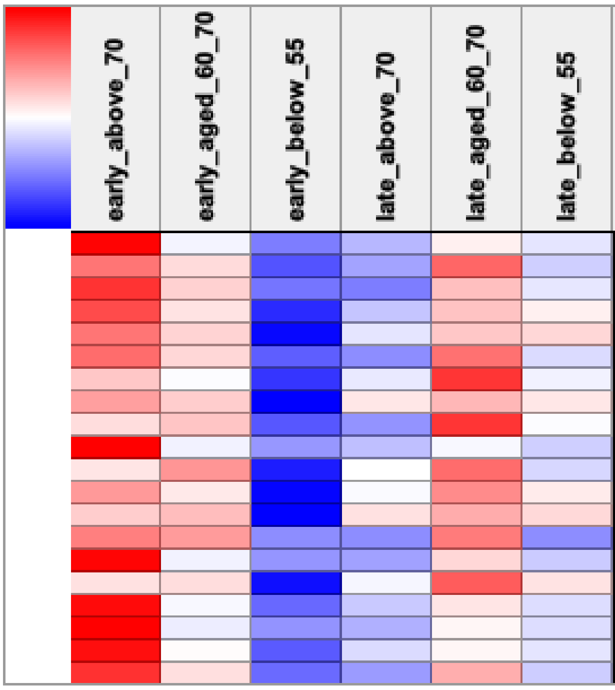

Probe ID	Description	Correlation
ATXN7L1		0.88173
GRAMD4		0.96401
GRK4		0.93701
NPAS3		0.98116
QSOX1		0.94086
SLC1A3		0.96038
ZNF862		0.84713
SNAI1		0.88471
AMPH		0.80829
COL12A1		0.81709
CPNE5		0.80500
FAM20A		0.92860
GLYATL2		0.81151
SLC14A2		0.84817
SLC40A1		0.87094
TRAF1		0.82307
MAST3		0.91627
MTHFR		0.84084
SLC43A2		0.90864
TPCN1		0.96391

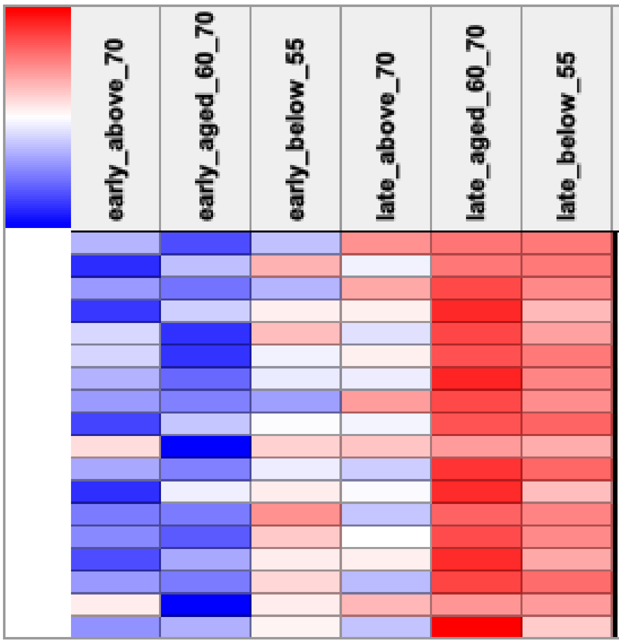

Probe ID	Description	Correlation
NUDCD1		0.88019
POLR3G		0.86661
UTP23		0.91912
WSB1		0.89093
C8orf37		0.91871
CENPQ		0.94715
CREBZF		0.97900
EPSTI1		0.88338
KHDRBS3		0.91682
SLC24A4		0.72270
STK3		0.96250
PPM1M		0.84038
SPIN4		0.90809
ACTL6A		0.98528
RTTN		0.95324
SERPINI1		0.95583
SH2D5		0.76668
TRAF5		0.89788

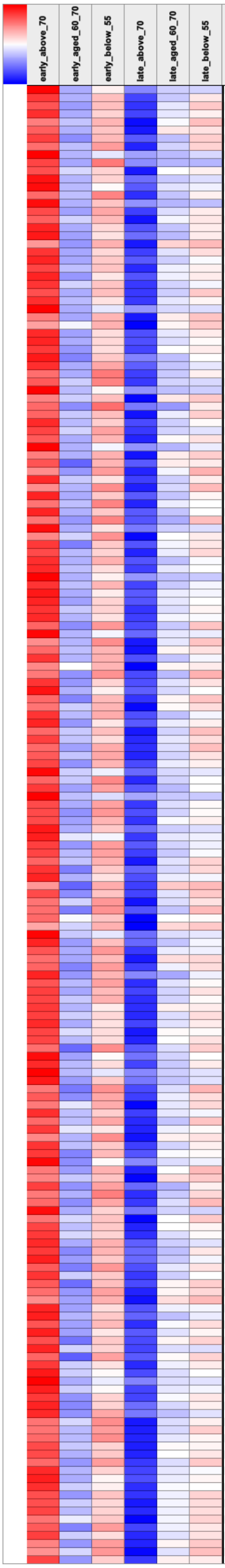

Probe ID	Descripti...	Correlation
AKAP1		0.91300
AP3B1		0.99578
ARHGAP18		0.98818
ARSB		0.99526
ATXN1		0.94479
CGN		0.97111
DHX29		0.97617
DIS3		0.97071
DIXDC1		0.84563
DSC2		0.87042
DYSF		0.97448
EHF		0.95382
ERC1		0.95811
ERLEC1		0.95980
ERLIN2		0.89344
EXOC6		0.99391
FAM184A		0.97495
FBXL4		0.98413
FGD4		0.96274
FNDC3B		0.90164
FTO		0.99465
G3BP2		0.98768
GCC2		0.99433
HEATR5B		0.98017
IFNAR1		0.98649
ITFG1		0.98838
JKAMP		0.98943
KANK1		0.87035
KIAA0586		0.94795
KIAA1217		0.86983
KLF3		0.98906
LRP6		0.98944
MDN1		0.98775
MGAT5		0.94879
MKLN1		0.97583
NUCB2		0.95190
PARP11		0.94946
PCM1		0.88684
PHF12		0.92670
PKP1		0.89199
PRDM5		0.97263
PRUNE2		0.99097
RAB27B		0.97326
RANBP2		0.98624
RFX3		0.88477
RHPN2		0.95052
RPL7L1		0.94835
RPRD1A		0.92867
RSPH3		0.98319
RTN4		0.93223
SAR1B		0.98524
SCARB2		0.95923
SEC23B		0.98403
SEC63		0.92672
SEL1L		0.94205
SEL1L3		0.92922
SLC4A7		0.98059
SNX25		0.98941
SOAT1		0.98532
SPG11		0.98916
SWT1		0.88444
TBC1D4		0.98831
TEX2		0.97098
TFDP2		0.98805
TGOLN2		0.99083
THRAP3		0.98454
TMED7		0.96075
TMEM131		0.93830
TMEM30A		0.93323
TMEM38A		0.97218
TMEM87B		0.98770
TSPAN31		0.90198
UFL1		0.93157
UTRN		0.99247
XPNPEP1		0.96818
YTHDC2		0.96239
ZC3HAV1L		0.95579
SNAP23		0.98176
SREBF2		0.98047
B2M		0.96543
PIGO		0.98897
RSPRY1		0.97397
SENP8		0.98423
SNTB2		0.99292
ZBTB4		0.92244
ABCA5		0.96477
ADPRHL1		0.97748
ANKRD31		0.82085
ARMCX5		0.99407
ATAD1		0.98070
ATP2C1		0.99371
AUH		0.95796
BEND6		0.93966
BMPR1B		0.97890
CACNA2D1		0.98819
DDX24		0.97592
DSG2		0.97585
EDNRB		0.98273
EPC1		0.86675
FBXO30		0.98562
GALC		0.95838
IBTK		0.93966
IQCH		0.93206
IRAK3		0.87311
KCNMA1		0.90733
LYPLAL1		0.97440
MDFIC		0.98067
MED23		0.95681
MIER3		0.97456
MPP1		0.93644
MPP5		0.99006
OPHN1		0.99738
PEX7		0.98973
PRDM2		0.97799
PSEN1		0.98603
RFFL		0.99058
RTN1		0.96727
SF3A1		0.98528
SLC30A5		0.93512
SLC35A5		0.94479
SLC35D1		0.98803
SOX5		0.88816
SRBD1		0.95539
STAM2		0.93460
STEAP2		0.98458
SYDE2		0.94190
TBC1D9		0.99144
TM9SF2		0.96681
TMC5		0.96558
WASF3		0.93599
ZDHHC2		0.99499
ZFX		0.98214
ZKSCAN1		0.92408
ZNF827		0.95798
ZNF747		0.92403
BNIP3		0.96318
HSPA5		0.95008
KIF5B		0.96399
MOAP1		0.94723
PITPNA		0.94611
SYT17		0.98966
COG7		0.98389
PYGO1		0.97571
ABHD3		0.97341
ACLY		0.97465
ADAM9		0.97269
AP1G1		0.98931
AP3M1		0.95775
ASAH2B		0.96774
ASRGL1		0.91920
CASP7		0.98132
CBR1		0.97145
CCDC87		0.99102
COG5		0.98060
CTNND1		0.96409
DIP2A		0.96909
EPB41		0.93913
FAN1		0.98330
FICD		0.96436
FITM2		0.90087
FLVCR1		0.96355
KBTBD4		0.98941
KLLN		0.97201
MAGED2		0.95300
MFAP3L		0.95089
NCSTN		0.96530
PHACTR4		0.97325
POGLUT1		0.98168
POLR3E		0.99029
PPP1R21		0.97167
RASSF6		0.99165
RBMXL1		0.97329
SEC24C		0.97075
SERINC5		0.99088
SH3PXD2B		0.97830
TM2D1		0.99121
TRIM62		0.93597
TSPYL4		0.97535
TTC30A		0.98627
TTC30B		0.94611
VRK3		0.91931
ZNF407		0.98605

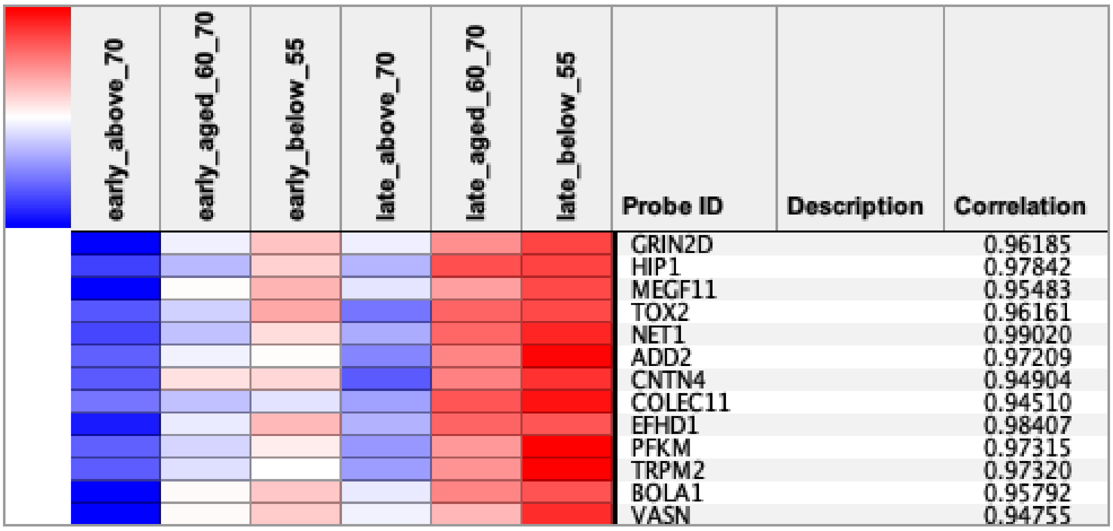
early_above_70
early_aged_60_70
early_below_55
late_above_70
late_aged_60_70
late_below_55
Probe ID
Description
Correlation
GRIN2D 0.96185
HIP1 0.97842
MEGF11 0.95483
TOX2 0.96161
NET1 0.99020
ADD2 0.97209
CNTN4 0.94904
COLEC11 0.94510
EFHD1 0.98407
PFKM 0.97315
TRPM2 0.97320
BOLA1 0.95792
VASN 0.94755

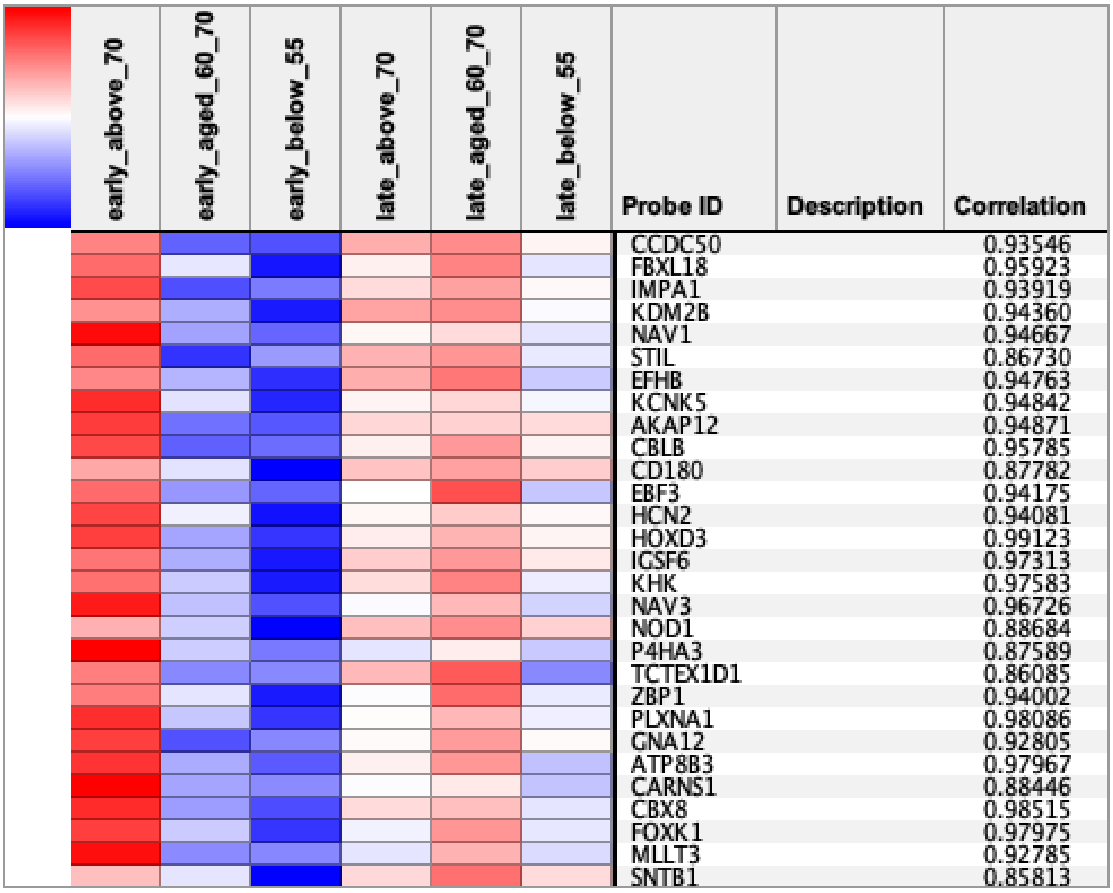
early_above_70
early_aged_60_70
early_below_55
late_above_70
late_aged_60_70
late_below_55
Probe ID
Description
Correlation
CCDC50 0.93546
FBXL18 0.95923
IMPA1 0.93919
KDM2B 0.94360
NAV1 0.94667
STIL 0.86730
EFHB 0.94763
KCNK5 0.94842
AKAP12 0.94871
CBLB 0.95785
CD180 0.87782
EBF3 0.94175
HCN2 0.94081
HOXD3 0.99123
IGSF6 0.97313
KHK 0.97583
NAV3 0.96726
NOD1 0.88684
P4HA3 0.87589
TCTEX1D1 0.86085
ZBP1 0.94002
PLXNA1 0.98086
GNA12 0.92805
ATP8B3 0.97967
CARNS1 0.88446
CBX8 0.98515
FOXK1 0.97975
MLLT3 0.92785
SNTB1 0.85813

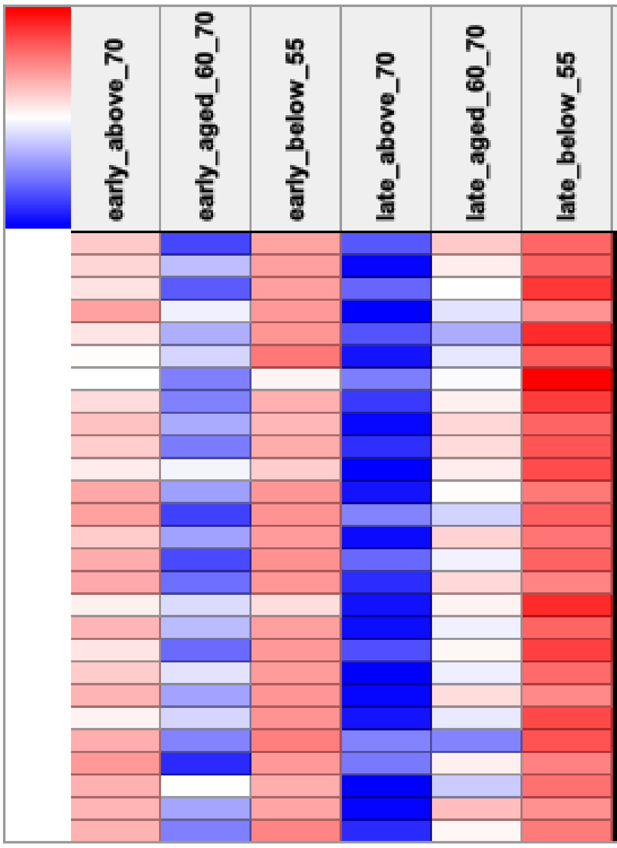

Probe ID	Description	Correlation
AP3S2		0.94308
DGKE		0.98488
FOXO3		0.95550
KDM4B		0.91995
ME1		0.93710
PLS1		0.95310
RAB18		0.86705
SLAIN2		0.98795
TBCK		0.98205
THOC2		0.98747
TMEM117		0.92543
UBR3		0.98820
UBXN4		0.90785
USP34		0.98160
CD2AP		0.94620
MBD5		0.97041
AAGAB		0.93625
FNDC3A		0.98803
FOLH1		0.97522
LARP4B		0.96076
PPP3CB		0.97511
SCPEP1		0.96498
RPL3L		0.87213
MANEA		0.89396
PMEPA1		0.91521
TEAD1		0.95297
ZMPSTE24		0.98503

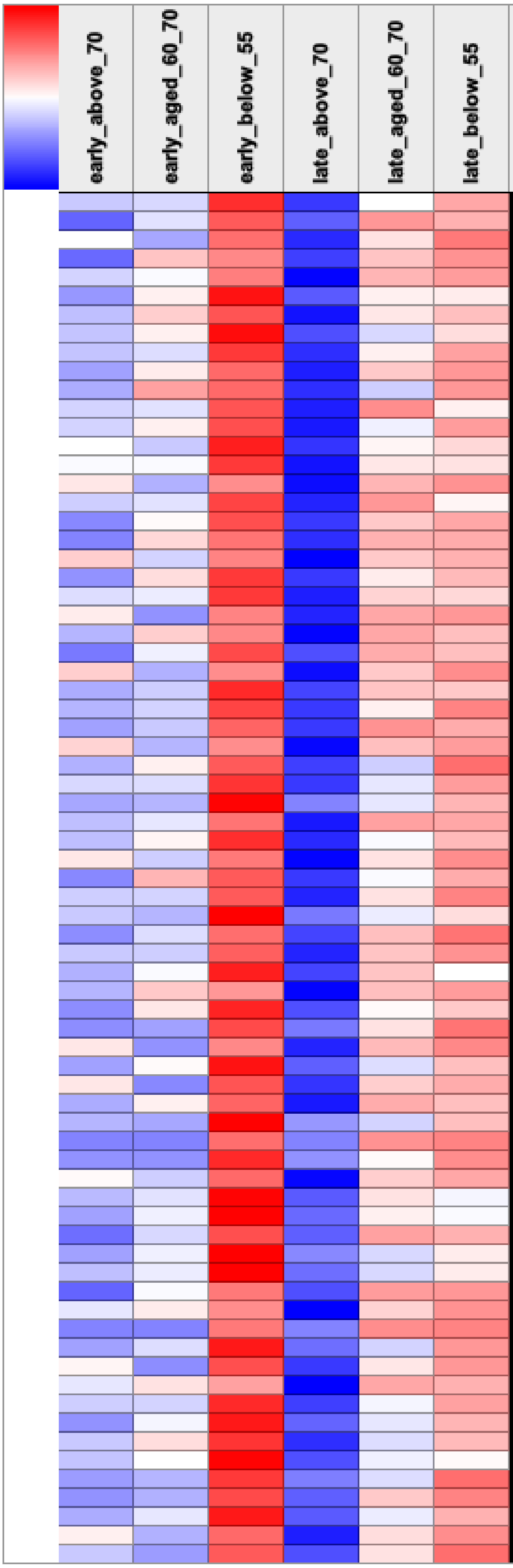

Probe ID	Descripti...	Correlation
AMPD3		0.99032
CACNA1D		0.92225
CAST		0.93194
CCNJL		0.88727
FLVCR2		0.95597
GGT1		0.93559
GHR		0.95508
HECW1		0.93015
ITPR1		0.99674
JAKMIP1		0.96882
LAMC2		0.87107
MCF2L		0.92837
NME1-N...		0.96967
PDE8B		0.95009
PXDN		0.95053
SEC22C		0.89647
SLC25A12		0.93401
SOBP		0.97418
SYN2		0.93131
TACC1		0.88580
TUBA4A		0.96460
ZDHHC6		0.97915
ZNF304		0.88255
MSX2		0.91250
RBM20		0.95065
SLC50A1		0.86999
SVIL		0.98276
ANKRD6		0.98818
APCDD1		0.95386
ATP7B		0.87622
COCH		0.92906
CRYZ		0.97656
DNAJA4		0.91531
ECE1		0.96294
ECHDC1		0.98132
EFR3A		0.92101
GALNT14		0.91649
GPR180		0.98583
LGALSL		0.90203
LRRC7		0.95682
LRRFIP1		0.98622
MAP7		0.94340
MCOLN3		0.90673
MYO1G		0.95283
ORC3		0.92393
PHIP		0.87964
PLIN2		0.93957
PPM1B		0.90939
PRTFDC1		0.96482
RIMS1		0.86841
SLC5A4		0.85370
SPAG6		0.89650
STK17B		0.95467
STXBP5L		0.92545
TESC		0.91138
TGDS		0.93620
AMD1		0.87190
B3GALNT1		0.90415
ID4		0.91323
PPP1R10		0.92929
FAM178B		0.84246
ACOT13		0.93522
ARHGAP12		0.92652
CDCP1		0.88712
ENTPD2		0.98422
F3		0.94697
ITPKB		0.94747
LMAN2L		0.92275
PEX13		0.90886
PUS10		0.95640
STK39		0.96599
TBC1D8		0.93819
ZNF292		0.95063

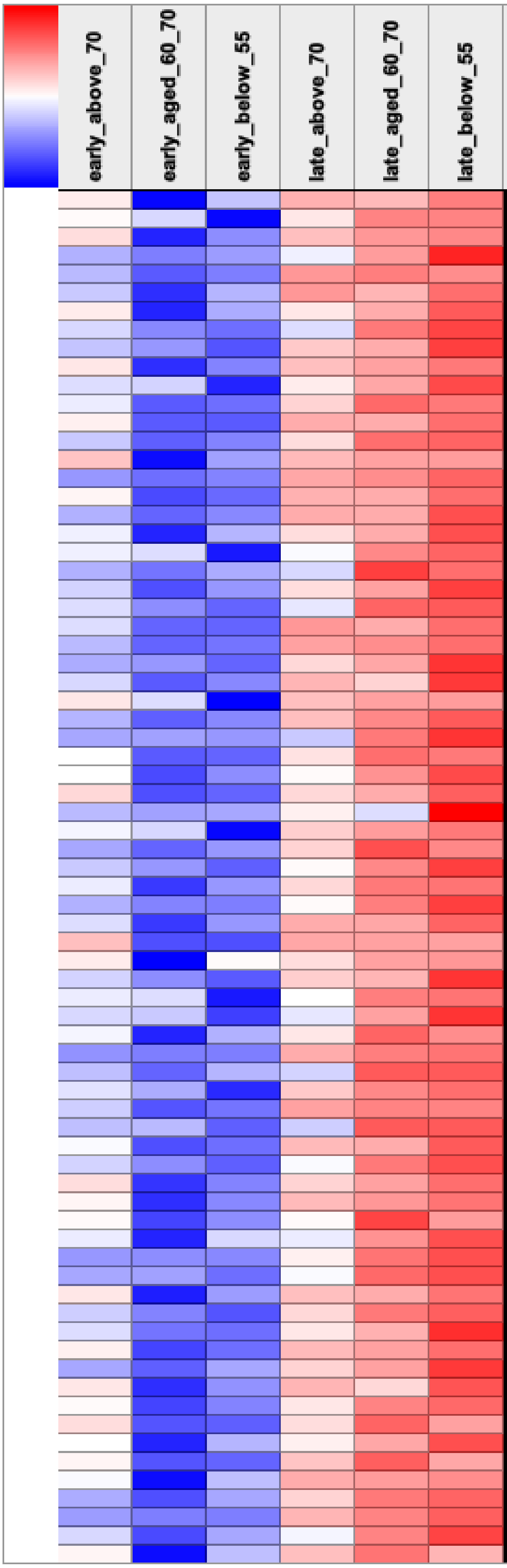

Probe ID	Descripti...	Correlation
ATAD2		0.88528
BTK		0.84096
C1GALT1		0.94207
CDK17		0.93748
CENPL		0.95498
CEP70		0.92860
DCK		0.94597
HHEX		0.95480
LACTB2		0.96067
MPHOSPH9		0.95932
OPTN		0.88235
OSBPL10		0.98813
PRDM16		0.96774
RNF19A		0.99150
SKAP2		0.88520
SLC19A2		0.95399
SLC37A3		0.97494
SLCO5A1		0.96739
SPIN1		0.95551
TGFBI		0.84884
TRIM14		0.89938
UBE2W		0.98465
UNC13A		0.95658
VEZT		0.96775
SPRY4		0.97373
AIDA		0.95190
C16orf72		0.95549
GPSM3		0.78111
ZNRF2		0.98490
ABI3BP		0.88456
ANGPT2		0.98540
APPL1		0.97997
ATP6V1C1		0.96564
BANK1		0.77133
CD86		0.84568
CDON		0.94176
CDR2		0.96464
CDYL		0.98264
CTNNAL1		0.96497
DEK		0.97099
DNAJC5B		0.89787
FAM124B		0.79614
GLIPR1		0.96289
IL12RB1		0.84569
IL2RA		0.88158
KRAS		0.94213
NDE1		0.94493
NLGN4X		0.91293
PDGFB		0.93524
PHF6		0.97432
POSTN		0.88377
PTBP3		0.98299
QKI		0.96793
SMC2		0.96257
STAU2		0.97090
TRIM32		0.93410
ZFP30		0.91007
SHC4		0.94482
BICD2		0.93708
KIAA0825		0.94361
ANP32E		0.98242
ATP6V1G2		0.97841
E2F3		0.97641
FAR1		0.95809
MASTL		0.92832
PATL1		0.98578
PTCH1		0.93870
RAB6A		0.94744
RIPK2		0.94528
RMI1		0.90599
SMC6		0.96792
ZBTB34		0.96080
ZBTB43		0.96809
ZNF217		0.88040

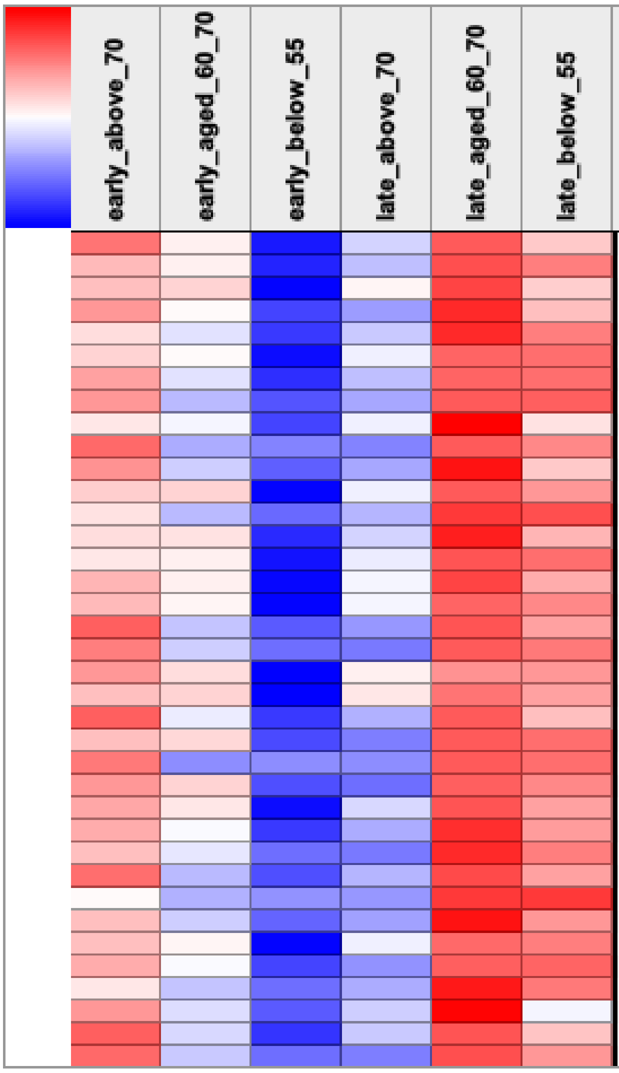

Probe ID	Description	Correlation
AHR		0.95293
CASP10		0.98898
CCDC3		0.91659
KIF26A		0.97591
MAP3K12		0.97749
MAP4K1		0.95639
MOB3B		0.98675
PDZD2		0.96420
PDZD7		0.90098
ENC1		0.91482
ADCY3		0.96020
CFH		0.94514
ECM2		0.93626
ESR1		0.95937
FGD2		0.95052
FGF1		0.96466
JAK3		0.95795
MAP7D3		0.95115
MDGA1		0.94734
MYO1F		0.90331
PLCB2		0.90463
PLXND1		0.96190
PRRX1		0.95311
PZP		0.88888
TSPAN32		0.94603
VASH1		0.97463
ZBTB46		0.99577
GIMAP1		0.95608
TTYH2		0.96594
FBN1		0.86659
KIF21B		0.96926
LAIR1		0.95861
MMRN2		0.97584
RCAN2		0.94459
SYNGAP1		0.90438
SYT11		0.95508
ZEB2		0.94180

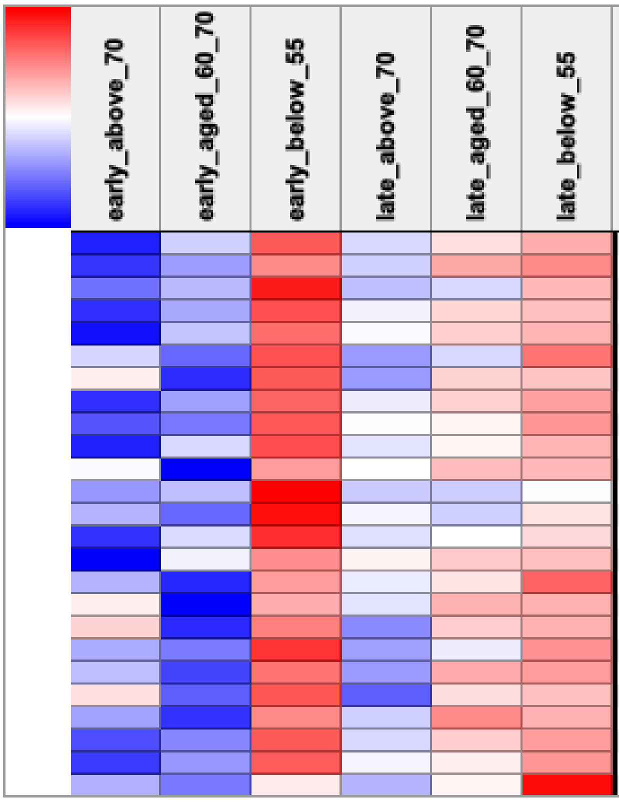

Probe ID	Description	Correlation
ASB3		0.89537
CNTLN		0.92192
HPCAL1		0.91529
MYO16		0.93075
REEP1		0.87643
WNT3		0.89665
CCDC112		0.82251
MZT1		0.94410
FADS1		0.97311
ZNF608		0.88138
HOXA7		0.77122
LNX1		0.81629
PM20D2		0.88073
RARS2		0.86646
RBX1		0.74651
RCN2		0.89659
SERTAD4		0.73541
SH3RF1		0.75052
ARSK		0.95477
ZNF550		0.93046
FAM169A		0.76832
GPR160		0.90974
PKD1L1		0.98133
RFESD		0.95260
TWF1		0.73154

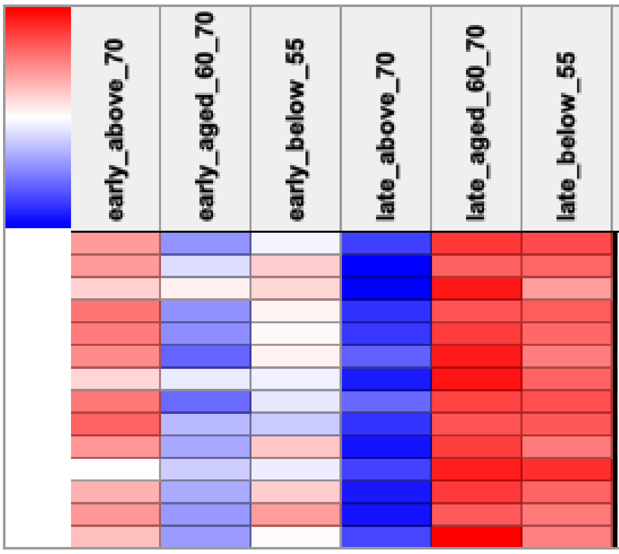

Probe ID	Description	Correlation
CCDC88A		0.99080
HDAC4		0.96700
KHNYN		0.92283
RORA		0.98541
TMEM168		0.99025
ZBTB32		0.96482
ADAM28		0.96304
CEP170		0.95355
HSPG2		0.96059
IFIH1		0.98867
ZEB1		0.93898
ZNF175		0.99252
FAT1		0.96213
PRKG1		0.97635

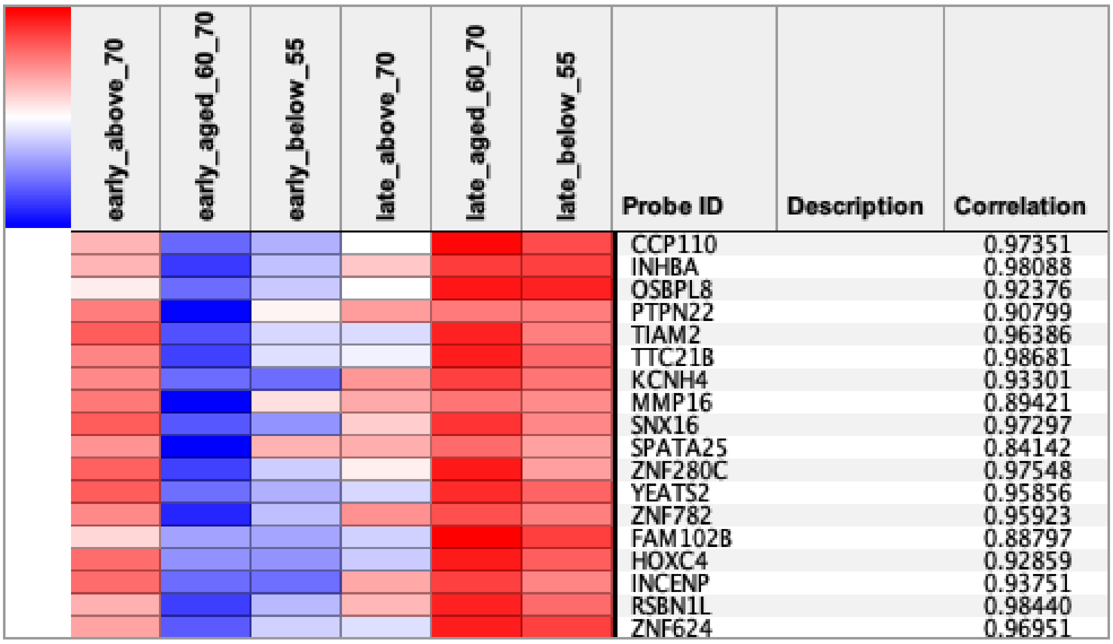
early_above_70
early_aged_60_70
early_below_55
late_above_70
late_aged_60_70
late_below_55
Probe ID
Description
Correlation
CCP110 0.97351
INHBA 0.98088
OSBPL8 0.92376
PTPN22 0.90799
TIAM2 0.96386
TTC21B 0.98681
KCNH4 0.93301
MMP16 0.89421
SNX16 0.97297
SPATA25 0.84142
ZNF280C 0.97548
YEATS2 0.95856
ZNF782 0.95923
FAM102B 0.88797
HOXC4 0.92859
INCENP 0.93751
RSBN1L 0.98440
ZNF624 0.96951

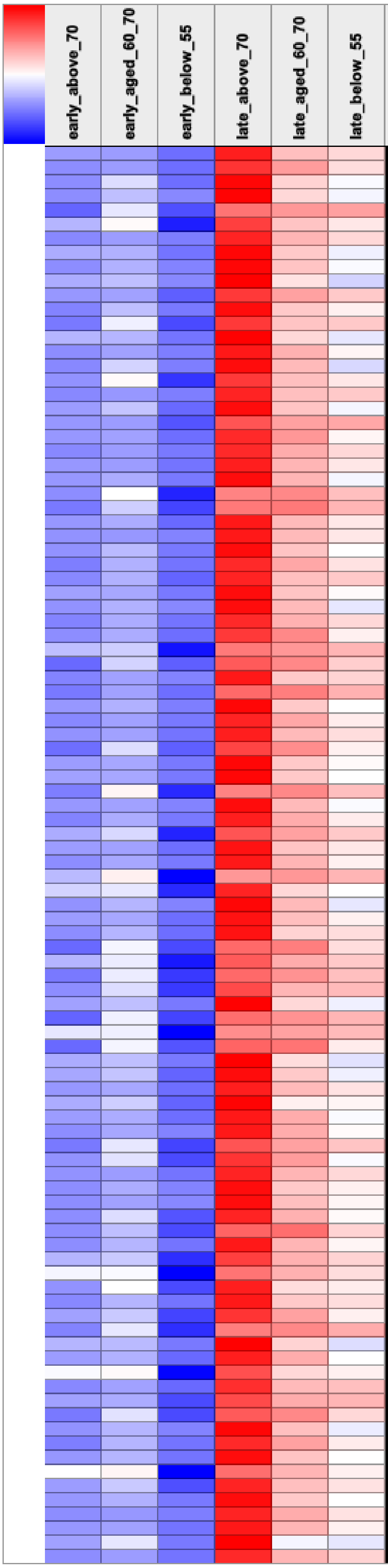

Probe ID	Descripti...	Correlation
ANLN		0.98889
ASPM		0.98785
BBC3		0.97839
BIRC5		0.96922
C1QTNF6		0.92259
CCM2		0.94920
CCNA2		0.98594
CDC25B		0.97920
CDKN3		0.97916
CHEK1		0.93734
DHH		0.98743
E2F1		0.98930
ERF		0.97495
GGCT		0.96420
HMMR		0.98797
HOXB3		0.96461
KLF10		0.96765
MAL2		0.97698
MALSU1		0.98853
MSL3		0.95841
MTHFD1L		0.98762
NCAPG		0.98592
NUF2		0.98911
PABPC1		0.98385
PKIB		0.90673
PMAIP1		0.93323
PRR11		0.99537
RPA3		0.98204
SKA1		0.98589
SPC25		0.99261
WDR62		0.99162
TCF19		0.98320
CDC25C		0.97294
CIT		0.99198
LIG1		0.98530
NDUFA4L2		0.91604
ARR3		0.96605
BCAS4		0.98032
CDC7		0.93833
CDCA5		0.98333
CENPF		0.98934
CEP55		0.99013
CLEC2D		0.97460
DTL		0.98180
GINS1		0.97895
IGFBP3		0.90134
KIF20A		0.98019
KIF4A		0.99045
LY86		0.96267
MCM10		0.98900
NUSAP1		0.99006
PCDHB10		0.84765
SPP1		0.94560
CCNB2		0.97689
KIF2C		0.99078
MRPS6		0.98820
PTCH2		0.93723
AIF1		0.94659
RAB3IL1		0.94338
AMDHD2		0.97316
AURKB		0.97031
C11orf80		0.93138
C1QB		0.83334
CDK18		0.93848
CENPM		0.95659
E2F2		0.98564
EME1		0.99382
ENTPD8		0.96510
ESPL1		0.98951
EXO1		0.98608
FTH1		0.96717
GPR35		0.98438
GTSE1		0.98837
HOXB6		0.98291
MCM3		0.98036
MCM5		0.99209
MGAT4B		0.95466
NCAPH		0.99389
NEIL3		0.98174
NKG7		0.85049
NRGN		0.96667
NTM		0.99057
OASL		0.99296
PKN3		0.91814
RAD51		0.95867
RFC4		0.99232
RGS16		0.86429
RRM2		0.98578
SHCBP1		0.97496
SHMT2		0.96476
SKA3		0.97800
SPAG5		0.99239
TACC3		0.98796
TBX2		0.83775
TCTEX1D2		0.99694
TONSL		0.98501
TOP2A		0.98366
TPX2		0.99153
WDR34		0.92387
WDR76		0.98774

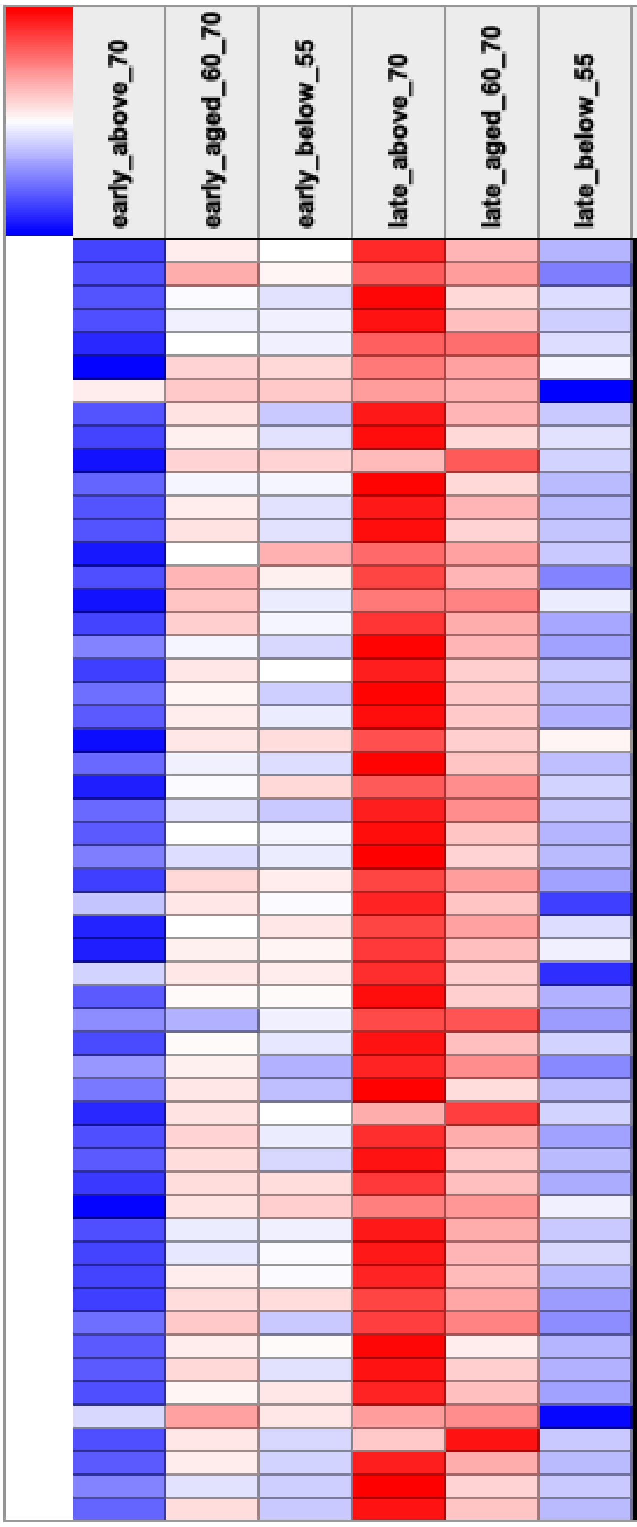

Probe ID	Descripti...	Correlation
ANKHD1		0.99856
ARL4A		0.94944
GTF3A		0.96330
HNRNPA1		0.98576
KCNIP2		0.94611
MAZ		0.89522
MRO		0.50681
MRPS25		0.98286
NOP16		0.96977
PABPC1L		0.80821
RPL6		0.97370
RPS13		0.99435
RPS20		0.98036
THOC1		0.91968
TSNAXIP1		0.96801
TWIST1		0.91289
RPS14		0.99414
RPS3A		0.96657
RPL38		0.98570
RPS29		0.96889
RPL24		0.98976
A1BG		0.91517
ASGR1		0.97622
BMX		0.95038
BZW2		0.96404
C18orf21		0.98896
CDCA7		0.94896
CHKA		0.98680
CTNNA2		0.84933
PIN4		0.96724
PKN1		0.95732
RBP4		0.80302
RPS25		0.98696
GDF9		0.88033
SOX12		0.98391
ALKBH2		0.92598
CPSF4		0.95069
CSAD		0.83754
KCNAB3		0.99423
NHP2		0.98471
NPM3		0.98329
PAQR8		0.89402
POLR2H		0.98649
RCE1		0.97914
RPL27		0.99689
RPL31		0.98320
RPL35A		0.95955
RPL7A		0.96756
RPL8		0.98635
RPS18		0.99032
S100A3		0.63751
SEC31B		0.73818
TIMM9		0.99210
ZNF3		0.93661
ZNF581		0.97701

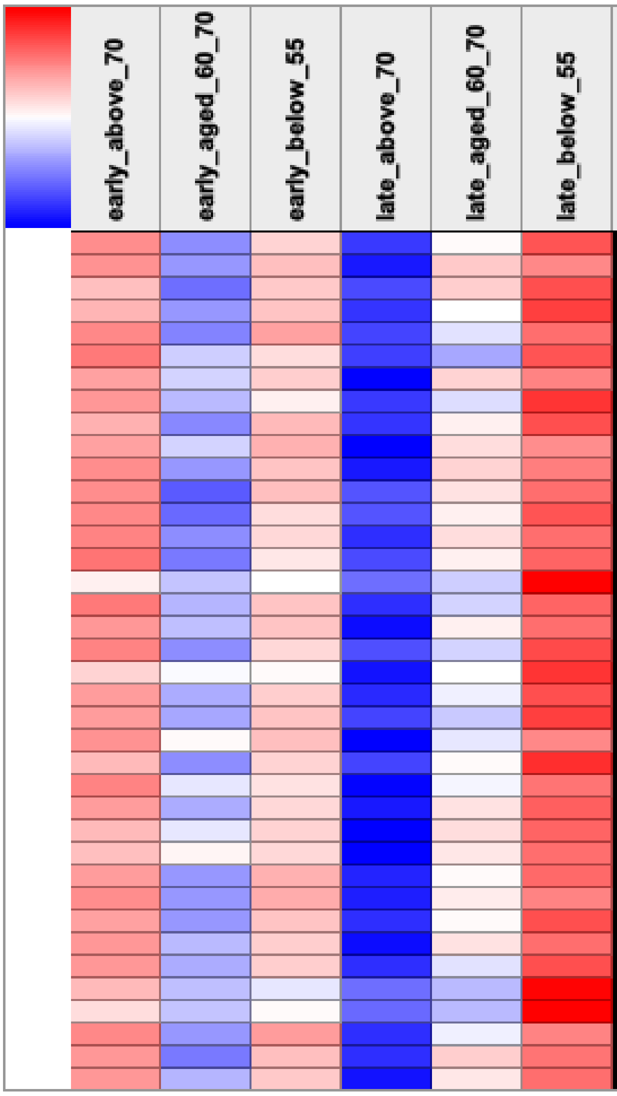

Probe ID	Description	Correlation
AKAP11		0.99261
DOCK9		0.96870
GBE1		0.95608
HSDL2		0.99266
KBTBD7		0.96688
LGMN		0.94035
LRRK1		0.96137
OSTM1		0.97889
PRKAR1A		0.98815
SCRN1		0.95407
SLC39A9		0.97951
TMEM106B		0.94960
TNKS2		0.96582
YME1L1		0.98143
ZFYVE16		0.96731
HSPA1A		0.86653
TAF13		0.98243
ABHD13		0.99145
BTBD3		0.97475
CNN3		0.92770
HOOK1		0.99853
IDH1		0.97617
KIT		0.93158
NPTN		0.98105
PDE8A		0.96135
PIKFYVE		0.99465
SNX1		0.95745
UNC79		0.92649
USP45		0.99096
ZMYND11		0.97759
RAB8B		0.99667
LRRC8B		0.98649
MAP2K1		0.99505
PNPO		0.88108
SCAMP5		0.87700
SCCPDH		0.97176
TC2N		0.96865
ZNF846		0.99136

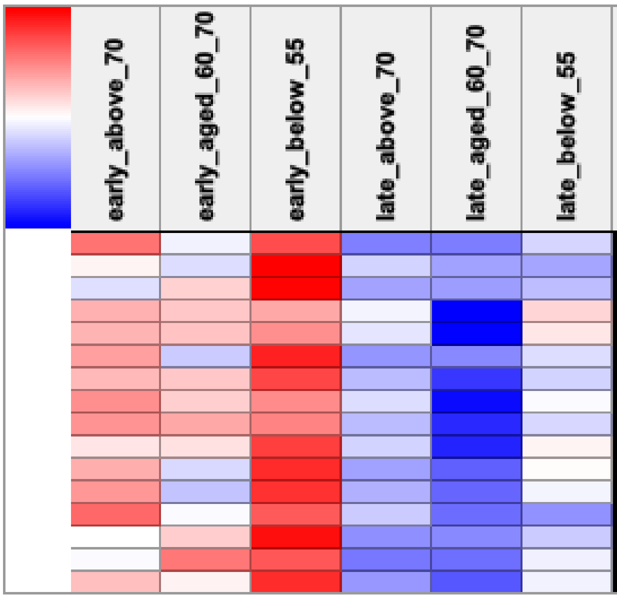

Probe ID	Description	Correlation
GCNT2		0.93380
SDK1		0.85811
TPT1		0.86632
CPO		0.83093
EYA4		0.89185
FKBP5		0.92989
IGSF1		0.99030
NDP		0.91779
PAGE4		0.93435
SLC27A6		0.96330
SLC39A8		0.96399
ZNF655		0.94250
FOXA3		0.90452
CDC42EP5		0.92463
FAH		0.85853
SLC22A4		0.99162

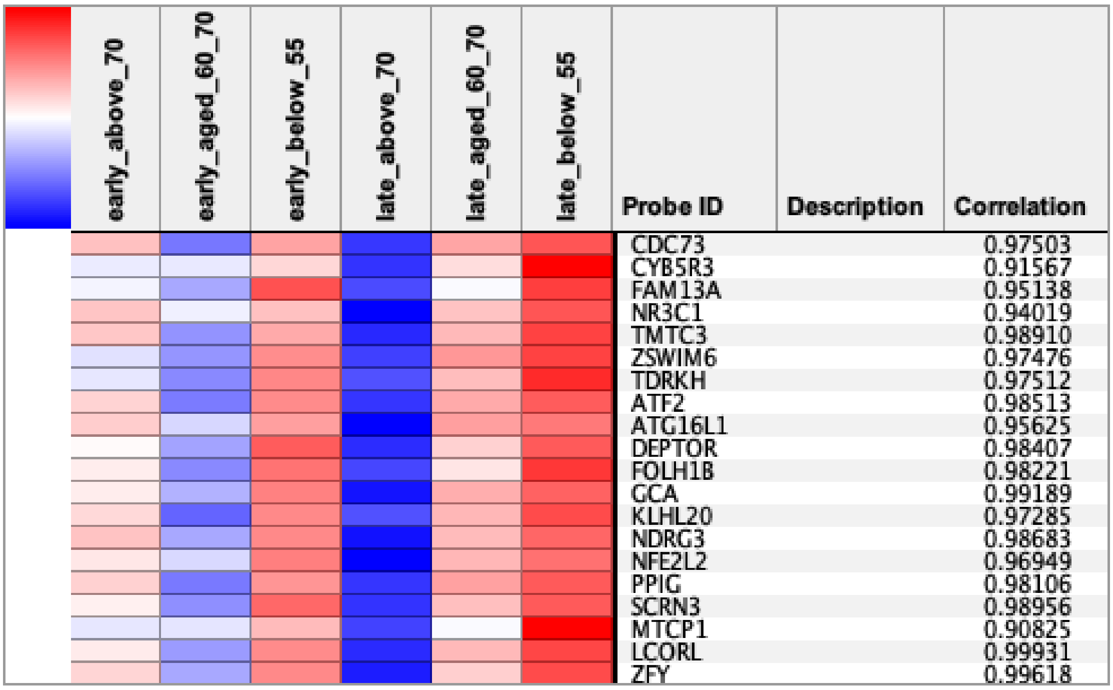
early_above_70
early_aged_60_70
early_below_55
late_above_70
late_aged_60_70
late_below_55
Probe ID
Description
Correlation
CDC73 0.97503
CYB5R3 0.91567
FAM13A 0.95138
NR3C1 0.94019
TMTC3 0.98910
ZSWIM6 0.97476
TDRKH 0.97512
ATF2 0.98513
ATG16L1 0.95625
DEPTOR 0.98407
FOLH1B 0.98221
GCA 0.99189
KLHL20 0.97285
NDRG3 0.98683
NFE2L2 0.96949
PPIG 0.98106
SCRN3 0.98956
MTCP1 0.90825
LCORL 0.99931
ZFY 0.99618

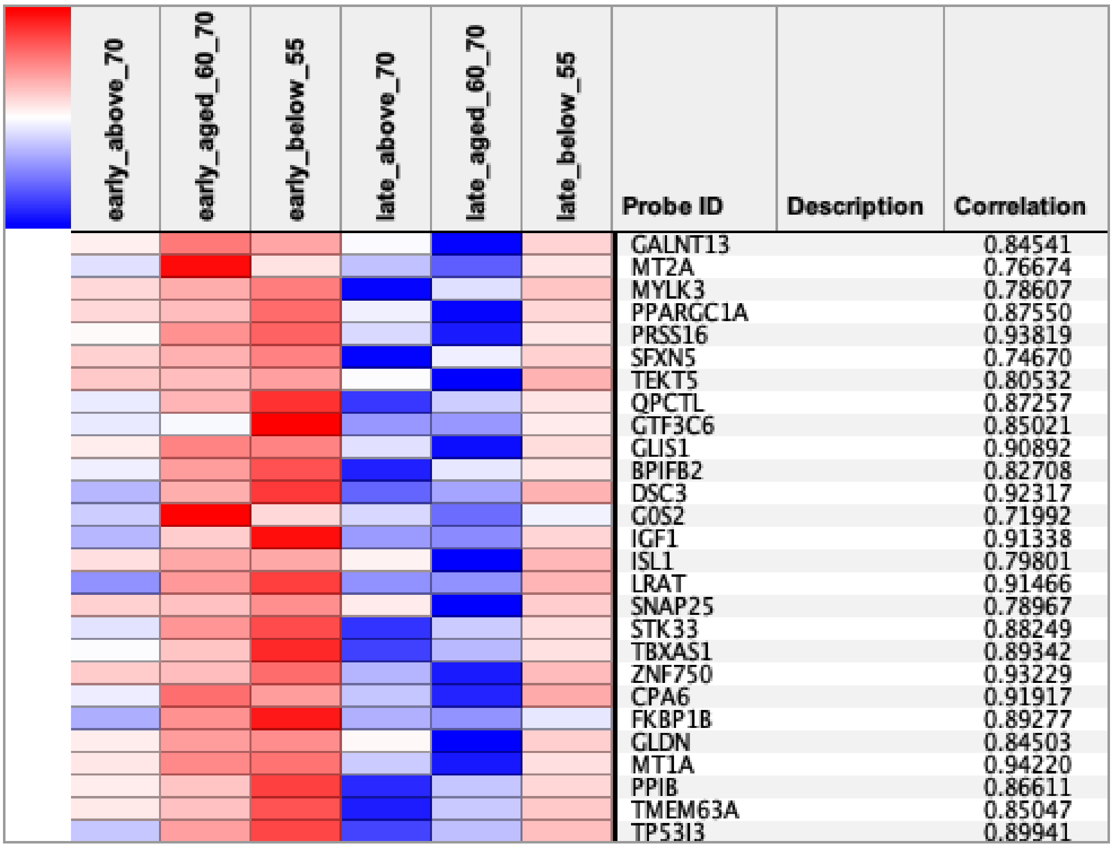
early_above_70
early_aged_60_70
early_below_55
late_above_70
late_aged_60_70
late_below_55
Probe ID
Description
Correlation
GALNT13 0.84541
MT2A 0.76674
MYLK3 0.78607
PPARGC1A 0.87550
PRSS16 0.93819
SFXN5 0.74670
TEKT5 0.80532
QPCTL 0.87257
GTF3C6 0.85021
GLIS1 0.90892
BPIFB2 0.82708
DSC3 0.92317
G0S2 0.71992
IGF1 0.91338
ISL1 0.79801
LRAT 0.91466
SNAP25 0.78967
STK33 0.88249
TBXAS1 0.89342
ZNF750 0.93229
CPA6 0.91917
FKBP1B 0.89277
GLDN 0.84503
MT1A 0.94220
PPIB 0.86611
TMEM63A 0.85047
TP53I3 0.89941

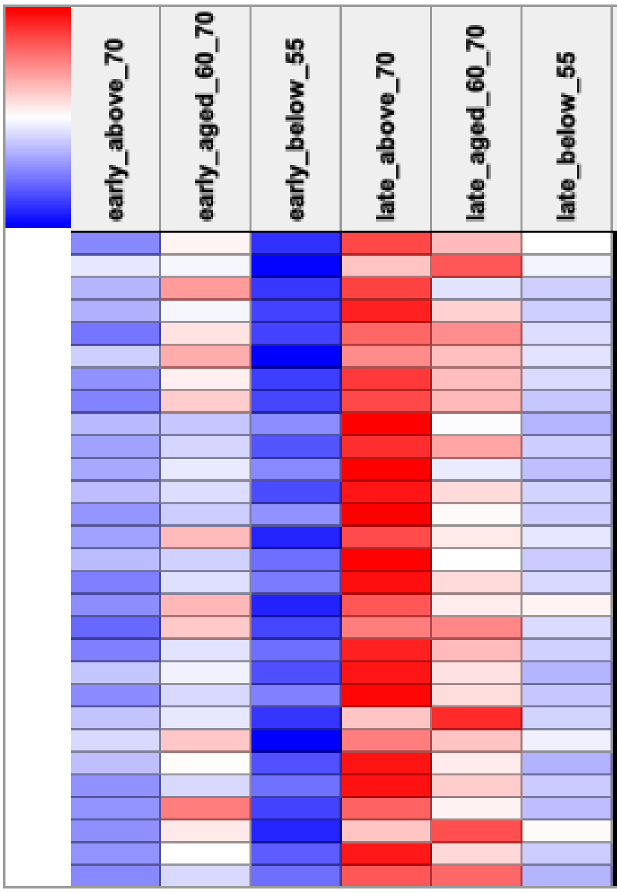

Probe ID	Description	Correlation
ABHD11		0.98481
CABLES1		0.78402
IER3		0.90020
PRR7		0.99279
ADAMTS13		0.96395
IER5		0.89412
SPIRE2		0.99834
GIGYF1		0.98344
GRB7		0.89504
HAGHL		0.97886
LAG3		0.90859
MAK		0.97424
ONECUT2		0.91281
GNB1L		0.96262
BRI3		0.93064
CDT1		0.95728
COMTD1		0.93800
ITIH4		0.93102
MXD3		0.97571
NDUFV1		0.97218
NUDT1		0.95001
PDE1B		0.76360
PLK3		0.91092
POLD2		0.97079
PRELID1		0.96873
S100A13		0.89151
THBS3		0.80787
TSEN54		0.98822
ZNF783		0.92310

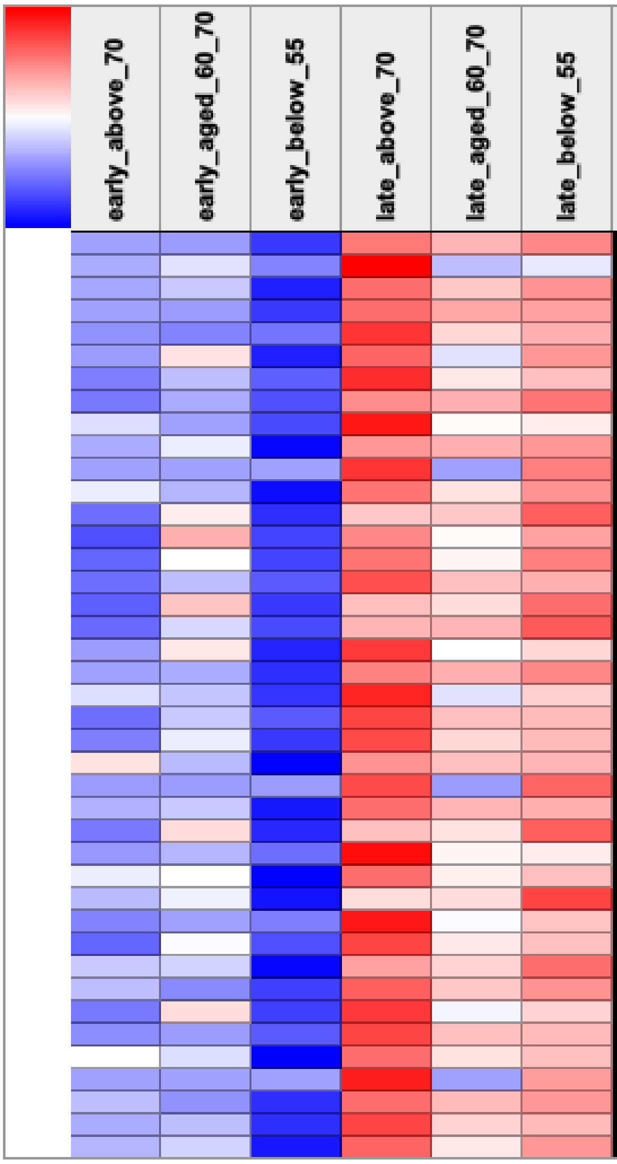

Probe ID	Description	Correlation
AMIGO2		0.96427
CCNO		0.79267
CD72		0.98987
FAM83D		0.96214
MCM6		0.93614
MLXIPL		0.94897
MNS1		0.96937
NRXN3		0.94555
SLC25A19		0.90152
YWHAH		0.95100
ANGPTL3		0.83321
ASS1		0.94947
ATP1B1		0.87638
CHRDL2		0.86783
CPS1		0.95890
DGAT2		0.97242
ERP27		0.84878
GNLY		0.90044
METRN		0.94110
MMD		0.96741
NFKBIA		0.93021
NGF		0.96768
RAB32		0.98527
RELT		0.85799
STMN2		0.82744
STX2		0.97773
TMEM176A		0.88327
HES6		0.91470
TLCD1		0.92215
ADM		0.84645
AKR1C3		0.92690
IER5L		0.95778
IL32		0.93950
KPNA2		0.96348
LCN12		0.92857
LMNB2		0.96585
OSCAR		0.91496
SHOX2		0.82870
SYK		0.96179
TMEM139		0.98869
ZFP36L1		0.99066

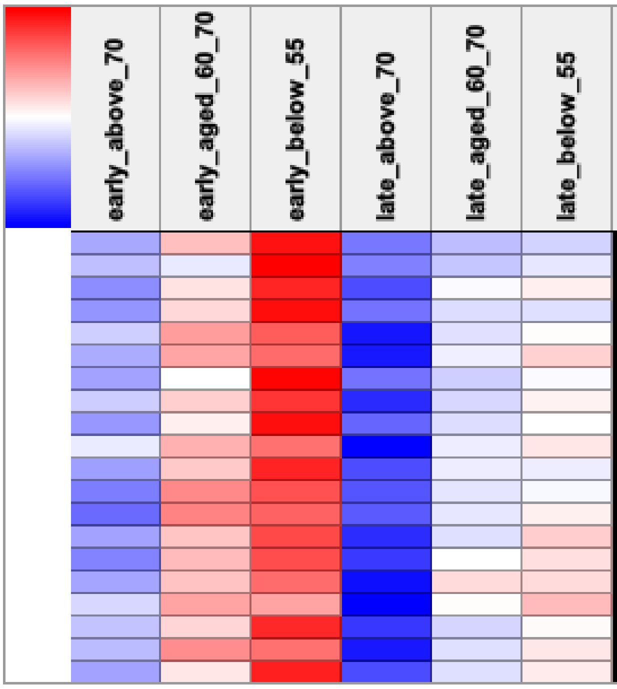

Probe ID	Description	Correlation
CYP3A5		0.94622
FAM81A		0.89578
MGST2		0.98299
MRAP2		0.95768
TRIM38		0.97125
ALDH1L1		0.97063
AOX1		0.94512
CCDC85A		0.98820
CHRD		0.96741
FRMD6		0.93991
GNAO1		0.98966
NOS1		0.96413
ZDHHC15		0.94510
PRICKLE2		0.98924
SNAI2		0.98351
PLEKHG4B		0.95276
SPON1		0.87851
MOGS		0.99043
PNKP		0.96089
TRIM13		0.98689

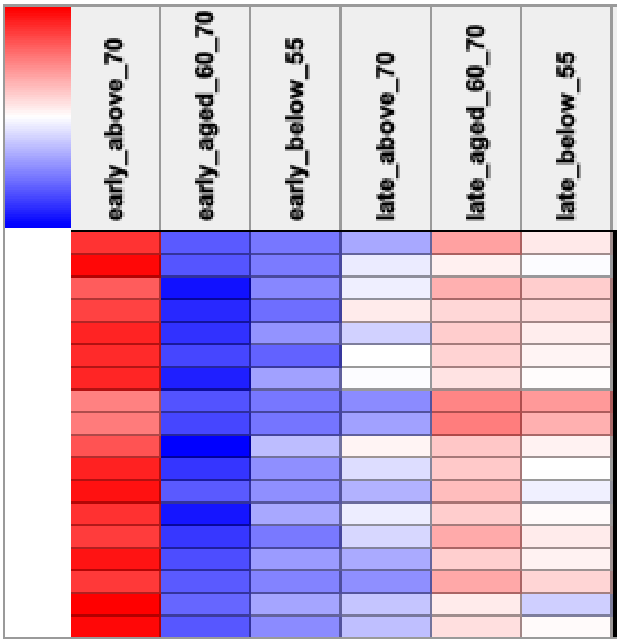

Probe ID	Description	Correlation
LPIN2		0.98131
PRDM4		0.97227
RABGEF1		0.97493
TRIM24		0.96699
GART		0.99788
GREB1L		0.97957
ME2		0.97663
MED13L		0.89223
RECK		0.92039
TFAM		0.94277
WWP1		0.99597
DTX3L		0.98168
LCLAT1		0.98452
METTL2B		0.99533
NEU3		0.98567
PLEKHA7		0.96868
STK38L		0.94139
ZNF445		0.98378

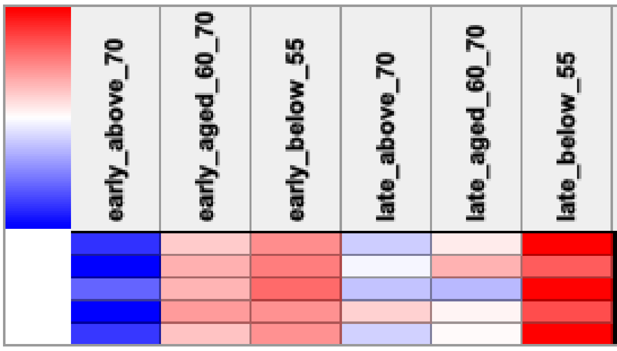

Probe ID	Description	Correlation
NAPA		0.98972
VWDE		0.95486
DNAJC12		0.94417
TMEM189		0.95815
ECH1		0.98912

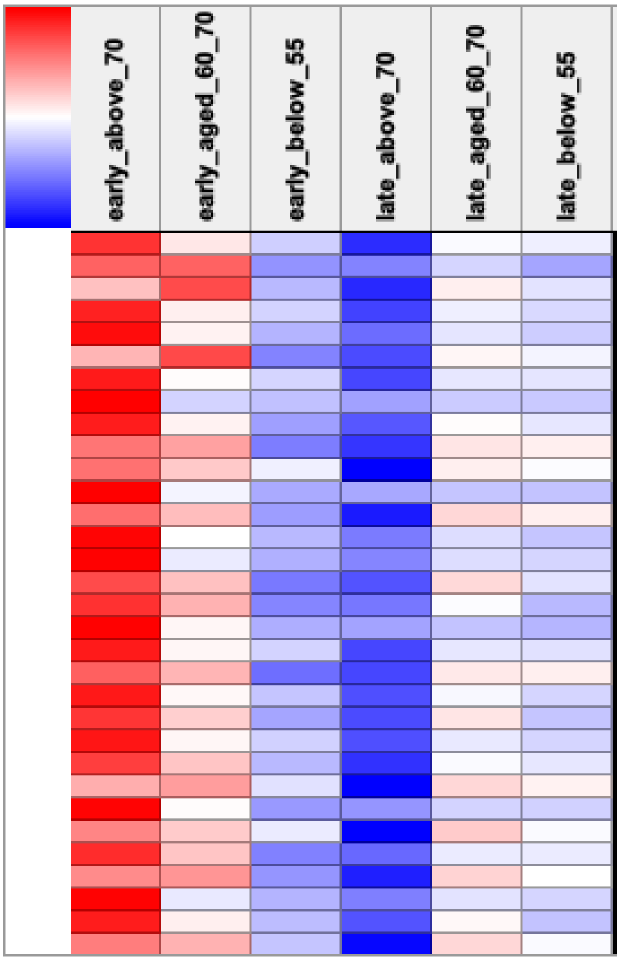

Probe ID	Description	Correlation
CABIN1		0.98486
CNN2		0.86977
LTBP4		0.78221
MEGF8		0.98191
PID1		0.97176
PRAM1		0.79198
STRADB		0.97129
SYT1		0.86343
MYO9B		0.98621
CHODL		0.93166
CTNS		0.91796
CYP2J2		0.88728
DISP2		0.94395
NCDN		0.95088
RPS6KA2		0.92911
TMEM86A		0.96889
PTP4A3		0.96548
ARHGAP1		0.89931
ATG9A		0.97560
BCL6		0.94468
CTC1		0.98140
CUL9		0.99005
GALNT10		0.97513
GOLGA3		0.99571
NSMCE1		0.83163
ORAI3		0.92875
PLEKHG5		0.88591
PNPLA2		0.97988
POU2F2		0.90816
TBC1D16		0.93586
ZDHHC14		0.98353
ZNF324		0.92569

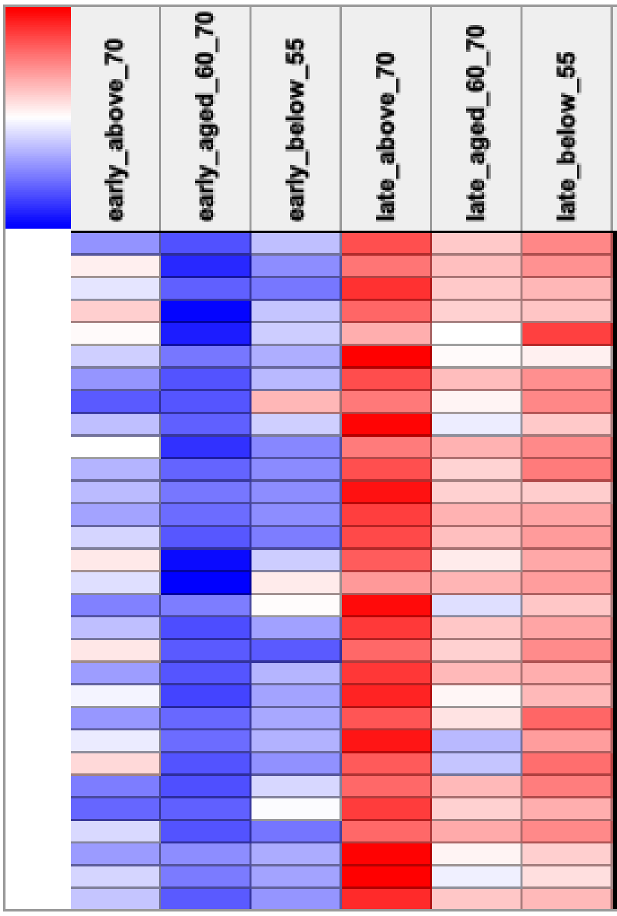

Probe ID	Description	Correlation
AP3M2		0.97715
AZIN1		0.93827
BRAF		0.96997
ECT2		0.89824
EFCAB11		0.85216
HILPDA		0.92439
ISCA1		0.97817
PAK1		0.78182
PRLR		0.95025
RAD23B		0.94103
ZWILCH		0.98001
MAD2L1		0.96148
TMEM70		0.97081
AGTPBP1		0.97982
BAG4		0.93688
DNAH11		0.85404
GAS2		0.87505
MAPK9		0.99617
PTPRR		0.91391
RASSF3		0.98143
SDCBP		0.97702
WASF1		0.96403
CMBL		0.91998
DAPK1		0.88220
GLO1		0.93914
MRPL50		0.91266
RRM1		0.95538
SLBP		0.93330
TPGS2		0.92805
ZNF273		0.98691

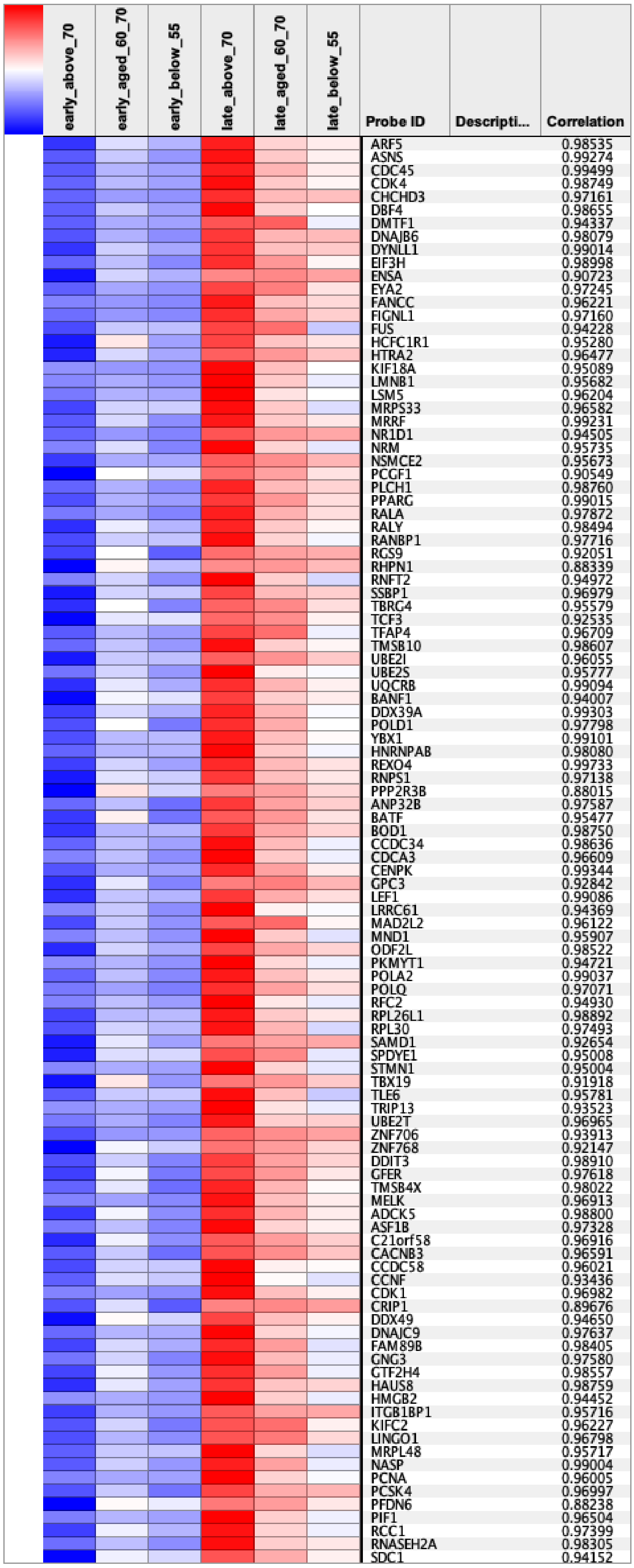

Probe ID	Descripti...	Correlation
ARF5		0.98535
ASNS		0.99274
CDC45		0.99499
CDK4		0.98749
CHCHD3		0.97161
DBF4		0.98655
DMTF1		0.94337
DNAJB6		0.98079
DYNLL1		0.99014
EIF3H		0.98998
ENSA		0.90723
EYA2		0.97245
FANCC		0.96221
FIGNL1		0.97160
FUS		0.94228
HCFC1R1		0.95280
HTRA2		0.96477
KIF18A		0.95089
LMNB1		0.95682
LSM5		0.96204
MRPS33		0.96582
MRRF		0.99231
NR1D1		0.94505
NRM		0.95735
NSMCE2		0.95673
PCGF1		0.90549
PLCH1		0.98760
PPARG		0.99015
RALA		0.97872
RALY		0.98494
RANBP1		0.97716
RGS9		0.92051
RHPN1		0.88339
RNFT2		0.94972
SSBP1		0.96979
TBRG4		0.95579
TCF3		0.92535
TFAP4		0.96709
TMSB10		0.98607
UBE2I		0.96055
UBE2S		0.95777
UQCRB		0.99094
BANF1		0.94007
DDX39A		0.99303
POLD1		0.97798
YBX1		0.99101
HNRNPAB		0.98080
REXO4		0.99733
RNPS1		0.97138
PPP2R3B		0.88015
ANP32B		0.97587
BATF		0.95477
BOD1		0.98750
CCDC34		0.98636
CDCA3		0.96609
CENPK		0.99344
GPC3		0.92842
LEF1		0.99086
LRRC61		0.94369
MAD2L2		0.96122
MND1		0.95907
ODF2L		0.98522
PKMYT1		0.94721
POLA2		0.99037
POLQ		0.97071
RFC2		0.94930
RPL26L1		0.98892
RPL30		0.97493
SAMD1		0.92654
SPDYE1		0.95008
STMN1		0.95004
TBX19		0.91918
TLE6		0.95781
TRIP13		0.93523
UBE2T		0.96965
ZNF706		0.93913
ZNF768		0.92147
DDIT3		0.98910
GFER		0.97618
TMSB4X		0.98022
MELK		0.96913
ADCK5		0.98800
ASF1B		0.97328
C21orf58		0.96916
CACNB3		0.96591
CCDC58		0.96021
CCNF		0.93436
CDK1		0.96982
CRIP1		0.89676
DDX49		0.94650
DNAJC9		0.97637
FAM89B		0.98405
GNG3		0.97580
GTF2H4		0.98557
HAUS8		0.98759
HMGB2		0.94452
ITGB1BP1		0.95716
KIFC2		0.96227
LINGO1		0.96798
MRPL48		0.95717
NASP		0.99004
PCNA		0.96005
PCSK4		0.96997
PFDN6		0.88238
PIF1		0.96504
RCC1		0.97399
RNASEH2A		0.98305
SDC1		0.94152

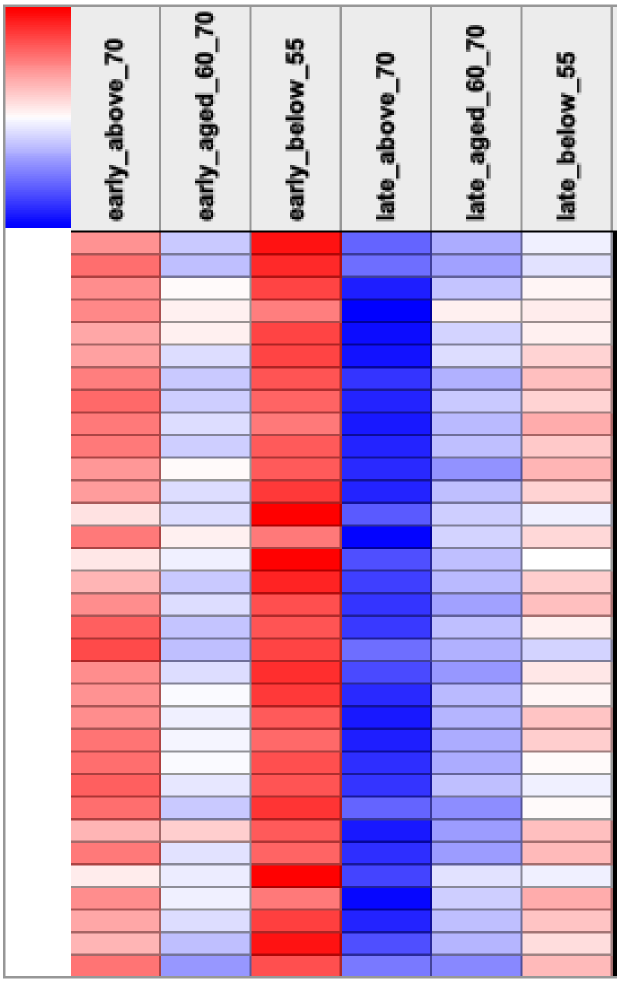

Probe ID	Description	Correlation
ATP8B1		0.96260
BNIP3L		0.95026
CRYM		0.98934
HMGXB3		0.92160
MCTP2		0.97854
NDFIP2		0.98716
PREP		0.98955
PRKACB		0.98291
SCP2		0.97032
SUSD1		0.98947
DPAGT1		0.97699
PDIA4		0.99712
ARSD		0.91292
CLN5		0.96217
CSGALNAC...		0.92570
CYP39A1		0.98161
NUDT12		0.98991
PGM3		0.98114
SPOCK1		0.92507
TMEM108		0.98917
TSC22D3		0.99392
HYOU1		0.99077
ADI1		0.98446
ADPGK		0.98780
B3GALNT2		0.97862
C1orf116		0.96563
CIB1		0.94605
ERC2		0.98111
GFOD2		0.90889
GLOD4		0.96282
HSP90B1		0.99261
LYRM2		0.97357
SPON2		0.92574

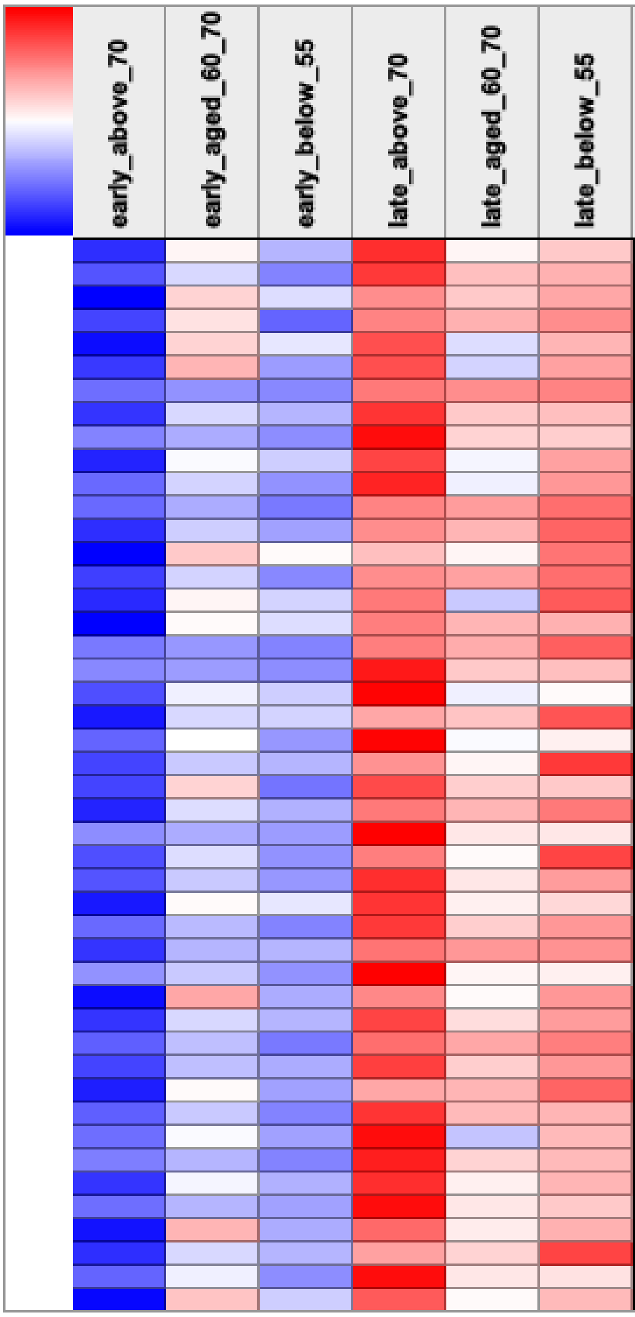

Probe ID	Descripti...	Correlation
CCDC114		0.97186
CMTM7		0.98286
ENO3		0.90644
GATA6		0.93731
ID2		0.90756
KLF5		0.91528
RAI14		0.89728
SRI		0.98604
CKS1B		0.91880
RSPH9		0.97336
AKR1C2		0.96688
ARHGAP15		0.91474
CHAC2		0.95133
CHL1		0.80779
LAMB1		0.94092
LRRC43		0.90204
LRRC4C		0.93434
NVL		0.90127
PACSIN1		0.91876
RHEB		0.89533
RNF130		0.90863
SLC25A39		0.91486
SPAG4		0.91357
ZNF787		0.94831
CNIH4		0.97185
CKS2		0.88419
AKR1C1		0.94178
ARPC5L		0.98801
ARRDC1		0.93603
CCNE1		0.97202
CDC26		0.95087
CHEK2		0.87225
FASTK		0.89685
IQCD		0.99680
IRF2BP2		0.94857
LRR1		0.98945
PLEKHH3		0.92272
RAC1		0.97267
SEMA3B		0.91237
SKA2		0.94355
SLC2A8		0.98719
TMEM203		0.94131
TMUB1		0.93119
TOMM40L		0.91455
URM1		0.94371
ZP3		0.92603

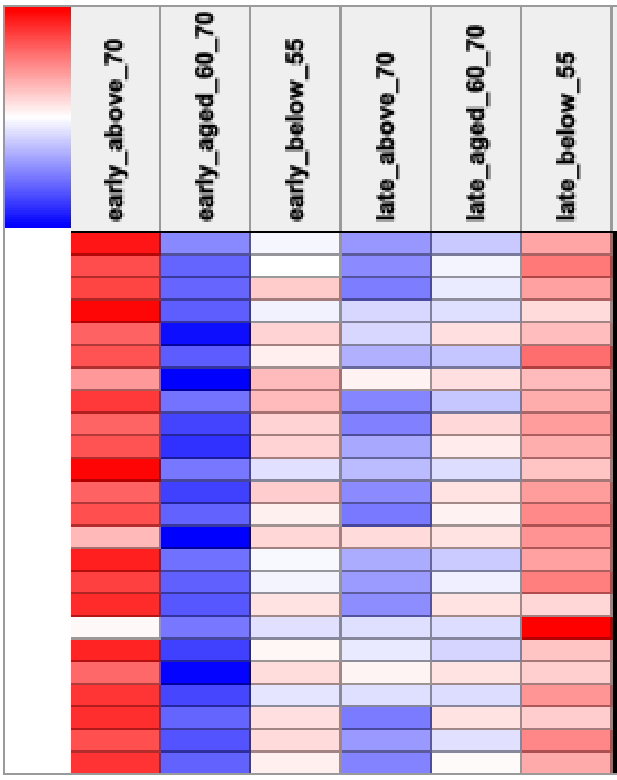

Probe ID	Description	Correlation
APC		0.94381
FER		0.97836
GALNT3		0.97340
GPN3		0.93562
GTF2H3		0.95334
RAB3GAP1		0.97007
SHBG		0.84863
ZRANB3		0.95050
TMED4		0.96661
BMPR1A		0.98714
DNAJC25		0.93645
EPC2		0.97588
FRRS1		0.97411
GGH		0.80278
MMS22L		0.96660
NPEPPS		0.98348
LCOR		0.95950
TOB1		0.63086
ACAA2		0.95644
C9orf64		0.89736
FBXL13		0.96761
SLC35E3		0.95455
SNX4		0.98910
SPTY2D1		0.98084

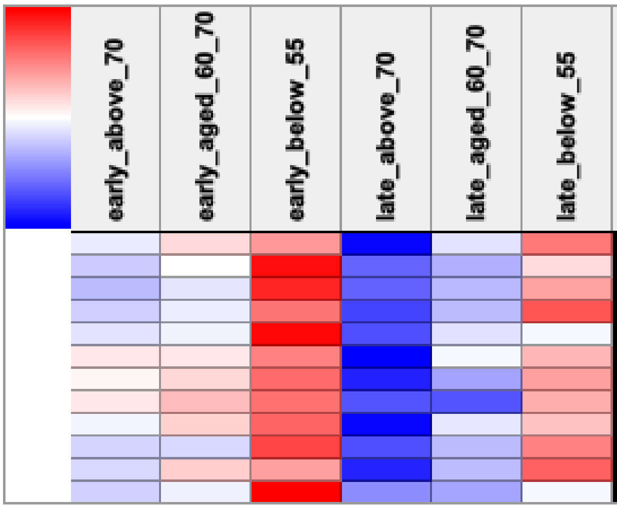

Probe ID	Description	Correlation
ITGB8		0.93284
SORCS1		0.94862
TDP2		0.96198
KBTBD6		0.94552
ARSJ		0.90903
ATRNL1		0.92746
GPM6B		0.97925
HCN4		0.89902
SH3TC1		0.96406
SMARCA1		0.97004
SUSD4		0.92518
ACYP2		0.86040

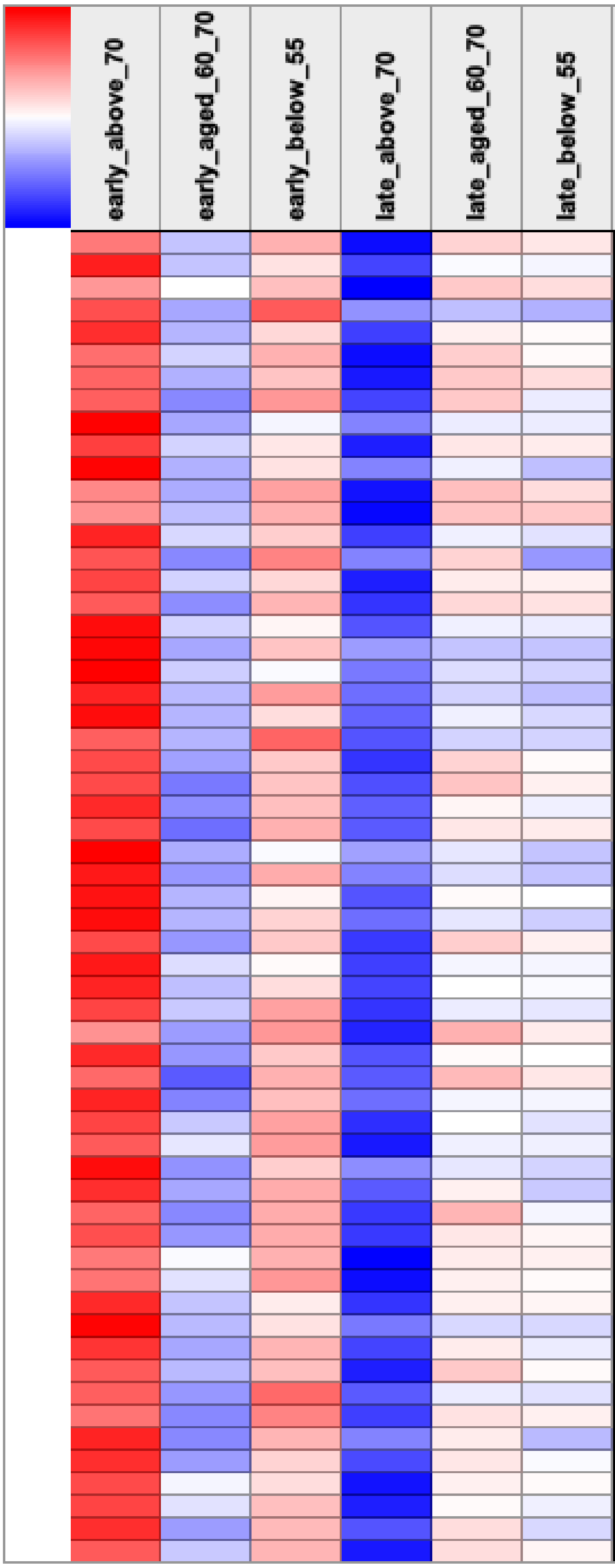

Probe ID	Descripti...	Correlation
AHI1		0.94470
FAM120B		0.98302
FBXO32		0.86089
GALNT7		0.84892
KAT6B		0.99180
LIPT1		0.95322
METTL8		0.95808
N4BP2		0.96695
POLR3B		0.91746
PTPRJ		0.96911
SIPA1L3		0.93607
TESK2		0.92503
TRIM2		0.90138
VCL		0.98184
VPS54		0.89119
WDR35		0.97492
ZBTB1		0.97213
ZKSCAN2		0.95558
C6orf52		0.89742
ZNF778		0.91244
ANTXR2		0.94888
HLCS		0.96736
MAML3		0.92333
ABI2		0.98817
BICD1		0.96300
CGRRF1		0.98838
DSP		0.96609
EDEM3		0.87908
ILDR1		0.94465
ITGA9		0.97002
KIF13A		0.95893
MPDZ		0.97980
PHACTR2		0.95407
PIK3C3		0.98693
PPP1R13B		0.98307
PRSS23		0.91088
RAB11FIP2		0.99101
RASSF8		0.92044
RNF13		0.97166
SPECC1L		0.98538
TTC17		0.95772
ZNF331		0.93958
COLEC12		0.98335
SLC36A4		0.95709
BTRC		0.98955
DNAJC28		0.91706
ELK4		0.94426
GIGYF2		0.97893
HSH2D		0.94440
LEO1		0.99909
PGBD4		0.97606
PIGN		0.93801
RAVER2		0.95003
ROR1		0.94953
SGMS1		0.99222
STK32C		0.94976
WIPI1		0.97755
ZSCAN30		0.99019
ZSWIM5		0.97417

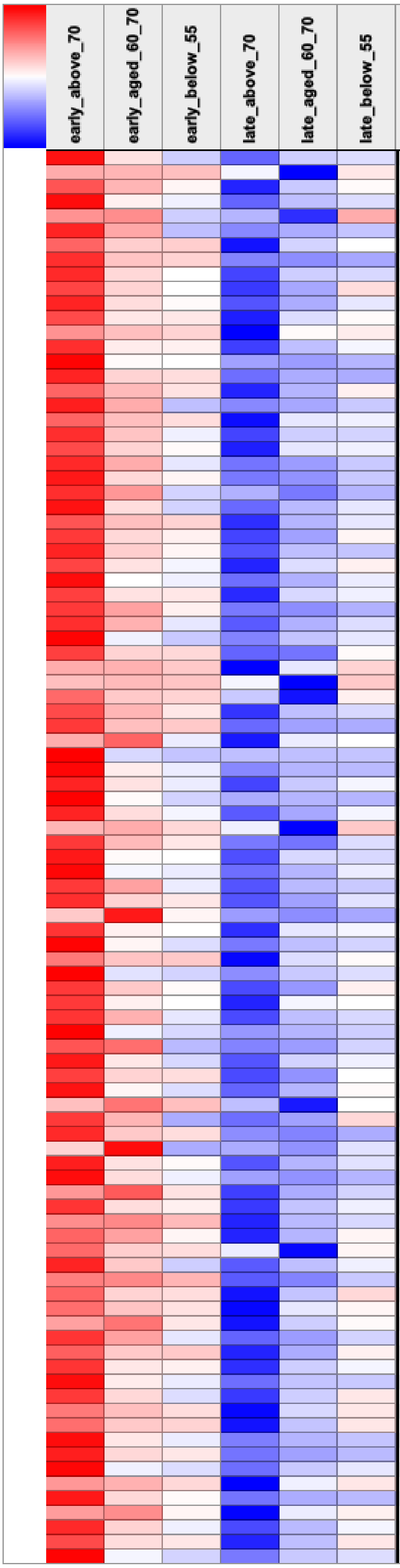

Probe ID	Descripti...	Correlation
ARHGAP26		0.95913
BCAN		0.54367
CA11		0.97300
CELSR1		0.97050
CFTR		0.70504
CHI3L2		0.93900
CREB3		0.93782
CREB3L1		0.94091
CYP20A1		0.98480
DHRS7B		0.97752
DUSP1		0.99498
EML2		0.95197
ETV6		0.83719
HM13		0.98225
ITM2C		0.91331
KCNK6		0.96509
LHPP		0.96397
NR4A3		0.93953
PDGFC		0.93283
PER2		0.98489
PIEZO1		0.95704
PITPNC1		0.97807
PLA2G15		0.97423
RHOB		0.89130
RTN2		0.97165
SIL1		0.97602
SUMF2		0.98999
TRPM4		0.98678
VPS53		0.95552
SEC61A1		0.96312
ABCA8		0.96708
ACP5		0.95968
BCAR1		0.99095
DHRS7		0.91282
ERP29		0.96774
FBN3		0.81701
FST		0.48135
GLRB		0.75200
GMPPA		0.98541
GSS		0.95809
HDC		0.81489
INPP4B		0.81062
NPR3		0.94322
PPL		0.98066
RHOBTB2		0.88150
RRBP1		0.99079
SCGN		0.53172
SH3BGRL		0.96984
SIDT2		0.96171
SLC26A4		0.95159
SLC4A11		0.97972
TCF25		0.99435
TF		0.64088
TTC28		0.95196
USP5		0.94598
WLS		0.91025
RNF150		0.89539
TXNRD2		0.99115
IL6R		0.93456
ABHD1		0.98746
ALDH2		0.90380
ANKRD24		0.90129
ATG13		0.96474
ATP6V0D1		0.98711
BLVRB		0.96475
C2CD4A		0.64306
CDC42EP1		0.92432
CDC42EP4		0.93708
CEBPD		0.56781
CHPF		0.99039
CLN3		0.92872
CYB5R2		0.87411
EFCAB6		0.98796
EMP2		0.89589
GUSB		0.96282
IGSF5		0.68813
LIMK2		0.97669
LRRC29		0.90553
MAN1B1		0.93292
MAPKAPK3		0.92466
MED16		0.85719
NAGPA		0.98257
NUCB1		0.96006
PBXIP1		0.97502
PGPEP1		0.96315
PIP5K1C		0.96743
PLCD1		0.91383
PLD3		0.93212
SLC9B2		0.95790
STAT6		0.97194
SYTL3		0.93554
TBC1D17		0.84846
TNK1		0.97275
TRAPPC12		0.85120
VAMP2		0.99278
VPS33B		0.96597
WDR45		0.92676

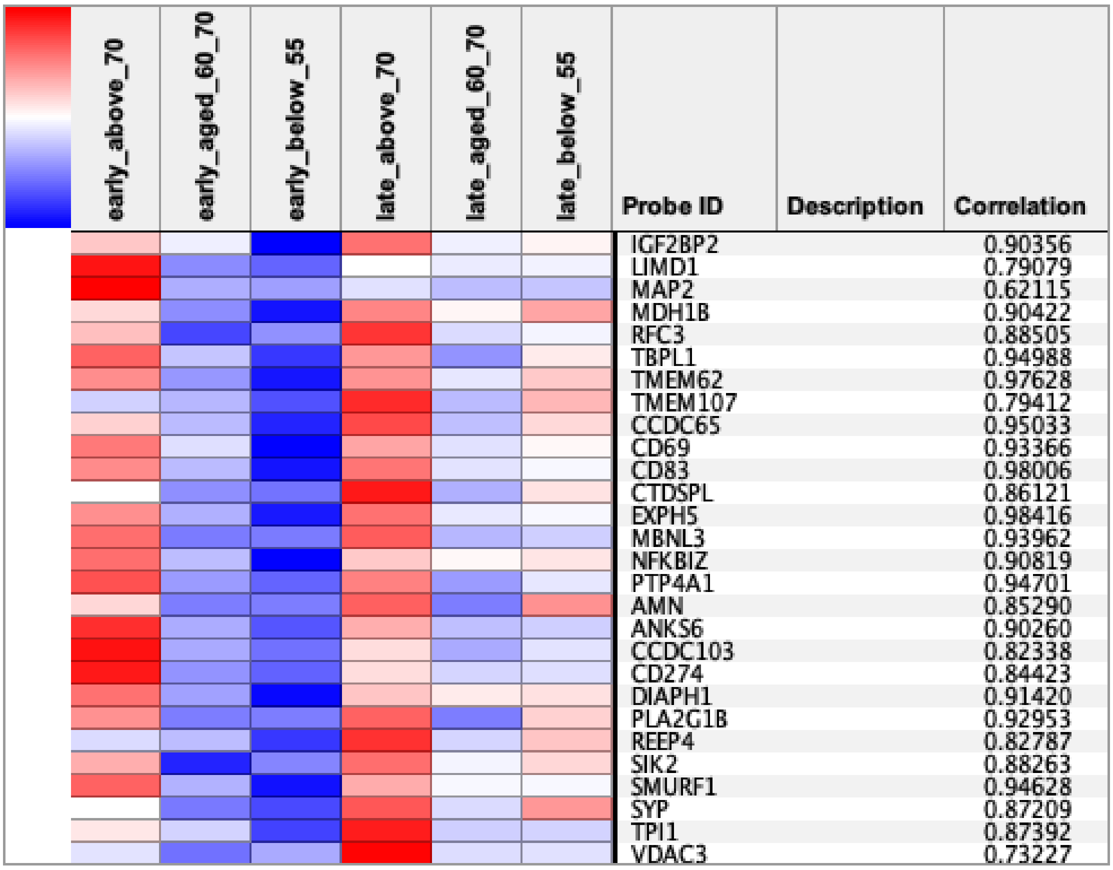
early_above_70
early_aged_60_70
early_below_55
late_above_70
late_aged_60_70
late_below_55
Probe ID
Description
Correlation
IGF2BP2 0.90356
LIMD1 0.79079
MAP2 0.62115
MDH1B 0.90422
RFC3 0.88505
TBPL1 0.94988
TMEM62 0.97628
TMEM107 0.79412
CCDC65 0.95033
CD69 0.93366
CD83 0.98006
CTDSPL 0.86121
EXPH5 0.98416
MBNL3 0.93962
NFKBIZ 0.90819
PTP4A1 0.94701
AMN 0.85290
ANKS6 0.90260
CCDC103 0.82338
CD274 0.84423
DIAPH1 0.91420
PLA2G1B 0.92953
REEP4 0.82787
SIK2 0.88263
SMURF1 0.94628
SYP 0.87209
TPI1 0.87392
VDAC3 0.73227

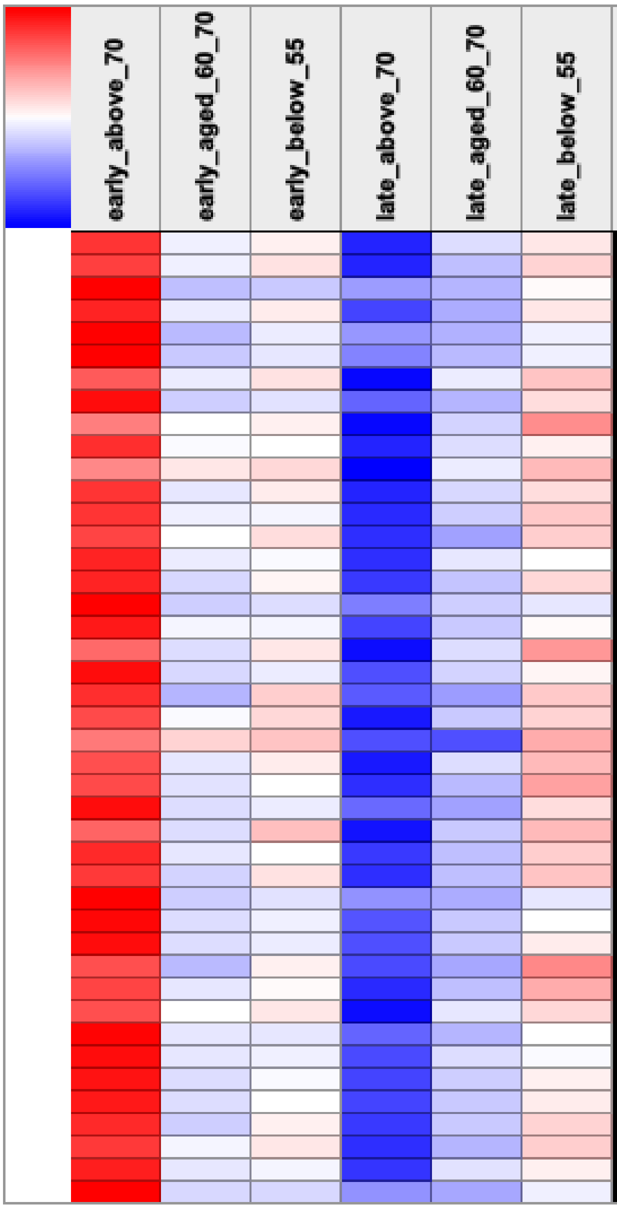

Probe ID	Description	Correlation
AAK1		0.98850
BCAS3		0.99033
CDC14B		0.90560
CELF2		0.99420
CREBL2		0.91708
ERBB3		0.94092
GTPBP1		0.95988
GYS1		0.97810
PGM1		0.90804
RIPK1		0.98625
SATB2		0.88688
SCAMP2		0.99236
SPHK2		0.99439
SREBF1		0.97930
SSPN		0.98277
SUMF1		0.99914
SLC11A2		0.93494
MADD		0.98900
ABCD3		0.93828
AP1S3		0.98498
CAT		0.96332
EVI5L		0.97681
GNAT2		0.83379
KAT2B		0.97445
RWDD2B		0.97455
SIAE		0.97772
SLC16A2		0.94619
SORT1		0.99834
TBC1D8B		0.98970
CNTNAP2		0.91685
ALG1		0.98207
APOLD1		0.98977
FAM217B		0.93854
GRAMD1C		0.98101
ICMT		0.96715
LDHD		0.97323
MPL		0.98059
PSAP		0.99364
SSR3		0.99589
TCTN1		0.99594
TMC4		0.99091
TRIM68		0.98795
ZNF652		0.92284

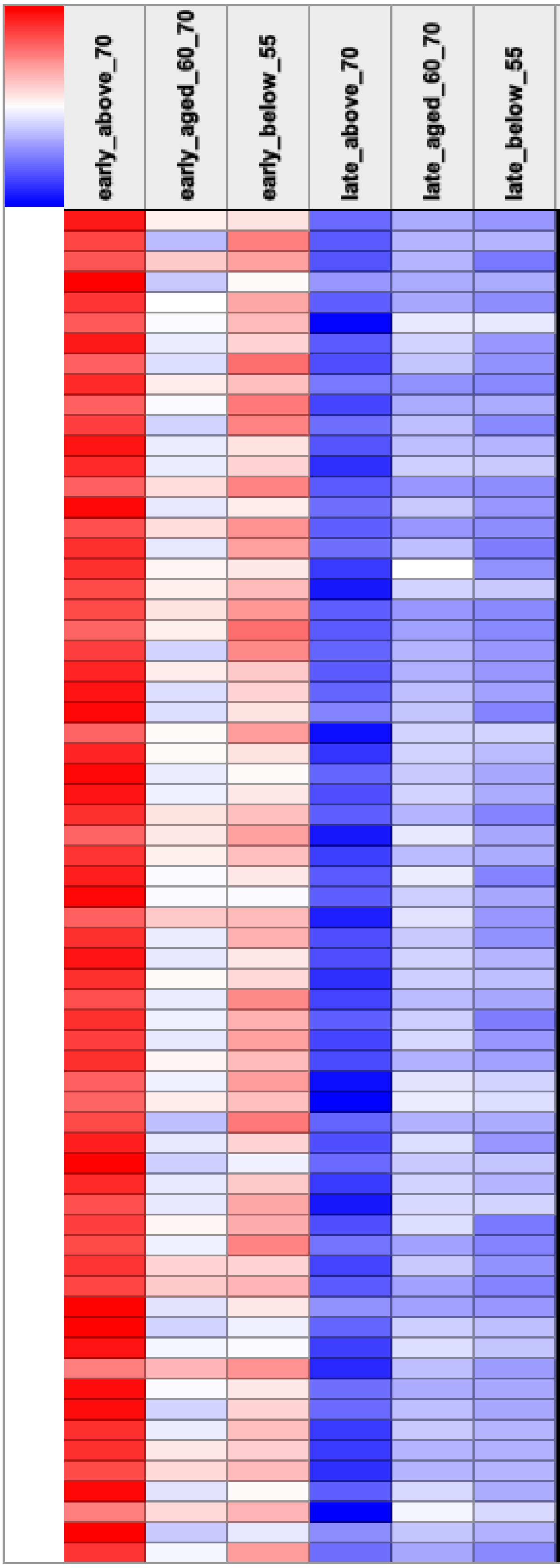

Probe ID	Descripti...	Correlation
ACAD8		0.97927
ACAT2		0.94907
AMT		0.96035
DUSP16		0.91856
ELP3		0.98800
FSTL4		0.93276
FZD1		0.99148
FZD3		0.94994
GMDS		0.96898
INTS9		0.95597
MANSC1		0.96144
MBTPS1		0.98650
MTFMT		0.98140
PEMT		0.94773
PTK2B		0.97334
PXN		0.95945
QSER1		0.98095
RAP1GAP2		0.96514
SIDT1		0.97284
SLC1A5		0.96726
SMPDL3B		0.94029
TIFA		0.96986
TSPAN18		0.99247
UBXN8		0.98528
ARHGEF37		0.96252
CLSTN1		0.95131
VPS39		0.98460
PRKCB		0.96681
CHD3		0.98392
MYBPC1		0.98330
ABCA10		0.95698
BCAS1		0.99678
BLNK		0.97080
HPN		0.96496
HS3ST3A1		0.95213
LRCH2		0.99666
MLEC		0.98064
NISCH		0.98550
RAPGEF4		0.97796
RPS6KC1		0.98791
SARDH		0.98892
SH3BP4		0.99696
SPCS3		0.94549
STARD8		0.92319
STYK1		0.94500
SUN2		0.98859
SYTL4		0.93227
WTAP		0.99274
YIPF3		0.96292
ANXA9		0.98179
C2		0.95599
CES2		0.98386
CNPY3		0.96772
DLGAP2		0.94368
DPH1		0.93994
FAM111A		0.95883
FAM174B		0.92015
FUT10		0.97695
LRRC8E		0.97973
NWD1		0.99552
PRSS8		0.99082
PSTK		0.97974
TEP1		0.96487
TIGD6		0.88686
TRAK1		0.90281
TTC38		0.98015

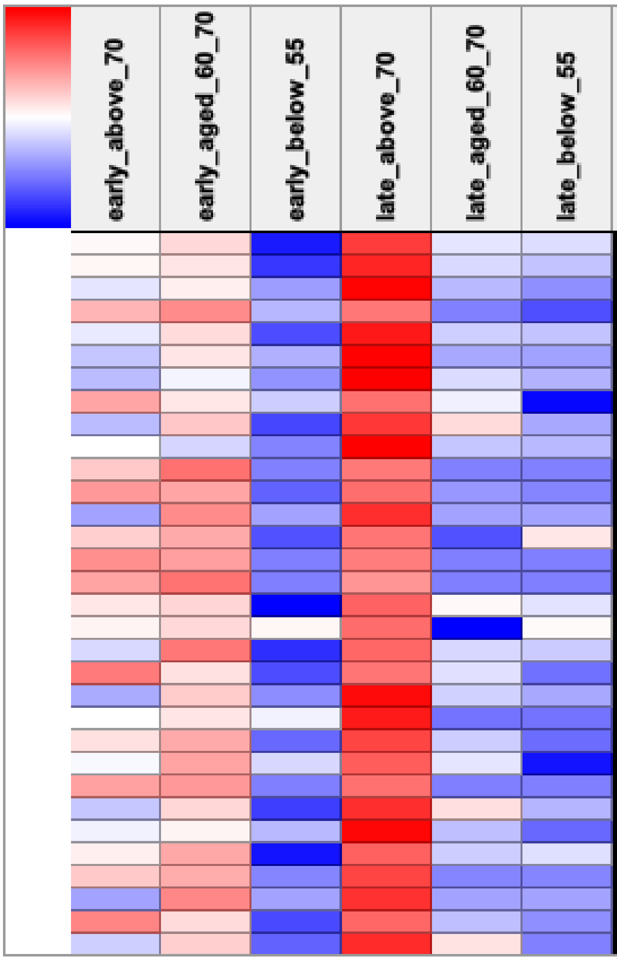

Probe ID	Description	Correlation
EGLN3		0.91174
KAZALD1		0.95008
MEPCE		0.94981
MPST		0.87603
SCNN1A		0.95722
SLC12A9		0.92104
MOSPD3		0.88489
TNFSF9		0.76559
CCDC85B		0.87289
DUSP2		0.89745
JSRP1		0.91563
JUND		0.92884
ANXA13		0.90300
CAPN6		0.80321
CYP11A1		0.89361
HTR2A		0.86461
IL1RN		0.83975
NECAB2		0.57786
PGF		0.89649
TCIRG1		0.86456
TRIP6		0.91923
TTR		0.85975
TMEM238		0.98407
DDX11		0.83035
EPHA8		0.91931
FKBPL		0.87684
GNB2		0.92578
KLF16		0.90754
LRFN2		0.97237
PCDHA4		0.90154
PDZK1IP1		0.90506
RAD9A		0.90735

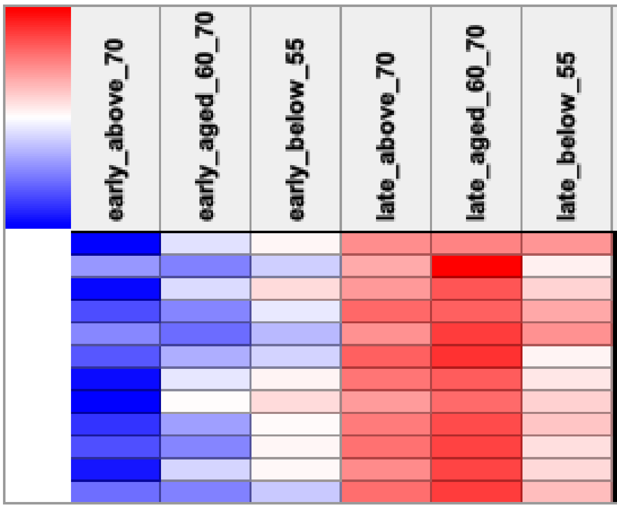

Probe ID	Description	Correlation
AKIRIN2		0.94036
FAM189A1		0.89786
HNRNPD		0.96363
NUP54		0.97817
ZC2HC1A		0.91596
HNRNPH1		0.97348
ABT1		0.96591
ARL16		0.92413
GATA2		0.99742
TMEM52		0.98366
ZBTB12		0.98411
ZNF7		0.96500

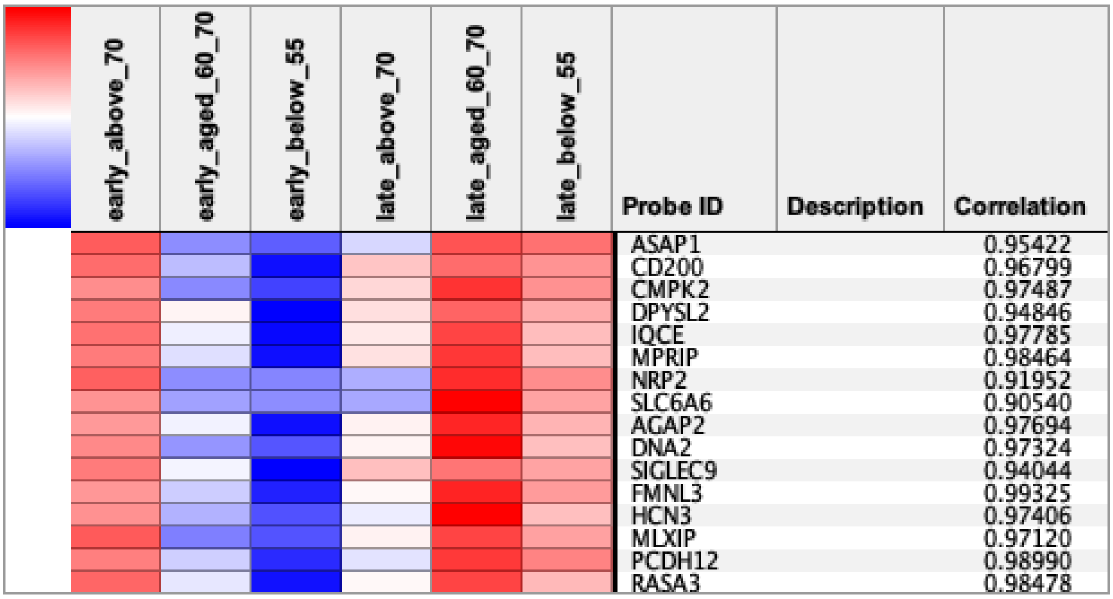
early_above_70
early_aged_60_70
early_below_55
late_above_70
late_aged_60_70
late_below_55
Probe ID
Description
Correlation
ASAP1 0.95422
CD200 0.96799
CMPK2 0.97487
DPYSL2 0.94846
IQCE 0.97785
MPRIP 0.98464
NRP2 0.91952
SLC6A6 0.90540
AGAP2 0.97694
DNA2 0.97324
SIGLEC9 0.94044
FMNL3 0.99325
HCN3 0.97406
MLXIP 0.97120
PCDH12 0.98990
RASA3 0.98478

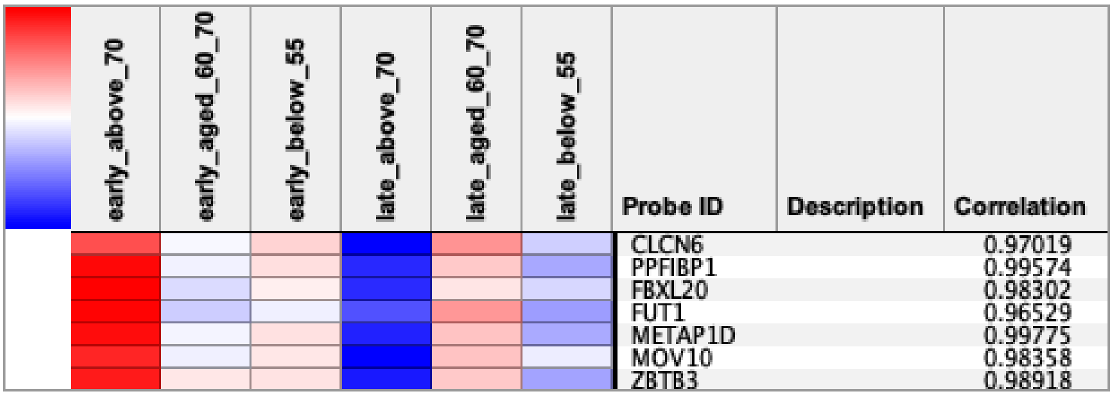
early_above_70
early_aged_60_70
early_below_55
late_above_70
late_aged_60_70
late_below_55
Probe ID
Description
Correlation
CLCN6 0.97019
PPFIBP1 0.99574
FBXL20 0.98302
FUT1 0.96529
METAP1D 0.99775
MOV10 0.98358
ZBTB3 0.98918

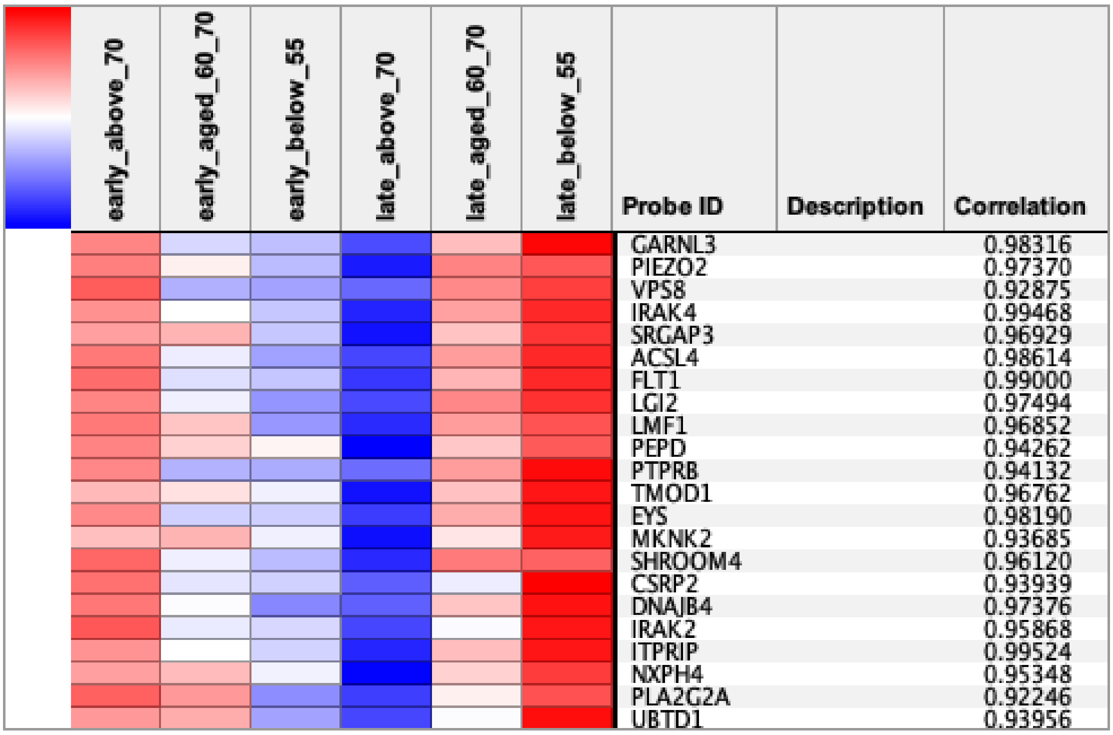
early_above_70
early_aged_60_70
early_below_55
late_above_70
late_aged_60_70
late_below_55
Probe ID
Description
Correlation
GARNL3 0.98316
PIEZO2 0.97370
VPS8 0.92875
IRAK4 0.99468
SRGAP3 0.96929
ACSL4 0.98614
FLT1 0.99000
LGI2 0.97494
LMF1 0.96852
PEPD 0.94262
PTPRB 0.94132
TMOD1 0.96762
EYS 0.98190
MKNK2 0.93685
SHROOM4 0.96120
CSRP2 0.93939
DNAJB4 0.97376
IRAK2 0.95868
ITPRIP 0.99524
NXPH4 0.95348
PLA2G2A 0.92246
UBTD1 0.93956

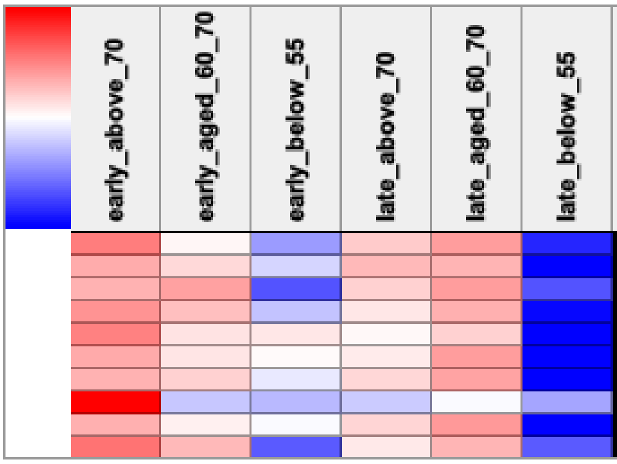

Probe ID	Description	Correlation
ATP8B4		0.98056
DHRS9		0.97392
EPO		0.87520
ICAM5		0.99276
TRPC6		0.93754
ZNF831		0.95492
BTBD19		0.97111
PITPNM2		0.64426
CD3G		0.95686
PXT1		0.90634

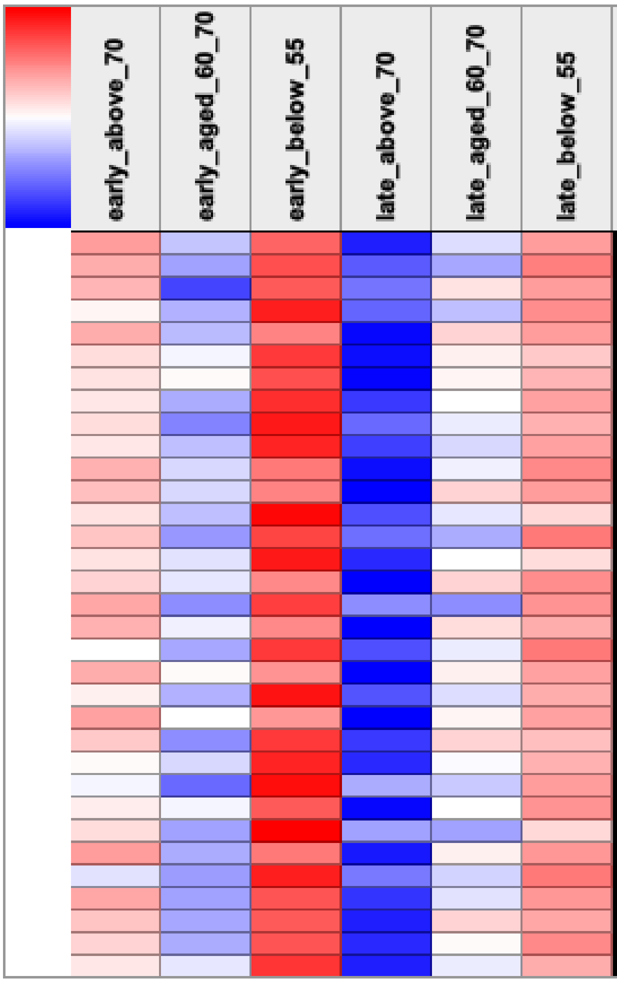

Probe ID	Description	Correlation
ALDH9A1		0.97850
CD164		0.94734
HAT1		0.89490
IDI1		0.95753
LRP11		0.94714
MYO1E		0.95936
OSBPL9		0.95358
PIBF1		0.99269
PPM1H		0.95818
STAMBP		0.98843
TMEM87A		0.96770
ATP1A1		0.94374
ACSL3		0.95991
CCNC		0.93872
CLGN		0.95973
CLIP4		0.93184
HGFAC		0.88334
IFNGR1		0.92347
KIF16B		0.97285
MBNL2		0.90989
MTHFS		0.97388
NEBL		0.90911
PIK3R1		0.96395
SLC46A3		0.97944
SMAD9		0.85250
PTER		0.96143
CCK		0.85236
ERAP1		0.96437
GLB1L		0.92170
LMBRD1		0.98726
MXRA7		0.97755
PLCB1		0.99168
SYNJ2BP		0.98364

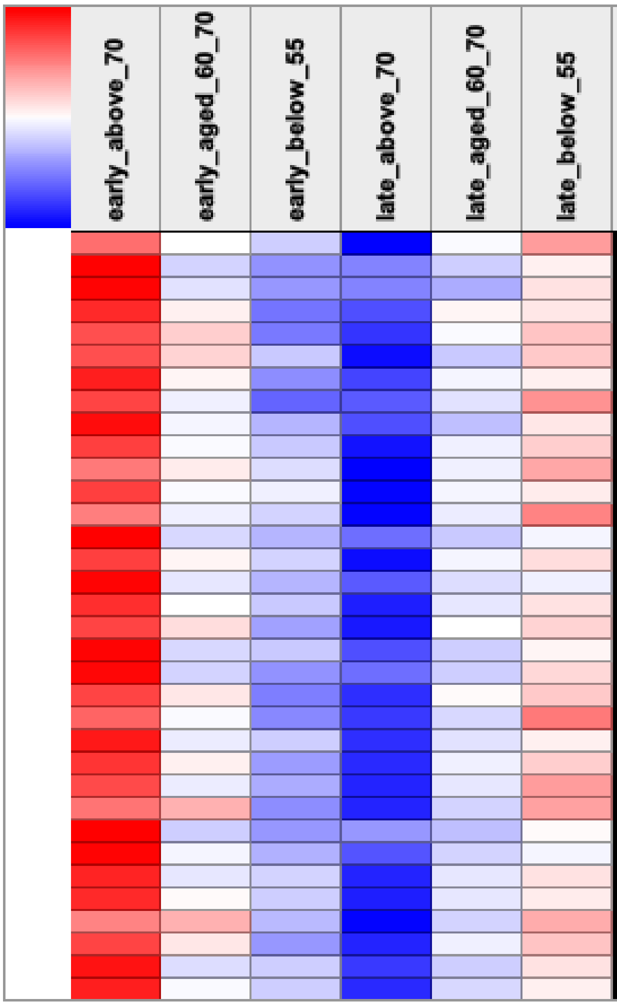

Probe ID	Description	Correlation
COL4A4		0.94176
DISP1		0.93536
DNAH9		0.93137
DTX4		0.96611
GFOD1		0.96240
NFIC		0.97237
PIK3IP1		0.98424
PIK3R3		0.93949
SOD2		0.98101
SYNE2		0.98837
TLE3		0.92922
WDR37		0.95728
TMEM143		0.91668
TOM1L2		0.94208
ADAMTS5		0.98202
ARHGEF5		0.95901
ATP6V0A1		0.99206
CERS4		0.98180
DHDDS		0.96819
IQSEC2		0.95884
PNPLA6		0.97822
POU2F3		0.93294
WDTC1		0.98510
NEGR1		0.99544
SLC20A2		0.97911
ABCA7		0.92181
C6orf132		0.90297
CAMTA2		0.97149
CCNDBP1		0.98543
GBA		0.98807
PTPRU		0.91298
SLC6A17		0.98920
SPRYD3		0.98165
THAP8		0.98767

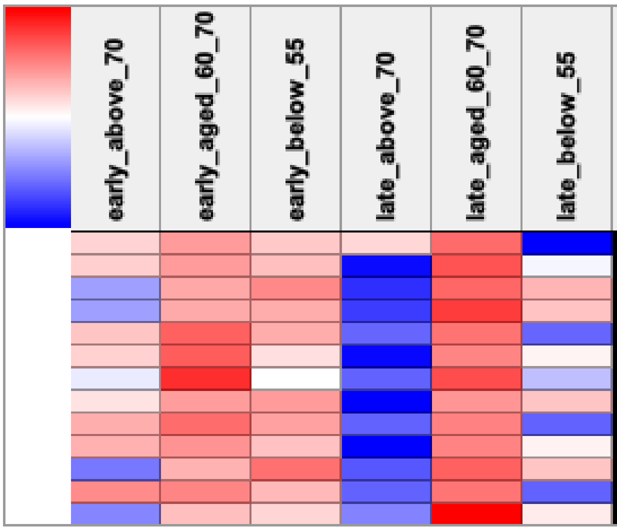

Probe ID	Description	Correlation
KCNN1		0.45634
KLHL29		0.96852
MAPK10		0.85689
STAMBPL1		0.87958
UCN2		0.90252
ADAMTS3		0.93379
COL9A1		0.92430
EPX		0.88979
GATA5		0.87782
PGBD1		0.91753
TMEM204		0.80602
PRCD		0.84906
SLC9A5		0.80194

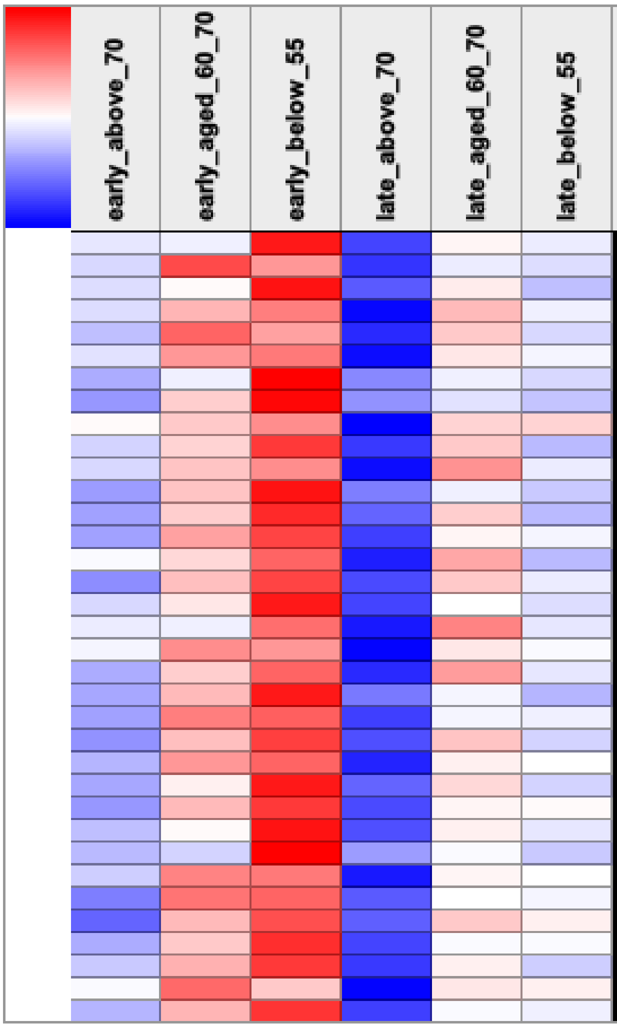

Probe ID	Description	Correlation
BBS2		0.93573
DNAJC17		0.87336
MGLL		0.94290
MTUS2		0.95107
MZF1		0.89916
RRNAD1		0.95668
EML6		0.88632
ASB2		0.90127
CATSPERB		0.88263
CBLC		0.97677
CELF6		0.91731
CIB2		0.93859
CRTAC1		0.96679
EBF4		0.98798
EDNRA		0.93040
MAPK13		0.98003
PDK4		0.96809
PDZRN3		0.88099
PLA2G5		0.90828
RBMS3		0.96118
RGS2		0.94709
RGS22		0.96294
RIMBP2		0.98027
RNF165		0.97813
SMPD3		0.96035
TFEB		0.98239
TMTC4		0.96121
ZNF614		0.82998
C2CD4D		0.95529
CLDND2		0.92853
KLK11		0.93382
MCC		0.98527
PLA2G6		0.99377
SHC2		0.81945
TMEM79		0.99053

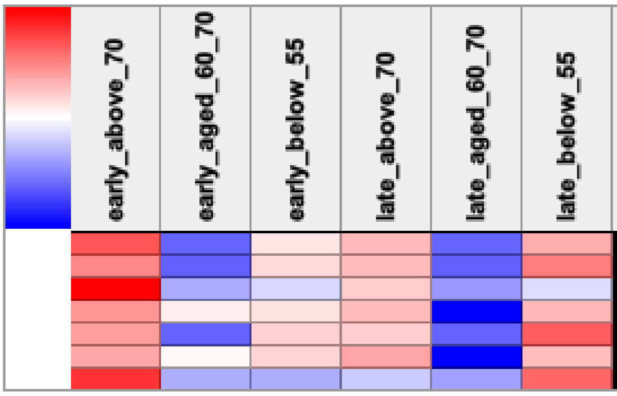

Probe ID	Description	Correlation
NR6A1		0.97070
IGSF10		0.95083
NEK6		0.78891
OPN3		0.86631
TMEM156		0.92097
LIPG		0.85214
PRR15L		0.80954

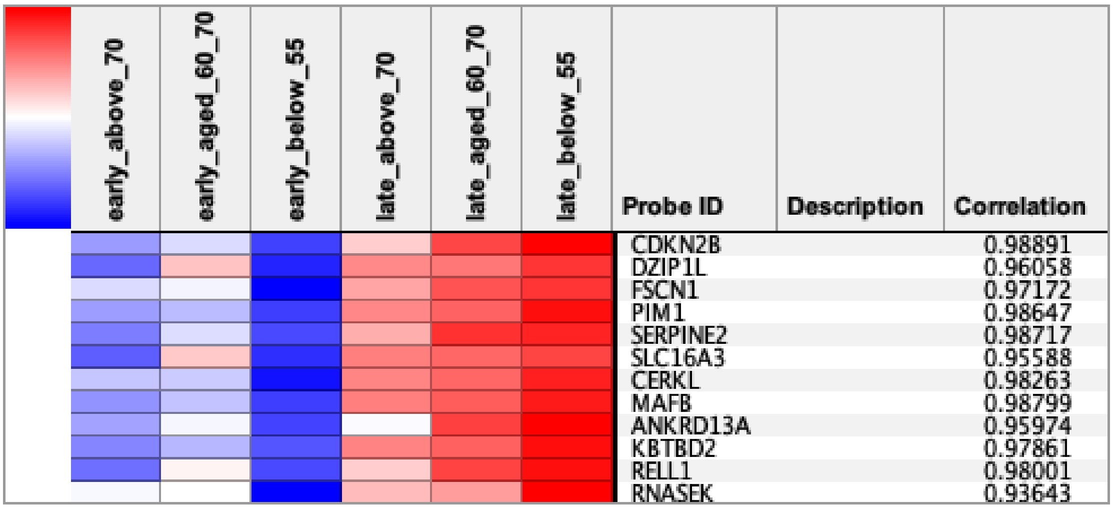
early_above_70
early_aged_60_70
early_below_55
late_above_70
late_aged_60_70
late_below_55
Probe ID
Description
Correlation
CDKN2B 0.98891
DZIP1L 0.96058
FSCN1 0.97172
PIM1 0.98647
SERPINE2 0.98717
SLC16A3 0.95588
CERKL 0.98263
MAFB 0.98799
ANKRD13A 0.95974
KBTBD2 0.97861
RELL1 0.98001
RNASEK 0.93643

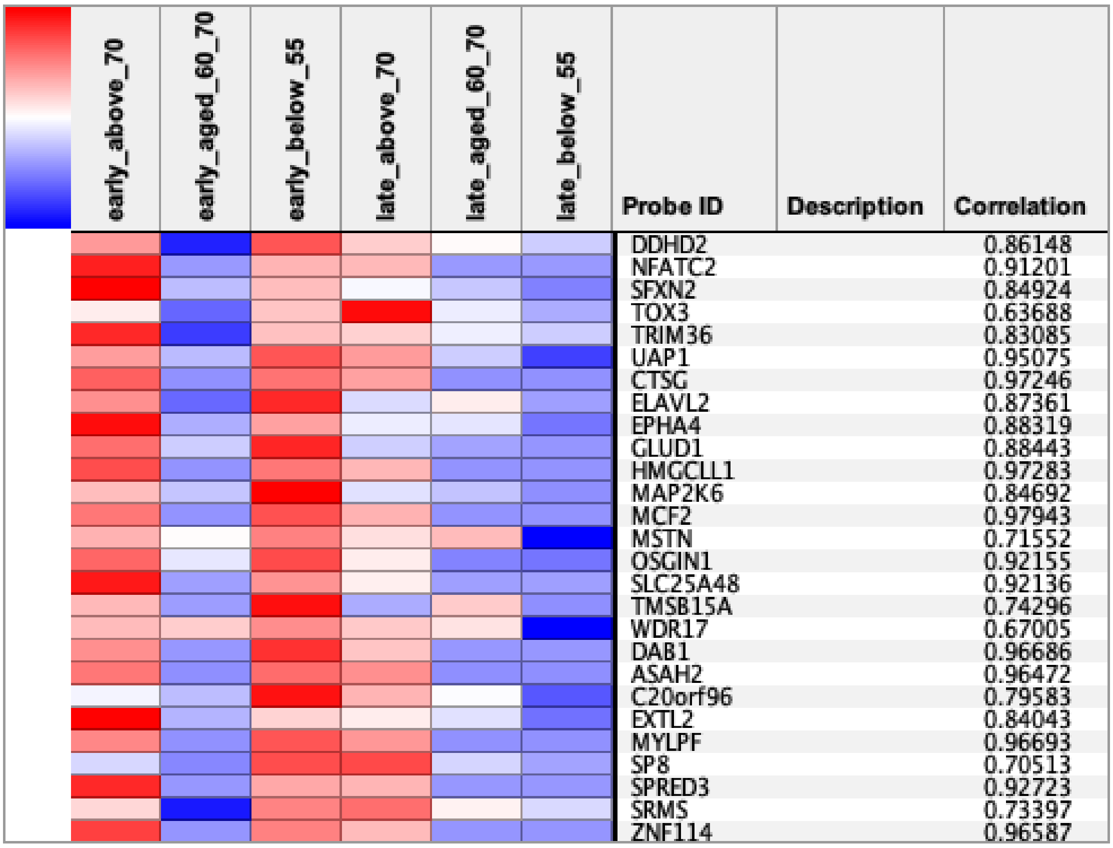
early_above_70
early_aged_60_70
early_below_55
late_above_70
late_aged_60_70
late_below_55
Probe ID
Description
Correlation
DDHD2 0.86148
NFATC2 0.91201
SFXN2 0.84924
TOX3 0.63688
TRIM36 0.83085
UAP1 0.95075
CTSG 0.97246
ELAVL2 0.87361
EPHA4 0.88319
GLUD1 0.88443
HMGCLL1 0.97283
MAP2K6 0.84692
MCF2 0.97943
MSTN 0.71552
OSGIN1 0.92155
SLC25A48 0.92136
TMSB15A 0.74296
WDR17 0.67005
DAB1 0.96686
ASAH2 0.96472
C20orf96 0.79583
EXTL2 0.84043
MYLPF 0.96693
SP8 0.70513
SPRED3 0.92723
SRMS 0.73397
ZNF114 0.96587

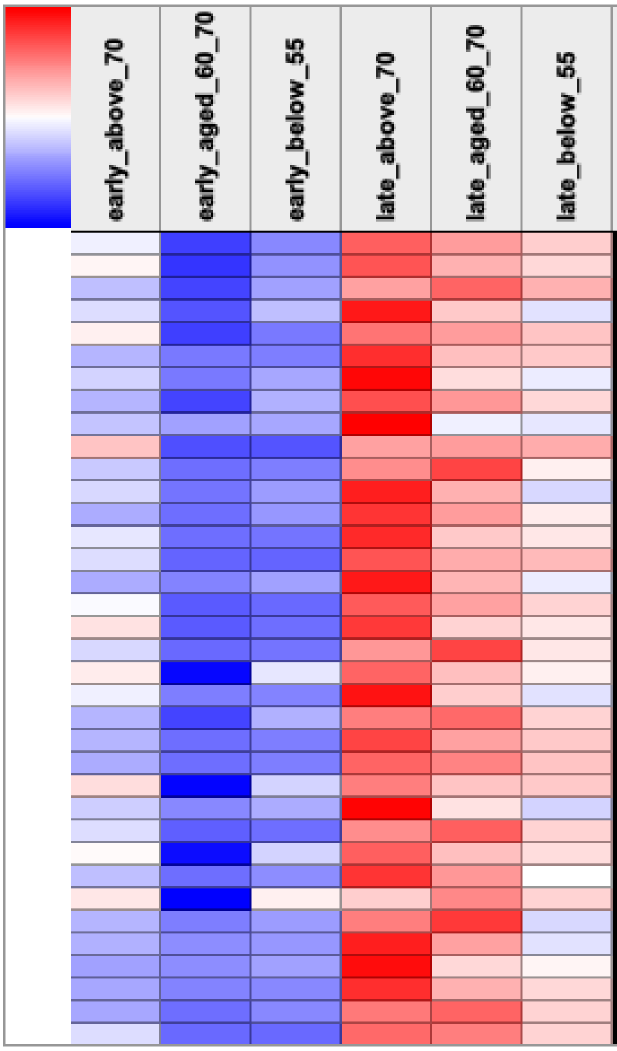

Probe ID	Description	Correlation
CEP78		0.98567
G3BP1		0.97604
HAUS6		0.91753
IGF2BP3		0.95038
KIF20B		0.96002
PLK4		0.97006
POLE2		0.93516
SNX10		0.98465
STARD3NL		0.84473
TTC26		0.86519
ZFAND1		0.92973
CDCA7L		0.96227
ARHGAP39		0.98199
BRCA2		0.97433
COL11A1		0.97690
DNMT3A		0.94983
ESRP1		0.98117
FANCD2		0.94720
IFIT2		0.91588
KLB		0.89772
MCM2		0.94180
MNX1		0.95155
NCAPG2		0.98479
OTUD6B		0.97008
TFR2		0.87447
ZNF618		0.90240
MYBL1		0.94410
SLC34A3		0.92402
C18orf54		0.98747
DCST1		0.73800
EME2		0.89478
FANCG		0.95175
GPR137C		0.92533
MRPL15		0.96904
MTBP		0.94848
RAD54B		0.97720

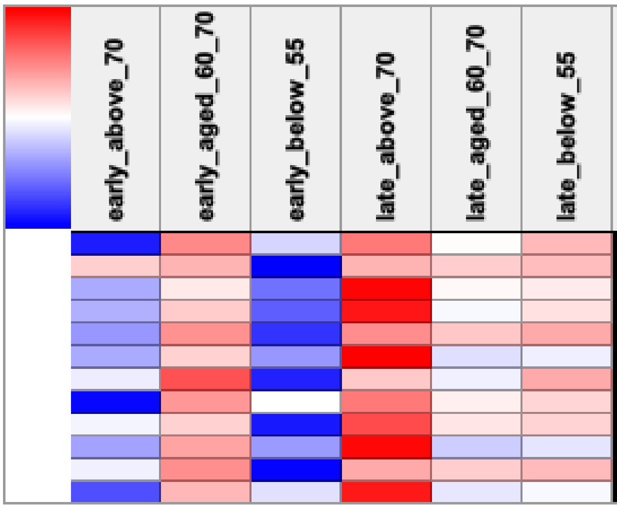

Probe ID	Description	Correlation
GUK1		0.80907
TNFRSF12A		0.72619
ZMYND10		0.94229
KCP		0.97355
CORO7		0.93494
DPM2		0.90342
KRT7		0.79291
TIMM13		0.71998
TMEM45A		0.91211
USP2		0.90916
WDR63		0.84386
ZNHIT1		0.83183

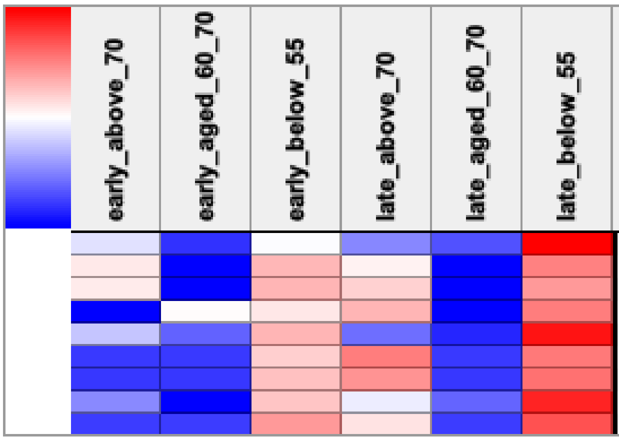

Probe ID	Description	Correlation
SCD		0.85657
SPINK5		0.90141
ALDH8A1		0.88577
BARD1		0.74551
C4orf33		0.87412
MADCAM1		0.91699
SLC16A7		0.93741
SNAP91		0.96501
AREG		0.96973

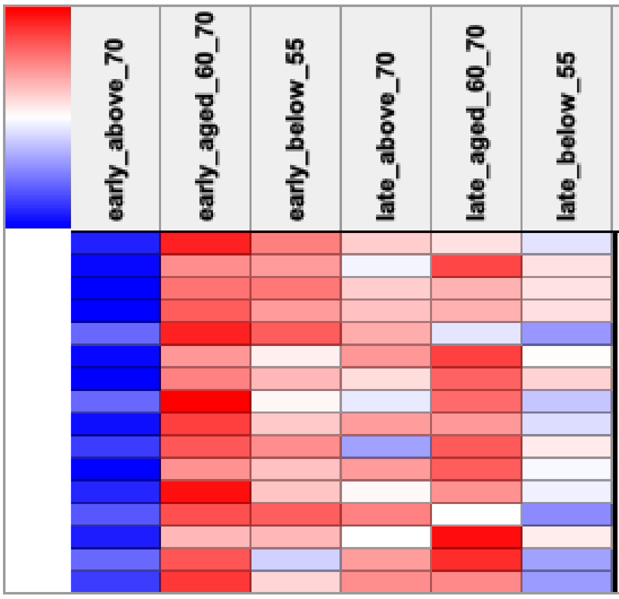

Probe ID	Description	Correlation
GSTP1		0.93686
ID3		0.93239
KPTN		0.95086
DPM3		0.96540
RPL13A		0.78980
ATP6V1B1		0.92327
ELF5		0.95527
NTN5		0.88339
TRIP10		0.98266
VIPR2		0.86957
BCO2		0.95831
DNAJC4		0.96963
MBLAC1		0.81425
SLC25A37		0.87615
TMPRSS9		0.83366
ZNF692		0.94097

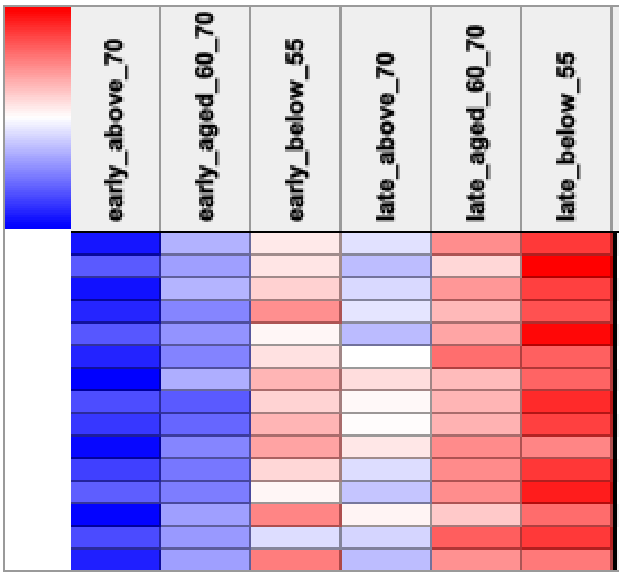

Probe ID	Description	Correlation
CCDC102B		0.98640
F2RL3		0.94071
LAMA3		0.98921
LAMTOR3		0.97861
PLSCR4		0.96058
REXO2		0.98070
SNRPD3		0.95606
TLL2		0.97384
TMEM123		0.98545
SSB		0.95630
TMEM45B		0.99263
TRNT1		0.96731
ATP6V1G1		0.94790
TMEM126B		0.93819
ZC3H15		0.94972

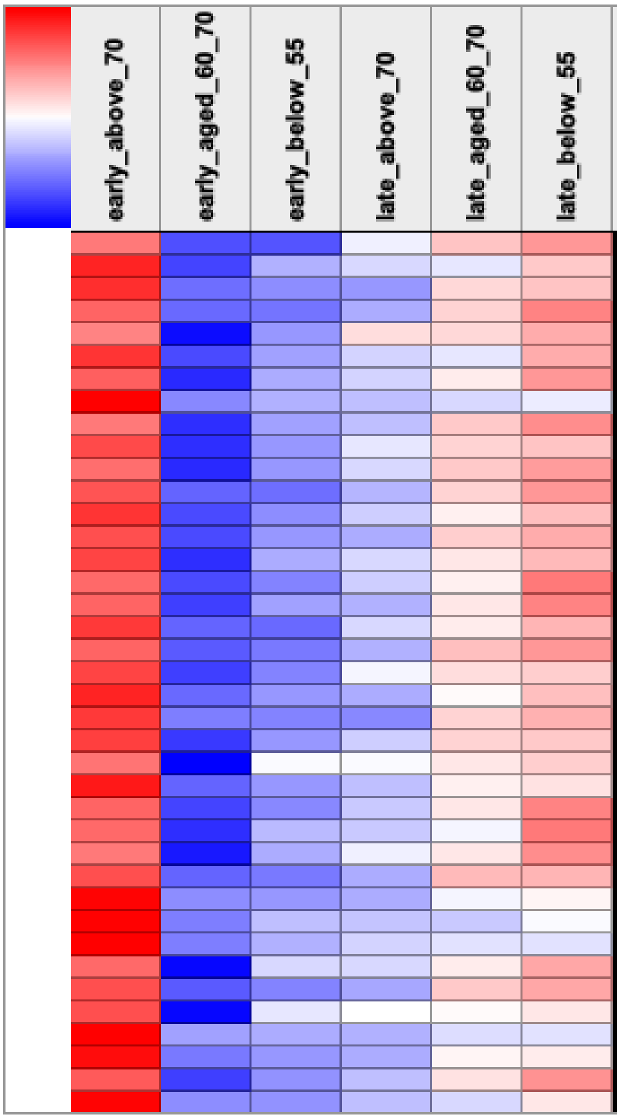

Probe ID	Description	Correlation
ARRDC3		0.93240
ATL2		0.97956
CLIP1		0.97424
FKBP14		0.95745
HS2ST1		0.90989
IARS2		0.98523
KIFAP3		0.98076
MTMR12		0.88723
NUP153		0.96009
NUP155		0.98647
PMS2		0.96928
SNX30		0.97185
ZFP64		0.99860
LRBA		0.98965
APAF1		0.99248
ARL6		0.96694
CAND1		0.97202
CBL		0.98317
CPNE3		0.96177
DIAPH2		0.97927
EARS2		0.98562
FAM160B1		0.95621
FKTN		0.99358
FOXN1		0.87406
NCEH1		0.97611
RCBTB1		0.97719
SNCA		0.95324
TBC1D12		0.94695
UGCG		0.97132
ATF7IP		0.93081
SLC12A8		0.89352
ABHD6		0.89138
ASPH		0.94482
CEP135		0.98050
CHRNA7		0.91078
CREB3L2		0.85323
PELI1		0.95551
SMC5		0.98671
TMPPE		0.92801

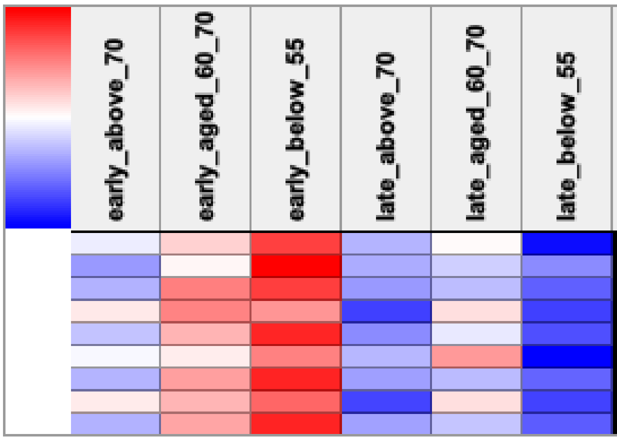

Probe ID	Description	Correlation
CHRM3		0.97202
FFAR2		0.89631
HAPLN2		0.95780
RNF128		0.89154
CCDC57		0.99487
MZB1		0.84992
RPS27L		0.96332
SERINC4		0.93659
ZNF511		0.97172

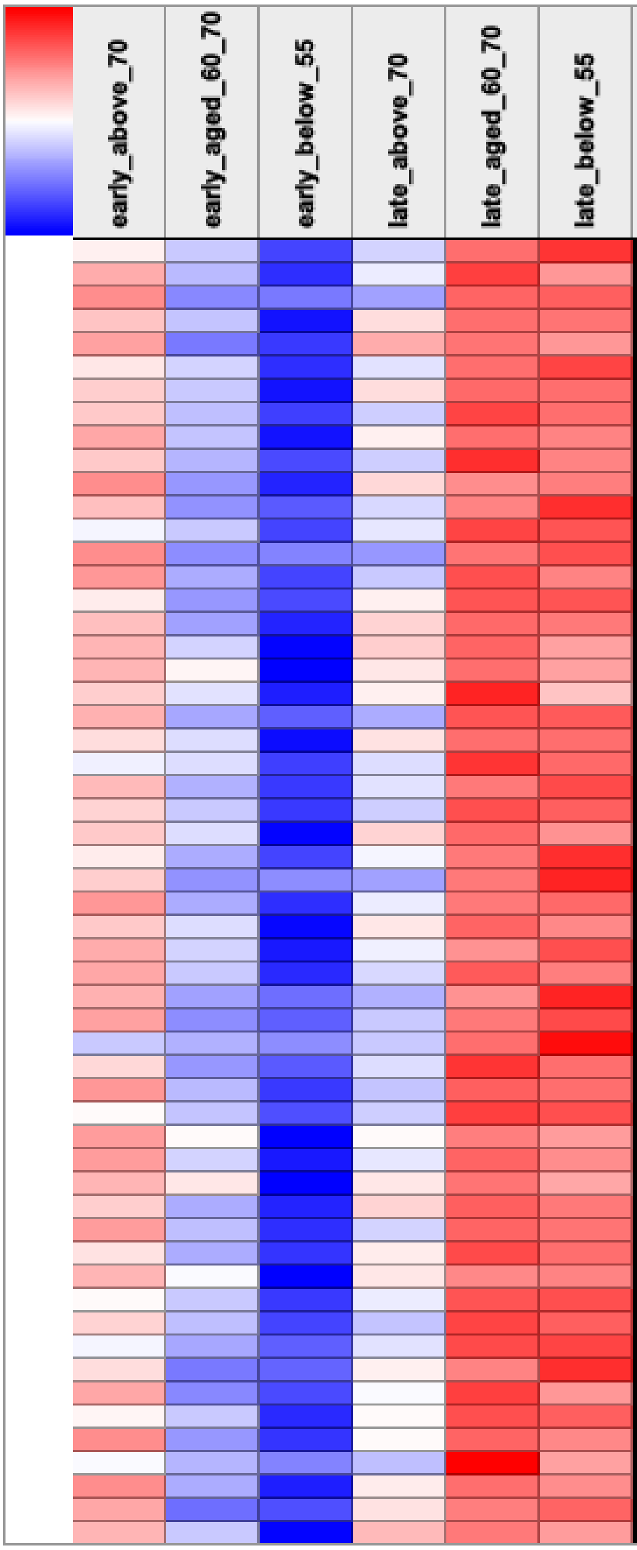

Probe ID	Descripti...	Correlation
FYN		0.96782
GAB2		0.98121
INPP4A		0.91879
KYNU		0.98110
PLEKHA8		0.91291
PLEKHG1		0.97920
PRR5L		0.98230
RASGRP3		0.99038
RFTN1		0.98127
RGS5		0.97800
SEMA4F		0.95410
SH3KBP1		0.96173
SULF1		0.96693
UBR5		0.89551
USP35		0.97631
ABCB4		0.97653
ADAM12		0.97857
APBB1IP		0.95206
ARHGAP9		0.92774
ARHGEF2		0.93059
ARHGEF6		0.96186
CD53		0.97370
CDH11		0.95487
CLIC2		0.99056
COL8A1		0.98851
CPXM2		0.95533
CREB5		0.96973
CTTNBP2NL		0.89605
DOCK10		0.98555
DRAM1		0.97133
ELF4		0.97515
ENPEP		0.98910
FGL2		0.92397
FN1		0.96032
FUT8		0.85027
MAP4K4		0.97651
MYCT1		0.97932
PIK3R5		0.96690
SASH3		0.93045
SCARF1		0.97943
SLC43A3		0.90915
SP140		0.98135
ST8SIA4		0.98716
TFEC		0.98633
TNFRSF1B		0.94007
TNFSF8		0.97853
UACA		0.98382
WIPF1		0.95719
ATXN7		0.93849
CSGALNA...		0.96512
FAM78A		0.98076
MYLIP		0.97505
BCL2		0.86944
CD5		0.97080
DNMT1		0.95109
STK17A		0.93623

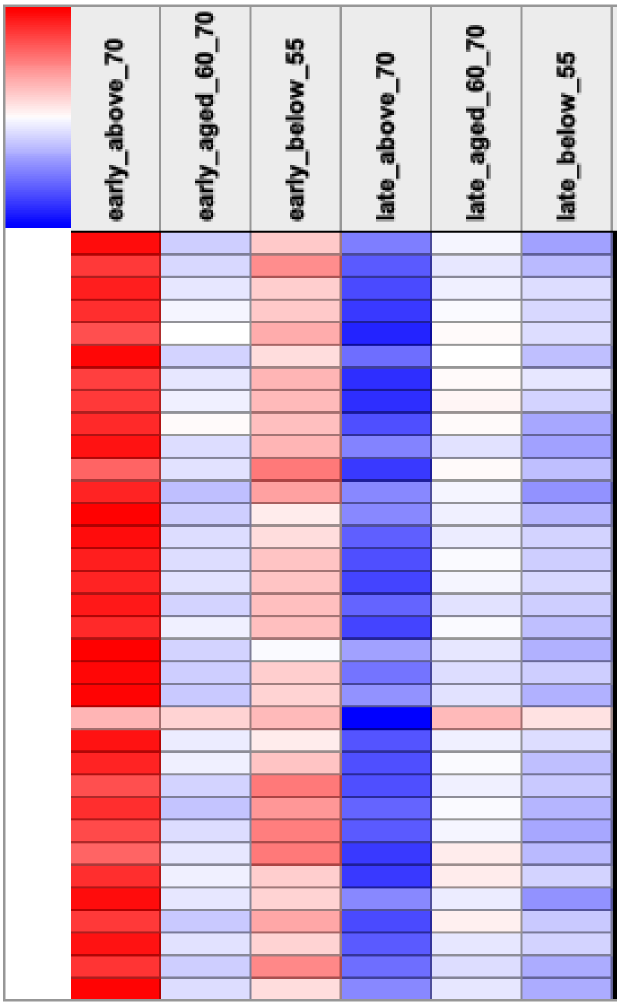

Probe ID	Description	Correlation
CNTN3		0.97942
CYP1B1		0.98363
ERGIC1		0.99096
ITPR2		0.98889
KCNQ1		0.96246
PIAS2		0.98249
PPP1R12B		0.98006
PRDM11		0.98408
RANBP10		0.98683
SEMA3C		0.97756
SLC22A23		0.94899
SMAD4		0.96285
TBC1D30		0.96060
VPS26B		0.98552
BCL2L2		0.99824
IST1		0.99471
COG8		0.99280
DCAF11		0.99591
DNAH5		0.92215
DOCK5		0.98261
IL1R1		0.96191
OSBPL5		0.71366
PSD4		0.97582
SLC27A2		0.99769
TERF2		0.96553
TNFSF10		0.98534
ACTN1		0.96927
BAIAP3		0.94436
DHX38		0.98505
IRS1		0.96404
KRBA2		0.98877
MTOR		0.99226
PTEN		0.97217
TECPR1		0.96736

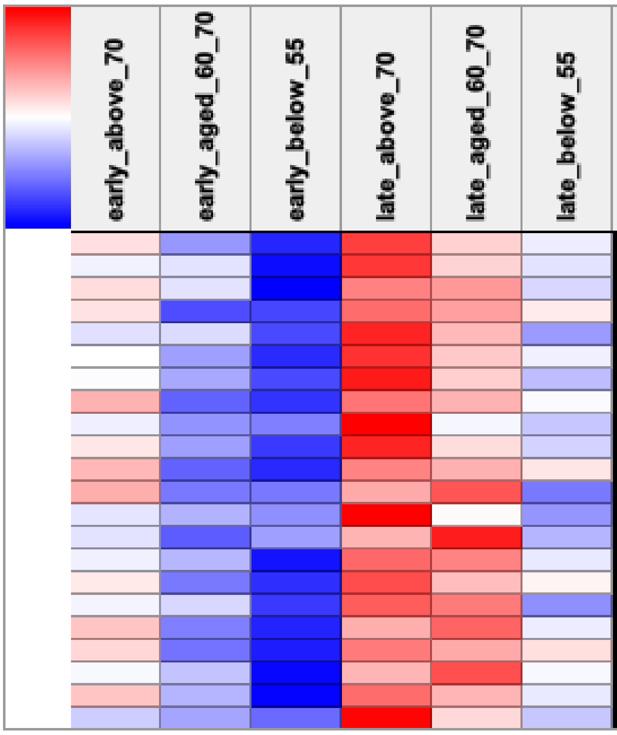

Probe ID	Description	Correlation
FBXL16		0.98347
FTL		0.94499
MAP3K8		0.93771
POLD3		0.94661
STAB2		0.92930
XYLB		0.98321
PABPC4		0.96873
KALRN		0.95179
C5		0.87471
CCDC148		0.97314
DCLRE1B		0.93275
FMO1		0.82806
HEY2		0.84551
LRIG3		0.76654
OSBPL3		0.97471
PTDSS1		0.97809
RHOBTB1		0.93575
SYBU		0.91969
CHMP4C		0.95071
FADS3		0.88403
GATAD2A		0.96976
PSRC1		0.91737

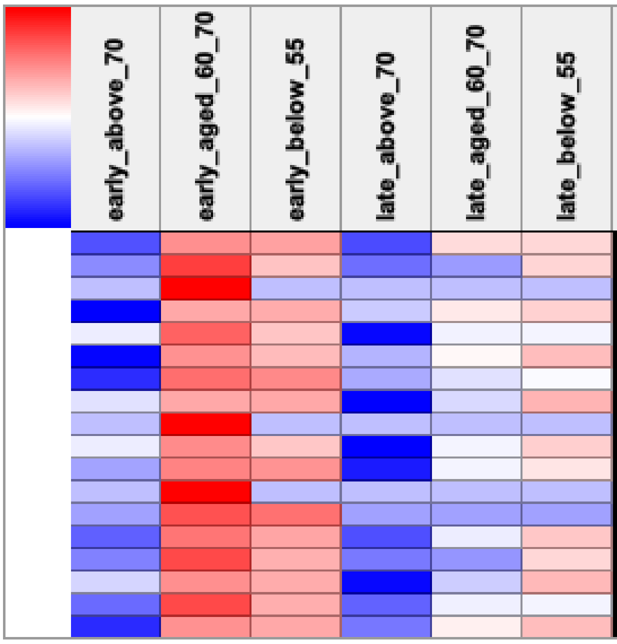

Probe ID	Description	Correlation
BARX2		0.92587
CYP4B1		0.96659
NFE2		0.72019
NRG1		0.76157
SHB		0.87860
TP63		0.82907
FITM1		0.90805
HSD11B1		0.82783
RASSF10		0.72019
C9orf24		0.83864
CCDC159		0.94180
CXCL17		0.72019
EFCAB5		0.83529
HR		0.97350
MT1X		0.96265
SCRN2		0.87627
SOCS1		0.98977
SP6		0.90412

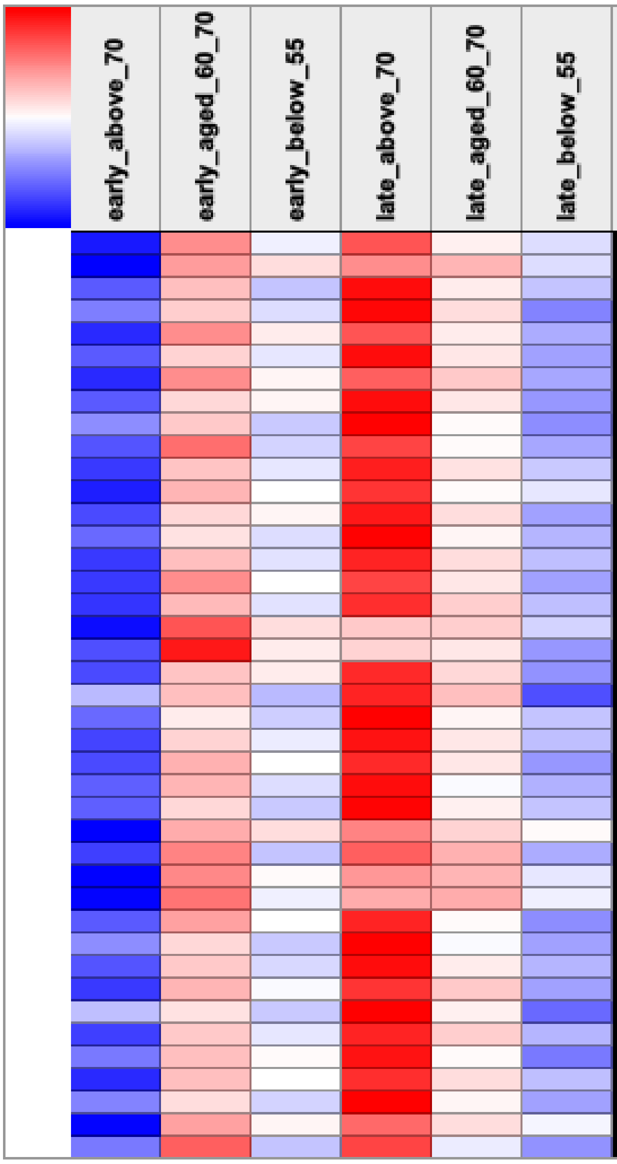

Probe ID	Description	Correlation
OVOL2		0.96480
RFX2		0.88853
RPL28		0.97944
RPL36		0.95917
RPS15		0.97693
RPS27A		0.98406
RPS5		0.97326
RPS6		0.97581
RPL12		0.94074
RPL4		0.96834
RPS16		0.99115
ECI1		0.96986
RPL34		0.98295
RPLP0		0.96273
RPS19		0.99406
RPS9		0.98904
EEF1B2		0.99378
MACROD1		0.79872
NACAD		0.73811
RPS8		0.98441
SLC2A4RG		0.85878
VTN		0.94379
LOC10028...		0.98717
RPL23A		0.99553
RPL29		0.98183
RPSA		0.96628
C1orf54		0.87920
CCDC116		0.95714
FAU		0.88591
INO80B		0.84405
POP5		0.98560
PSMG4		0.93247
RPL14		0.98562
RPL19		0.99403
RPL26		0.85291
RPL27A		0.99290
RPS10		0.96077
RPS11		0.99106
RPS2		0.94759
SLC25A6		0.93062
ZNF593		0.92543

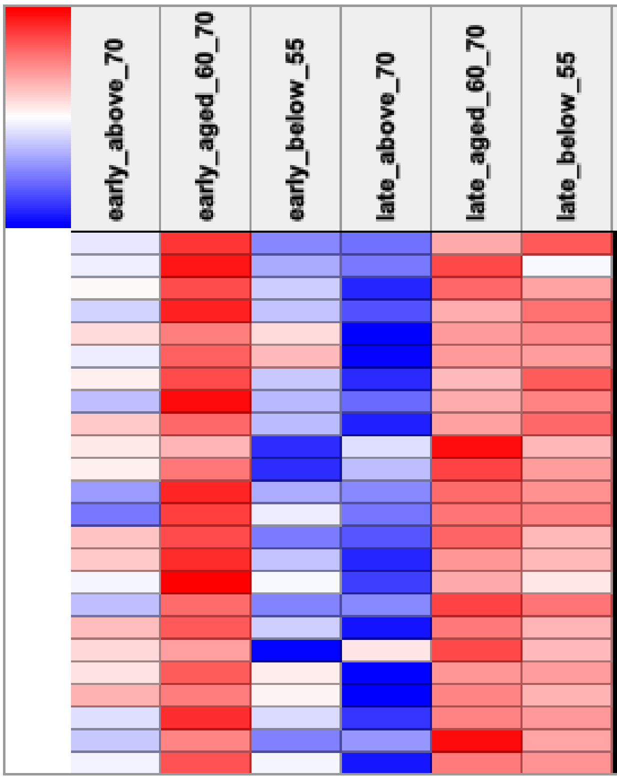

Probe ID	Description	Correlation
ABCC3		0.94820
CCDC146		0.91265
CLDN11		0.99046
ETS2		0.96157
EXTL1		0.87518
MAN2C1		0.86529
RABAC1		0.94874
HSD11B1L		0.94602
AVPR2		0.94803
CCDC18		0.70339
DRP2		0.83252
FHL2		0.93226
GABRE		0.88882
GFPT2		0.95650
PPP1R13L		0.96162
SERPINF1		0.90442
SNCAIP		0.93176
TBC1D2		0.95299
VENTX		0.58844
XDH		0.91938
ZNF423		0.88064
ARHGAP23		0.98344
CLIP2		0.88596
MYOZ1		0.96718

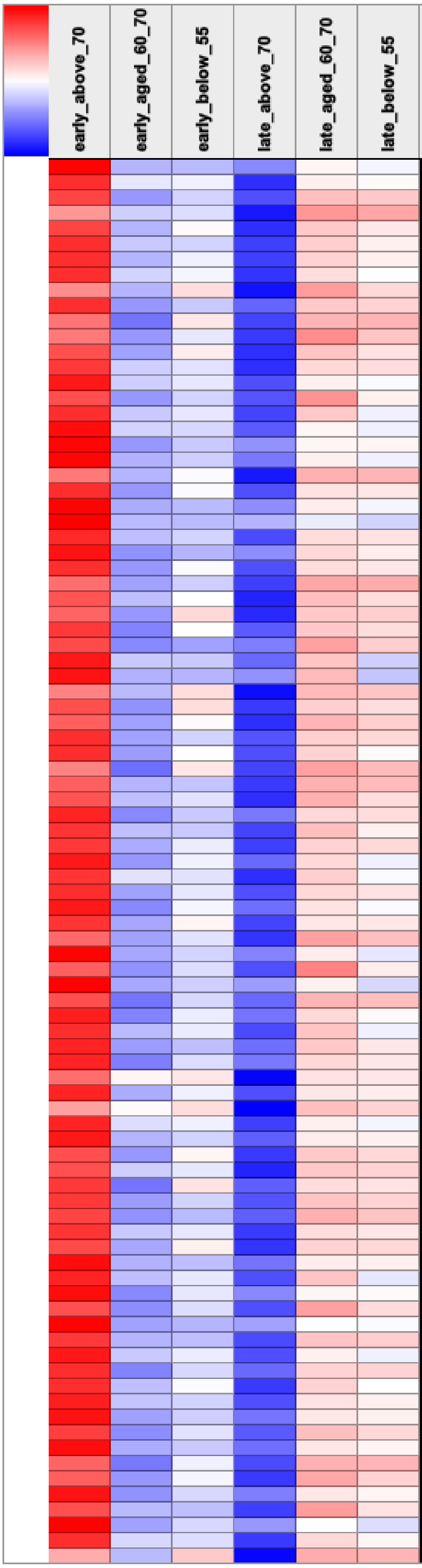

Probe ID	Descripti...	Correlation
AGAP1		0.93506
ARHGAP28		0.96034
ATM		0.98765
ATP8A1		0.88047
BCL9		0.98699
CFLAR		0.99049
CHD8		0.99599
CORIN		0.97833
CPEB2		0.90347
DMXL2		0.98759
FRYL		0.92996
GON4L		0.93666
HEATR6		0.97941
HIVEP2		0.98487
KIAA0556		0.97280
MYO10		0.97055
NR3C2		0.98609
PARD3B		0.96037
PARP4		0.94298
RFC1		0.96088
SAV1		0.93657
SHPRH		0.99114
SLC16A1		0.94328
SLIT2		0.84987
TLN1		0.99266
TRIO		0.95261
TRPM7		0.99290
TTI1		0.94589
TTLL7		0.97807
TUBGCP4		0.94993
USP30		0.98530
RPRD2		0.93511
SRRM2		0.95146
CDK5R1		0.91229
CHD2		0.91634
SMARCAD1		0.97154
ARNT2		0.97141
AP4E1		0.99559
ARID1B		0.99170
ASH1L		0.90483
ATP9A		0.96433
CEP85L		0.98142
FAM168A		0.97271
FOXN3		0.99160
FRMD4B		0.99632
HERC6		0.97999
KCNN3		0.97173
KDM5A		0.99820
LARS2		0.97432
LCA5		0.98884
MAU2		0.95944
PLOD2		0.95075
PRKAB2		0.95152
PTPRD		0.91324
ROCK1		0.96216
SASH1		0.97829
SETBP1		0.98834
SLC23A2		0.98427
SMURF2		0.97456
SP100		0.88368
SYNJ2		0.99219
TGFBR3		0.81630
YLPM1		0.96635
ZFYVE9		0.98494
ZNF141		0.98390
ZNF436		0.97501
ZNF45		0.96149
BRWD1		0.99388
KLF7		0.96990
PRRC2A		0.98980
ATXN1L		0.98312
CDC42BPB		0.96723
DDX60L		0.98212
DENND1C		0.95471
ITGA1		0.97810
MSMP		0.91072
MYO18A		0.98577
MYO1D		0.97430
NF1		0.98327
PPP2R2C		0.98889
SLX4		0.98678
SMCR8		0.97716
TBL1XR1		0.99112
TCP11L2		0.97590
TMEM184C		0.95149
TXK		0.97243
USP24		0.97063
VANGL1		0.96961
ZDHHC23		0.91650
ZHX2		0.98144
ZNF546		0.84065

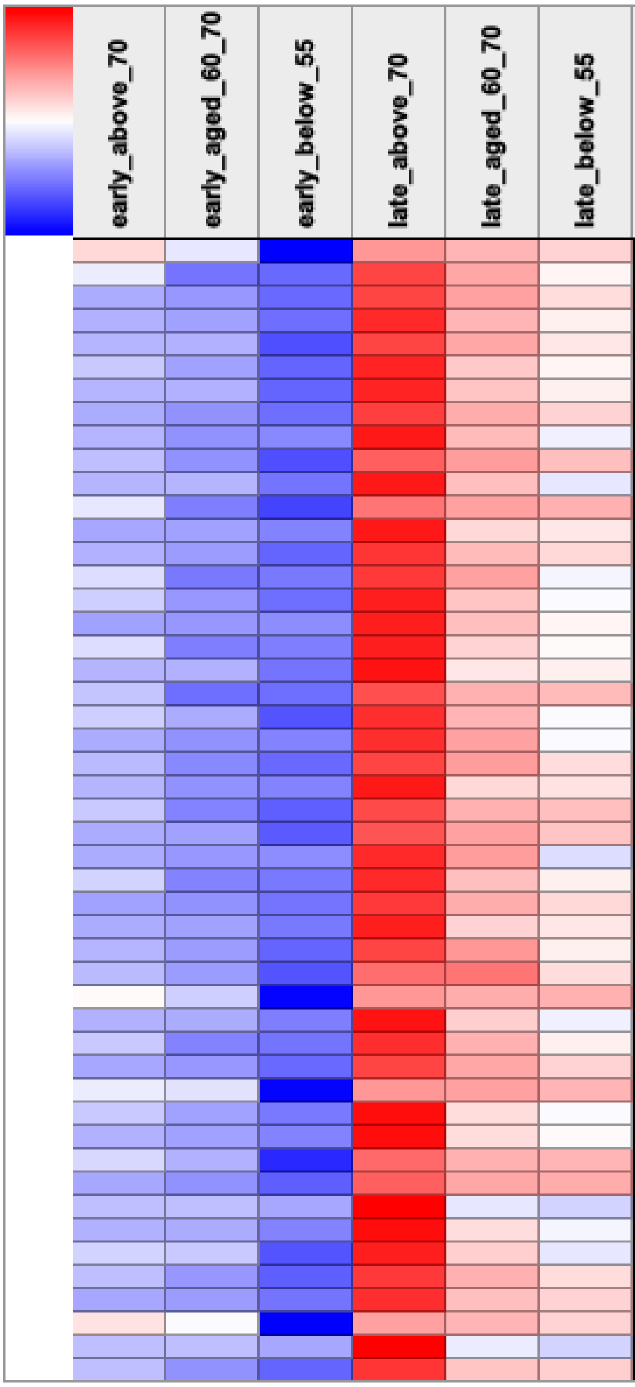

Probe ID	Descripti...	Correlation
ARRB2		0.78655
BRCA1		0.97289
BUB1B		0.99108
CDCA8		0.99586
CDKN2C		0.99288
CDKN2D		0.99500
CELSR3		0.99429
CENPE		0.99330
DEPDC1B		0.97914
DIAPH3		0.97521
EZH2		0.97931
GLCCI1		0.93682
HELLS		0.97792
KIF23		0.99695
NCAPD2		0.97611
PANK1		0.99055
TMPO		0.97777
DSCC1		0.97630
SPC24		0.97627
YWHAG		0.96990
FANCA		0.99250
KIF15		0.98175
BMP6		0.98975
BRIP1		0.98291
CENPI		0.98442
CLSPN		0.98015
FBXO43		0.96091
KIF11		0.98831
KIF14		0.98838
MCM4		0.98720
MKI67		0.99030
PLCE1		0.95388
STK32B		0.84262
ZWINT		0.97629
KNTC1		0.99223
BUB1		0.98954
C1QC		0.84189
CDC6		0.97665
CDCA2		0.97606
CENPP		0.94595
FANCI		0.96086
HNF1A		0.86804
PBK		0.96713
POC1A		0.96974
PRC1		0.99813
RMI2		0.99173
TAGLN2		0.76566
VGF		0.87423
WDHD1		0.99497

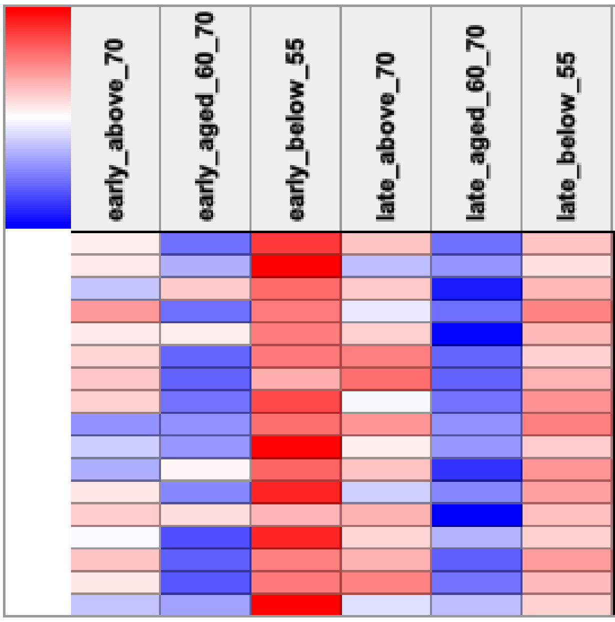

Probe ID	Description	Correlation
ART3		0.98361
CALML4		0.84924
CHGB		0.79496
GALNT5		0.87776
IGSF11		0.85025
KCNQ2		0.91980
LRP2		0.85586
PTH2R		0.95726
SLC35F4		0.86084
TDRD1		0.92364
INSM1		0.85930
ORM2		0.92075
SLC35G1		0.74056
FAM174A		0.91005
GDF5		0.94375
INSIG1		0.92463
MSMO1		0.83299

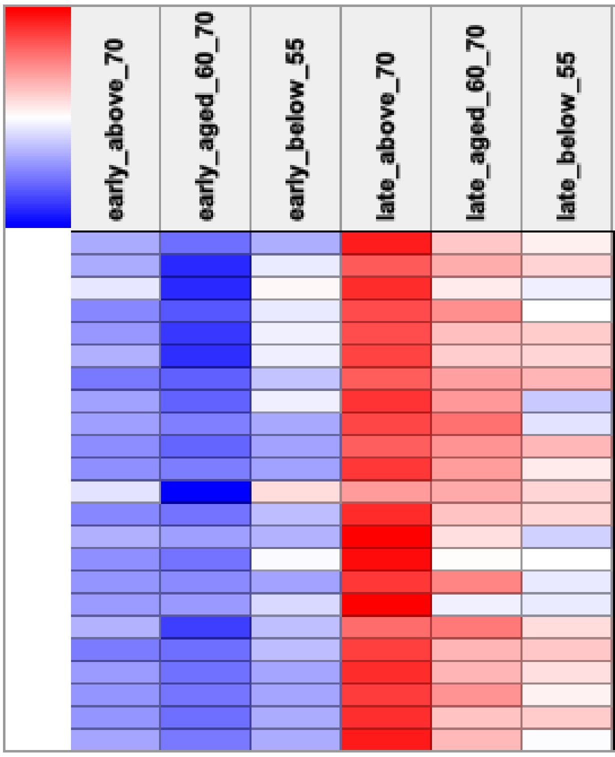

Probe ID	Description	Correlation
C1orf112		0.98812
KLHL7		0.96741
MRI1		0.90529
MSH2		0.97974
TSPAN13		0.97220
YEATS4		0.96962
ZNF92		0.96544
ZFP62		0.95676
CEP72		0.94986
FBXO5		0.95955
GPSM2		0.98207
INHA		0.81166
RNF138		0.98877
TLE4		0.91977
PSMD3		0.93880
ZNF696		0.95860
AP3S1		0.90105
CMTM8		0.95672
MRPL13		0.98276
RUVBL1		0.98862
TRIM59		0.98393
UNG		0.98469
ZNF138		0.98489

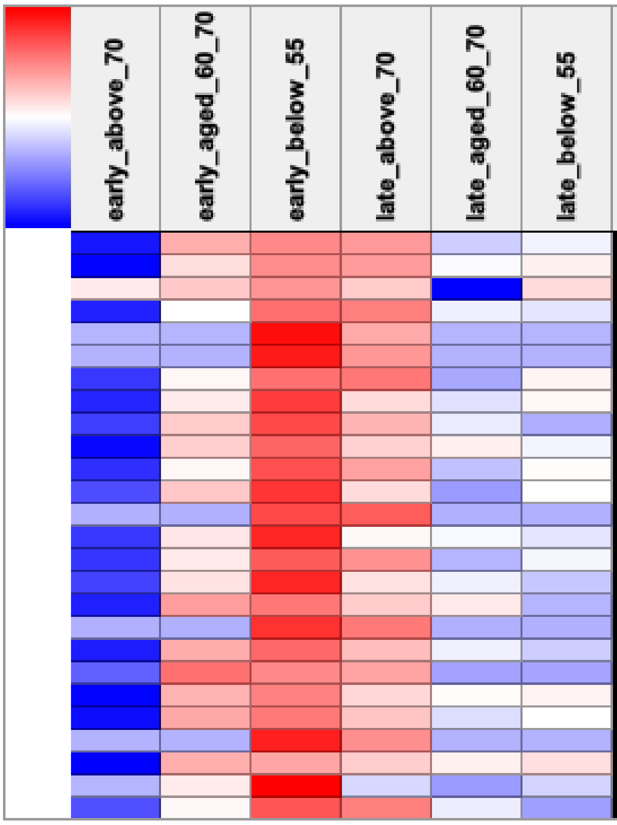

Probe ID	Description	Correlation
BCL11A		0.94277
DGUOK		0.91850
NOG		0.40956
ACSM3		0.96119
CDH19		0.83402
DPP6		0.83593
EBP		0.95150
FBXO15		0.96582
GPM6A		0.97578
LRRC17		0.93343
RSPH4A		0.98625
SERP2		0.95917
SLC24A2		0.80928
ZC3H12B		0.94328
ZC4H2		0.98573
ACOT11		0.96411
CHMP4A		0.89908
CA9		0.82858
COMMD6		0.95352
CYP4F12		0.86358
HMGN3		0.89588
TRAPPC6A		0.93463
ULBP3		0.83527
VSNL1		0.83544
YBX2		0.78646
ZBTB7C		0.95364

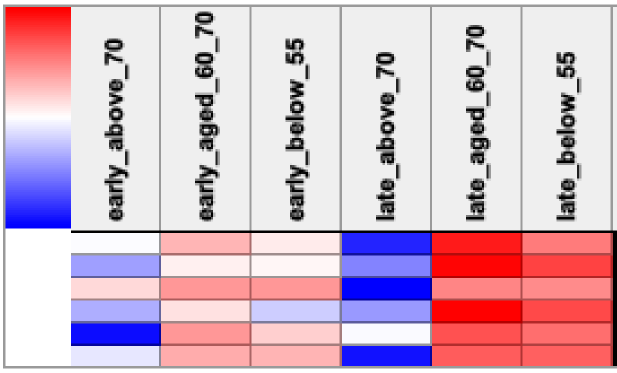

Probe ID	Description	Correlation
ADAMTS16		0.96086
APBA1		0.96146
CTTNBP2		0.83061
MCF2L2		0.92388
MYH3		0.73826
NAALAD2		0.95200

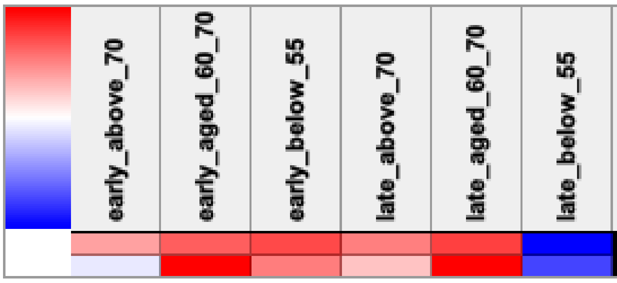

Probe ID	Description	Correlation
ACVR1C		0.96540
RERGL		0.96540

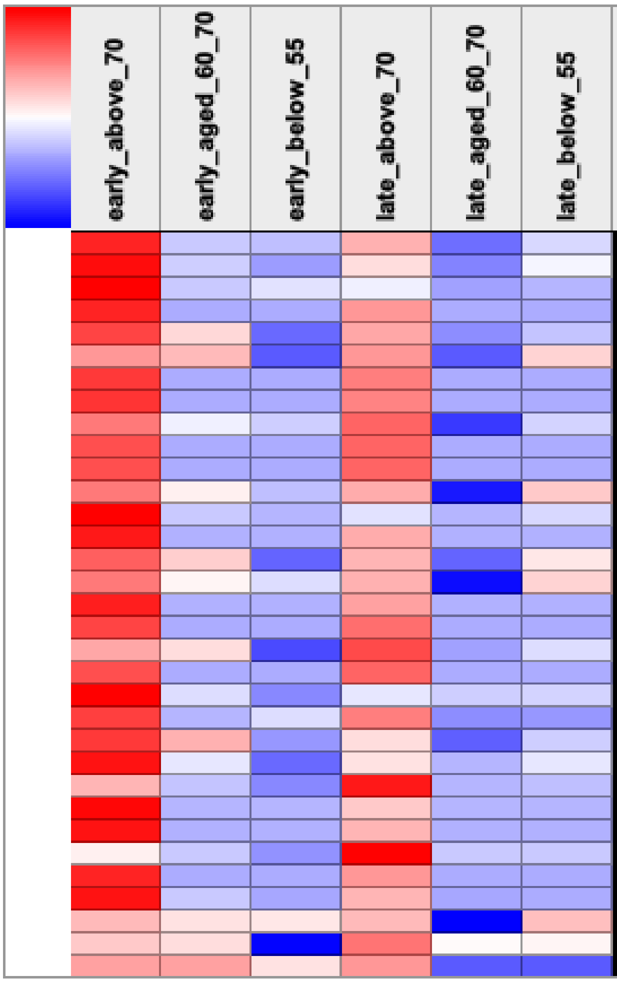

Probe ID	Description	Correlation
ABHD10		0.97542
ERBB2		0.94157
LGALS3		0.84689
NR1I2		0.96782
PPP1R15A		0.94766
SLC26A8		0.78801
C11orf21		0.96786
CR2		0.96847
GNG13		0.91322
HAPLN1		0.95261
IL12RB2		0.95482
LMX1B		0.81211
NACC2		0.83560
PAK3		0.96047
RSPO4		0.87457
SIRPB1		0.79185
SLC10A6		0.96499
SLITRK3		0.96216
SPA17		0.83091
TMEM236		0.95572
TNFAIP8		0.83394
C17orf58		0.94922
GADD45B		0.87654
SLC2A3		0.91406
CDC25A		0.80769
IKZF3		0.93941
KCTD14		0.95566
LNP1		0.65351
ODF3L1		0.96806
OTX1		0.97181
RHOF		0.57791
TPRN		0.58521
ZNF683		0.64975

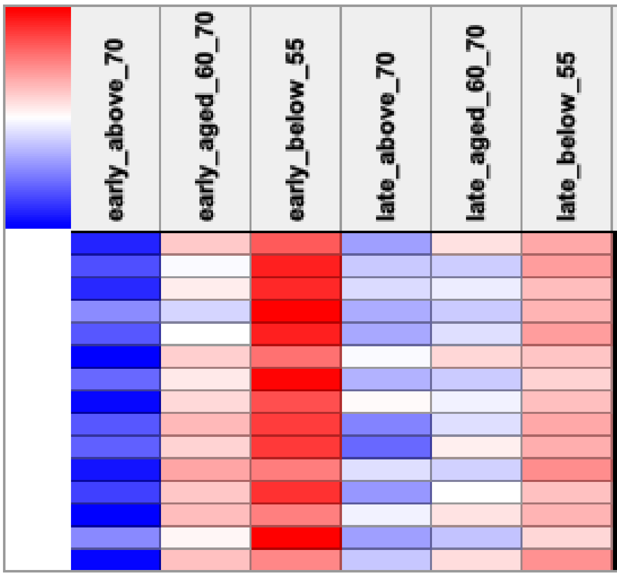

Probe ID	Description	Correlation
INPP1		0.97566
MCOLN2		0.97445
SLC8A2		0.98914
SULT2B1		0.90371
ANXA1		0.97921
CDH26		0.92316
DLEC1		0.95738
DTNA		0.95567
LDHB		0.96977
NINL		0.95068
PTGER3		0.93564
TIMP4		0.98789
FTCD		0.92357
REG4		0.91695
TCAP		0.93719

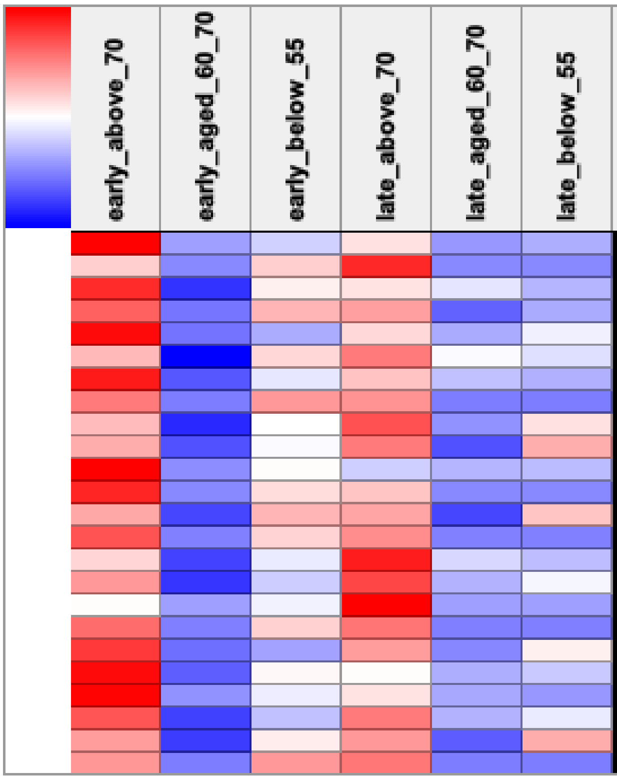

Probe ID	Description	Correlation
BPHL		0.86642
CDHR2		0.83313
NLN		0.89504
PRIM2		0.95247
SCO1		0.86625
TRERF1		0.82986
BNC2		0.93760
ATP6V0D2		0.90474
BCHE		0.87656
CRB2		0.83166
CTH		0.77062
CYSLTR2		0.92904
HSD17B3		0.83520
MS4A1		0.95562
MTAP		0.81936
SLC6A12		0.90818
ZIC2		0.71065
SPATC1		0.94945
ZNF460		0.89935
DEPDC4		0.89057
GSTA4		0.88065
NPNT		0.95232
PSAT1		0.84423
UGT8		0.88943

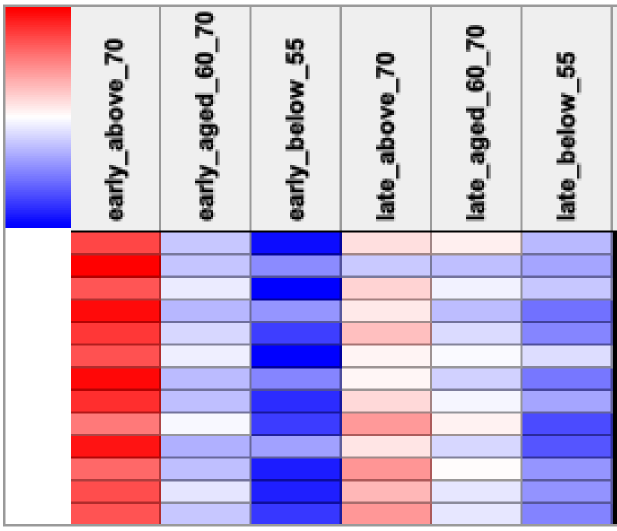

Probe ID	Description	Correlation
ACRBP		0.96915
ARRB1		0.88587
B4GALNT3		0.95133
BHMT		0.93543
GRHL3		0.99619
SRD5A1		0.92445
NUAK2		0.95248
ST3GAL5		0.99347
MTRNR2L1		0.92097
CCDC77		0.92169
HS3ST1		0.95906
IGFLR1		0.98587
TPCN2		0.97624

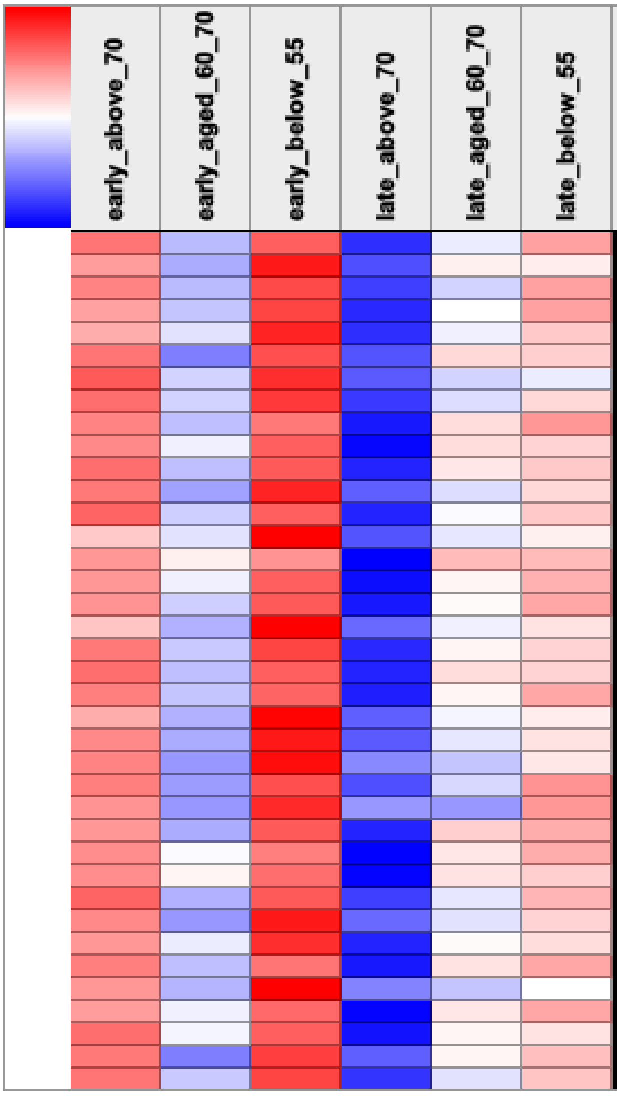

Probe ID	Description	Correlation
CANX		0.98567
CD46		0.97429
COG6		0.98468
ELMO2		0.98949
FAM135A		0.98148
FNIP2		0.95873
MED12L		0.94613
MTUS1		0.98912
PRKCA		0.96458
RGS17		0.96771
RNF141		0.99022
SLC12A2		0.97495
SLC17A5		0.98823
TAOK3		0.92650
HELQ		0.88872
TAF8		0.97389
WWOX		0.98736
ARHGAP6		0.93596
CHSY1		0.99666
CORO2A		0.98368
CWH43		0.98683
ELOVL5		0.95515
ENOX1		0.97902
FAXC		0.92221
NUS1		0.96695
PCYT1B		0.87752
PEX12		0.97347
ST3GAL6		0.94656
TMEM181		0.94783
TOM1L1		0.98706
USP43		0.96700
CPNE4		0.98232
TIGD2		0.97450
UBE2J1		0.91137
OMA1		0.96070
CCDC68		0.96932
MMADHC		0.96712
NT5DC1		0.99574

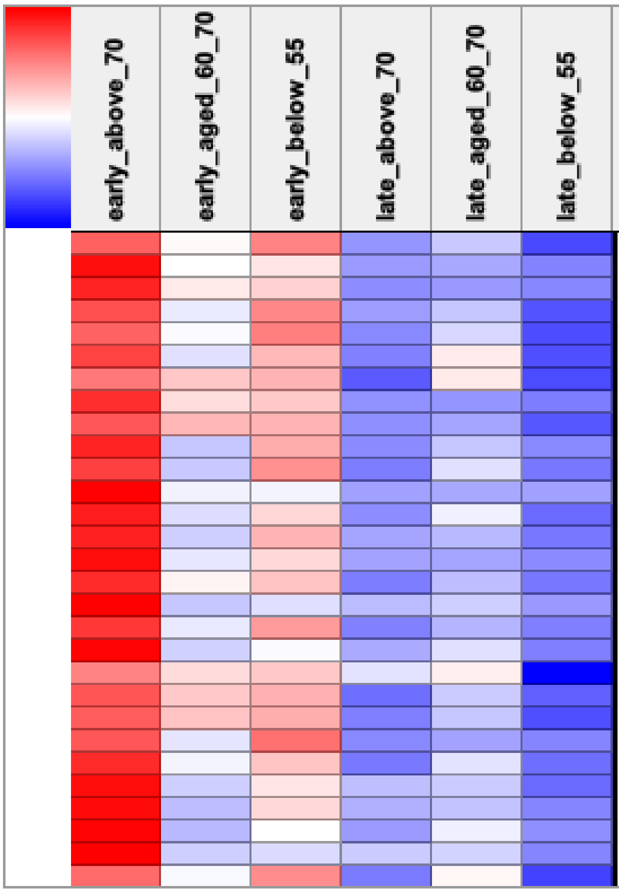

Probe ID	Description	Correlation
C22orf23		0.95614
CDK6		0.97618
GDF15		0.97027
NFIB		0.97077
PASK		0.95547
PHLPP1		0.95992
RNF43		0.88607
RSPO2		0.96641
SLC43A1		0.93713
TBX3		0.97804
WWC1		0.96837
C15orf48		0.93174
ARSG		0.98512
CARD10		0.98523
FUCA2		0.97496
HSD17B6		0.99212
MAPK4		0.89744
PALMD		0.98351
SLC7A1		0.95034
SV2C		0.81136
TCEA3		0.95549
DEF6		0.94937
KBTBD11		0.93657
SNTG2		0.99336
ARHGEF10		0.96833
CADM1		0.96478
CHD7		0.93354
CYP4V2		0.89698
FCRLB		0.93251

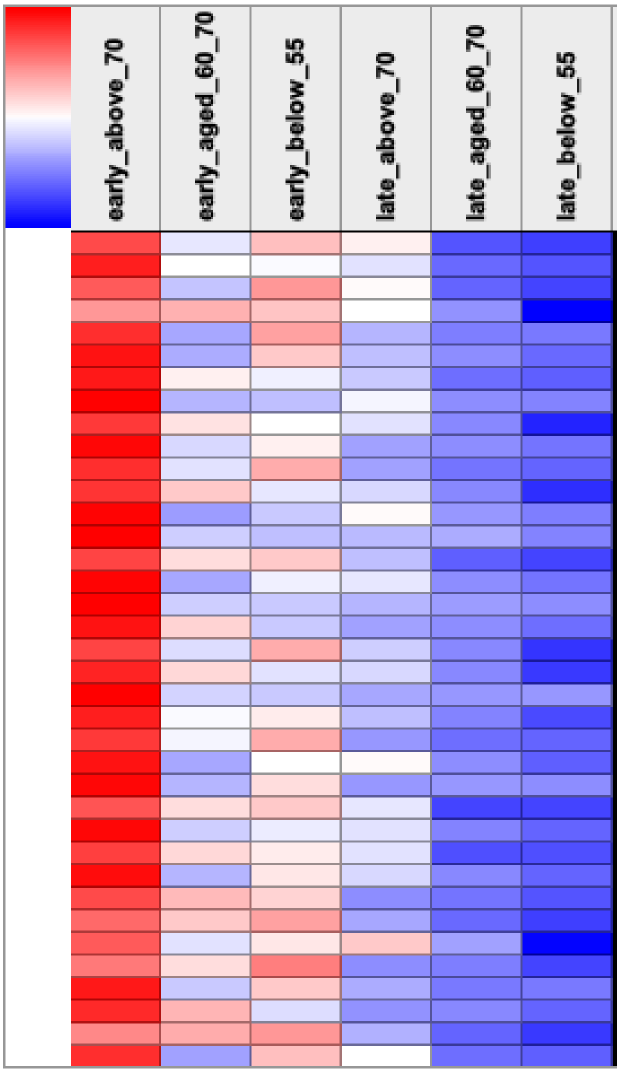

Probe ID	Description	Correlation
AGFG2		0.95601
CASC1		0.98953
FOXP2		0.91996
GCHFR		0.82785
GNPNAT1		0.93652
MCPH1		0.96553
MGST1		0.98637
PNKD		0.91006
RBFA		0.97103
WDR93		0.97719
KCNH6		0.97047
BAIAP2		0.95643
CITED2		0.89553
CUEDC1		0.92260
HMG20B		0.97343
LONRF1		0.94855
MIPEP		0.92458
RAMP1		0.93669
RLN1		0.96963
SAT2		0.96523
SEC14L5		0.91858
SMS		0.99768
ST6GALNA...		0.96452
TNFRSF19		0.94906
TUSC3		0.94259
WNT16		0.95357
C2orf72		0.97610
PAX5		0.97027
ANXA4		0.97683
ELMO3		0.93473
FBXL8		0.92132
NUDT4		0.89019
PGAP3		0.87980
PODXL2		0.97691
RPH3AL		0.92305
SERINC2		0.86413
ZNF589		0.94550

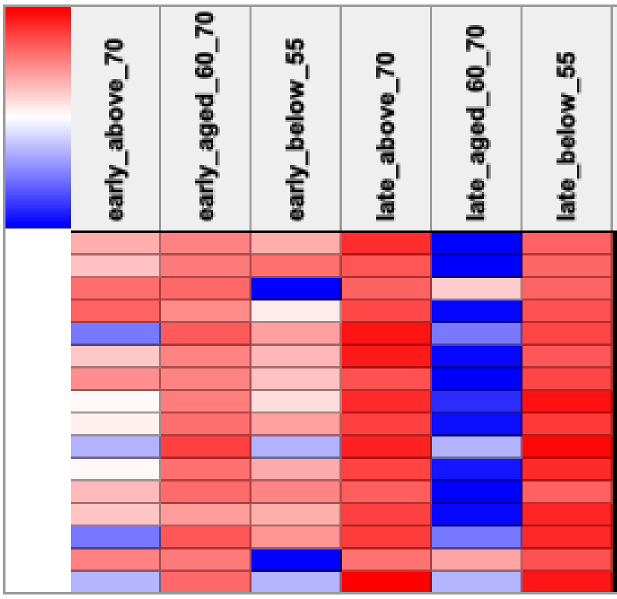

Probe ID	Description	Correlation
BEST4		0.94733
DEPDC7		0.89907
GPRC5A		0.39122
PTPRO		0.91259
SLC17A7		0.85321
CACNG8		0.96822
CEACAM21		0.94137
FGF14		0.99372
MORN3		0.96852
POF1B		0.85548
SCG3		0.97352
TAF7L		0.91675
AMHR2		0.95876
FOXC2		0.84648
PLAUR		0.30576
TMEM150B		0.85651

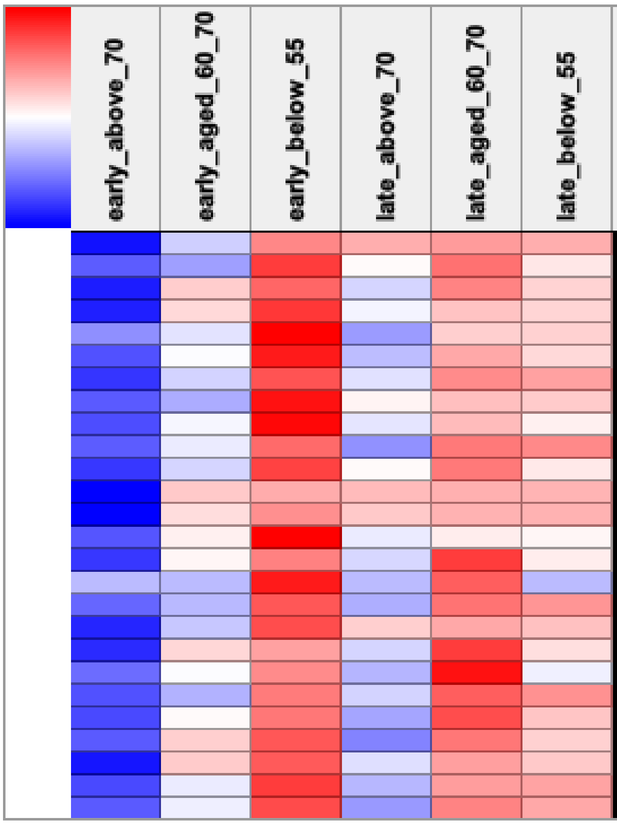

Probe ID	Description	Correlation
IFT20		0.87799
THSD7A		0.93952
ANKRD10		0.96844
BMP7		0.95281
DNASE2B		0.87519
FBN2		0.97413
FGF13		0.98512
IRAK1BP1		0.92116
MMEL1		0.95310
NCALD		0.93638
NKAIN1		0.97879
NTHL1		0.82408
PYCR2		0.87798
THPO		0.88756
TXNIP		0.94893
ELAVL3		0.79018
ATG9B		0.94275
C1orf131		0.94046
CCDC40		0.91265
GAPDHS		0.86619
MTHFD2L		0.93680
PNISR		0.95578
SH3RF3		0.92860
SLC25A45		0.96428
TUBD1		0.97933
WNK2		0.96453

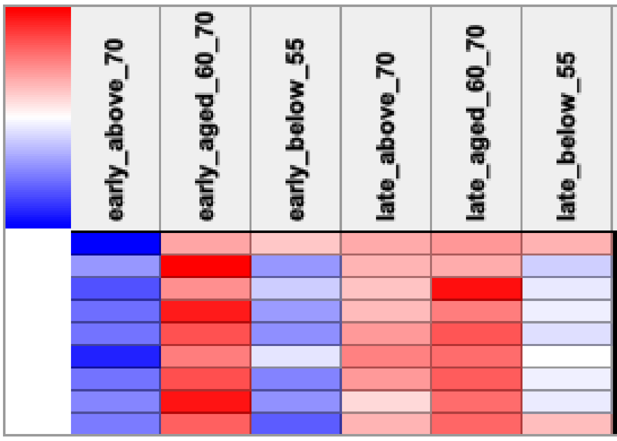

Probe ID	Description	Correlation
CACNA1A		0.76508
CCR10		0.91040
MEOX2		0.93380
TMEM91		0.98133
MC1R		0.98484
CLASRP		0.94668
DCST2		0.98622
NFATC4		0.96119
RNF207		0.93743

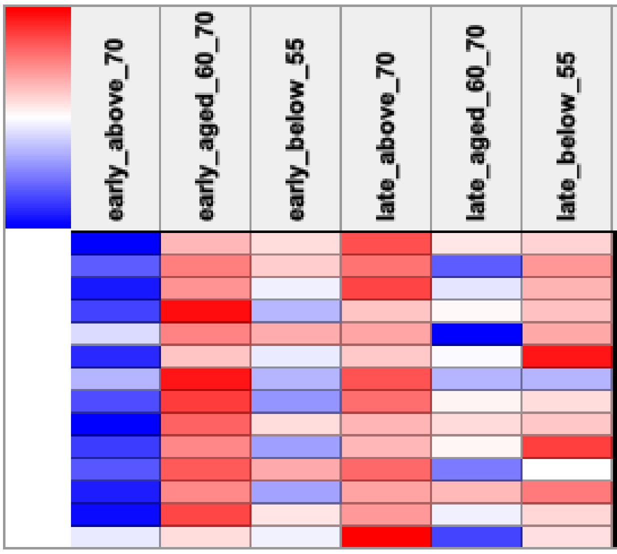

Probe ID	Description	Correlation
COX6B1		0.89058
POU2AF1		0.90751
RAB34		0.97289
CCDC81		0.89059
CDHR3		0.60352
TNNC1		0.78721
VTCN1		0.74535
CHCHD10		0.91294
MYL6B		0.89705
ANXA2		0.85095
IL17RE		0.87962
ITPKA		0.84867
MRPL55		0.95412
SGK2		0.60352

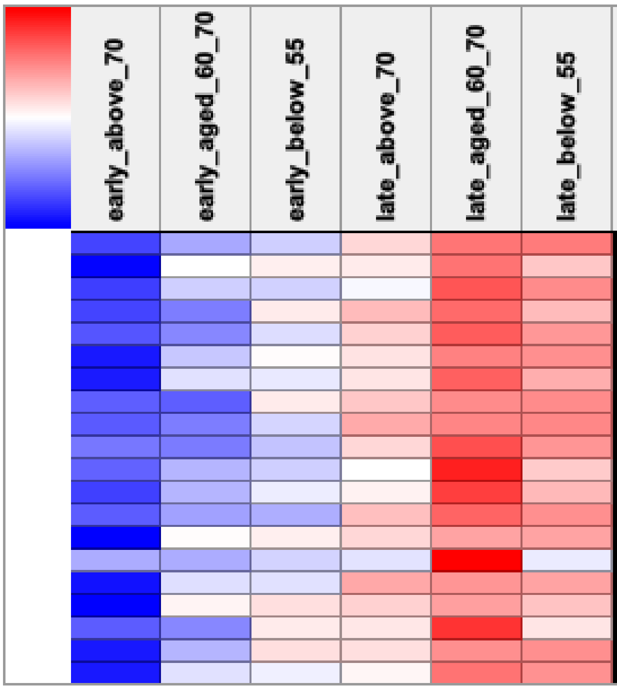

Probe ID	Description	Correlation
BTG1		0.97860
CLK1		0.93548
DDC		0.97334
FAM76B		0.96368
NAA40		0.97881
PTK6		0.98230
RGS3		0.98328
SLC4A8		0.92140
TDG		0.94269
SUN1		0.94095
LRRC39		0.94178
PNN		0.98763
PPFIA2		0.95536
TRMT112		0.90915
LHX4		0.75446
PSMC6		0.94847
SS18L2		0.88775
TRIM52		0.93543
ZMYND8		0.96577
ZNF438		0.97404

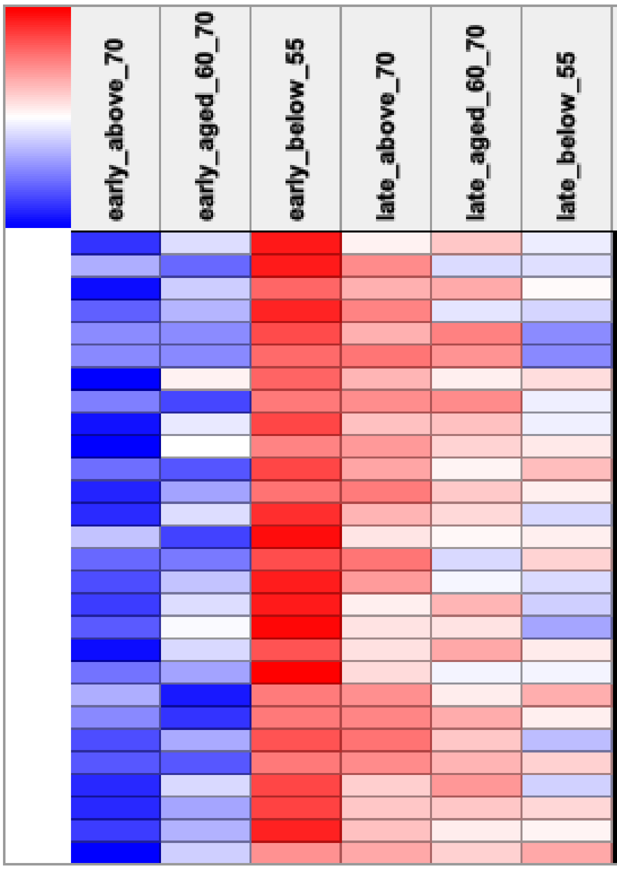

Probe ID	Description	Correlation
FANCL		0.94115
TSPAN8		0.87419
ZNF385B		0.95808
MARCKSL1		0.94743
AFF2		0.87345
CDH15		0.86273
COX7A2		0.88777
ELOVL4		0.89611
FRA10AC1		0.95470
IMMP1L		0.89061
KCNE4		0.92505
LOX		0.96778
PLA1A		0.97021
PLP1		0.82990
SRSF12		0.91484
SYCP2L		0.96661
VLDLR		0.93385
HSD11B2		0.88225
ZNF547		0.93500
ZNF563		0.93303
CAPN5		0.78230
CCDC138		0.88162
CDH4		0.96307
FRMD5		0.92932
LEKR1		0.94399
MYCBP		0.97829
NEU1		0.98498
SYNCRIP		0.88078

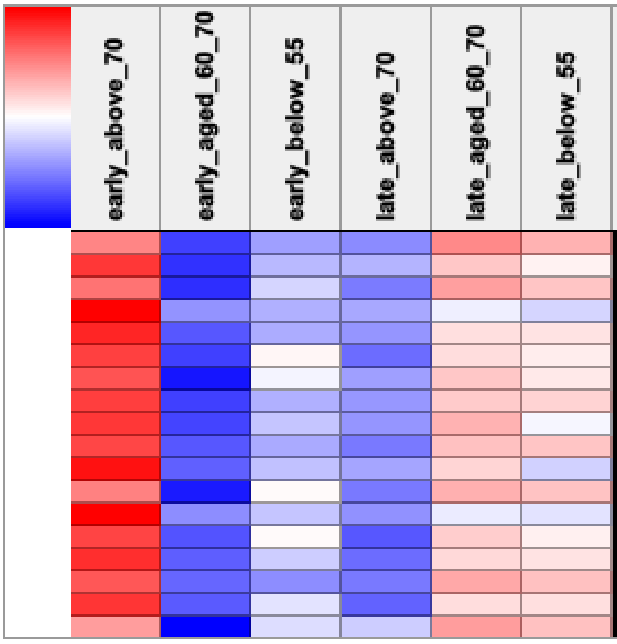

Probe ID	Description	Correlation
BAZ2A		0.92835
GDAP1		0.98522
HEATR1		0.96907
LATS2		0.87939
MAN1A1		0.98639
NAF1		0.97158
RAD50		0.97507
RASA2		0.99320
XPO4		0.98939
ZBTB25		0.98556
ZBTB37		0.95235
SMG8		0.94083
PDE10A		0.90262
DCBLD1		0.96715
MFN1		0.99022
PARP9		0.95806
RICTOR		0.98383
ZNF860		0.88116

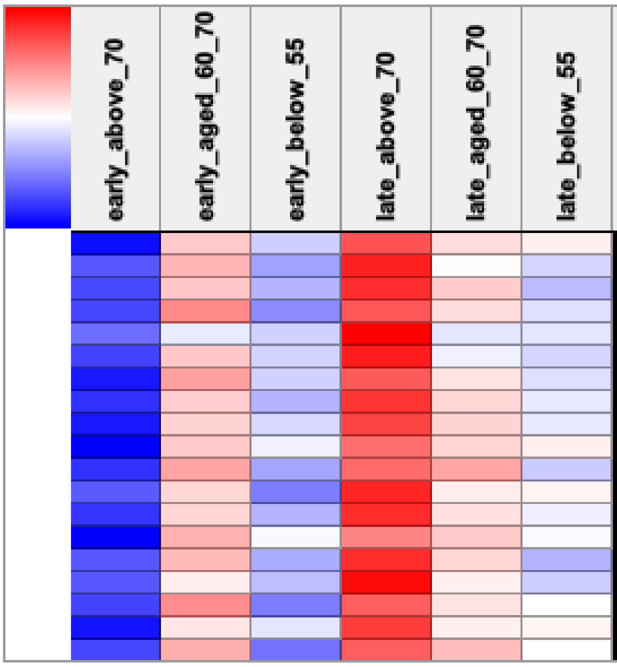

Probe ID	Description	Correlation
C19orf53		0.97967
CLN6		0.97913
EEF1D		0.98313
EMP3		0.96564
HOXA3		0.89890
HSPBP1		0.97561
POLR2I		0.98259
RPLP1		0.99878
THOC6		0.98815
MRPL28		0.94339
KRTCAP2		0.95947
POLR2L		0.96161
BCL7C		0.99623
PRRT4		0.92382
PUS1		0.97353
RPL35		0.96626
S100A16		0.95614
TOMM40		0.96742
VMO1		0.95472

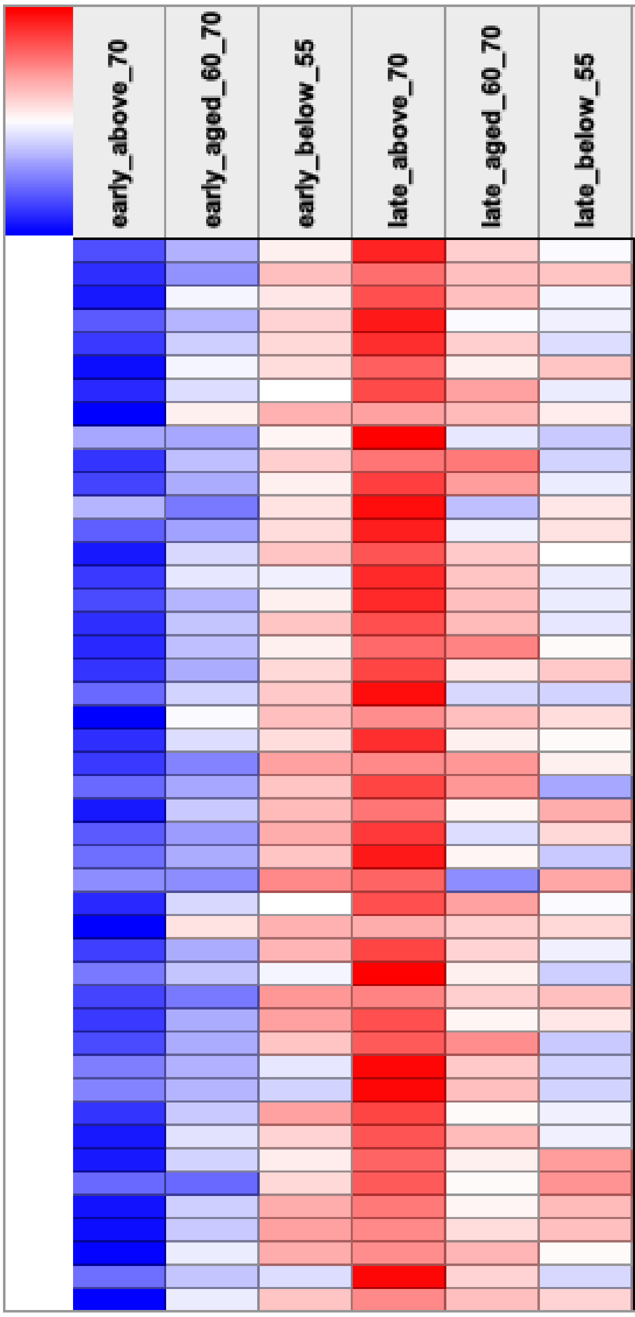

Probe ID	Descripti...	Correlation
CBFB		0.98476
CBR4		0.95941
CHCHD2		0.96706
COX7A2L		0.96978
COX7C		0.98621
FOXRED1		0.94731
FRS3		0.95724
GSTO1		0.87894
METTL21A		0.83948
NFE2L3		0.92062
NOP58		0.96722
PBLD		0.79515
PSPH		0.95842
SNRPB		0.98916
TRIAP1		0.95766
GEMIN6		0.98116
SNRPD1		0.98957
NAA38		0.94282
BET1		0.98148
CRLS1		0.91716
EPB41L3		0.92140
EXOSC8		0.98695
FBXL2		0.91748
GRB14		0.90591
NDUFA8		0.93513
NUFIP1		0.93595
PCDHB3		0.93872
RASGEF1C		0.72422
RPIA		0.96683
SPATA9		0.84582
TMEM97		0.98928
TUBB1		0.90983
CETN3		0.90574
FAM200A		0.97711
ALPK2		0.94040
C5orf34		0.90653
COX17		0.88632
EEF1E1		0.97459
HOXA4		0.97953
HSPE1		0.92489
LIMS1		0.87244
MLF1		0.93955
POLR2G		0.92986
PRKAR1B		0.93031
RECQL4		0.91600
SNRPG		0.93310

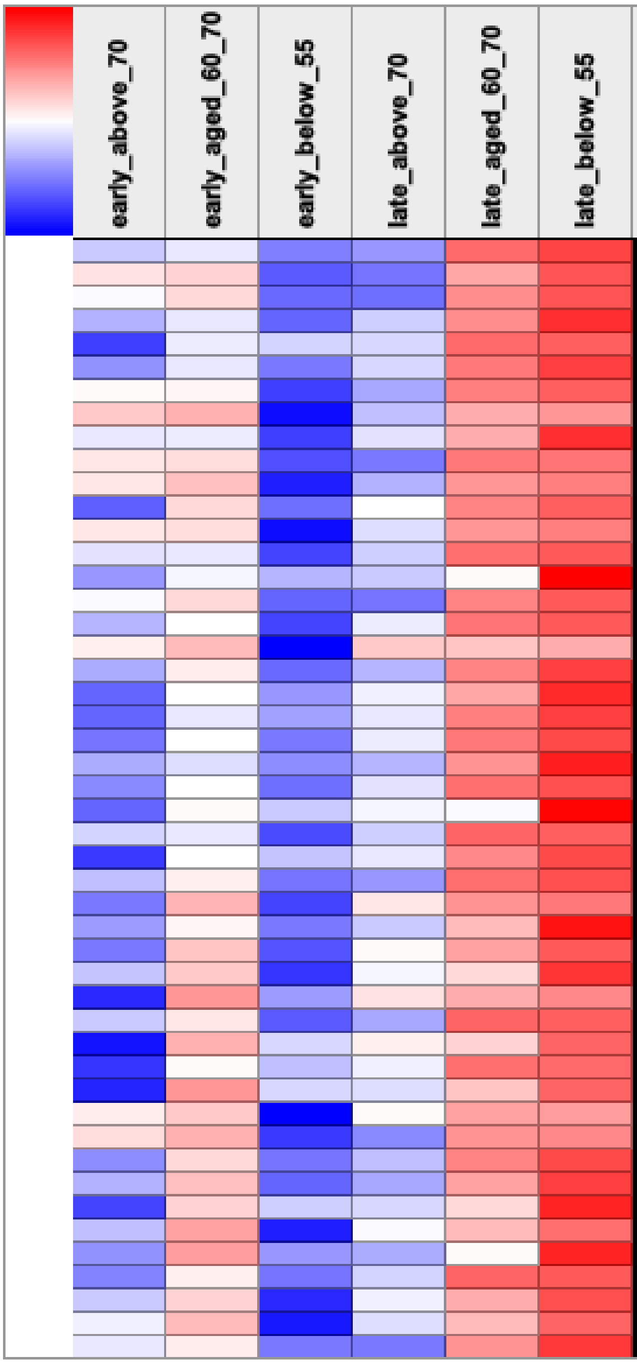

Probe ID	Descripti...	Correlation
EFNA3		0.94499
ESR2		0.87071
ETV1		0.90232
ICAM2		0.98118
LGALS8		0.83029
MECOM		0.97144
MOXD1		0.92697
PLAC8		0.79895
RGS1		0.94865
SH2D3C		0.86710
PLCD3		0.87884
RARG		0.94642
BCAT1		0.87250
CARD6		0.94853
CCL21		0.84797
CLEC4F		0.90771
FBLN5		0.97583
IGFALS		0.72893
LUM		0.99517
MID1		0.93038
SDC4		0.92065
SFRP2		0.96368
TBC1D22B		0.95334
THBS4		0.97805
TMEM176B		0.79751
TNFSF13B		0.94999
VAV3		0.86300
VDR		0.96942
JDP2		0.92562
KLHL35		0.97027
PLEKHA4		0.96092
ACHE		0.94053
ADAMTSL5		0.79200
AXL		0.97029
C19orf73		0.72518
CNPY4		0.84906
EPHA1		0.76243
IGDCC4		0.81646
MLKL		0.84325
MST1R		0.99097
NOS3		0.98518
NTNG1		0.83337
RHOV		0.92152
SLPI		0.87572
SPSB1		0.97089
TYMP		0.96641
UPP1		0.91534
ZNF385A		0.92890

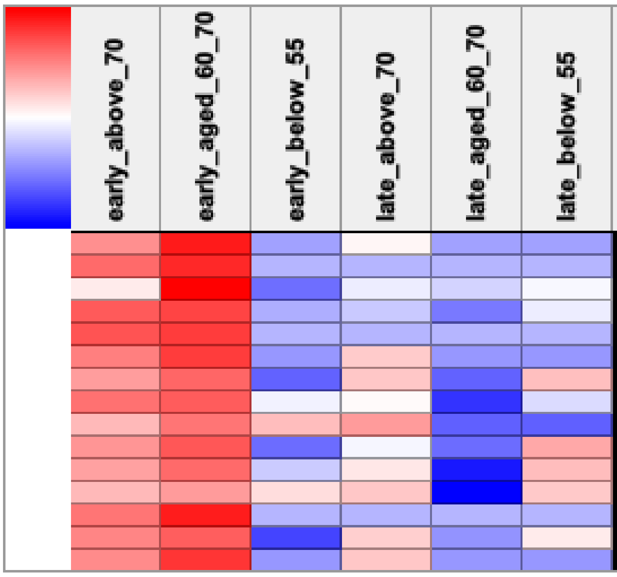

Probe ID	Description	Correlation
CEACAM5		0.95985
C22orf24		0.91789
FOSL1		0.83617
SERTAD1		0.96572
CDH22		0.90999
CERS3		0.94841
GABRP		0.88668
SELE		0.93914
SPOCD1		0.68223
WIF1		0.88634
B3GNT3		0.87562
GPR3		0.70697
GPR39		0.91901
P2RY2		0.90482
SYNPO2L		0.94571

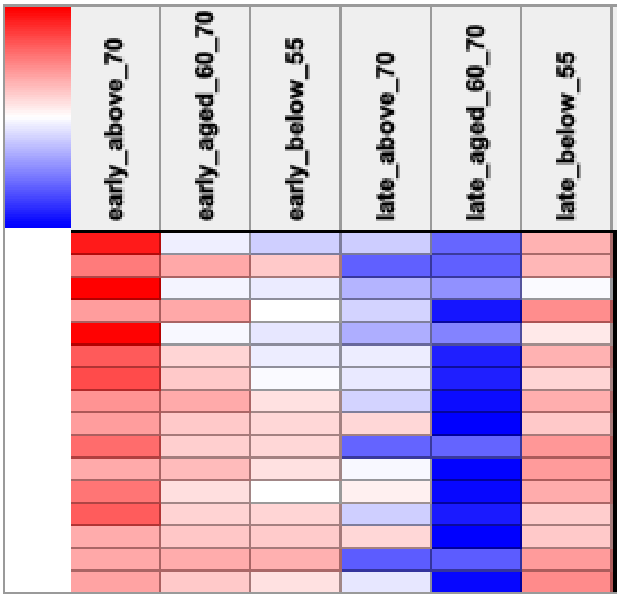

Probe ID	Description	Correlation
ACOT2		0.89305
ANGPTL6		0.89447
FAM83F		0.79515
ITGB6		0.96232
C17orf97		0.84294
CCL20		0.98255
FRMPD1		0.98067
MS4A2		0.97679
MTMR8		0.91232
PHEX		0.90797
SCML4		0.94723
CR1		0.96219
ERBB4		0.99124
CELA2B		0.89656
EVX1		0.85255
SAMD12		0.95934

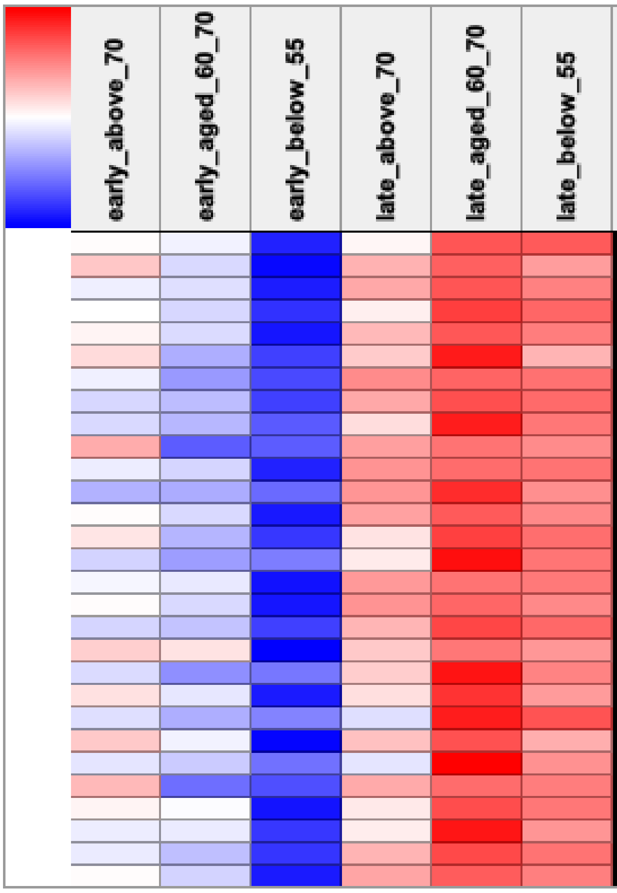

Probe ID	Description	Correlation
CTSC		0.96720
GPNMB		0.96413
IRF8		0.98762
KIF17		0.98644
PITPNM1		0.99179
RSAD2		0.96766
SLC20A1		0.96456
SMAD7		0.98407
TTYH3		0.97479
ARG1		0.88381
BST1		0.97596
CYTH3		0.93672
EEF1A2		0.98640
EPB41L2		0.98907
GIMAP2		0.93635
HAVCR2		0.96917
MS4A4A		0.97726
PLS3		0.98654
PRG4		0.91650
ST6GALNA...		0.94810
STAT4		0.97802
TM6SF1		0.90158
VAV1		0.95329
DCLK2		0.91334
GMPS		0.92171
PLCB3		0.97143
PLXDC1		0.97106
XKR9		0.99598
ZSWIM4		0.98977

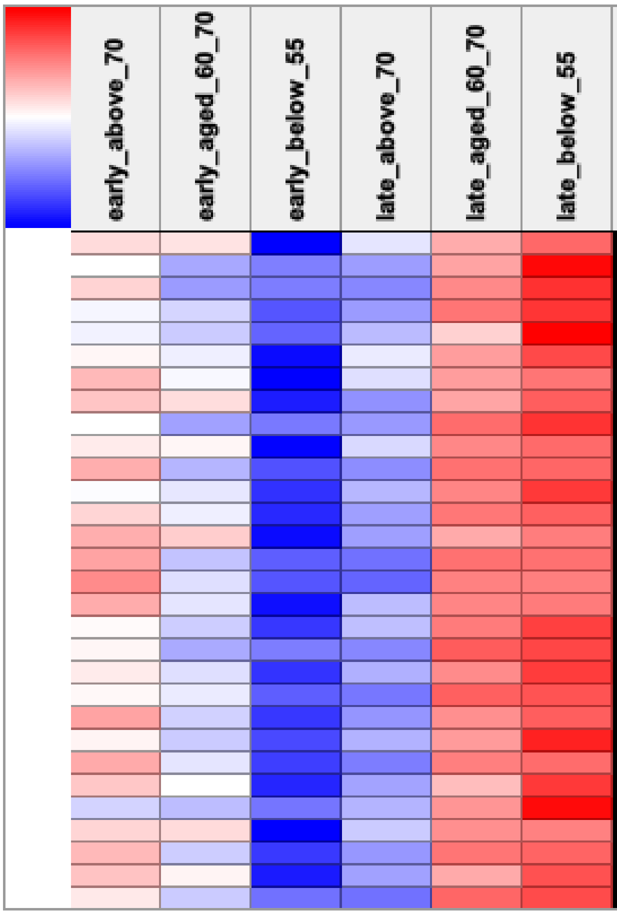

Probe ID	Description	Correlation
BTG3		0.92780
CPT1A		0.93104
HECW2		0.94805
PLBD1		0.97962
SLC7A5		0.91526
SRGN		0.95730
HLA-DMA		0.94582
ACVRL1		0.96424
CALCRL		0.94740
CCRL2		0.95604
CD96		0.97027
CYTIP		0.98675
EHD4		0.99441
FGR		0.92999
GIMAP6		0.95289
HMCN1		0.93388
LCP2		0.96756
PPP2R5B		0.98656
SH3BP5		0.94578
SIGLEC10		0.99636
TIE1		0.96977
TMEM47		0.98173
VNN1		0.98290
VWF		0.97613
ZNF385D		0.97612
KCTD12		0.92024
C1S		0.93517
FMNL1		0.98829
GPR4		0.97869
KLF13		0.96505

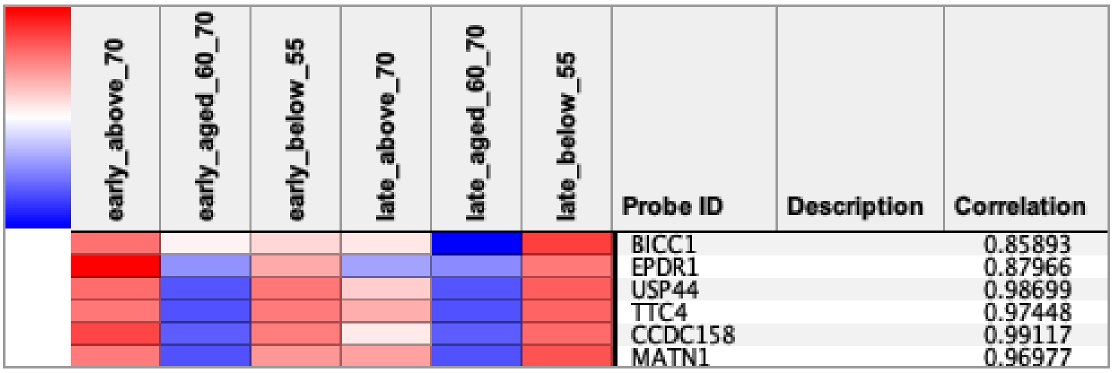

early_above_70
early_aged_60_70
early_below_55
late_above_70
late_aged_60_70
late_below_55
Probe ID
Description
Correlation
BICC1 0.85893
EPDR1 0.87966
USP44 0.98699
TTC4 0.97448
CCDC158 0.99117
MATN1 0.96977

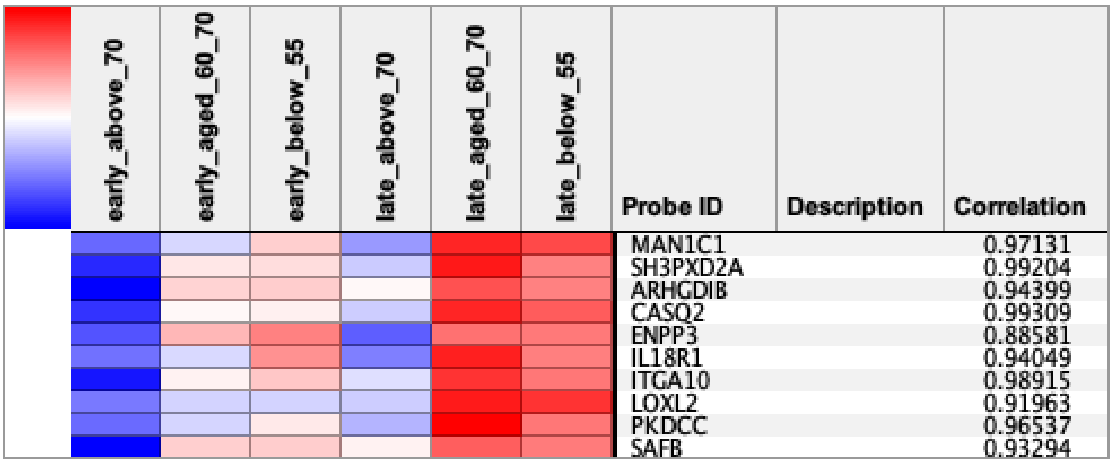

early_above_70
early_aged_60_70
early_below_55
late_above_70
late_aged_60_70
late_below_55
Probe ID
Description
Correlation
MAN1C1 0.97131
SH3PXD2A 0.99204
ARHGDIB 0.94399
CASQ2 0.99309
ENPP3 0.88581
IL18R1 0.94049
ITGA10 0.98915
LOXL2 0.91963
PKDCC 0.96537
SAFB 0.93294

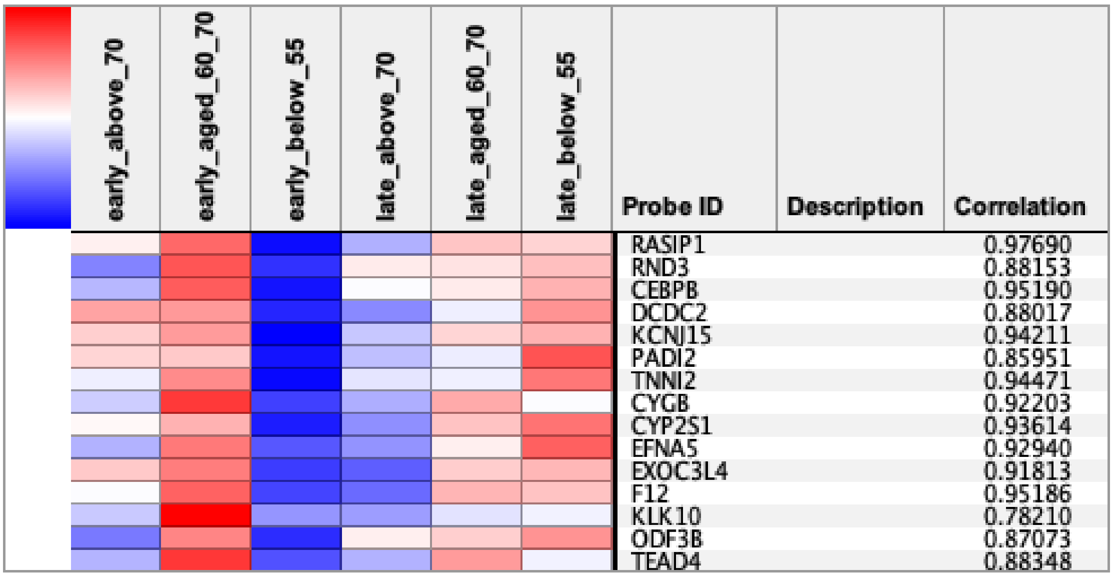

early_above_70
early_aged_60_70
early_below_55
late_above_70
late_aged_60_70
late_below_55
Probe ID
Description
Correlation
RASIP1 0.97690
RND3 0.88153
CEBPB 0.95190
DCDC2 0.88017
KCNJ15 0.94211
PADI2 0.85951
TNNI2 0.94471
CYGB 0.92203
CYP2S1 0.93614
EFNA5 0.92940
EXOC3L4 0.91813
F12 0.95186
KLK10 0.78210
ODF3B 0.87073
TEAD4 0.88348

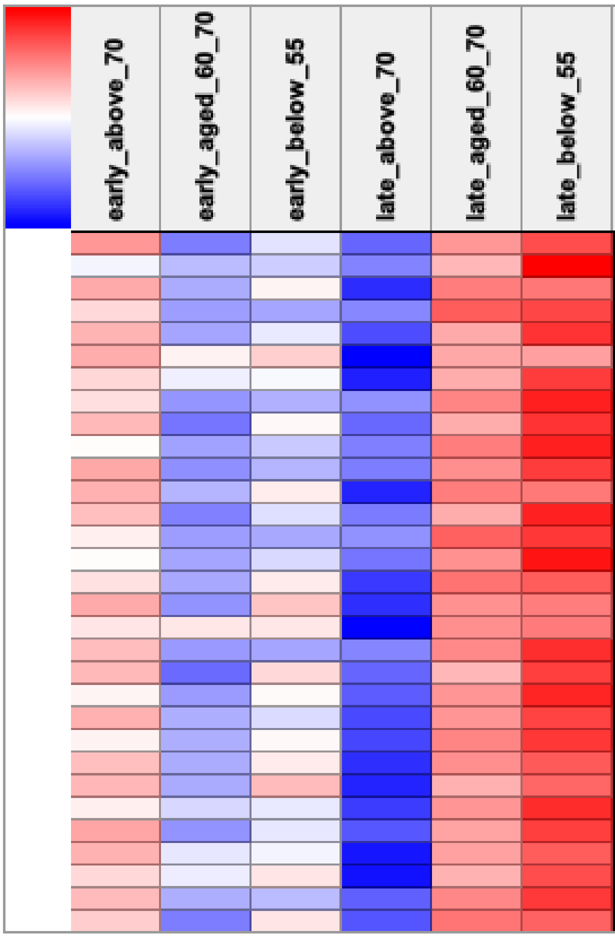

Probe ID	Description	Correlation
AFAP1L1		0.96984
ANKRD50		0.91692
CASP8		0.97019
ELK3		0.93472
FAM126B		0.99233
FARP2		0.83635
MALL		0.96036
NCOA3		0.93938
SCAF11		0.97212
SLC41A2		0.95590
TANC1		0.95569
XYLT1		0.96595
ZFAT		0.96876
RNF139		0.92358
ACVR1		0.96455
ALMS1		0.97996
ATP11B		0.94024
FBP2		0.87678
MOSPD2		0.94805
RAB30		0.94907
RPS6KA5		0.98163
TEK		0.99338
TMTC1		0.98710
WDR20		0.98605
ZNF644		0.94046
IGSF3		0.97785
DNASE1L1		0.98682
EMCN		0.95860
PEX11A		0.94180
SMAD1		0.98018
TLK1		0.96997

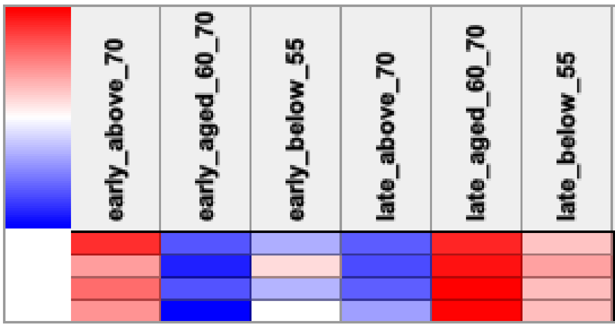

Probe ID	Description	Correlation
DDX60		0.96955
CAMK2D		0.97381
PAG1		0.98786
RBM12B		0.97446

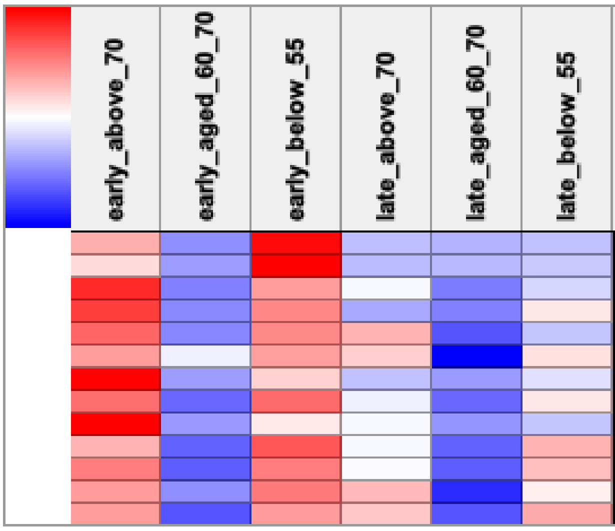

Probe ID	Description	Correlation
CD9		0.83762
EAF2		0.75372
FDFT1		0.95725
HMGCR		0.94055
MCCC2		0.93693
NOL4		0.82066
TNFRSF10A		0.84778
ADCYAP1R1		0.99280
CMAS		0.82415
CNTNAP3		0.92883
FREM2		0.96903
MAOB		0.93145
TAS1R1		0.89848

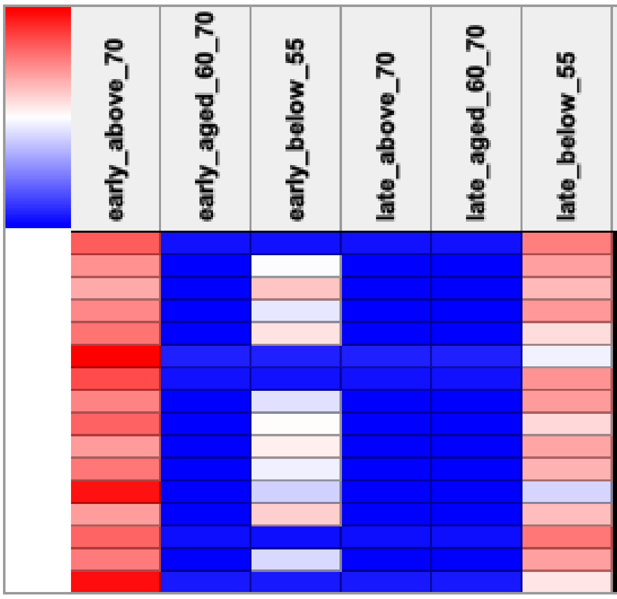

Probe ID	Description	Correlation
GAS2L3		0.91267
GRIK4		0.97929
LGR5		0.93907
MTTP		0.98749
TP53TG5		0.96770
ARHGAP36		0.87656
ATP2B2		0.91643
GRM4		0.99014
HS3ST3B1		0.98139
IL1RL1		0.96791
LILRA1		0.98956
MAEL		0.94646
RGS20		0.94973
UPB1		0.91019
KCNT2		0.99383
LDLRAD1		0.90053

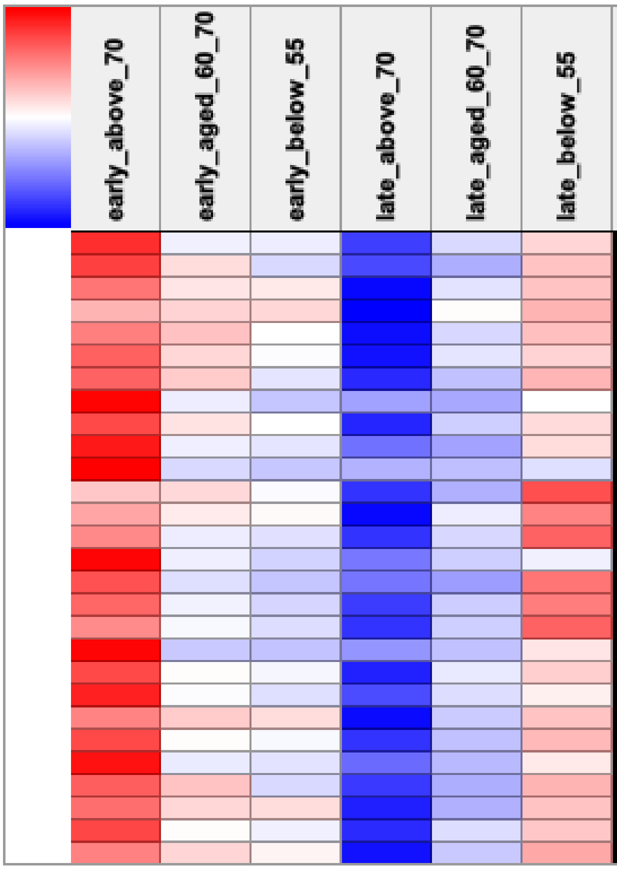

Probe ID	Description	Correlation
BCAP29		0.98391
CYB561D2		0.98590
ERRFI1		0.96446
FURIN		0.86694
INPP5K		0.95817
KLHL22		0.97604
MAN2B1		0.98541
NR4A2		0.84861
PDK2		0.98838
SLC1A4		0.94538
SLC24A1		0.77937
TNFRSF21		0.83390
USP3		0.91232
SLC9A1		0.92510
SND1		0.89480
AMOT		0.91400
CITED1		0.96538
LRRIQ1		0.92842
MORC4		0.86934
N4BP1		0.98782
PSTPIP2		0.96329
PXMP4		0.94582
SGPP1		0.99859
CYB5D2		0.94360
LMF2		0.97615
LSR		0.96490
PREB		0.99487
STK40		0.96552

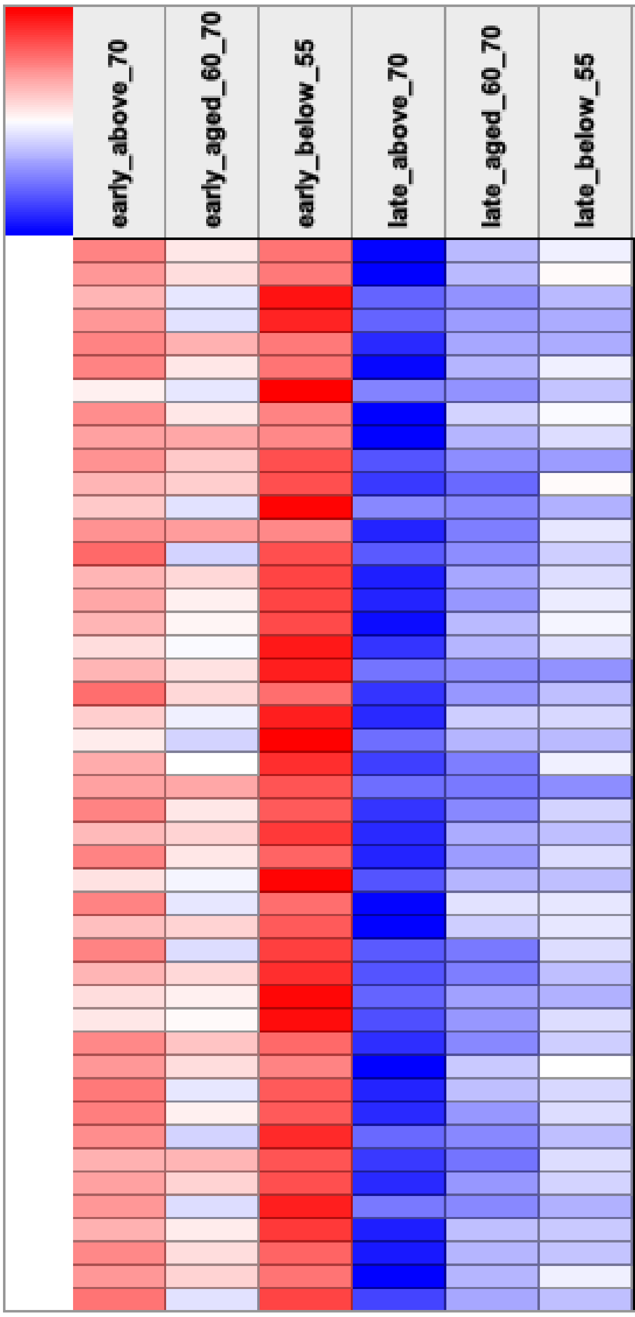

Probe ID	Descripti...	Correlation
DCBLD2		0.96485
DERL2		0.95394
DNAJB5		0.96387
GPR161		0.96502
LGR6		0.95106
ME3		0.96898
NUDT15		0.89750
RCBTB2		0.93817
ST3GAL3		0.93518
TEX14		0.97069
UCHL3		0.96590
VILL		0.92234
AGTRAP		0.93188
PAK1IP1		0.95512
PARP6		0.99397
PDIA3		0.99468
ADPRH		0.97919
ANGPT1		0.96513
ANGPTL1		0.94877
ANO4		0.97545
BEST3		0.96723
CAP2		0.90248
CPE		0.98524
DPT		0.92258
FAM124A		0.99010
FLNC		0.99036
GMPR		0.98785
HGD		0.95037
MYLK4		0.94246
OGN		0.96271
PDIA5		0.96759
PGC		0.98147
RAB9B		0.95045
TMEFF2		0.95449
TPD52L1		0.97819
TSPAN9		0.94023
FAT3		0.97326
CCDC125		0.98901
GPT2		0.95785
KRT14		0.97248
PPP1R7		0.99586
SEC14L2		0.94936
SH3BGRL2		0.99023
SLC25A42		0.98438
SPAG7		0.96406
TMEM220		0.97573

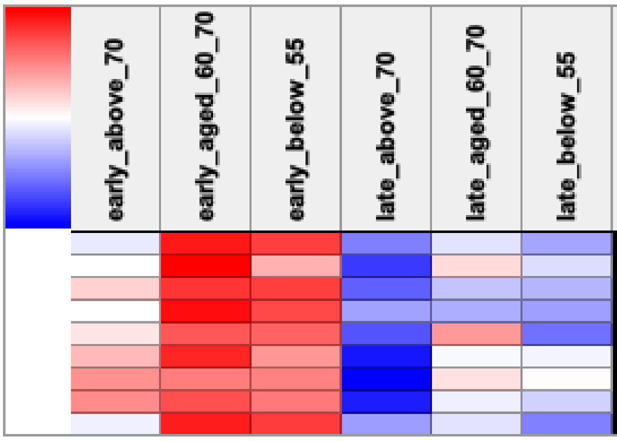

Probe ID	Description	Correlation
ECHDC2		0.96084
EMILIN1		0.93996
GSTM5		0.97875
SH2D3A		0.92427
TMEM44		0.91125
DUOX1		0.95525
PHF21B		0.88045
TRPC4		0.95241
LIMS2		0.93304

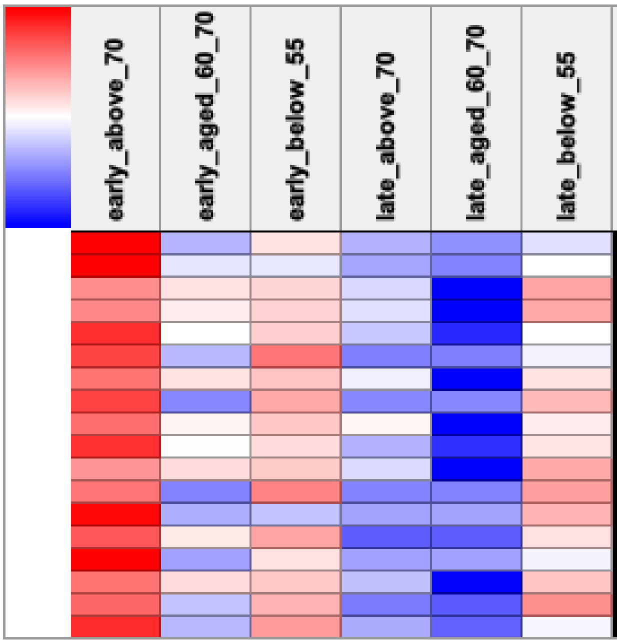

Probe ID	Description	Correlation
DDAH1		0.89773
DNAH6		0.90019
SEMA3A		0.89784
ARL10		0.90708
GABRB3		0.97603
ALG5		0.90627
EYA1		0.91616
FLG		0.91134
GNRHR		0.90108
MEGF10		0.98723
PPP4R4		0.88596
PXDNL		0.86145
RCAN3		0.82866
SLC26A3		0.93398
SLC6A3		0.88310
TTC22		0.93937
ACOT1		0.93984
SPINT2		0.95810

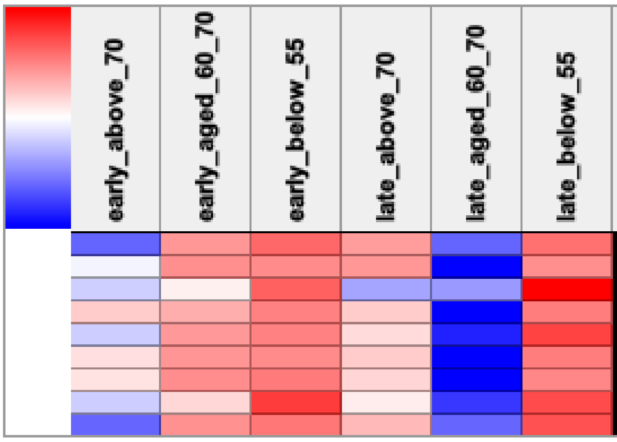

Probe ID	Description	Correlation
SEZ6		0.90274
PTPRZ1		0.95325
QPCT		0.78500
RAB40AL		0.94029
SLC44A5		0.99510
TTBK1		0.95333
ZNF711		0.95552
KCNG3		0.97158
ZFR2		0.91670

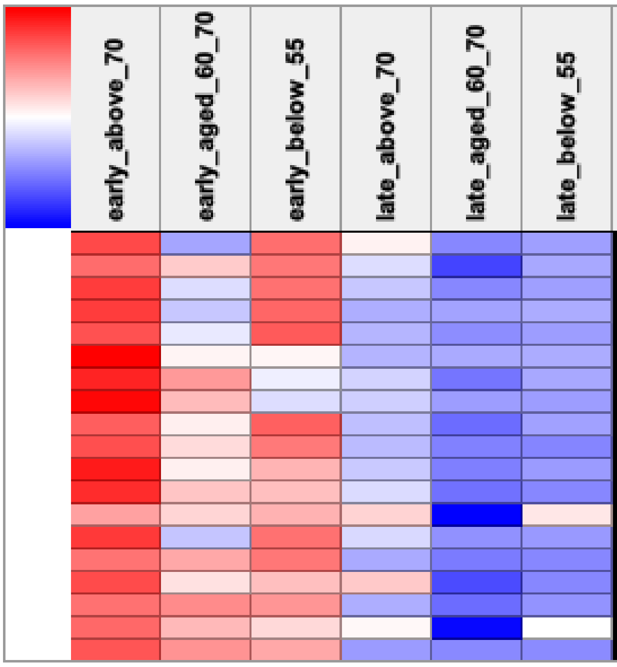

Probe ID	Description	Correlation
AGPAT5		0.87160
BCL2L14		0.96976
GSR		0.95754
HNMT		0.90985
PDE4D		0.94317
TMEM53		0.89279
ZFP36		0.88259
FOSB		0.85436
CACNG4		0.97137
CHRNA2		0.98070
FBXO25		0.98651
SORBS3		0.98258
TMEM132B		0.73386
ADH5		0.93229
DCXR		0.94143
EPS8L1		0.92149
PACSIN3		0.93267
SERPINB5		0.83827
TRADD		0.93432

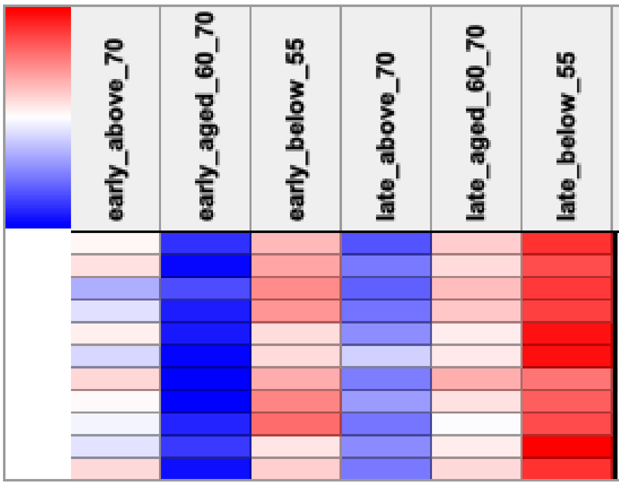

Probe ID	Description	Correlation
ITGB1		0.98782
LRRC40		0.99082
MOB4		0.95126
PPP3CA		0.99150
PRKAA1		0.98242
RBM18		0.95065
SENP7		0.96354
MBLAC2		0.97908
LYSMD3		0.97139
RAB2A		0.96365
RP2		0.98706

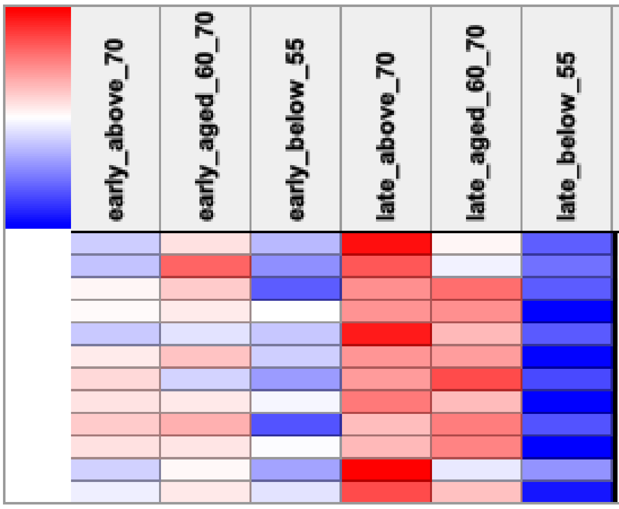

Probe ID	Description	Correlation
RPS12		0.90570
FDXR		0.80925
HTR2B		0.91177
LIN7A		0.93532
NEK2		0.92142
NPC1L1		0.96423
PDE1A		0.87604
TNFRSF8		0.94935
TRIM67		0.85150
AMZ1		0.89542
RPS6KB2		0.79893
TTC16		0.97738

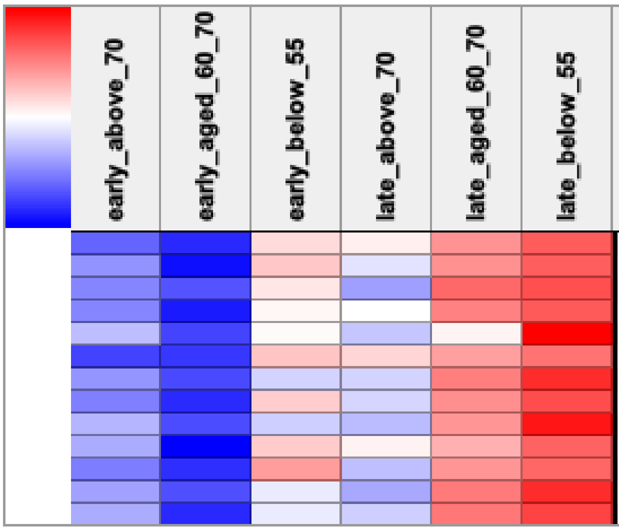

Probe ID	Description	Correlation
C16orf87		0.97467
C1orf109		0.98757
GABPA		0.96960
KRR1		0.98762
PHLDB2		0.91260
UCHL5		0.92795
BCOR		0.97265
ACTR3		0.99390
GDPD1		0.95362
ZNF254		0.95833
NAALADL2		0.96051
PDIK1L		0.96853
SP3		0.98400

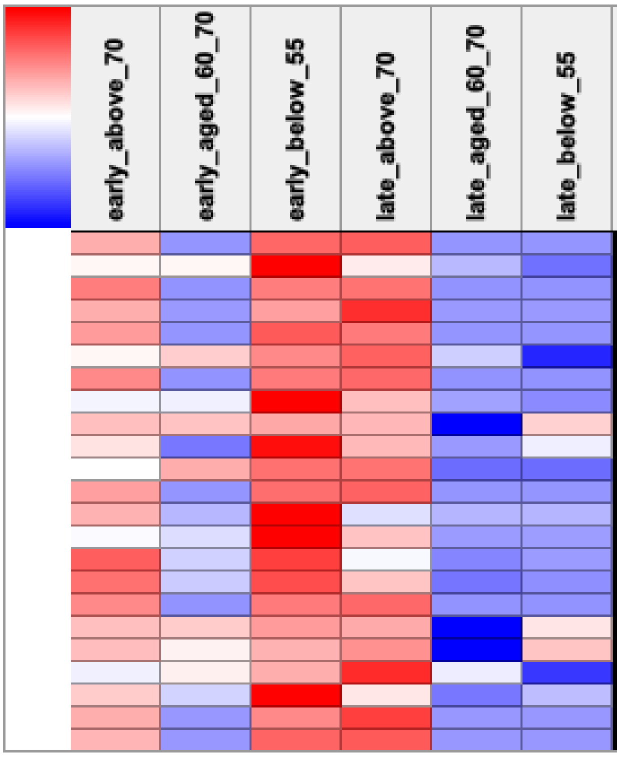

Probe ID	Description	Correlation
ASCL1		0.96455
EPHB1		0.83349
GCNT3		0.94387
HOXB8		0.89948
KIRREL3		0.97255
LFNG		0.77122
PTCD1		0.94881
RSPO3		0.89081
ZNF391		0.64512
FADS2		0.86526
CDH7		0.83771
LRRTM2		0.96089
ODC1		0.80094
PI15		0.90154
SEMA3D		0.85995
SEMA6A		0.94981
SLC16A12		0.94836
STAP1		0.71387
WFDC3		0.68591
LOC10050...		0.69792
PPP2R2A		0.90934
PRDM12		0.92911
ULBP2		0.96436

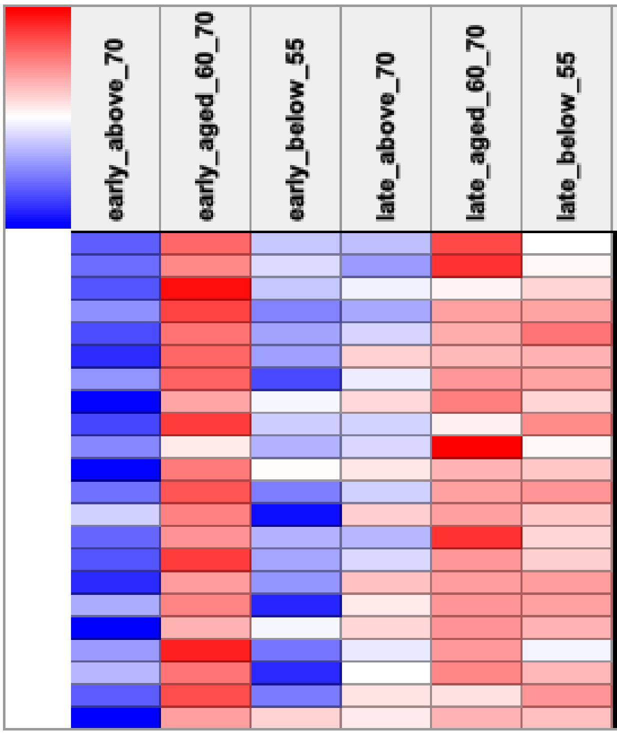

Probe ID	Description	Correlation
CAPN3		0.91742
CNIH3		0.85304
NNAT		0.87946
PDLIM1		0.92815
PSD		0.96064
SMARCD3		0.96155
PCOLCE		0.93680
DKK3		0.87505
GATA3		0.89752
HUNK		0.74086
JPH3		0.88119
IGFBP4		0.97142
ARID5A		0.77180
CLEC11A		0.91319
EFEMP2		0.98272
MSX1		0.93683
MUC1		0.86579
S100A11		0.86588
SRRM3		0.89527
TBXA2R		0.87035
TMEM88		0.93080
TPRG1		0.79516

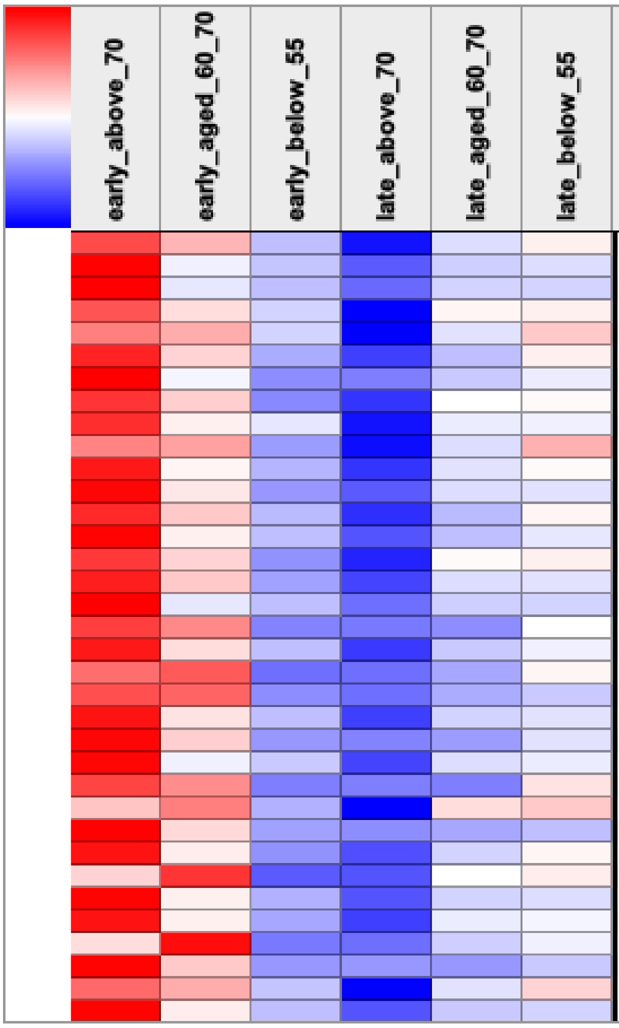

Probe ID	Description	Correlation
ALPK1		0.97800
CD82		0.94990
DLG2		0.92941
HOOK2		0.93261
ITGA3		0.90215
LRFN3		0.99457
MAST4		0.94284
PLXNA2		0.98206
RPTOR		0.94935
TG		0.90512
TNRC6C		0.98043
TTC7A		0.98126
VPS18		0.99333
THSD4		0.97334
TLN2		0.97548
ACAD10		0.99533
ATP8B2		0.92516
CXCL6		0.93387
CYP46A1		0.99015
ICAM4		0.88666
LTF		0.91560
MPPED2		0.98523
PROM1		0.95539
SMG6		0.95712
TMEM132D		0.91558
CAPN13		0.77691
BDH2		0.93172
CDH3		0.98578
CX3CL1		0.70785
ERCC2		0.97466
FOSL2		0.98189
PIGR		0.66109
RNF39		0.92826
S100A1		0.94116
SPTLC3		0.97012

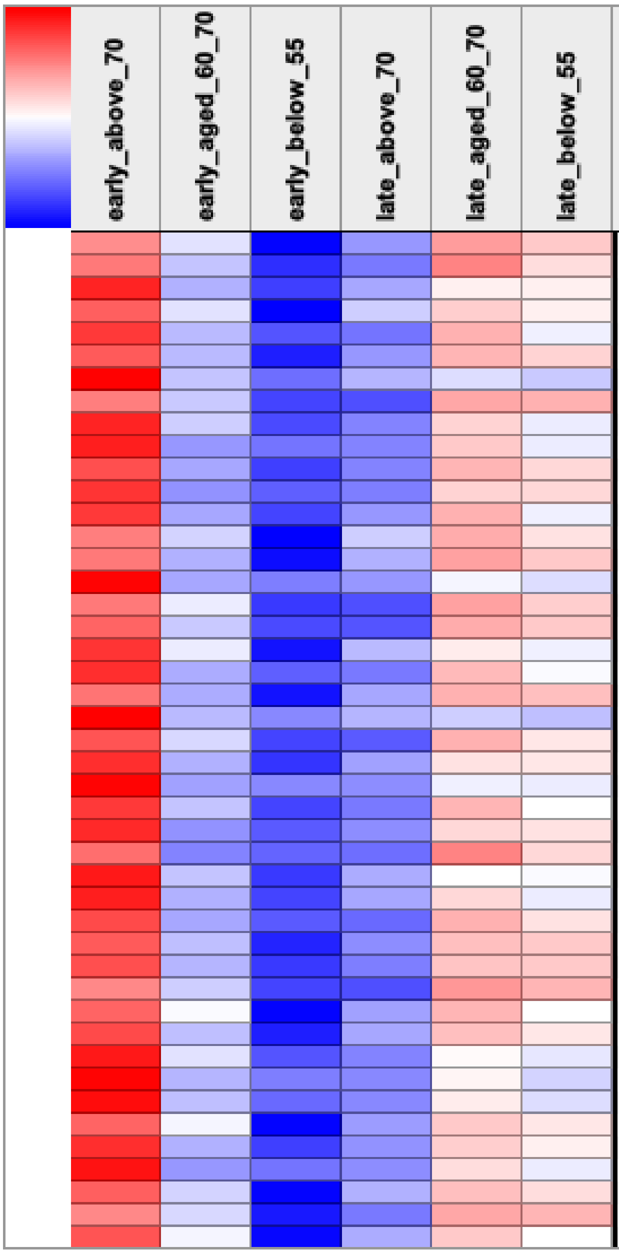

Probe ID	Description	Correlation
ARNTL2		0.92814
CAD		0.95566
CHD4		0.98224
DUSP6		0.94354
EPS8		0.98585
ITGAL		0.98611
KLF6		0.89042
MEF2C		0.93710
MNT		0.98713
NDST1		0.96421
PLD1		0.99104
PRRC2B		0.97778
RAPGEF5		0.98914
SHQ1		0.92718
SLCO2B1		0.95017
TRAM2		0.92050
CD55		0.94169
SIRPB2		0.96521
AMPD2		0.95767
BACH2		0.98620
DOCK2		0.95834
DYRK3		0.84228
F13A1		0.97789
IL2RB		0.99088
NTRK2		0.91870
PHACTR1		0.99465
PIK3CG		0.98028
PREX2		0.92706
RPGRIP1		0.96889
TRAF3		0.98289
TULP4		0.98295
WDFY4		0.98765
GXYLT2		0.98995
PEAK1		0.91908
ACAP1		0.95522
ARHGAP30		0.99130
CYB561D1		0.96051
HCFC1		0.93112
IP6K1		0.95835
NPR1		0.96282
PPP1R16B		0.99718
SETD1B		0.95742
TMC8		0.97155
TMEM200A		0.94032
ZMYND15		0.96528

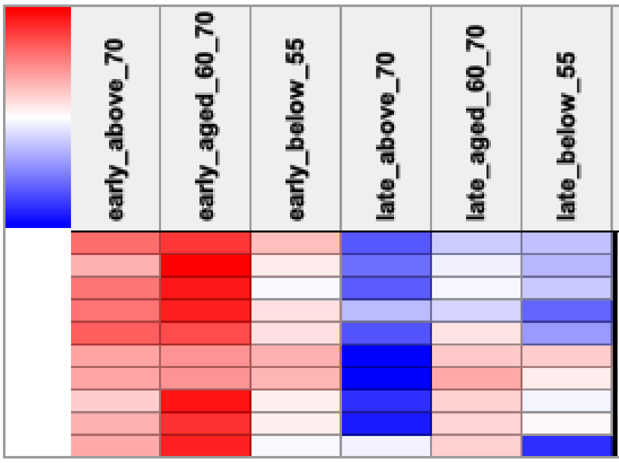

Probe ID	Description	Correlation
ACCS		0.96023
FAIM2		0.96780
FBLN1		0.98006
AEN		0.86678
EDA2R		0.97508
LRP4		0.82680
PSD2		0.84466
TCF21		0.95368
VWA5A		0.95288
CXXC1		0.72751

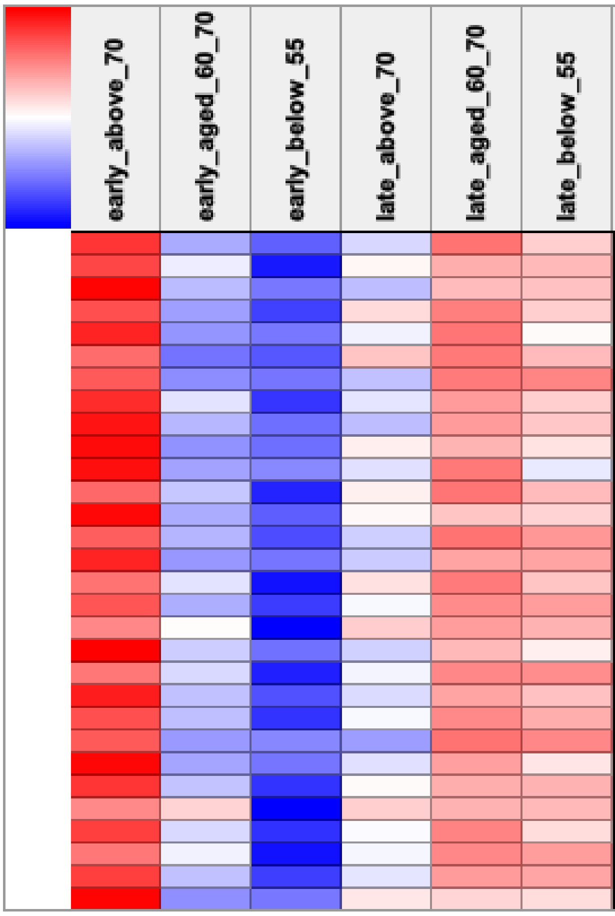

Probe ID	Description	Correlation
ARID3A		0.98429
CPVL		0.96984
KIAA1328		0.95158
MCCC1		0.97641
MDC1		0.95584
PTPDC1		0.92809
SSH1		0.94106
STK10		0.98638
TUFT1		0.96983
ZC3HAV1		0.95534
SLIT3		0.92057
BCL11B		0.97527
CBX4		0.96704
CD84		0.97354
DOCK11		0.96762
FMO3		0.95048
GBP5		0.98824
HCK		0.89364
HECA		0.94282
IL10RA		0.95509
PLXNC1		0.99140
PRICKLE1		0.99394
SESN3		0.90716
SLC31A2		0.96522
WNT5A		0.99093
AGBL4		0.83920
ADCY9		0.98584
ITGB2		0.94577
NCKAP1L		0.99455
RAB3A		0.92729

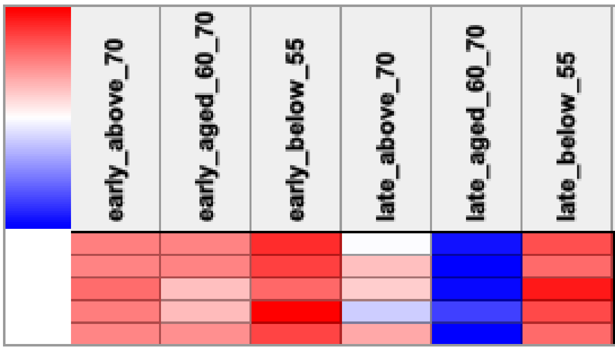

Probe ID	Description	Correlation
MYO18B		0.99296
MACROD2		0.98853
PTPRT		0.97568
ABO		0.94905
NEK10		0.98003

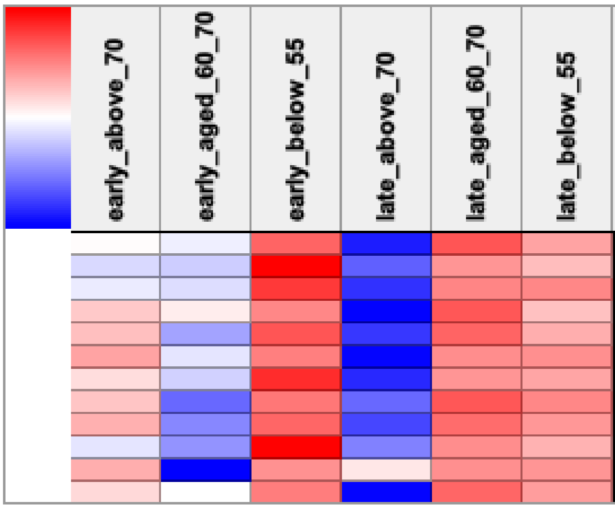

Probe ID	Description	Correlation
GPATCH1		0.96550
HOMER2		0.91988
MTO1		0.96808
PALLD		0.91263
ZNF549		0.99322
DOCK1		0.94266
EPHA7		0.98197
SCN3A		0.95252
CEP290		0.97833
INPP5F		0.92521
NME9		0.57790
NUMA1		0.94450

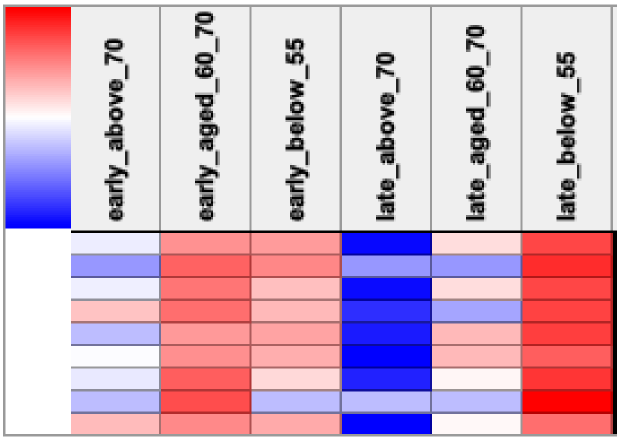

Probe ID	Description	Correlation
FAM171A1		0.97887
GPR55		0.85371
NR1H2		0.98869
NTN1		0.93390
PDE4A		0.95331
TSC22D1		0.95173
DLL1		0.99370
BMP3		0.76919
ST18		0.93473

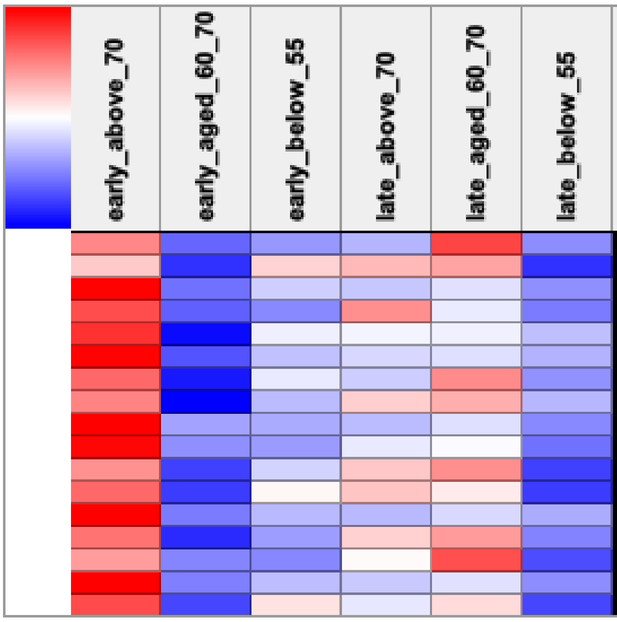

Probe ID	Description	Correlation
CDC14A		0.82687
KLRD1		0.78058
MFHAS1		0.92331
MOV10L1		0.88057
MYO1B		0.92621
PDPR		0.92509
RALBP1		0.93430
SLC36A1		0.90258
L3MBTL3		0.87464
ARL9		0.93623
DIO1		0.90849
KCNMB2		0.92709
PLD6		0.88642
RTN4RL1		0.94431
VSTM2L		0.82228
WDR55		0.91712
WNT8B		0.94560

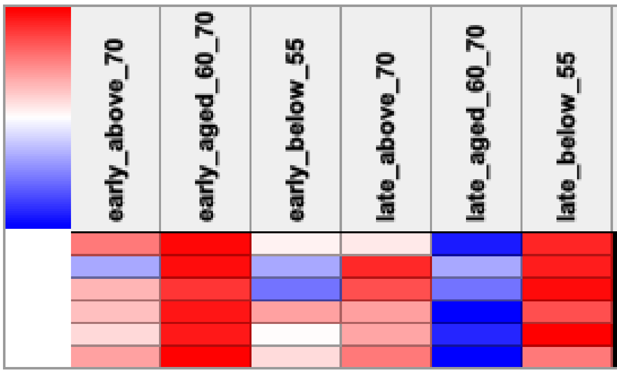

Probe ID	Description	Correlation
CEACAM6		0.93580
TRPM3		0.83295
ABCA12		0.91197
RGS13		0.93732
DKK1		0.98689
S100A14		0.95461

early_above_70	early_aged_60_70	early_below_55	late_above_70	late_aged_60_70	late_below_55	Probe ID	Description	Correlation
						PIH1D2		0.99497
						CLDN4		0.97210
						DUSP5		0.93823
						JUNB		0.96879
						RND1		0.86484
						SOCS3		0.98461
						SLC34A2		0.89763
						TRHDE		0.85184
						CXCL2		0.98250
						RELB		0.86316

early_above_70	early_aged_60_70	early_below_55	late_above_70	late_aged_60_70	late_below_55	Probe ID	Description	Correlation
						BAMBI		0.97502
						CDV3		0.93700
						LY75		0.97172
						RAB11FIP4		0.96808
						RAD18		0.92988
						SNX24		0.91162
						TMEM38B		0.91737
						EPB41L4B		0.87882
						MYH15		0.99661
						RGS4		0.94588
						SMCHD1		0.90751
						STON2		0.96737
						TGFBR1		0.96443
						IGSF21		0.82382
						AIF1L		0.94173
						BSN		0.91192
						JPH1		0.93555
						SH2D2A		0.80704
						TM7SF2		0.90814

early_above_70	early_aged_60_70	early_below_55	late_above_70	late_aged_60_70	late_below_55	Probe ID	Description	Correlation
						ADCY2		0.91929
						CNTNAP1		0.96023
						LTBP1		0.91537
						PLA2G4A		0.97734
						SERPINE1		0.94504
						RUNX1		0.96192
						CAMK4		0.96456
						IL4R		0.90703
						MREG		0.98510
						RIN3		0.94457
						TSPAN7		0.95567
						GDPD5		0.88312
						PLVAP		0.97592
						BTN3A2		0.94415
						KCNIP4		0.97948
						LAMA2		0.93002
						PPM1N		0.85631

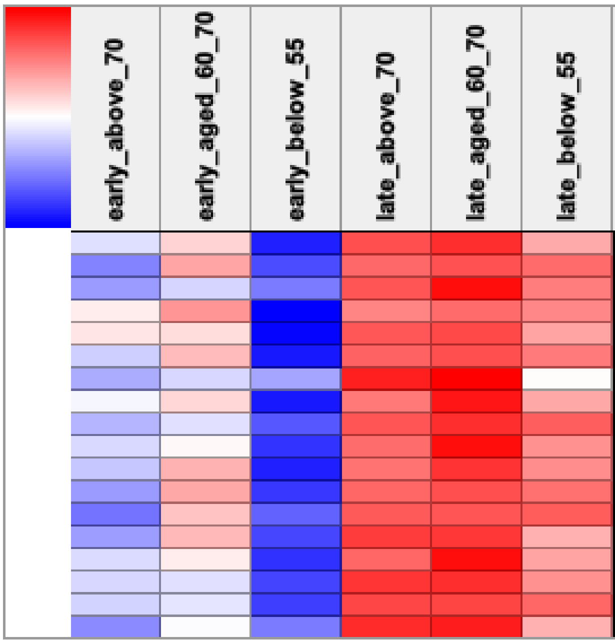

Probe ID	Description	Correlation
FGD3		0.98952
LYL1		0.95631
PPP2R2B		0.95055
SERPINB1		0.90597
SKAP1		0.95038
UNC13D		0.98249
CEP152		0.85941
COL5A3		0.97313
SFRP4		0.97048
SH3BP2		0.98898
VAX2		0.98327
TSPAN4		0.96976
MYO1H		0.94846
BST2		0.98029
DOK1		0.98895
DOK3		0.97776
HPSE		0.97516
WDR90		0.95477

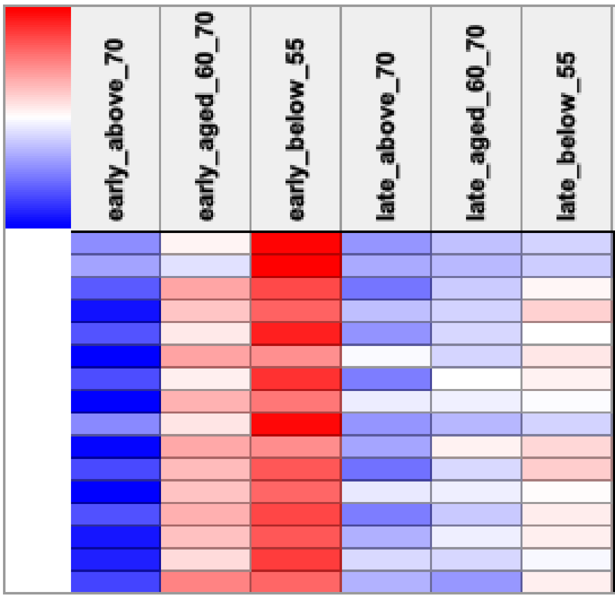

Probe ID	Description	Correlation
KCNN4		0.91065
RFPL2		0.84126
WNT5B		0.97144
HTATIP2		0.97638
ACSBG1		0.98026
ANKRD22		0.90016
BIVM		0.96486
ISM2		0.93500
NECAB1		0.92035
SYNDIG1		0.93068
MMP15		0.96598
CRYAB		0.95023
CTF1		0.98122
DLGAP1		0.98853
OGDHL		0.98050
SERF2		0.94332

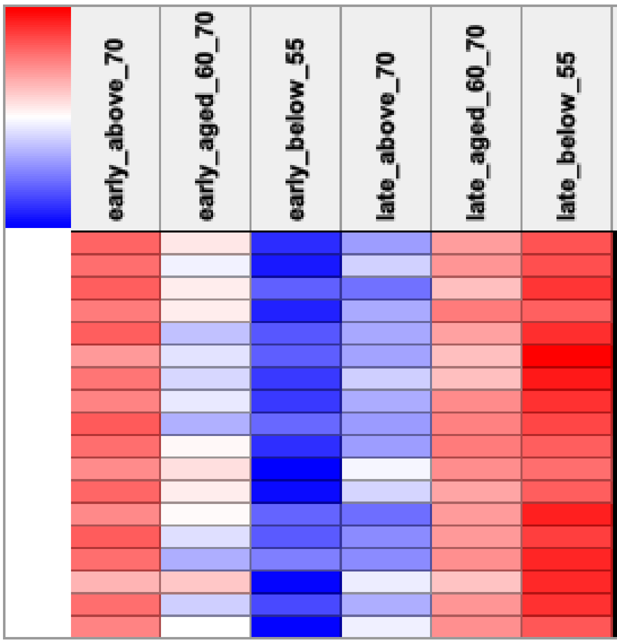

Probe ID	Description	Correlation
CCDC80		0.98795
DLL4		0.98918
ENPP2		0.96388
GRB10		0.98288
PRDM1		0.98179
SOX6		0.96331
CAPN2		0.98163
ADAMTS12		0.99698
ANKRD44		0.96176
CCR7		0.98712
CD33		0.93727
CD74		0.97544
MAP1B		0.96807
NLRC4		0.98495
TTC39B		0.95046
C15orf39		0.92445
FOXP3		0.99249
MB21D2		0.96495

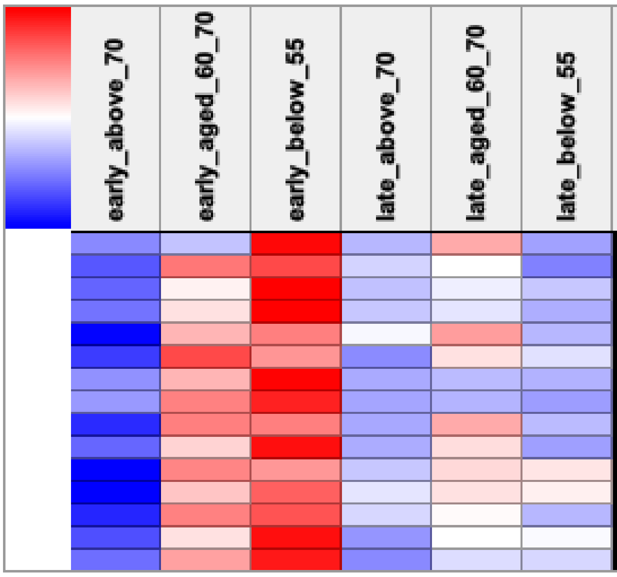

Probe ID	Description	Correlation
CHN2		0.82425
PPP1R1B		0.95400
AGR2		0.94776
CAV1		0.94708
CYP2E1		0.88827
LPAR6		0.88506
P2RX1		0.92292
RND2		0.90201
TMPRSS11E		0.92770
IRX4		0.97321
CXXC4		0.86776
EIF3I		0.91423
PCP2		0.97406
PDE9A		0.95610
SLC16A5		0.96331

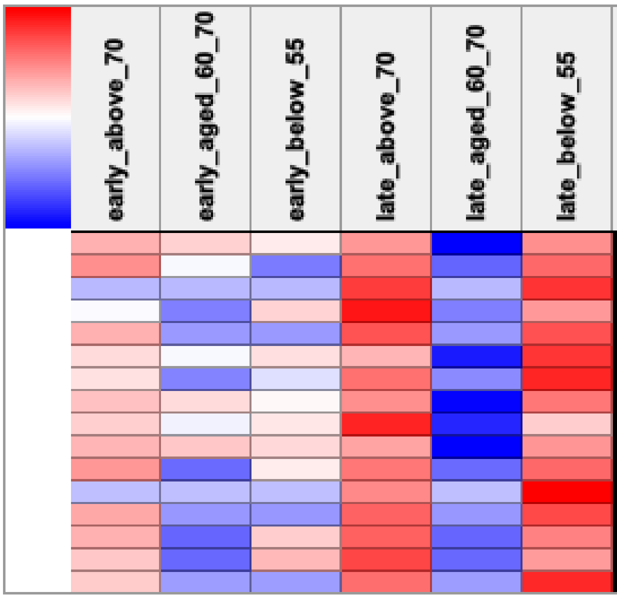

Probe ID	Description	Correlation
CXCL13		0.83841
GOLT1A		0.88459
BDKRB1		0.84043
CCDC150		0.88528
EDAR		0.92849
FAM171B		0.90108
ITM2A		0.95198
MYO3B		0.88342
PTGFR		0.87041
SIRPG		0.80375
TNFAIP6		0.93681
TNN		0.80268
FGF9		0.92498
FOXH1		0.92203
SLC39A5		0.89062
TRIM54		0.92416

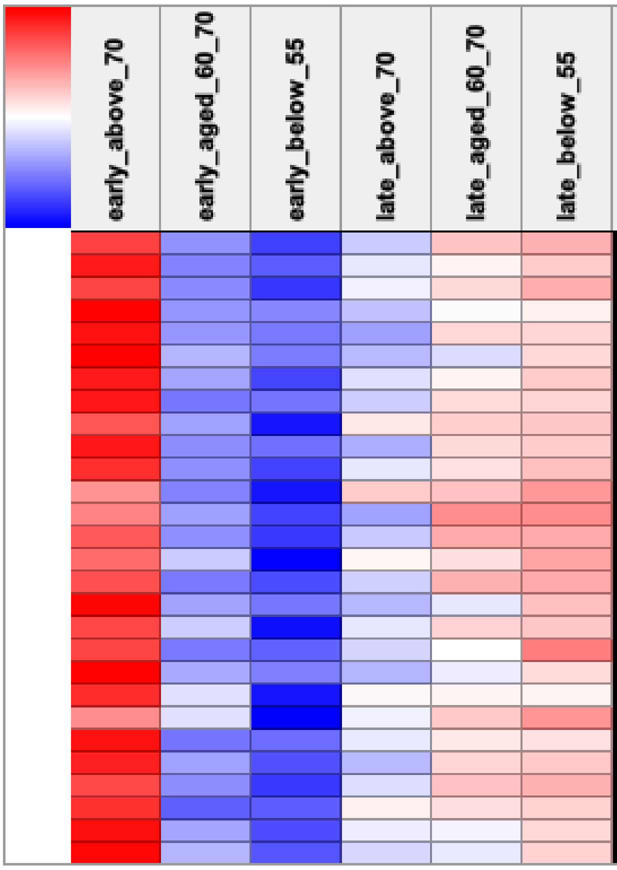

Probe ID	Description	Correlation
C12orf49		0.99157
CAMSAP1		0.99013
LASP1		0.99146
MAGI3		0.94093
MAP3K1		0.96062
MAP3K9		0.93592
OXCT1		0.99443
STK35		0.97684
TFRC		0.96065
TTF2		0.97576
ZMIZ1		0.99884
FGD6		0.88170
ITGA4		0.89810
RAPGEF1		0.96942
RBMS2		0.93284
SKIL		0.97412
TNFAIP3		0.95799
WNT11		0.96799
NOXO1		0.96201
PIGX		0.94485
IL4I1		0.93443
KBTBD8		0.88328
MMGT1		0.97487
RASGRP1		0.99125
SLC6A9		0.98885
TDRD7		0.96449
TRIB1		0.98038
ZBTB7B		0.97124

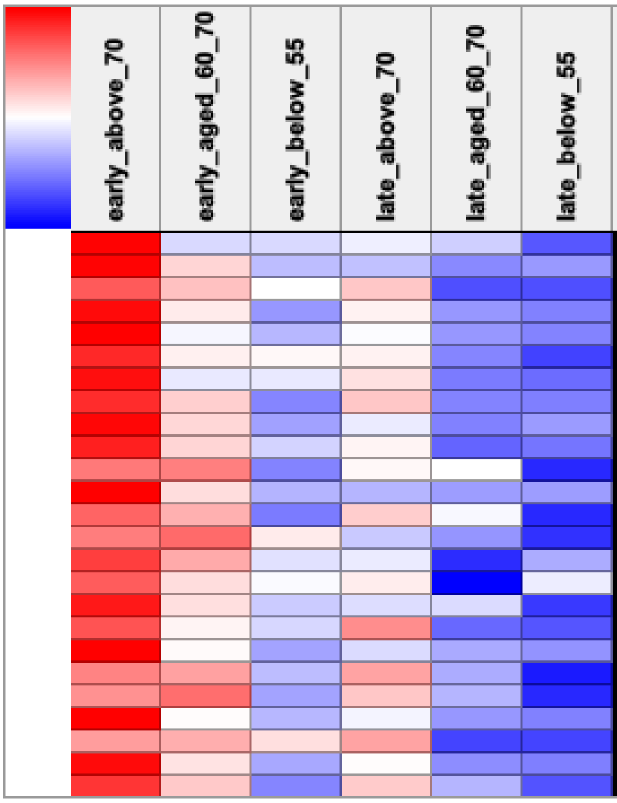

Probe ID	Description	Correlation
CCNG1		0.91409
COPZ2		0.94394
DLGAP3		0.93724
EGR1		0.97557
TNFRSF10B		0.96255
TP53		0.96204
TUBB2B		0.95720
IER2		0.96710
ATF3		0.96490
FOS		0.98866
SLC5A10		0.82222
EGR3		0.92482
IMPDH2		0.86162
NRTN		0.83819
PDLIM4		0.90595
CT62		0.75457
EFNB2		0.93986
F2RL1		0.91928
HBEGF		0.94055
JUN		0.88750
NMB		0.85806
POLD4		0.96880
SIT1		0.81569
TST		0.98510
VPS37B		0.96088

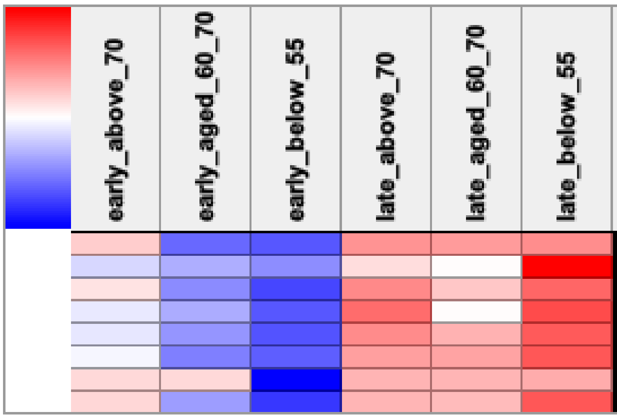

Probe ID	Description	Correlation
DYRK2		0.94561
SAT1		0.86557
CHAF1A		0.99177
PCDHB4		0.95961
BPGM		0.98850
PARD6B		0.98319
PLAU		0.81611
SLC9A6		0.98889

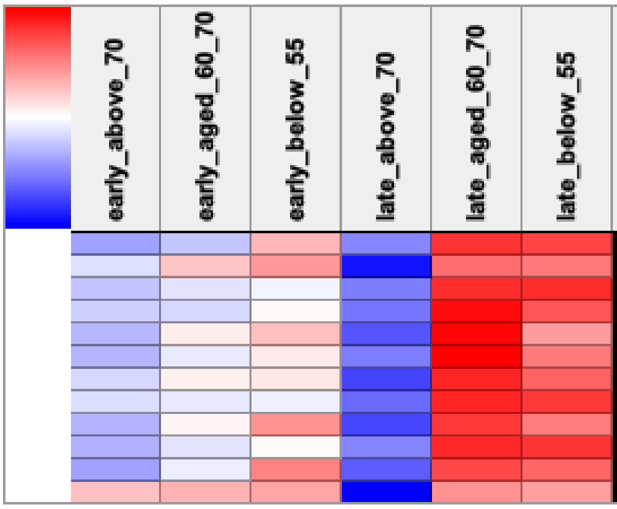

Probe ID	Description	Correlation
KITLG		0.95908
SPATS2L		0.92517
CAMK1D		0.95860
ADAMTSL3		0.97422
ANK2		0.97210
DLX5		0.96886
KCNK13		0.99274
MARK1		0.96377
PTK7		0.97406
SEMA5A		0.96480
CDC42SE1		0.96106
SLC35F3		0.78053

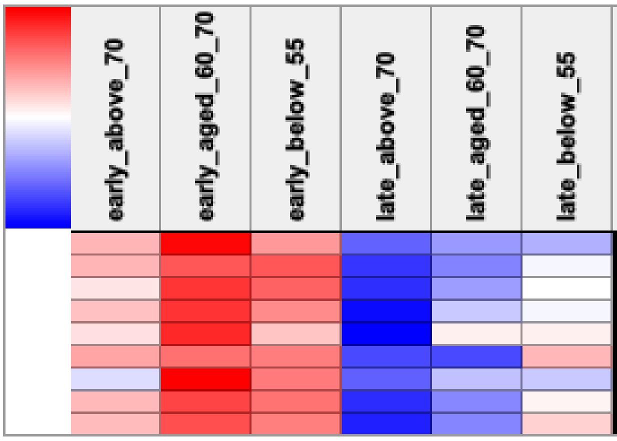

Probe ID	Description	Correlation
KLHDC4		0.94989
SH3RF2		0.98234
KRT8		0.99342
FKBP2		0.98691
COL17A1		0.90837
RNF175		0.92113
SFN		0.92404
MT1M		0.99351
NTRK1		0.98259

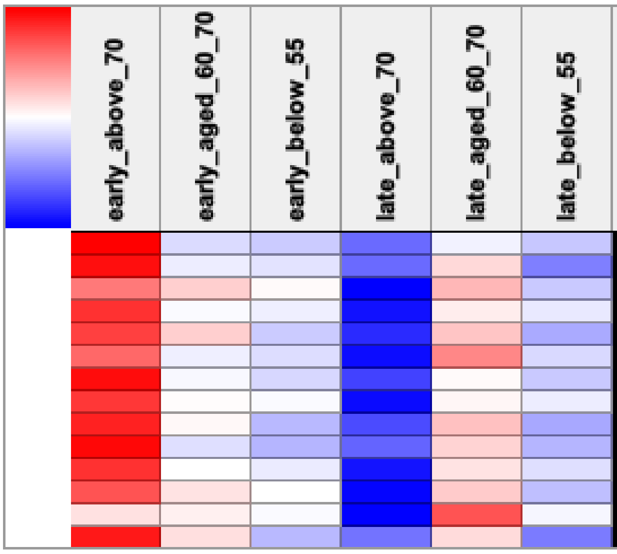

Probe ID	Description	Correlation
DLG5		0.92738
GLUL		0.94633
RIN2		0.94464
SARM1		0.98100
SGSM2		0.98438
ANKRD36		0.95471
ASAP3		0.97603
DSC1		0.97050
SYT2		0.98179
RAPH1		0.95698
ABCC10		0.98942
SCN5A		0.98097
SLC29A2		0.74274
SLC29A3		0.93232

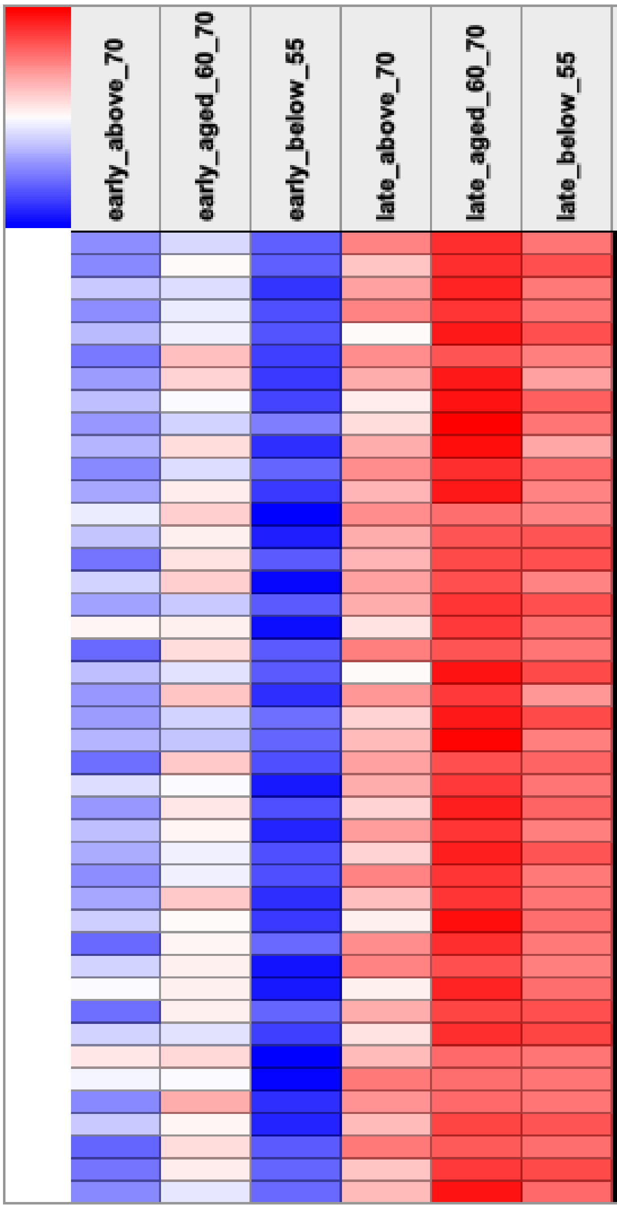

Probe ID	Description	Correlation
ABCC5		0.97271
ADAMTS2		0.98496
COL10A1		0.98460
COL1A1		0.98371
ELMO1		0.96475
FXYD5		0.96416
IFFO1		0.97623
ITGA11		0.97619
LOXL3		0.94458
MFGE8		0.97527
MMP11		0.97521
PAQR5		0.99364
SPI1		0.92807
RAP2B		0.98636
ABHD12		0.97405
ARHGAP4		0.96763
ASPN		0.97692
CD2		0.93987
CLEC2B		0.95740
COL4A2		0.95656
GLIS2		0.97420
GZMK		0.96625
IFI44L		0.96228
IL18		0.96714
KCNAB2		0.98116
LMO2		0.98922
LY9		0.99129
NOX4		0.98913
SLC17A9		0.98369
TGFB1		0.98855
VCAN		0.96959
WNT2B		0.96754
GMFG		0.97123
SVEP1		0.94856
NINJ2		0.97327
ENO2		0.96938
FSTL3		0.90789
HRH1		0.93103
IL34		0.95108
RHBDF2		0.98799
RTP4		0.95177
TMEM106C		0.97438
TPST1		0.98002

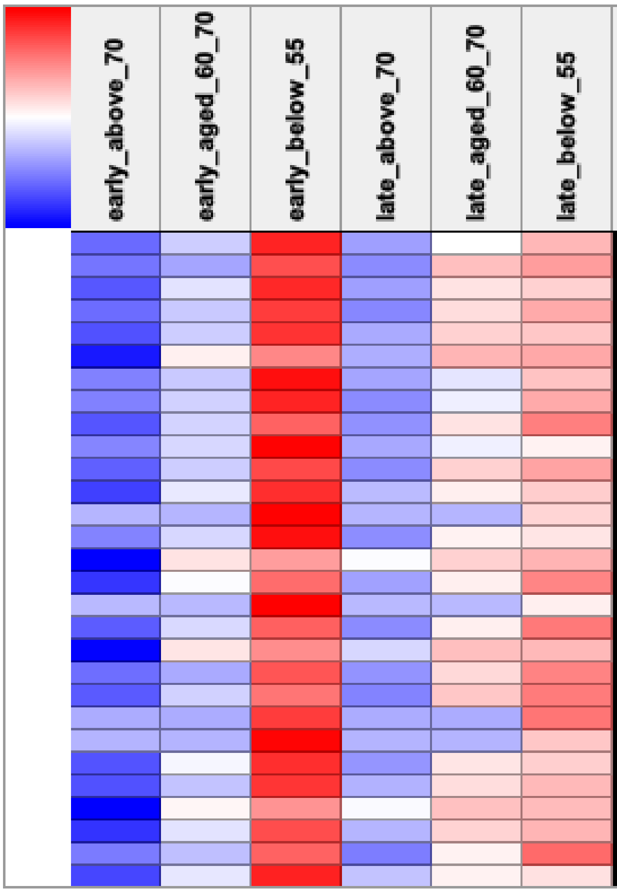

Probe ID	Description	Correlation
ALPK3		0.99339
CDK19		0.95782
ERG		0.98911
GULP1		0.98978
HDAC1		0.98979
LMTK3		0.90501
RAB11A		0.96821
SNX14		0.97879
WBP4		0.97513
CACNA1B		0.93990
CCNJ		0.98815
COX16		0.98537
EDN3		0.86828
FHL1		0.96044
FMN2		0.81150
KCNG1		0.96163
LIX1		0.84622
MTRF1		0.97128
PDE6A		0.86441
PECR		0.96500
PLA2G7		0.94884
SH3GL2		0.87053
UNC5D		0.87668
IL5RA		0.98419
NEB		0.99383
GSDMD		0.83406
NDRG1		0.98307
RGMB		0.94684
RGS9BP		0.97239

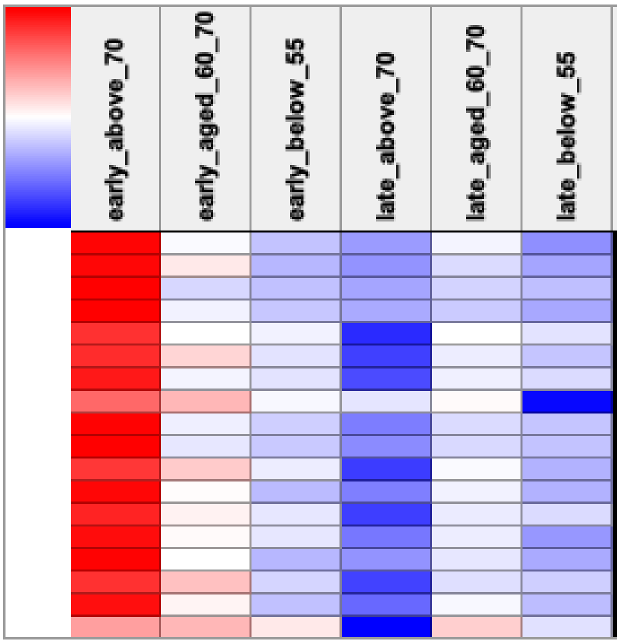

Probe ID	Description	Correlation
EXTL3		0.97286
FBXO31		0.98076
UNC5A		0.94846
ALDH3B2		0.95969
ARHGAP44		0.95291
CD99L2		0.98016
DAGLA		0.97945
IGFBP2		0.69546
IL7		0.98477
MMACHC		0.97597
PYROXD1		0.96926
RIMS3		0.99084
TRPM6		0.97649
WDR59		0.99277
BDH1		0.98031
MFSD5		0.96815
NOL9		0.99541
SLC24A3		0.73965

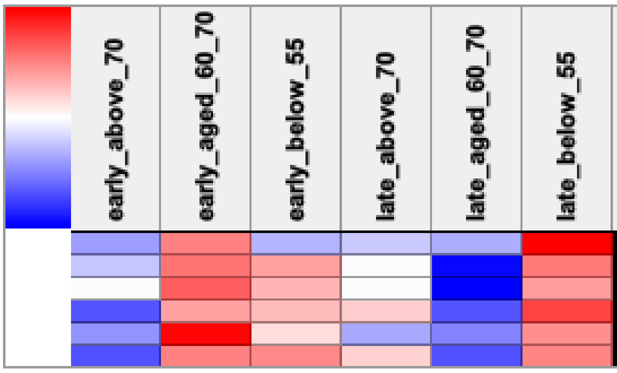

Probe ID	Description	Correlation
SECTM1		0.80559
ACTN2		0.96303
ALDH3A1		0.90078
RGS18		0.94136
SOD3		0.90436
GAL3ST3		0.93208

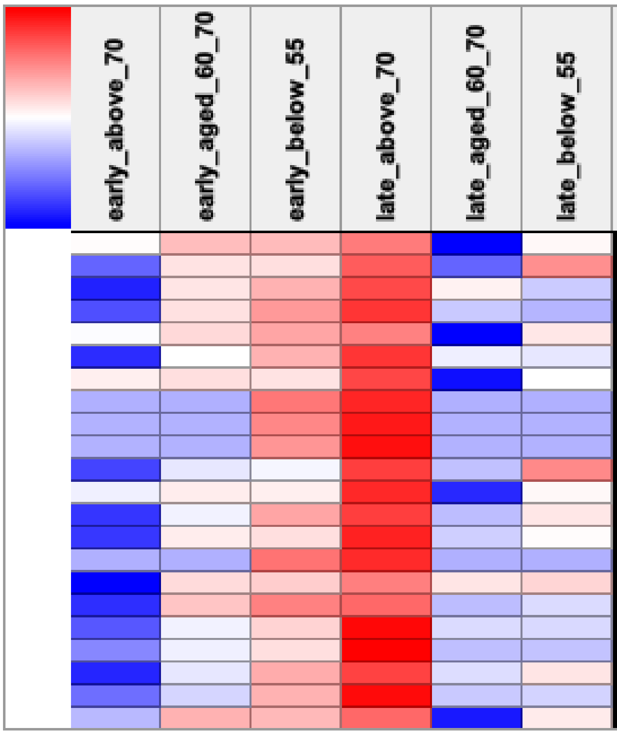

Probe ID	Description	Correlation
ACRV1		0.68853
CDH17		0.86065
NDUFA2		0.86699
NUTF2		0.94811
PACRG		0.70480
SHC3		0.92861
AMBP		0.73400
AMDHD1		0.89303
ART4		0.90270
CLDN10		0.90676
DMC1		0.82599
GAL3ST1		0.81320
KREMEN2		0.96005
METTL24		0.94728
NLRP2		0.89023
PSMB3		0.74738
TSPO		0.91212
C4orf47		0.95245
CTXN1		0.94878
GTF2F2		0.92534
RPS4Y1		0.96655
VSIG2		0.82479

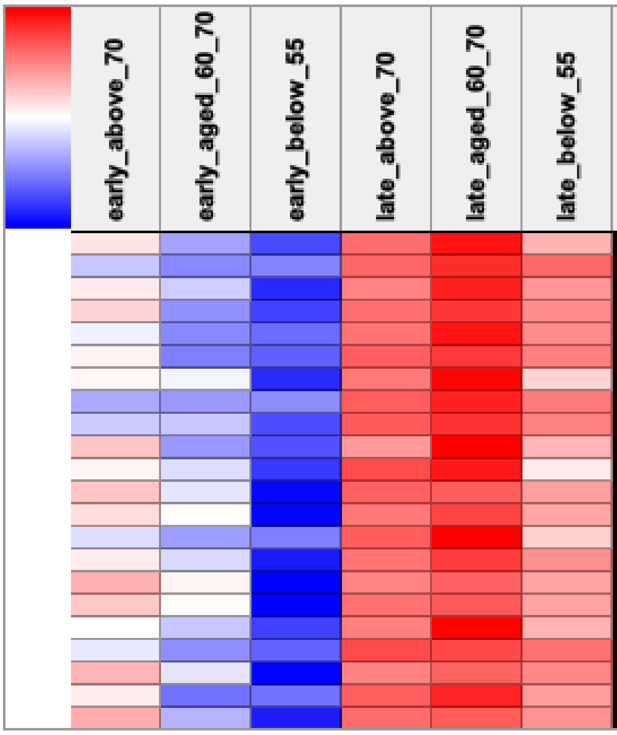

Probe ID	Description	Correlation
ADAMTS6		0.99256
CSPP1		0.93333
FANCE		0.99385
FRY		0.98705
MRPS28		0.97151
PHTF2		0.97392
PIWIL4		0.96328
PTPN12		0.91432
SLC29A1		0.97292
LBH		0.96425
ADAP1		0.96132
CD37		0.96427
CST7		0.96017
IFIT3		0.94229
MSR1		0.98991
SDS		0.91989
SNX8		0.94746
CPLX1		0.98822
DSN1		0.96416
IFI30		0.94901
OAS3		0.96455
TRIM69		0.96593

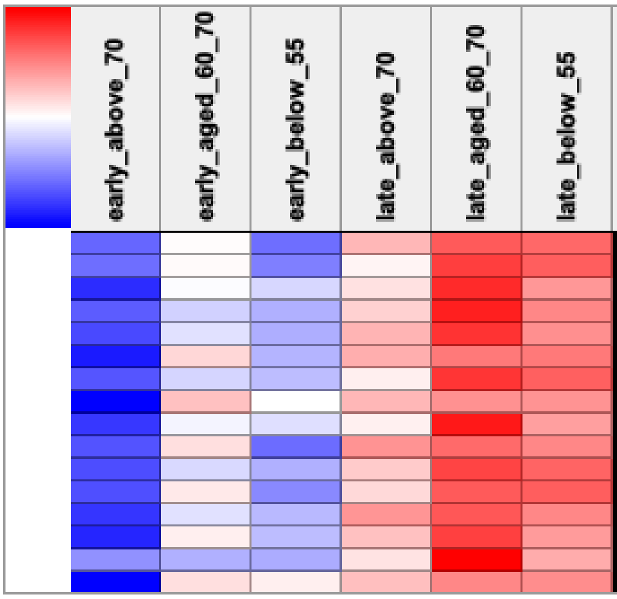

Probe ID	Description	Correlation
AJUBA		0.96186
GAS7		0.95828
SMAD6		0.98258
NUDT17		0.97980
CHN1		0.99074
EFNB3		0.96890
LURAP1L		0.97458
SLAMF1		0.89333
MUS81		0.96654
RCN3		0.94529
C17orf67		0.98881
CD8A		0.97647
DHFR		0.98079
SLC25A34		0.99084
VSIG10		0.89591
ZC3H12D		0.90297

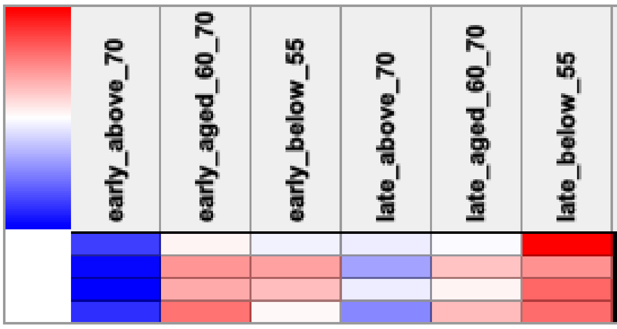

Probe ID	Description	Correlation
KCNK1		0.89458
HRC		0.95784
FAM71E1		0.97756
SPHK1		0.95447

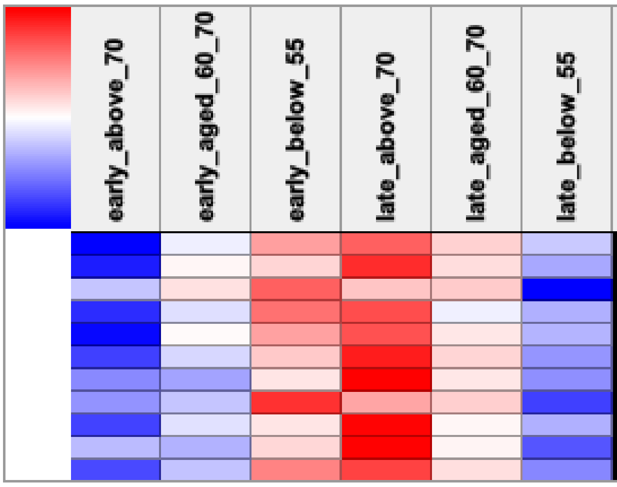

Probe ID	Description	Correlation
KIF22		0.93750
FAM136A		0.95575
KIAA0319		0.71347
NCAM1		0.96189
UQCRQ		0.95670
ACSL5		0.98390
BRF2		0.92193
FBP1		0.84615
NACA		0.94845
SAPCD2		0.89152
ZDHHC13		0.99234

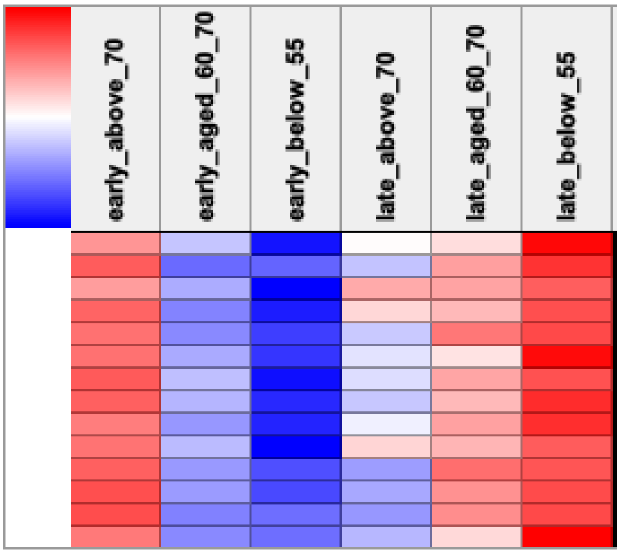

Probe ID	Description	Correlation
BHLHE40		0.95961
CLIC5		0.97577
DBP		0.91254
NEK11		0.97081
TTL		0.98434
C19orf38		0.97889
XPO6		0.98775
HGF		0.99316
IL13RA1		0.99668
IL13RA2		0.95460
ITK		0.96153
PTPRC		0.97874
SAMHD1		0.95451
PFKFB2		0.95253

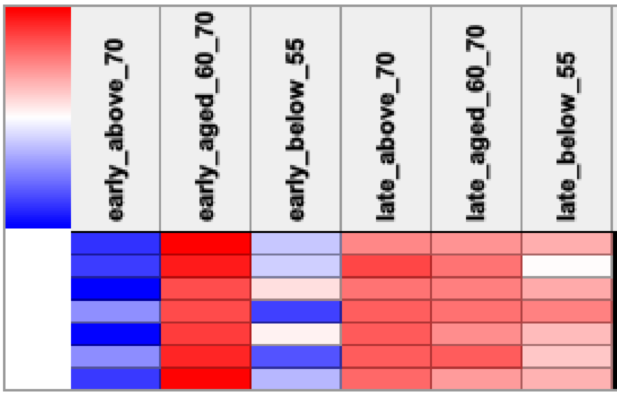

Probe ID	Description	Correlation
ID1		0.98511
TP73		0.97509
CCDC12		0.92997
CYBA		0.90017
PHLDA3		0.95549
S100A4		0.93197
S100A6		0.99287

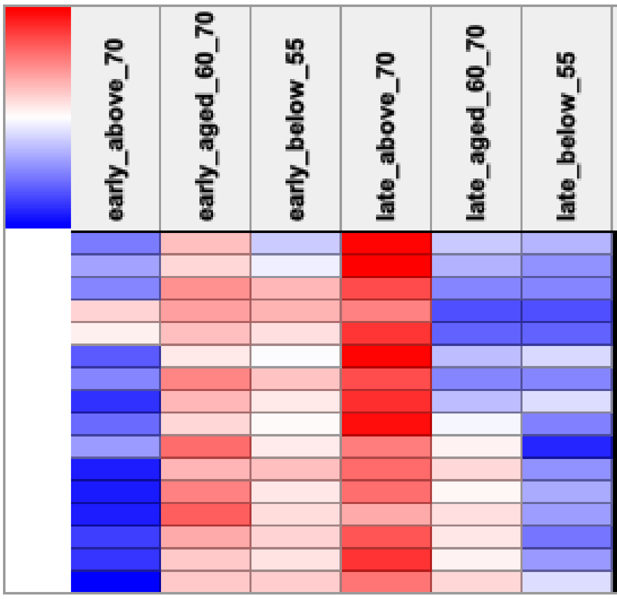

Probe ID	Description	Correlation
CISH		0.92900
ADTRP		0.92414
BBOX1		0.94868
CPLX2		0.63035
DUSP9		0.75843
HOXA5		0.91599
KCNK2		0.94945
RASSF7		0.94770
EPHB4		0.96727
HSF4		0.87253
RPL11		0.91919
RPL18		0.93470
RPL9		0.85197
RPS23		0.95949
RPS24		0.96297
STYXL1		0.84030

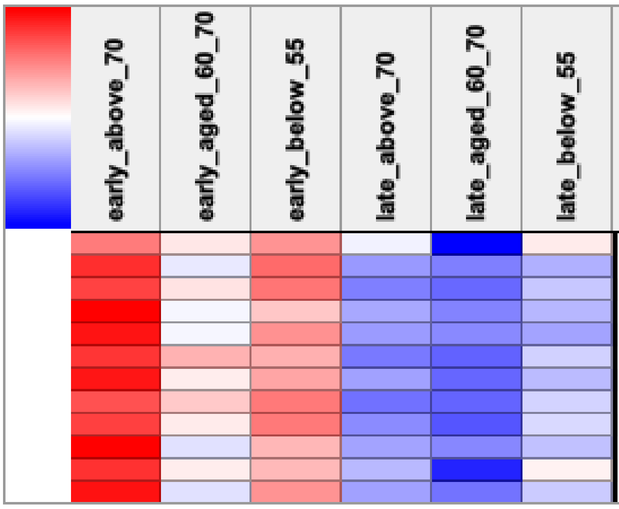

Probe ID	Description	Correlation
ASXL3		0.85121
CCDC25		0.97436
INHBE		0.98486
KLF4		0.96352
PEBP4		0.97696
SFTPD		0.96680
CD38		0.99476
PRRG4		0.96938
PYGB		0.99105
SNPH		0.96557
ST8SIA6		0.95246
ZG16B		0.98575

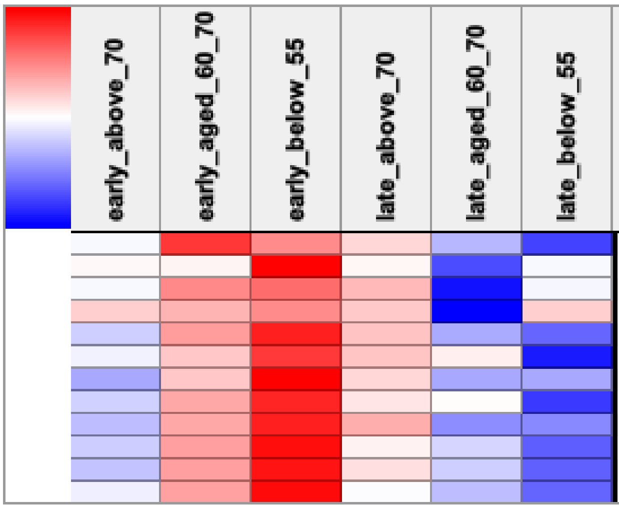

Probe ID	Description	Correlation
C12orf57		0.85894
ATP8A2		0.82854
BEX1		0.79534
C4orf19		0.60097
EBPL		0.99192
HHIPL2		0.84883
INA		0.95624
KIAA0895L		0.90546
MAL		0.98372
NOL3		0.96353
NYAP1		0.97145
SMTN		0.97212

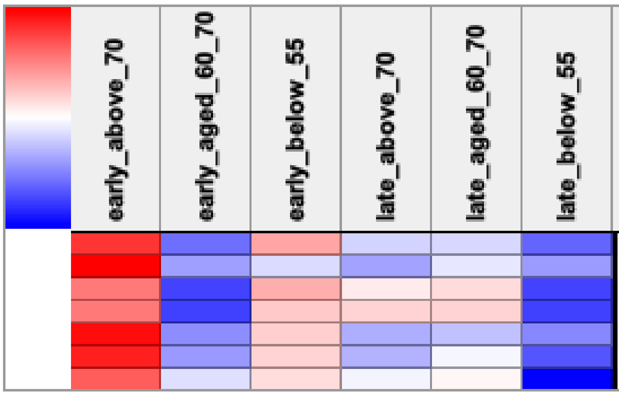

Probe ID	Description	Correlation
LEPROTL1		0.97980
PLCB4		0.88880
ASTN1		0.92411
DDX25		0.90597
ENDOD1		0.94799
GNAI1		0.98485
GPR158		0.89952

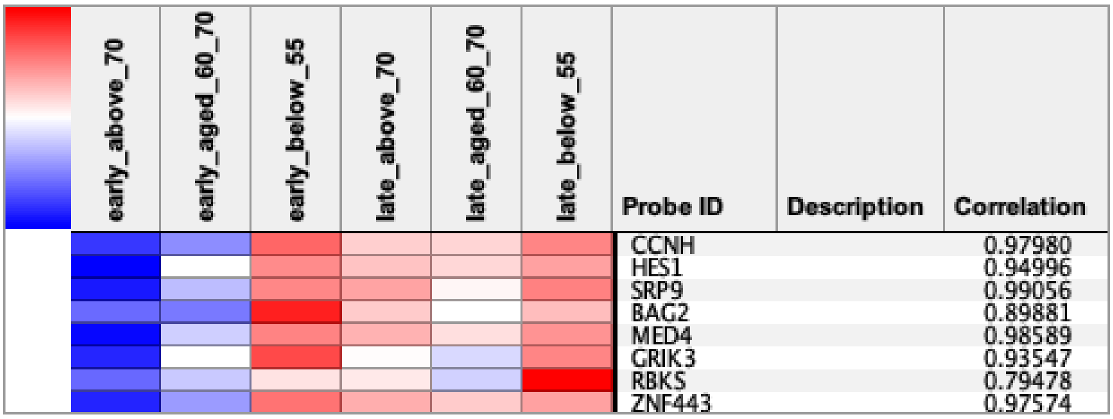
early_above_70
early_aged_60_70
early_below_55
late_above_70
late_aged_60_70
late_below_55
Probe ID
Description
Correlation
CCNH 0.97980
HES1 0.94996
SRP9 0.99056
BAG2 0.89881
MED4 0.98589
GRIK3 0.93547
RBKS 0.79478
ZNF443 0.97574

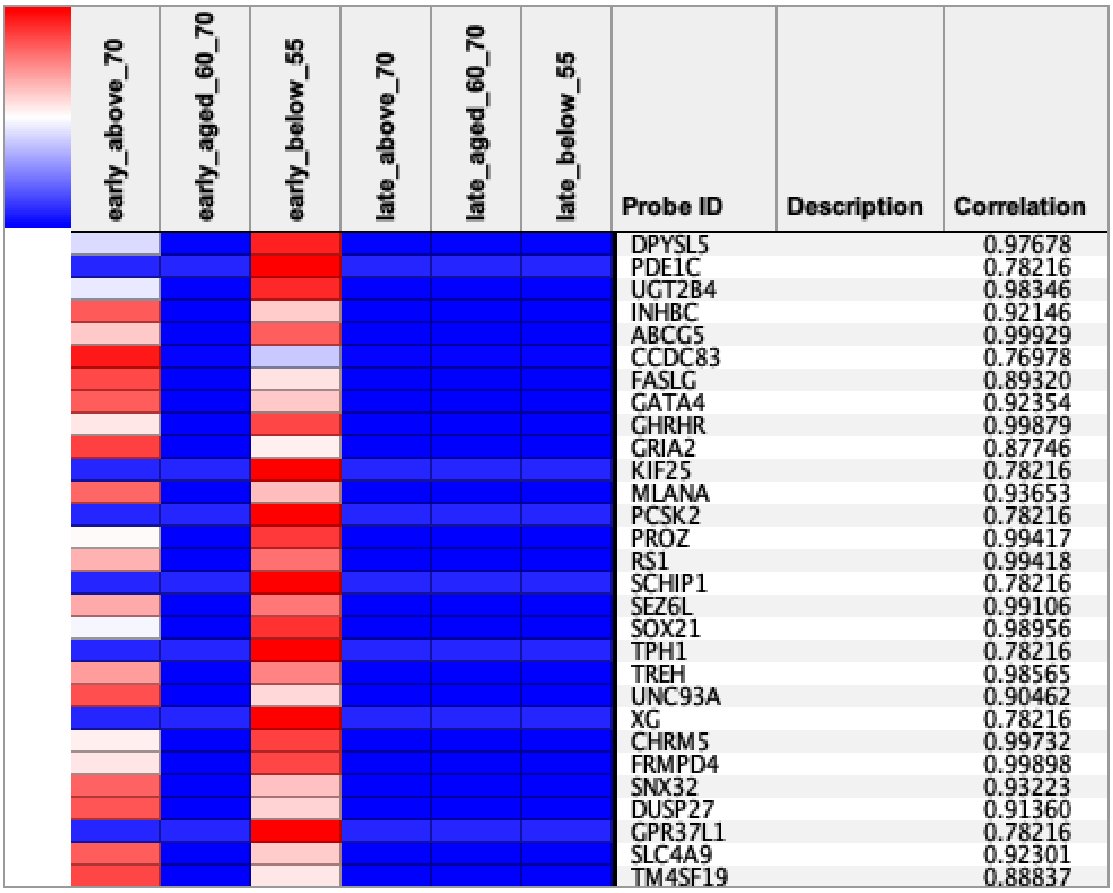
early_above_70
early_aged_60_70
early_below_55
late_above_70
late_aged_60_70
late_below_55
Probe ID
Description
Correlation
DPYSL5 0.97678
PDE1C 0.78216
UGT2B4 0.98346
INHBC 0.92146
ABCG5 0.99929
CCDC83 0.76978
FASLG 0.89320
GATA4 0.92354
GHRHR 0.99879
GRIA2 0.87746
KIF25 0.78216
MLANA 0.93653
PCSK2 0.78216
PROZ 0.99417
RS1 0.99418
SCHIP1 0.78216
SEZ6L 0.99106
SOX21 0.98956
TPH1 0.78216
TREH 0.98565
UNC93A 0.90462
XG 0.78216
CHRM5 0.99732
FRMPD4 0.99898
SNX32 0.93223
DUSP27 0.91360
GPR37L1 0.78216
SLC4A9 0.92301
TM4SF19 0.88837

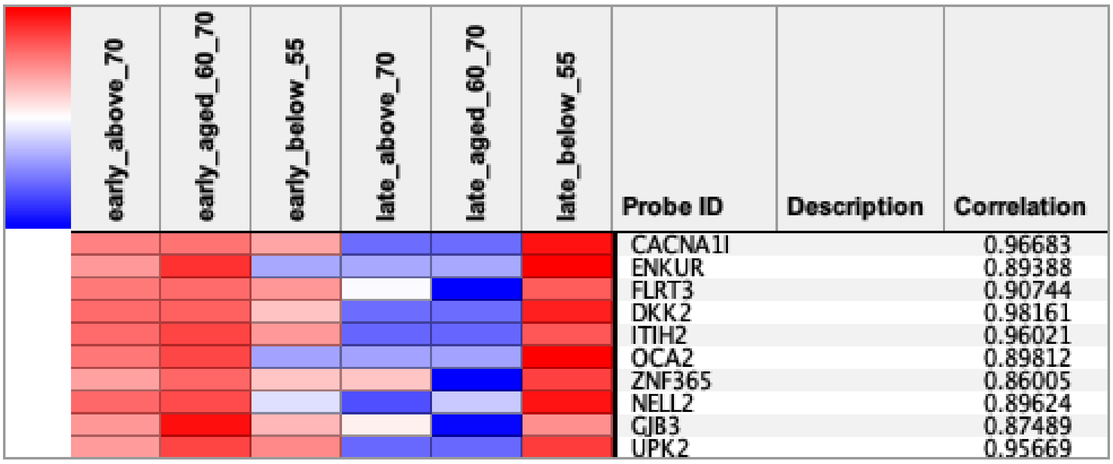
early_above_70
early_aged_60_70
early_below_55
late_above_70
late_aged_60_70
late_below_55
Probe ID
Description
Correlation
CACNA1I 0.96683
ENKUR 0.89388
FLRT3 0.90744
DKK2 0.98161
ITIH2 0.96021
OCA2 0.89812
ZNF365 0.86005
NELL2 0.89624
GJB3 0.87489
UPK2 0.95669

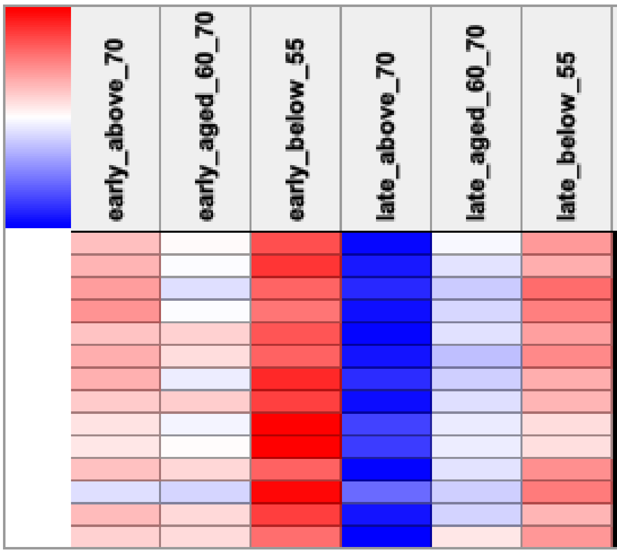

Probe ID	Description	Correlation
CACNA2D2		0.99357
IGF1R		0.99648
PLEKHB2		0.96129
TCTA		0.96702
TRIM5		0.98906
TP53INP2		0.98099
PACSIN2		0.98809
SLC2A9		0.98878
STEAP4		0.94575
XK		0.94937
ADAMTS9		0.98614
C11orf54		0.89457
CADM4		0.99238
SRL		0.96233

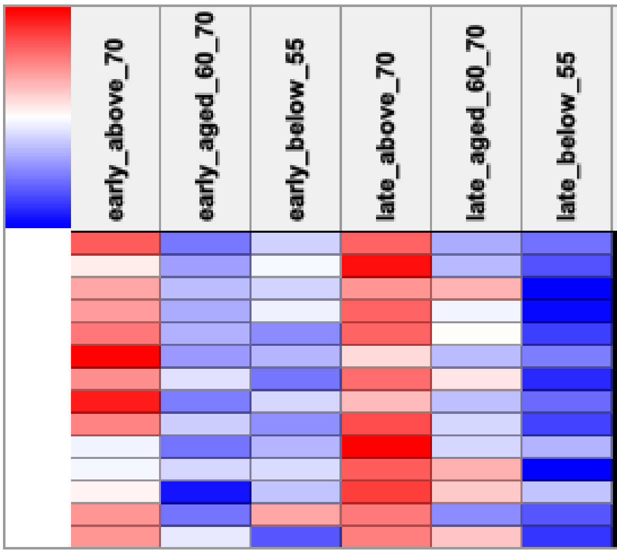

Probe ID	Description	Correlation
ERI1		0.94050
FHOD3		0.89621
GRAP2		0.89824
RPL17		0.97167
FAM186B		0.97796
FECH		0.78314
FGF7		0.93030
GALNT12		0.85779
LAMA1		0.96687
SYTL5		0.79811
ESCO2		0.82877
IQCB1		0.74985
KLHL32		0.79796
MYC		0.87320

II - Rubicz et al. data

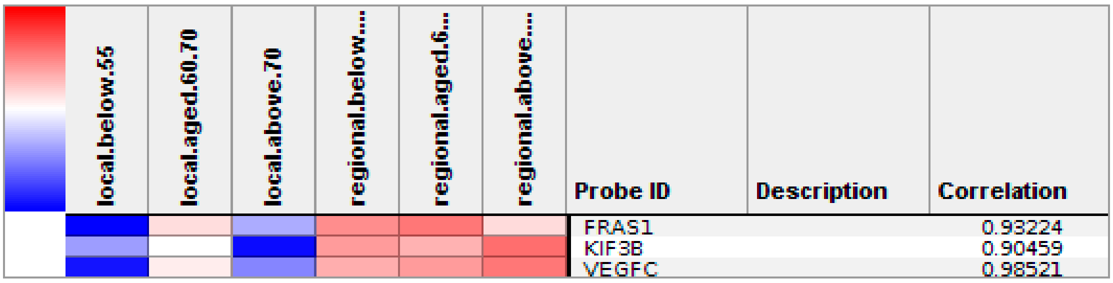

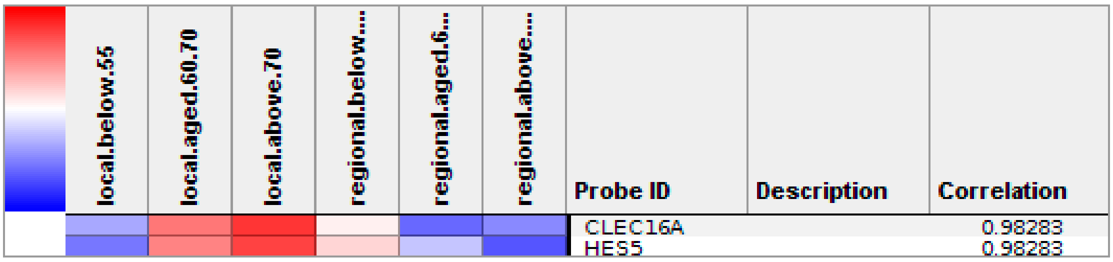

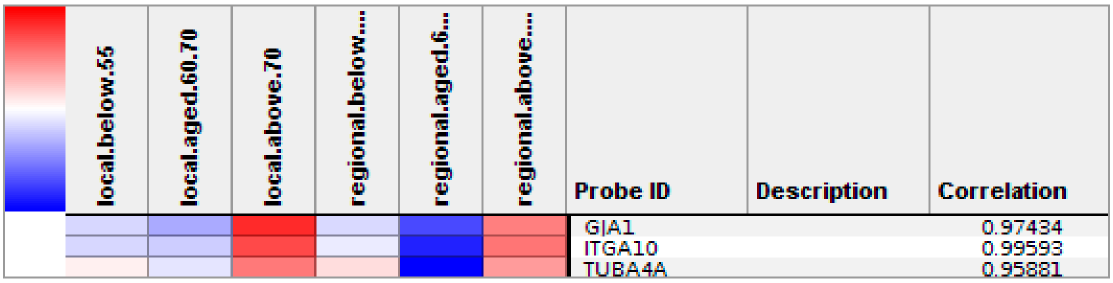

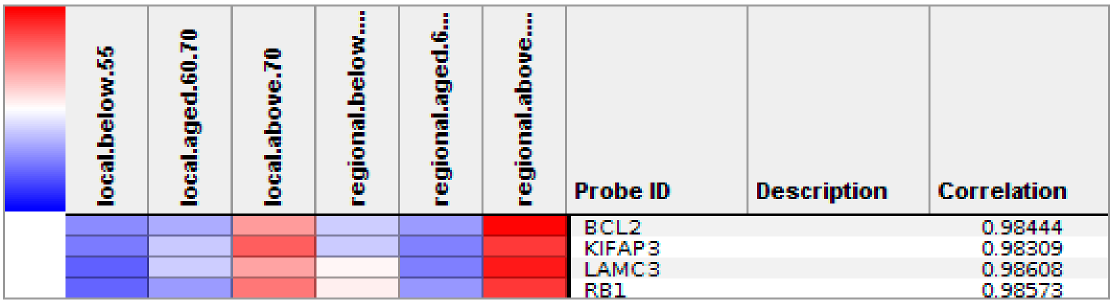

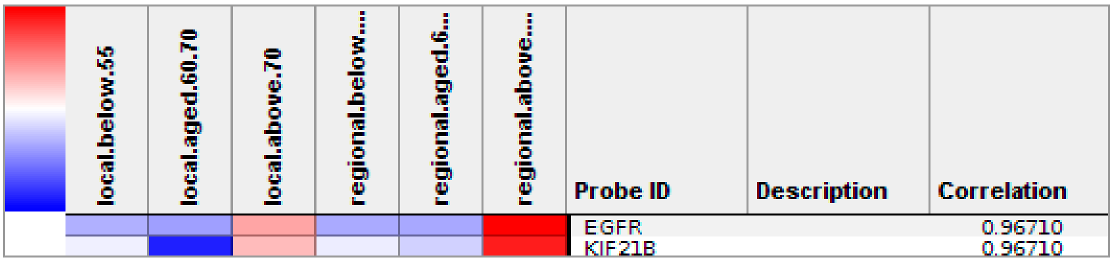

local.below.55	local.aged.60.70	local.above.70	regional.below....	regional.aged.6...	regional.above....	Probe ID	Description	Correlation
						MAP2		1.00000

local.below.55	local.aged.60.70	local.above.70	regional.below....	regional.aged.6...	regional.above....	Probe ID	Description	Correlation
						FCHO2		0.98727
						TCF4		0.98727

local.below.55	local.aged.60.70	local.above.70	regional.below....	regional.aged.6...	regional.above....	Probe ID	Description	Correlation
						ITGA6		0.99464
						NOTCH1		0.99464

local.below.55	local.aged.60.70	local.above.70	regional.below....	regional.aged.6...	regional.above....	Probe ID	Description	Correlation
						CDH11		0.98818
						COCH		0.91429
						COL8A1		0.89229

local.below.55	local.aged.60.70	local.above.70	regional.below....	regional.aged.6...	regional.above....	Probe ID	Description	Correlation
						ANK2		0.98122
						ANXA1		0.74328
						CDKN1A		0.91938
						COL14A1		0.92732
						DYNC1H1		0.94493
						FN1		0.90043
						HES4		0.97251
						HIF1A		0.95195
						KIF3A		0.91419

local.below.55	local.aged.60.70	local.above.70	regional.below....	regional.aged.6...	regional.above....	Probe ID	Description	Correlation
						ITGA8		0.97234
						MYO1F		0.97790
						TIP3		0.95555

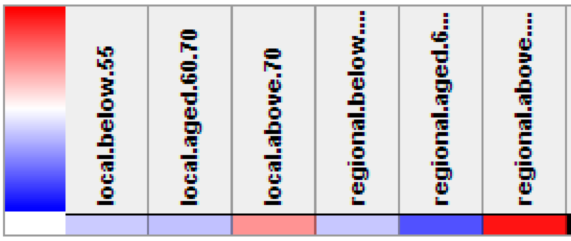

Probe ID	Description	Correlation
ITGB2		1.00000

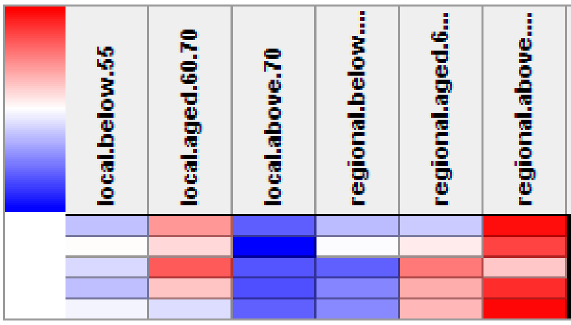

Probe ID	Description	Correlation
CDHR2		0.91421
HEY1		0.91499
ICAM1		0.80495
ICAM5		0.98612
MATN1		0.92201

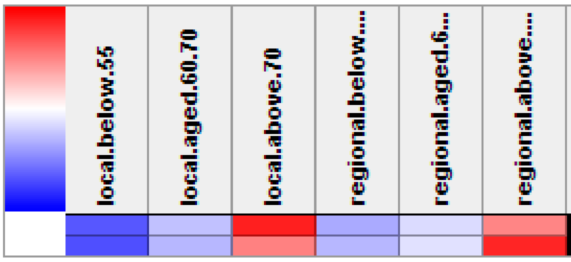

Probe ID	Description	Correlation
DNMT1		0.97973
VEGFA		0.97973

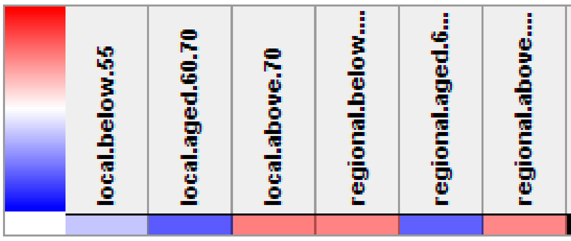

Probe ID	Description	Correlation
LAMA1		1.00000

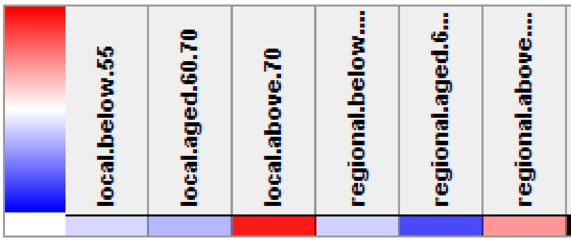

Probe ID	Description	Correlation
TP63		1.00000

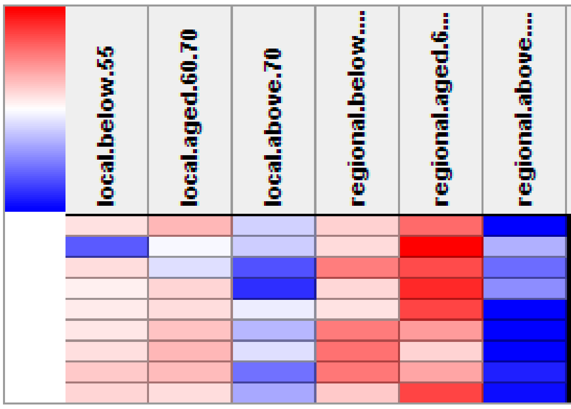

Probe ID	Description	Correlation
CDHR5		0.95959
CDK12		0.67174
FLG		0.90666
FLNB		0.86871
GATA1		0.93989
HDAC1		0.95630
HEY2		0.86966
KIF21A		0.93829
MYO1D		0.98597

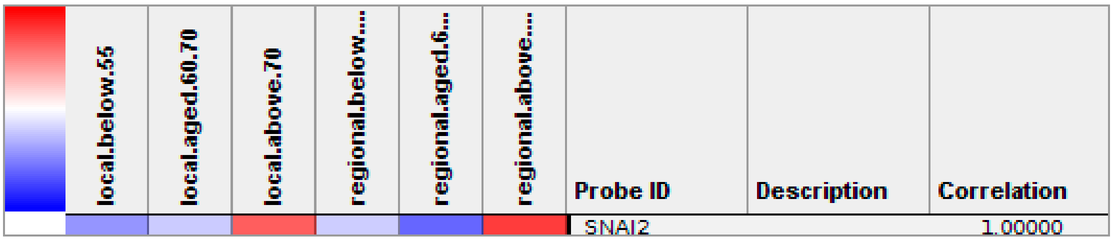
local.below.55
local.aged.60.70
local.above.70
regional.below....
regional.aged.6...
regional.above....
Probe ID
Description
Correlation
SNAI2 1.00000

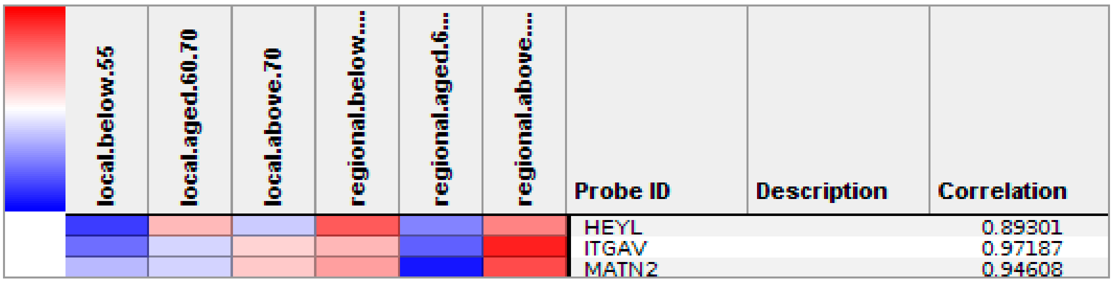
local.below.55
local.aged.60.70
local.above.70
regional.below....
regional.aged.6...
regional.above....
Probe ID
Description
Correlation
HEYL 0.89301
ITGAV 0.97187
MATN2 0.94608

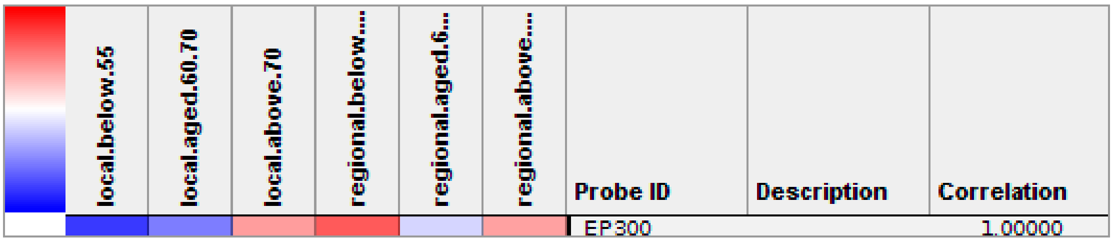
local.below.55
local.aged.60.70
local.above.70
regional.below....
regional.aged.6...
regional.above....
Probe ID
Description
Correlation
EP300 1.00000

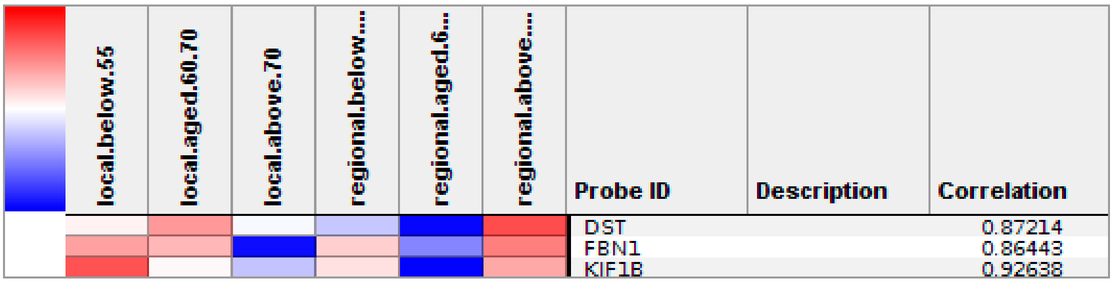
local.below.55
local.aged.60.70
local.above.70
regional.below....
regional.aged.6...
regional.above....
Probe ID
Description
Correlation
DST 0.87214
FBN1 0.86443
KIF1B 0.92638

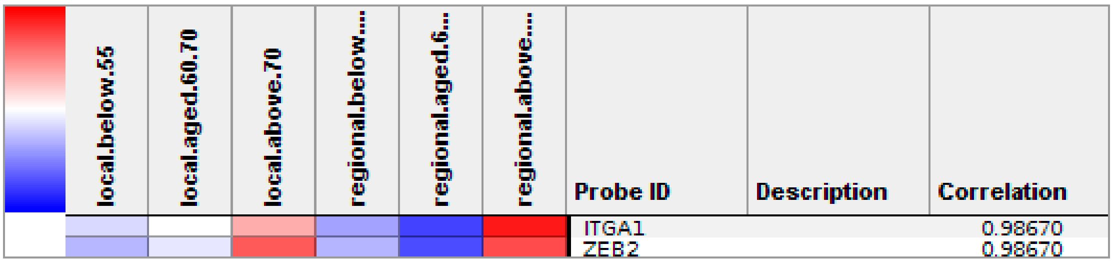
local.below.55
local.aged.60.70
local.above.70
regional.below....
regional.aged.6...
regional.above....
Probe ID
Description
Correlation
ITGA1 0.98670
ZEB2 0.98670

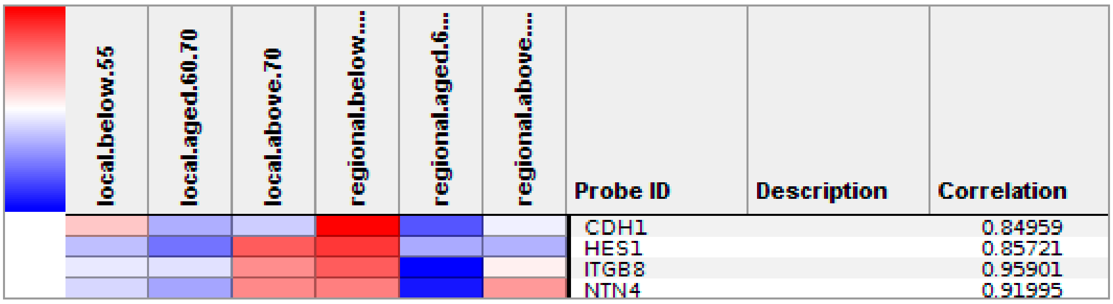
local.below.55
local.aged.60.70
local.above.70
regional.below....
regional.aged.6...
regional.above....
Probe ID
Description
Correlation
CDH1 0.84959
HES1 0.85721
ITGB8 0.95901
NTN4 0.91995

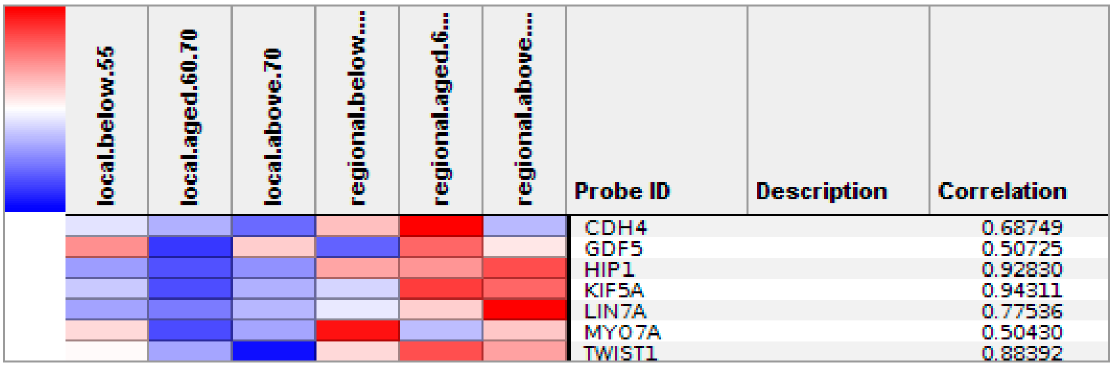
local.below.55
local.aged.60.70
local.above.70
regional.below....
regional.aged.6...
regional.above....
Probe ID
Description
Correlation
CDH4 0.68749
GDF5 0.50725
HIP1 0.92830
KIF5A 0.94311
LIN7A 0.77536
MYO7A 0.50430
TWIST1 0.88392

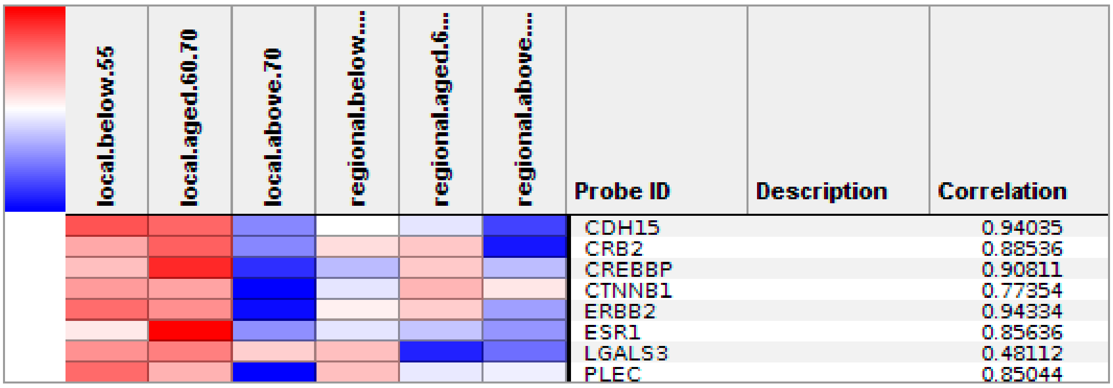
local.below.55
local.aged.60.70
local.above.70
regional.below....
regional.aged.6...
regional.above....
Probe ID
Description
Correlation
CDH15 0.94035
CRB2 0.88536
CREBBP 0.90811
CTNNB1 0.77354
ERBB2 0.94334
ESR1 0.85636
LGALS3 0.48112
PLEC 0.85044

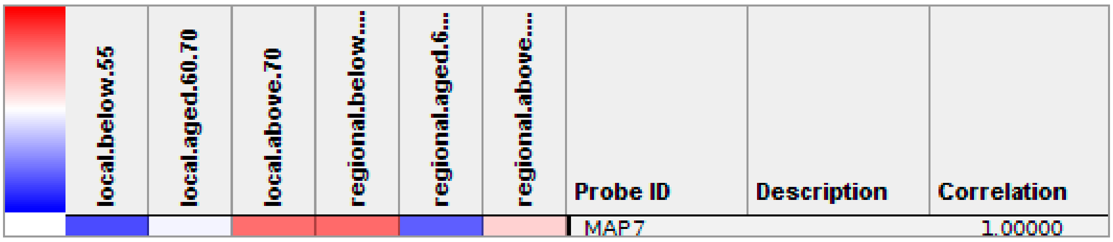
local.below.55
local.aged.60.70
local.above.70
regional.below....
regional.aged.6...
regional.above....
Probe ID
Description
Correlation
MAP7 1.00000

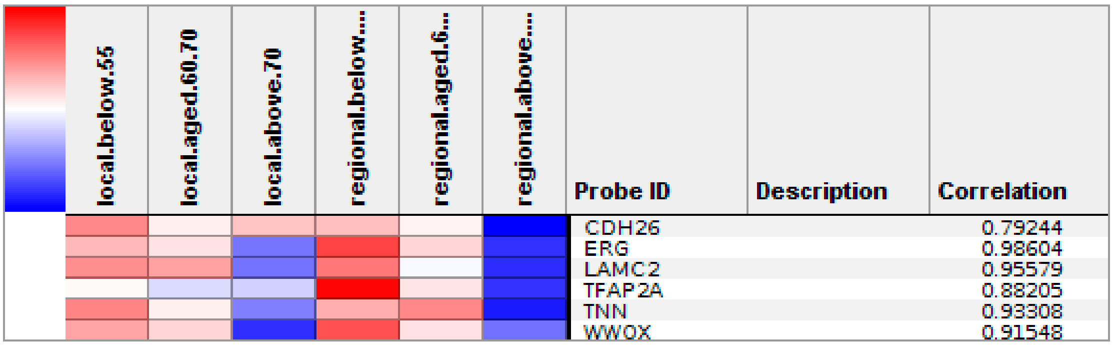
local.below.55
local.aged.60.70
local.above.70
regional.below....
regional.aged.6...
regional.above....
Probe ID
Description
Correlation
CDH26 0.79244
ERG 0.98604
LAMC2 0.95579
TFAP2A 0.88205
TNN 0.93308
WWOX 0.91548

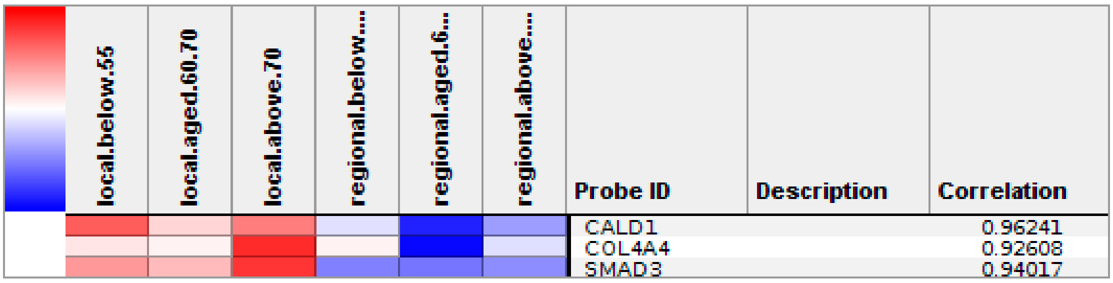
local.below.55
local.aged.60.70
local.above.70
regional.below....
regional.aged.6...
regional.above....
Probe ID
Description
Correlation
CALD1 0.96241
COL4A4 0.92608
SMAD3 0.94017

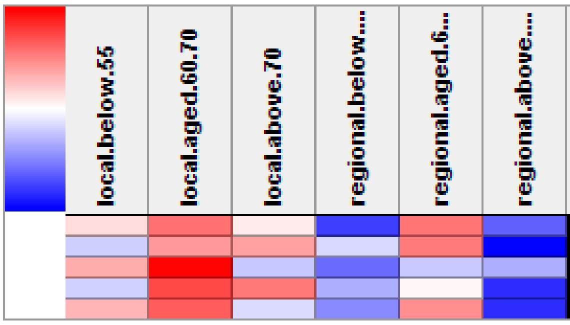

Probe ID	Description	Correlation
CTNNA2		0.94178
FGF9		0.85542
HDAC6		0.76529
MYC		0.89441
NPNT		0.95056

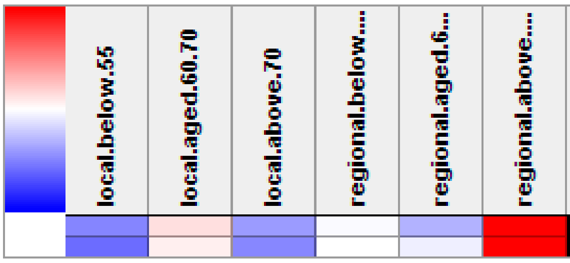

Probe ID	Description	Correlation
FGF1		0.99370
MAPRE3		0.99370

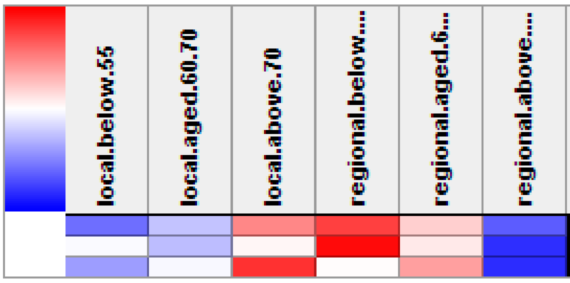

Probe ID	Description	Correlation
CDK19		0.96837
FGF12		0.86019
MZT1		0.83596

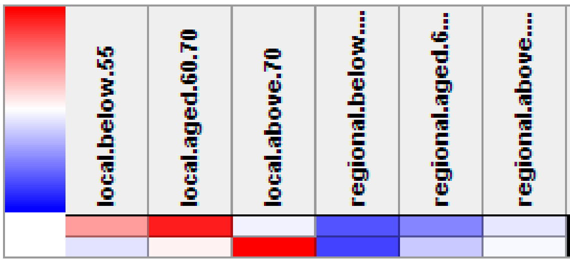

Probe ID	Description	Correlation
COL6A2		0.81924
MMP7		0.81924
